# Functionalized Tetrazoles as Latent Active Esters in the Synthesis of Amide Bonds

**DOI:** 10.1021/acs.orglett.2c03971

**Published:** 2022-12-16

**Authors:** Jessica M. L. Elwood, Martyn C. Henry, J. Daniel Lopez-Fernandez, Jenna M. Mowat, Mhairi Boyle, Benjamin Buist, Keith Livingstone, Craig Jamieson

**Affiliations:** Department of Pure and Applied Chemistry, University of Strathclyde, 295 Cathedral Street, Glasgow G1 1XL, U.K.

## Abstract

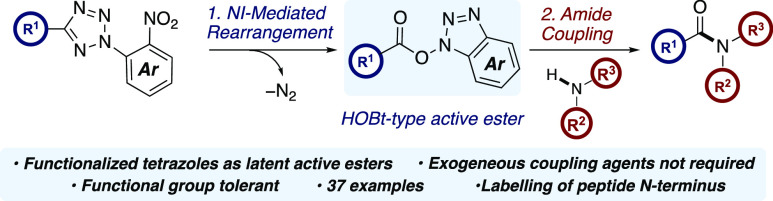

We report the use of *N*-2,4-dinitrophenyltetrazoles as latent active esters (LAEs) in the synthesis of amide bonds. Activating the tetrazole generates an HOBt-type active ester without the requirement for exogenous coupling agents. The methodology was widely applicable to a range of substrates, with up to quantitative yields obtained. The versatility and functional group tolerance were exemplified with the one-step synthesis of various pharmaceutical agents and the *N*-acylation of resin-bound peptides.

The importance of the amide bond cannot be over emphasized, with this moiety comprising the backbone of peptides, proteins, and a host of biomolecules critical to the function of life. Furthermore, it is estimated that amidation methods account for 25% of all reactions carried out in a drug discovery setting.^1^ Significant efforts have been directed toward the development of novel and efficient amidation methodologies which has led to an armamentarium of coupling protocols being currently used.^2^ These principally employ electrophilic carboxylic acid derivatives through the addition of stoichiometric quantities of an activating or coupling reagent (Scheme 1a). However, recent safety concerns associated with commonly used coupling agents, in addition to the often poor atom economy of these processes, have increased interest in new methods for amide bond formation.^3^ Accordingly, novel amidation protocols have been reported in recent years which avoid the use of toxic, sensitizing, and atom inefficient coupling reagents.^4^ These methods include the amidation of carboxylic acids via the corresponding acyl fluorides^5^ or by the direct condensation of unactivated carboxylic acids with amines using silicon reagents^6^ or utilizing boron,^7^ transition metal,^8^ or photoredox catalysis.^9^ Alternative strategies involve the direct amidation of esters,^10^ the oxidative amidation of aldehydes,^11^ or palladium-catalyzed carbonylation of aryl halides.^12^

**Scheme 1:**
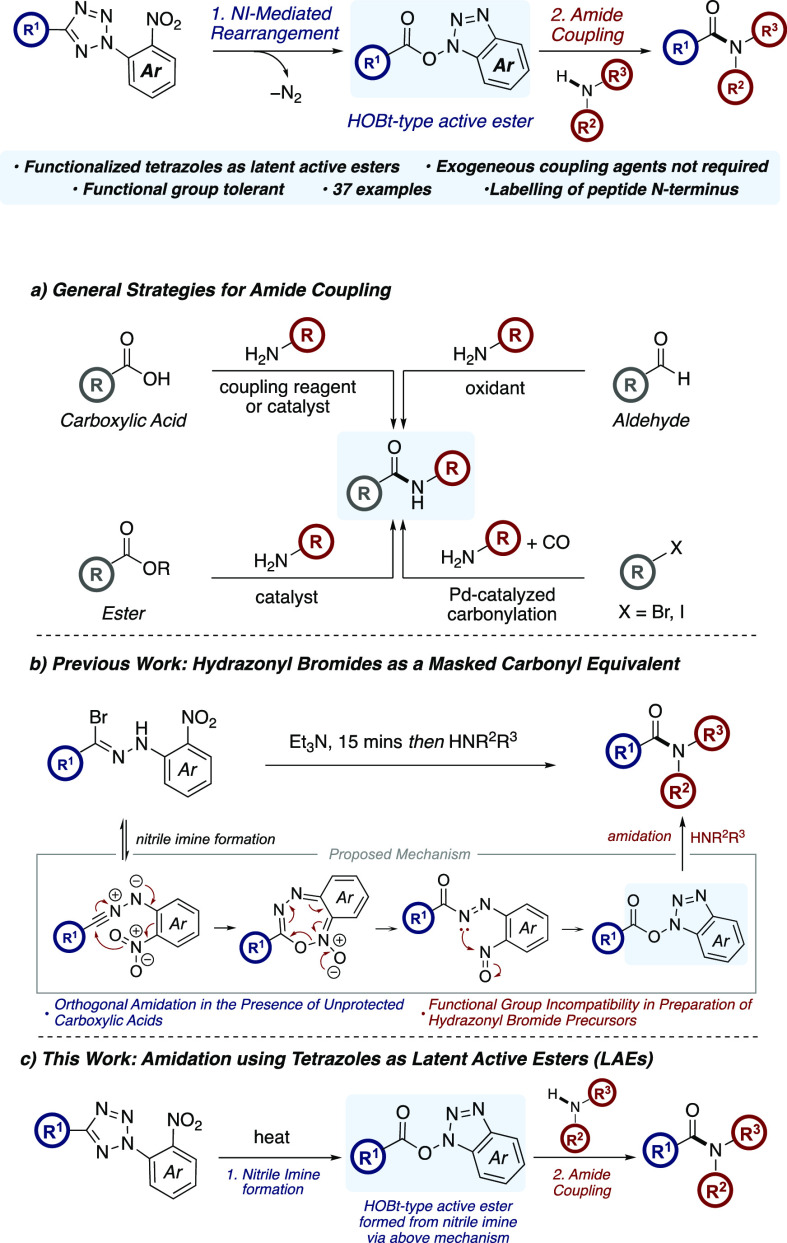
General Amide Bond Forming Strategies and Proposed Study

During our recent investigations into the applicability of nitrile imines (NIs) in organic synthesis,^13^ we noted a rearrangement of NIs bearing a 2-nitrophenyl motif at the *N*-terminus that was reported in the late 1960s,^14^ where a 1,7-electrocyclisation occurs between the NI 1,3-dipole and the ancillary *ortho*-nitro group, which results in the formation of an *N*-hydroxybenzotriazole activated ester. The utility of this was not fully appreciated at the time due to the proclivity of this species to hydrolyze to the corresponding carboxylic acid.

Recently, we exploited this observation for the *in situ* preparation of an HOBt-type active ester via the base-mediated dehydrohalogenation of hydrazonyl bromides and leveraged this for amide bond formation in a one-pot process (Scheme 1b).^15^ This method was highly effective for the preparation of a range of amides, including the orthogonal *N*-acylation of unprotected amino acids under mild conditions.

The versatility and general applicability of this methodology notwithstanding, a significant limitation did arise regarding the preparation of the requisite 2-nitrophenyl-substituted hydrazonyl bromide substrates. These were synthesized from the corresponding aldehyde via acid-mediated condensation with 2-nitrophenylhydrazine and subsequent bromination employing molecular bromine. In addition to the associated toxicity and safety concerns associated with the use of elemental bromine, the highly reactive nature of this necessarily precluded the application of aldehydes bearing electron-rich arenes, with either undesired overbromination or substrate decomposition observed under harsh oxidative conditions.

In the current study, we report how *N*-aryl substituted tetrazoles bearing an *ortho*-nitro substituent may serve as *latent active esters* (LAEs) (Scheme 1c), obviating the need for oxidizing conditions. Upon activation by an appropriate external stimulant, the tetrazole substrates will efficiently generate the NI intermediate which will undergo rearrangement to the HOBt-type active ester shown in Scheme 1b. The desired amide bond can then be formed by trapping this intermediate with an amine, with no requirement for exogeneous coupling reagents.

Our study began with the preparation of the *N*-2,4-dinitrophenyltetrazole substrates. This was achieved via a nucleophilic aromatic substitution reaction between phenyl tetrazole and fluoro-2,4-dinitrobenzene in the presence of triethylamine (Scheme 2a) furnishing the desired tetrazole adduct **1a**.^16^ This S_N_Ar procedure was a very effective approach toward the synthesis of *N*-functionalized tetrazoles and was tolerant of a wide-range of electron-rich, electron-poor (hetero)aromatic and alkyl *NH-*tetrazoles, thus significantly expanding the scope of our progenitor process requiring the preparation of hydrazonyl bromides^15^ (see the Supporting Information).

**Scheme 2:**
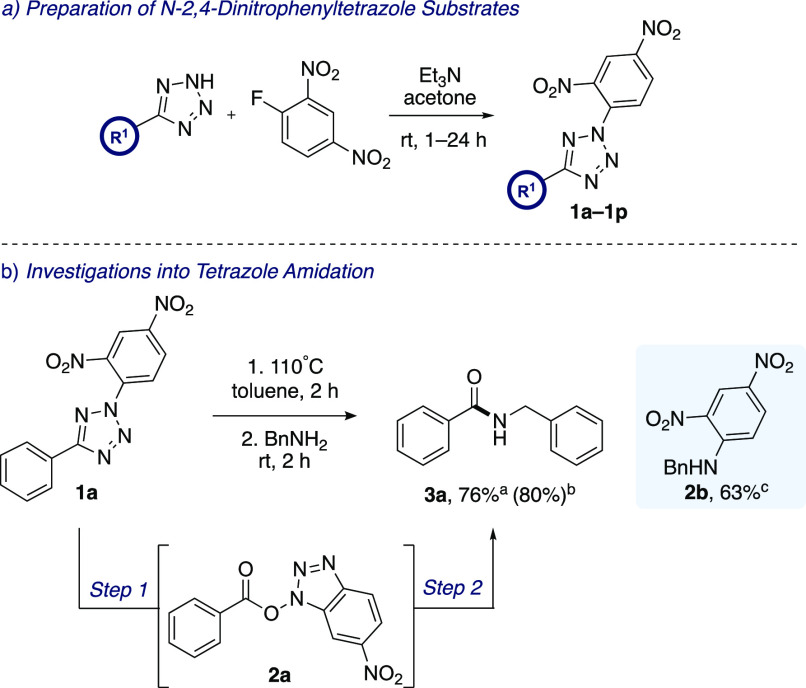
Preparation of Substrates and Tetrazole Amidation Isolated yield. Reaction was performed on a 3.3 mmol scale. S_N_Ar adduct **2b** forms in 63% yield in the absence of an activation period as determined by ^1^H NMR spectroscopy with 1,3,5-trimethoxybenzene as an internal standard (see the Supporting Information).

With a palette of *N*-functionalized tetrazoles in hand, the amidation reaction was investigated (Scheme 2b). After an unprotracted optimization, it was found that an activation period was required during the thermolysis of the tetrazole **1a** to allow the generation of the NI-intermediate and subsequent rearrangement to active ester **2a**. Stirring **1a** in toluene at 110 °C for 2 h was sufficient to allow full conversion to active ester **2a**, which was then treated with benzylamine to afford amide **3a** in 76% yield.^17^ In the absence of this activation period, no desired amide **3a** was observed. Instead, nucleophilic displacement of the tetrazole with benzylamine led to the formation of arylamine **2b** in 63% yield. Tetrazole **1a** was then subjected to the thermolysis/amidation conditions on a 3.3 mmol scale which gave amide **3a** in 80% yield.

The scope of the amine was next explored using *N*-2,4-dinitrophenyltetrazole 1a (Scheme 3). A range of primary amines were *N*-acylated to give amides **3a**–**3i** in excellent yields. Interestingly, the presence of an α-methyl group did not affect the reaction outcome and the isolation of amide **3b** was achieved in 89% yield.

**Scheme 3:**
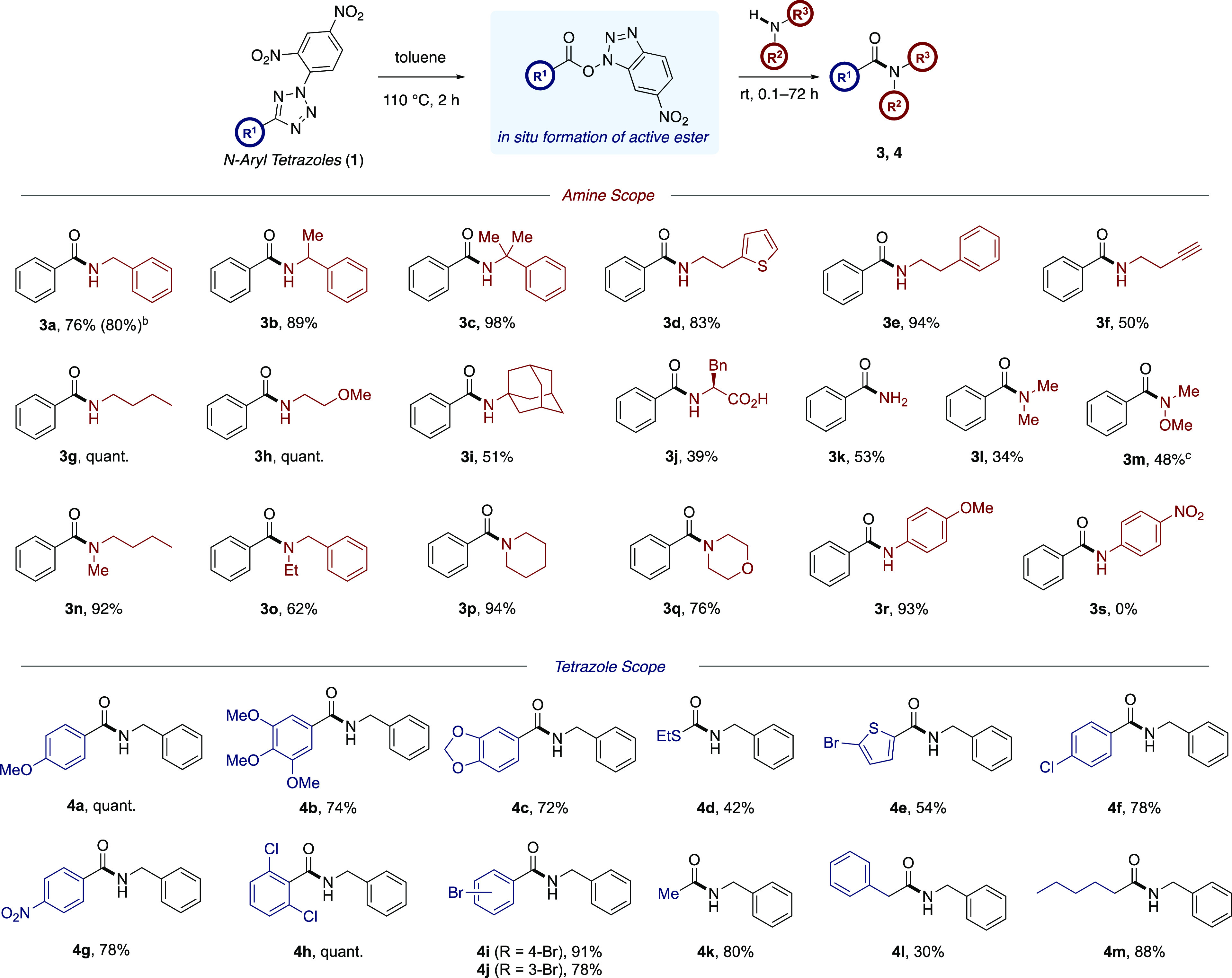
Exploration of Amine and Tetrazole Substrates Isolated yields. Reaction was performed on a 3.3 mmol scale One equiv of Et_3_N was added to the reaction mixture during the amidation step as the hydrochloride salt of *N*,*O*-dimethylhydroxylamine was used.

Similarly, amide **3c**, which features a sterically bulky *gem*-dimethyl group, was isolated in an excellent 98% yield. This contrasts with our previous procedure utilizing hydrazonyl bromides as NI precursors, in which a *gem*-dimethyl group severely hindered the reaction and gave the *N*-acylated product in only 31% yield.^15^ Thiophene-2-ethylamine and phenylethylamine were efficiently *N*-acylated under our conditions to afford amides **3d** and **3e** in 83% and 94% yield, respectively. Other primary amines were successfully employed in the reaction to give amides **3f**, **3g**, and **3h** in excellent yields. It is worth noting that in most cases, upon completion of the reaction of the amine with *in situ* generated active ester **2a**, the hydroxybenzotriazole byproduct was simply collected by filtration, and an aqueous workup was sufficient to isolate the desired amide in excellent yield and purity. In the case of amide **3i**, a combination of steric hindrance and the requirement for flash column chromatography led to a diminished yield of 51%. We also sought to apply the methodology to the selective *N*-acylation of the proteinogenic amino acid *L*-phenylalanine. The amidation reaction was successful and gave *N*-acyl derivative **3j** in 39% yield in the presence of an unprotected carboxylic acid group. After the generation of **2a***via* activation of **1a**, aqueous ammonia was used as an amine source and gave primary amide **3k** in 53% yield. Similarly, using dimethylamine and *N*,*O*-dimethylhydroxyamine as *N*-nucleophiles afforded **3l** and Weinreb amide **3m** in acceptable yields. It was reasoned that the lower yields obtained in these cases may be due to loss of material during basic/acidic aqueous workup. More challenging aliphatic and cyclic secondary amines were also examined. *N*-Methylbutylamine underwent effective *N*-acylation and afforded amide **3n** in 92% yield. For compound **3o**, a more bulky secondary amine resulted in significant steric hindrance during the amidation step and resulted in a comparatively lower yield of 62%. Although an extended reaction time of up to 72 h was required, the use of cyclic secondary amines piperidine and morpholine were tolerated and gave amides **3p** and **3q** in 94% and 76% yield, respectively. 4-Methoxyaniline underwent *N*-acylation with active ester **2a**, and amide **3r** was obtained in 93% yield. By contrast, only trace quantities of amide **3s** were observed with electron-deficient 4-nitroaniline, even when heating the reaction at 110 °C for 48 h.

Using benzylamine as the *N*-nucleophile, the scope of the tetrazole was next examined (Scheme 3). Disubstituted tetrazole substrates featuring electron-rich aryl rings were well tolerated in the coupling process and afforded benzamides **4a**–**4c** in excellent yields. It should be noted that these substrates are not compatible with the progenitor process^15^ owing to their susceptibility to elemental bromine. Thiocarbamate **4d**, a compound isolated from the leaves of *Moringa oleifera*,^18^ was synthesized in 42% yield from the corresponding thioether-substituted tetrazole **1e**. Tetrazole **1f** featuring an electron-rich 2-bromothiophene moiety gave amide **4e** in 54% yield. Electron-deficient tetrazoles with *C*-terminal aryl rings bearing *ortho-*, *meta-*, and *para*- halogen or nitro-substituents were efficient substrates and allowed the synthesis of amides **4f**–**4j** in excellent isolated yields. Of particular interest was sterically demanding *ortho*-dichlorotetrazole **1i**, which was successfully applied in the process to afford amide **4h** in quantitative yield. In addition to aromatic tetrazoles, aliphatic tetrazole substrates **1l**–**1n** were examined. Tetrazole **1l**, with a simple methyl substituent, was subjected to the standard conditions and gave *N*-acylbenzamide (**4k**) in 80% yield. Other alkyl tetrazoles were also studied, with the synthesis of *N*-benzylphenylacetamide (**4l**) occurring in only 30% yield while amide **4m**, with a pentyl chain, was isolated in 88% yield.

This method was then applied to the synthesis of biologically relevant targets **5a**–**5f** (Scheme 4). Moclobemide (**5a**), a reversible monoamine oxidase inhibitor,^19^ and compound **5b**, a precursor to the sodium channel blocker procainamide (PCA),^20^ were synthesized in 76% and 65% yields, respectively. This represents a considerable improvement over our previous protocol employing the analogous hydrazonyl bromides, which furnished **5a** and **5b** in 51% and 21% yields, respectively.^15^ The thermal activation of electron-rich tetrazole **1o** afforded the corresponding active ester which was trapped with the required *para*-functionalized benzylamine nucleophile, which again would have been incompatible with the original reaction manifold. This afforded itopride, a combined D_2_ receptor antagonist and acetylcholinesterase inhibitor,^21^ in 72% yield in a single step. Similarly, tubulin inhibitor **5d**,^22^ featuring another electron-rich benzamide moiety, was synthesized in 53% yield by reaction with tetrazole **1c** and indoline. Moreover, tetrazole **1p** featuring a furazan heterocyclic motif was successfully coupled with piperidine to provide AMPA receptor modulator farampator (**5e**)^23^ in 60% yield. Finally, ALDA-1 (**5f**), an inhibitor of the human aldehyde dehydrogenase enzyme (ALDH2),^24^ was synthesized in 78% yield from the electron-deficient and sterically crowded 2,6-dichlorophenyltetrazole **1i**.

**Scheme 4:**
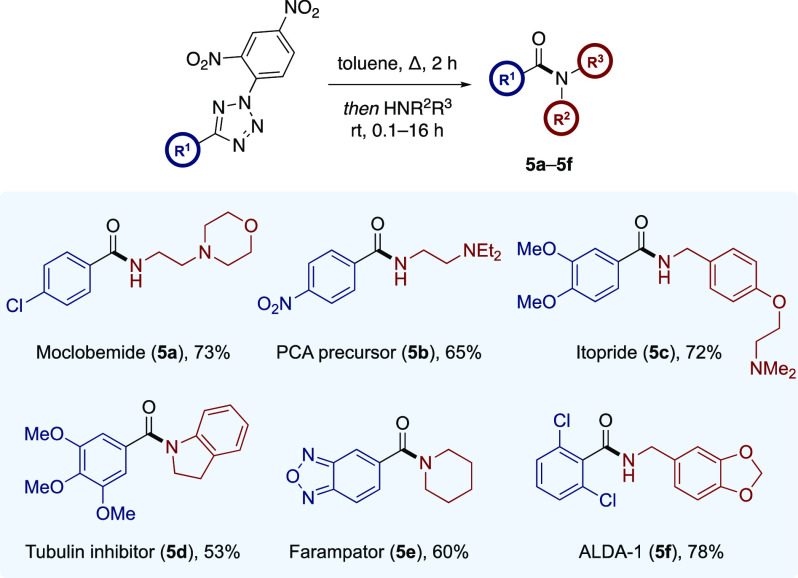
Synthesis of Biologically Active Agents

In the final stage of the study, we sought to demonstrate the value of our methodology by employing *N*-aryltetrazole LAEs in the context of peptide labeling *via* acylation of the *N*-terminus (Scheme 5). Model peptides leucine enkephalin **6** and substance P (**7**) were first prepared using standard SPPS methodology. Activation of tetrazole **1a** led to the formation of the corresponding HOBt-active ester **2a** which was coupled with resin-bound **6** to afford *N*-benzoylated peptide **8a** in 91% purity as determined by HPLC analysis. The method was next utilized for the introduction of a mixed isotope label to the *N*-terminus of peptides, as a probe in mass spectrometry. In this regard, an *M*+5 deuterated tetrazole was prepared *via* alkylation with deuterated methyl iodide followed by chelation-controlled, iridium-catalyzed C–H deuteration under a D_2_ atmosphere following the procedure of Kerr and co-workers.^25^ To provide the mixed isotope tetrazole ([D_0_]/[D_5_] = 1:1), the *M*+5 deuterated tetrazole was mixed with the nondeuterated counterpart in a 1:1 ratio. Heating this isotopic tetrazole mixture afforded the desired active ester which underwent near quantitative coupling with **6** to provide peptide **7b**, with a unique isotopic signature (*M*, *M*+5), in 92% purity. Finally, undecapeptide substance P (**7**) underwent successful acylation with tetrazole **1a**, which gave peptide **9** in 95% purity.

**Scheme 5:**
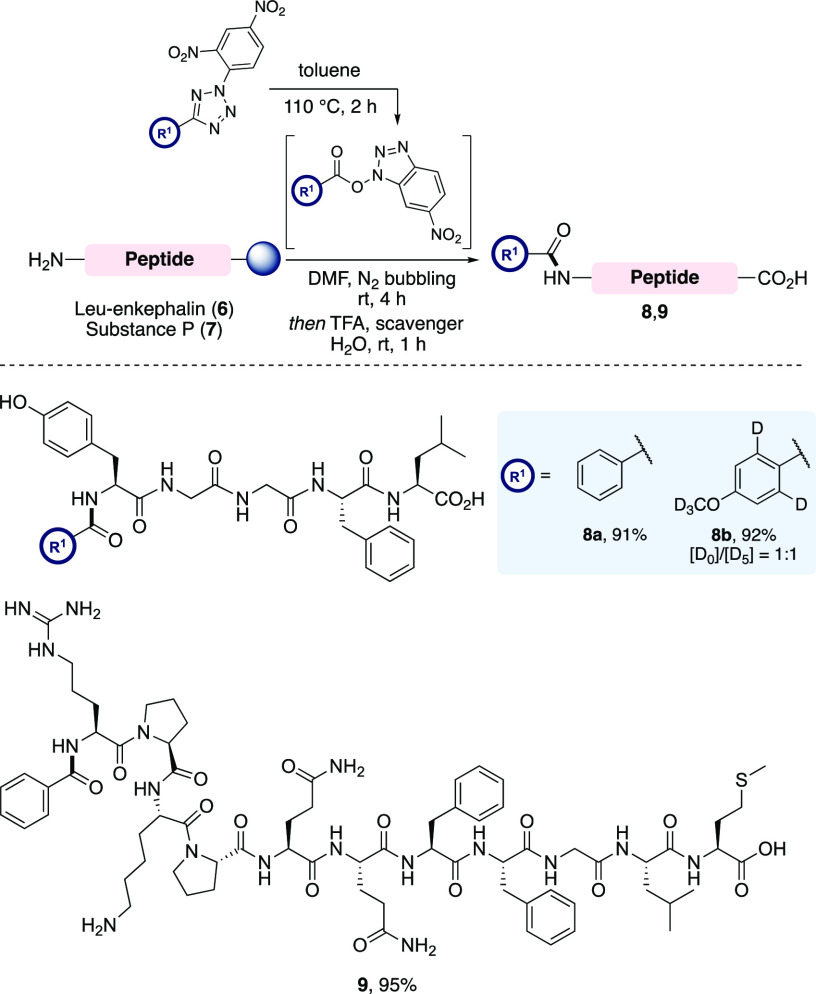
Peptide *N*-Terminal Labelling with Tetrazole LAEs

In summary, we have demonstrated that HOBt active esters may be generated from bench-stable tetrazole precursors using an external stimulus, thus enabling the concept of a latent active ester. The *N*-2,4-dinitrophenyltetrazole precursors were readily prepared using a nucleophilic aromatic substitution reaction which was widely tolerant of electron-rich aromatic functionality in contrast to our previous approach employing hydrazonyl bromides *via* electrophilic bromination. These tetrazoles were applied in the activation protocol affording the corresponding amides in excellent yields, often isolated by simple filtration and aqueous workup.

When leucine enkephalin and substance P were employed as *N*-nucleophiles, this methodology allowed the efficient capping of the peptide *N*-terminus. The orthogonal nature of this transformation is currently being investigated in the context of biomolecular labeling, alongside complementary approaches to activate the *N*-2,4-dinitrophenyltetrazole precursor.

## Data Availability

The data underlying this study are available in the published article and its Supporting Information.

## Supporting Information Available

Experimental procedures, characterization data, and NMR spectra of all compounds (PDF)
