# Supplementary material for: Functionalized Tetrazoles as Latent Active Esters in the Synthesis of Amide Bonds
Source: Org Lett. 2022 Dec 16;24(51):9491–6. doi: 10.1021/acs.orglett.2c03971 (PMC9806851; doi:10.1021/acs.orglett.2c03971)
Supplement: Supplementary file 1 — ol2c03971_si_001.pdf [file ol2c03971_si_001.pdf]

Supporting Information for:

Functionalized Tetrazoles as Latent Active Esters in the Synthesis of Amide Bonds

Jessica M. L. Elwood‡, Martyn C. Henry‡, J. Daniel Lopez-Fernandez‡,
Jenna M. Mowat, Mhairi Boyle, Benjamin Buist, Keith Livingstone and
Craig Jamieson*

Department of Pure and Applied Chemistry, University of Strathclyde, Glasgow G1 1XL, United Kingdom. Email: craig.jamieson@strath.ac.uk

Table of Contents

1. General Experimental S2
2. Experimental Procedures and Spectroscopic Data S4
3. General and Experimental Procedures for Peptide Synthesis S39
4. References S49
5. ^{1}H and ^{13}C NMR Spectra for all Compounds S51
6. HPLC Reports for Peptides S165

1. General Experimental

All reagents and starting materials were obtained from commercial sources and used as received without further purification, unless otherwise stated. Acetone, dichloromethane, ethyl acetate, methanol, petroleum ether 40–60 °C, and tetrahydrofuran were used as obtained from suppliers without further purification. All dry solvents were purified using a PureSolv SPS-400-5 Solvent Purification System.

All reactions were performed using round-bottom flasks or microwave vials of appropriate volume. Reactions were carried out at elevated temperatures using a temperature regulated hotplate/stirrer and DrySyn block with a contact thermometer, behind a blast shield where appropriate. Room temperature generally refers to ~ 20 °C. Reactions requiring a reduced temperature were performed using an ice bath (0 °C). Brine refers to a saturated aqueous solution of sodium chloride.

Reactions were monitored by thin layer chromatography (TLC) using Merck silica gel 60 covered aluminium backed plated F254. TLC plates were visualised under UV light and staining using potassium permanganate solution, vanillin or ninhydrin. Flash column chromatography was performed with silica gel 60 (40−63 μm). Reverse-phase HPLC purification of the *N*-acylated amino acid was conducted using a Gilson preparative HPLC system of 322 pumps coupled to a 151 UV/Vis 163 spectrometer, 234 Autoinjector and a GX-271 liquid handler using an Agilent Zorbax SB-C18 column (21.2 x 150 mm, 5 μm packing diameter) at room temperature. Purifications were performed using a gradient method ranging from 5–95 % MeCN in H_2O over 30 minutes at a flow rate of 15 mL/min, with a 0.1% TFA modifier and UV monitoring at 214 nm. Analysis was conducted using Gilson Trilution v2.0 software.

Infrared spectra were recorded on a FTIR spectrometer; wavenumbers are indicated in cm^{-1}. NMR spectras were recorded using dilute solutions in deuterated solvent on a Bruker NMR spectrometers at either 400 or 500 MHz using the deuterated solvent as the internal deuterium lock. ^{1}H chemical shift data are given as units δ relative to the residual protic solvent where δ ($CDCl_3$) = 7.26 ppm and (D_6-DMSO) = 2.50 ppm. ^{1}H signals are described as singlets (s), doublets (d), triplets (t), quartets (q), multiplets (m), broad (br), app (apparent) or a combination of these. ^{13}C chemical shift data were recorded at were recorded on Bruker NMR spectrometers at either 101 or 126 MHz and are given in units δ relative to the solvent where δ ($CDCl_3$) = 77.2 ppm and (D_6-DMSO) = 39.5 ppm.

High-resolution mass spectra were recorded using a Thermoscientific Exactive Plus equipped with a Vanquish LC. Low-resolution mass spectra were obtained using an Agilent Technologies 1200 series instrument with a 6130 single quadropole LC/MS using a poroshell EC-C18 column. Analysis was performed using a gradient method, eluting with 5–95% MeCN (containing 5nM ammonium acetate)/H_2O (containing 5nM ammonium acetate) over 18 minutes at a flow rate of 1 mL/min, with UV monitoring at 254 nm.

Optical rotation values were determined as solutions in methanol irradiating with the sodium D line (λ = 589 nm) using a PerkinElmer 341 polarimeter. $[\alpha]_D$ values are given in units 10^{-1} deg cm^2 g^{-1}.

2. Experimental Procedures and Spectroscopic Data

General Procedure A: Preparation of Tetrazoles[1]

To a stirred solution of nitrile (1.0 equiv.) in DMF (3 mL/mmol) was added sodium azide (1.1 equiv.) and ammonium chloride (1.1 equiv.). The resulting solution was heated at 110 °C for 20 hours. The reaction mixture was cooled to 0 °C, diluted with water and concentrated HCl was added dropwise until pH 1 was achieved. The resulting precipitate was collected, washed with water and dried under high vacuum. In the instances where the tetrazole product failed to precipitate, the mixture was diluted with EtOAc (20 mL) and washed with 2 M aqueous HCl (2 × 20 mL). The organic extract was dried over $MgSO_4$, filtered and concentrated *in vacuo*, to afford the desired tetrazole product.

5-(4-Methoxyphenyl)-2*H*-tetrazole (S1)

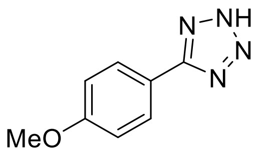

The reaction was performed as described in general procedure A using 4-methoxybenzonitrile (200 mg, 1.50 mmol). Recrystallisation from EtOH afforded 5-(4-methoxyphenyl)-2*H*-tetrazole (**S1**) (94.6 mg, 36%) as a colourless solid. ^{1}H NMR (400 MHz, D_6-DMSO) δ 7.98 (d, J = 8.8 Hz, 2H), 7.16 (d, J = 8.8 Hz, 2H), 3.84 (s, 3H); ^{13}C NMR (101 MHz, D_6-DMSO) δ 161.5, 152.9, 128.6 (2 × CH), 121.7, 114.8 (2 × CH), 55.4; LCMS (ESI) m/z: $[M - H]^-$ Calcd for $C_8H_7N_4O$ 175.1; Found 174.7 at 5.83 mins. This compound was known in the literature and the spectral data agreed with that of the reported values.[2]

5-(3,4,5-Trimethoxyphenyl)-2*H*-tetrazole (S2)

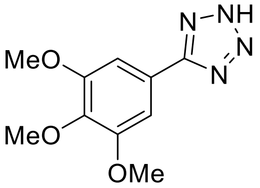

The reaction was performed as described in general procedure A using 3,4,5-trimethoxybenzonitrile (400 mg, 2.07 mmol) to afford 5-(3,4,5-trimethoxyphenyl)-2*H*-tetrazole

(**S2**) (487 mg, Quant.) as a grey solid. ^{1}H NMR (400 MHz, D_6-DMSO) δ 7.37 (s, 2H), 3.88 (s, 6H), 3.74 (s, 3H); ^{13}C NMR (101 MHz, D_6-DMSO) δ 153.5, 139.7, 104.4, 60.2, 56.1; LCMS (ESI) m/z: $[M + H]^+$ Calcd for $C_{10}H_{13}N_4O_3$ 237.1; Found 237.4 at 5.99 mins. This compound was known in the literature and spectral data are agreed with that of reported values.[3]

5-(Benzo[*d*][1,3]dioxol-5-yl)-2*H*-tetrazole (S3)

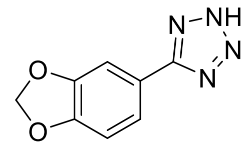

The reaction was performed as described in general procedure A using 3,4-(methylenedioxy)benzonitrile (400 mg, 2.72 mmol) to afford 5-(benzo[*d*][1,3]dioxol-5-yl)-2*H*-tetrazole (**S3**) (478 mg, 92%) as a grey solid. ^{1}H NMR (400 MHz, D_6-DMSO) δ 7.59 (d, *J* = 8.0 Hz, 1H), 7.54 (s, 1H), 7.09 (d, *J* = 8.0 Hz, 1H), 6.12 (s, 2H); ^{13}C NMR (101 MHz, D_6-DMSO) δ 155.9, 149.1, 148.0, 121.4, 119.2, 109.0, 106.8, 101.7; LCMS (ESI) m/z: $[M - H]^-$ Calcd for $C_8H_5N_4O_2$ 189.1; Found 189.2 at 5.77 mins. This compound was known in the literature and spectral data are agreed with that of reported values.[4]

5-Pentyltetrazole (S4)

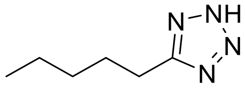

The reaction was performed as described in general procedure A using hexanenitrile (247 µL, 2.06 mmol). The reaction mixture was cooled and diluted with EtOAc (10 mL) then 6 M aqueous HCl (10 mL) was added slowly at 0 °C. The organic layer was separated and washed with 2 M aqueous HCl (2 × 20 mL) and dried over $MgSO_4$, filtered and concentrated *in vacuo* to afford 5-pentyltetrazole (**S4**) (129 mg, 45%) as a brown oil. ^{1}H NMR (400 MHz, $CDCl_3$) δ 3.09–3.02 (m, 1H), 2.34 (t, *J* = 7.3 Hz, 1H), 1.85 (quin., *J* = 7.3 Hz, 1H), 1.67 (dt, *J* = 11.6, 7.3 Hz, 1H), 1.52–1.30 (m, 4H), 0.95–0.84 (m, 3H); ^{13}C NMR (101 MHz, $CDCl_3$) δ 157.0, 120.0, 31.2, 30.9, 27.5, 25.2, 23.6, 22.3, 21.2, 17.2, 13.9, 13.9 (^{13}C NMR spectrum presented as a 1:1 mixture of tautomers); LCMS (ESI) m/z: $[M + H]^+$ Calcd for $C_6H_{13}N_4$ 141.2; Found 141.3. This compound was known in the literature and spectral data are agreed with that of reported values.[5]

5-(2*H*-Tetrazol-5-yl)benzo[*c*][1,2,5]oxadiazole (S5)

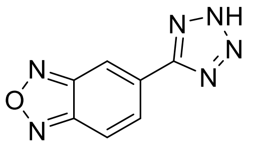

The reaction was performed as described in general procedure A using benzo[c][1,2,5]oxadiazole-5-carbonitrile (200 mg, 1.38 mmol) to afford 5-(2*H*-tetrazol-5-yl)benzo[*c*][1,2,5]oxadiazole (**S5**) (198 mg, 76%) as a brown solid. IR vmax (solid) 2956, 1544, 1395, 1020, 993, 888, 748 cm^{-1}; ^{1}H NMR (500 MHz, D_6-DMSO) δ 8.70 (s, 1H), 8.32 (d, *J* = 9.3 Hz, 1H), 8.22 (d, *J* = 9.3 Hz, 1H); ^{13}C NMR (126 MHz, D_6-DMSO) δ 155.7, 148.8, 148.8, 130.8, 128.6, 117.9, 114.9; HRMS (ESI) m/z: $[M + H]^+$ Calcd for $C_7H_5N_6O$ 189.0519; Found 189.0515.

General Procedure B: Preparation of 2,4-Dinitrophenyl Tetrazole Intermediates

To a stirred solution of the appropriate tetrazole (1.0 equiv.) in acetone (8 mL/mmol) was added triethylamine (1.0 equiv.) and 1-fluoro-2,4-dinitrobenzene (0.9–1.0 equiv.). The reaction mixture was stirred at room temperature for 1–20 hours. The resulting precipitate was obtained by filtration, dried *in vacuo* and used without further purification unless otherwise stated. In the instances where the product would fail to precipitate, the mixture was concentrated *in vacuo* and the resulting residue was purified by flash column chromatography and/or recrystallisation to afford the disubstituted tetrazole product.

2-(2,4-Dinitrophenyl)-5-phenyl-2*H*-tetrazole (1a)

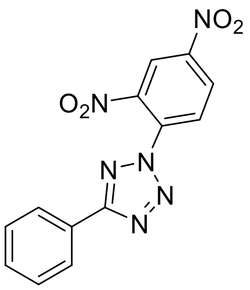

The reaction was performed as described in general procedure B using 5-phenyl-1*H*-tetrazole (2.00 g, 13.7 mmol), triethylamine (1.90 mL, 13.6 mmol) and 1-fluoro-2,4-dinitrobenzene (1.70

mL, 13.5 mmol). The resulting solution was stirred for 18 hours. Recrystallisation from EtOH afforded 2-(2,4-dinitrophenyl)-5-phenyl-2*H*-tetrazole (**1a**) (4.20 g, quant.) as a pale yellow solid. R*f* = 0.15 (pet. ether/EtOAc 9:1); IR vmax (solid) 3088, 3007, 1822,1546, 1431, 1358, 1325, 1293, 1219, 1017, 989, 913, 845, 837, 737 cm^{-1}; ^{1}H NMR (500 MHz, D_6-DMSO) δ 9.10 (d, *J* = 2.4 Hz, 1H), 8.82 (dd, *J* = 8.9, 2.4 Hz, 1H), 8.51 (d, *J* = 8.9 Hz, 1H), 8.19–8.09 (m, 2H), 7.68–7.60 (m, 3H); ^{13}C NMR (126 MHz, D_6-DMSO) δ 165.3, 148.3, 142.7, 131.6, 131.5, 129.6 (2 × CH), 129.0, 127.9, 126.8 (2 × CH), 125.5, 121.5; HRMS (ESI) m/z: $[M + H]^+$ Calcd for $C_{13}H_9N_6O_4$ 313.0679; Found 313.0685.

2-(2,4-Dinitrophenyl)-5-(4-methoxyphenyl)-2*H*-tetrazole (**1b**)

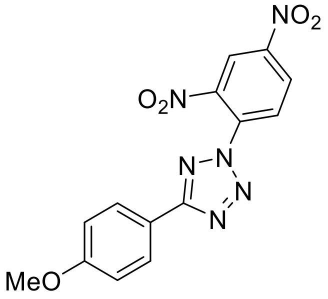

The reaction was performed as described in general procedure B using 5-(4-methoxyphenyl)-2*H*-tetrazole (**S2**) (80.0 mg, 454 µmol), triethylamine (64.0 µL, 459 µmol) and 2,4-dinitrofluorobenzene (57.0 µL, 454 µmol). The resulting solution was stirred for 18 hours. Recrystallisation from EtOH afforded 2-(2,4-dinitrophenyl)-5-(4-methoxyphenyl)-2*H*-tetrazole (**1b**) (155 mg, quant.) as a yellow solid. *Rf* = 0.62 (pet. ether/EtOAc 3:2); IR (solid) 3092, 1612, 1547, 1533, 1470, 1348, 1252, 1177, 1111, 1018, 1005, 980, 833, 737 cm^{-1}; ^{1}H NMR (400 MHz, $CDCl_3$) δ 8.80 (d, *J* = 2.4 Hz, 1H), 8.65 (dd, *J* = 8.9, 2.4 Hz, 1H), 8.34 (d, *J* = 8.9 Hz, 1H), 8.14 (d, *J* = 9.0 Hz, 2H), 7.04 (d, *J* = 9.0 Hz, 2H), 3.90 (s, 3H); ^{13}C NMR (101 MHz, $CDCl_3$) δ 166.6, 162.4, 147.8, 133.7, 132.9, 129.2 (2 × CH), 127.8, 126.5, 121.2, 118.4, 114.7 (2 × CH), 55.6; HRMS (ESI) m/z: $[M]^+$ Calcd for $C_{14}H_{10}N_6O_5$ 342.0707; Found 342.0718.

2-(2,4-Dinitrophenyl)-5-(3,4,5-trimethoxyphenyl)-2*H*-tetrazole (1c)

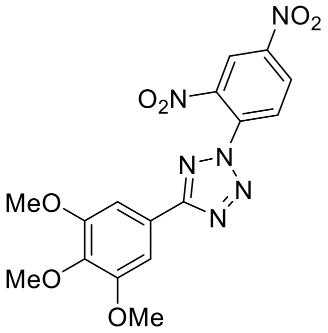

The reaction was performed as described in general procedure B using 5-(3,4,5-trimethoxyphenyl)-2*H*-tetrazole (**S3**) (200 mg, 847 µmol), triethylamine (118 µL, 847 mmol) and 2,4-dinitrofluorobenzene (86.0 µL, 685 µmol). The resulting solution was stirred for 18 hours. Recrystallisation from EtOH afforded 2-(2,4-dinitrophenyl)-5-(3,4,5-trimethoxyphenyl)-2*H*-tetrazole (**1c**) (201 mg, 73%) as an orange solid. R*f* = 0.28 (pet. ether/EtOAc 3:2); IR (solid) 3109, 2964, 1548, 1486, 1470, 1429, 1349, 1245, 1221, 1131, 987, 863, 764, 740 cm^{-1}; ^{1}H NMR (400 MHz, $CDCl_3$) δ 8.85 (d, *J* = 2.2 Hz, 1H), 8.68 (dd, *J* = 8.9, 2.2 Hz, 1H), 8.33 (d, *J* = 8.9 Hz, 1H), 7.45 (s, 2H), 3.98 (s, 6H, 2 × CH_3), 3.94 (s, 3H); ^{13}C NMR (126 MHz, $CDCl_3$) δ 166.6, 154.0, 148.0, 143.1, 141.0, 132.9, 130.3, 128.0, 126.9, 121.3, 121.0, 104.7 (2 × CH), 61.2, 56.5 (2 × CH_3); HRMS (ESI) m/z: $[M - H]^+$ Calcd for $C_{16}H_{14}N_6O_7$ 401.0918; Found 401.0911.

5-(Benzo[*d*][1,3]dioxol-5-yl)-2-(2,4-dinitrophenyl)-2*H*-tetrazole (1d)

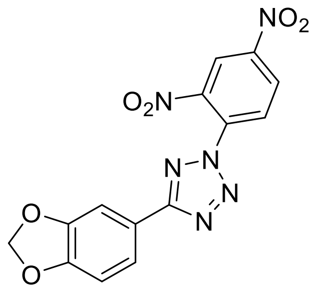

The reaction was performed as described in general procedure B using 5-(benzo[*d*][1,3]dioxol-5-yl)-2*H*-tetrazole (**S4**) (200 mg, 1.05 mmol), triethylamine (146 µL, 1.05 mmol) and 2,4-dinitrofluorobenzene (107 µL, 852 µmol). The resulting solution was stirred for 18 hours. Recrystallisation from EtOH afforded 5-(benzo[*d*][1,3]dioxol-5-yl)-2-(2,4-dinitrophenyl)-2*H*-tetrazole (**1d**) (171 mg, 56%) as a yellow solid. R*f* = 0.64 (pet. ether/EtOAc 3:2); IR (solid) 1603, 1551,1526, 1470, 1422, 1337, 1244, 1209, 1036, 1015, 982, 831, 739 cm^{-1}; ^{1}H NMR (400 MHz, $CDCl_3$) δ 8.81 (d, *J* = 2.1 Hz, 1H), 8.66 (dd, *J* = 8.8, 2.1 Hz, 1H), 8.33 (d, *J* = 8.8 Hz, 1H), 7.78 (d, *J* = 8.2 Hz, 1H), 7.62 (s, 1H), 6.96 (d, *J* = 8.2 Hz, 1H); ^{13}C NMR (126 MHz,

$CDCl_3$) δ 166.5, 164.0, 159.1, 150.6, 148.6, 132.9, 127.9, 126.5, 122.5, 121.2, 119.7, 109.2, 107.6, 101.9; HRMS (ESI) m/z: $[M + H]^+$ Calcd for $C_{14}H_9N_6O_6$ 357.0578; Found 357.0571.

2-(2,4-Dinitrophenyl)-5-(ethylthio)-2*H*-tetrazole (1e)

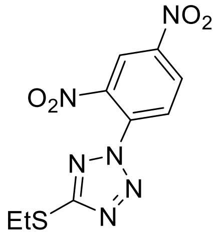

The reaction was performed as described in general procedure B using 5-(ethylthio)-1*H*-tetrazole (200 mg, 1.54 mmol), triethylamine (213 µL, 1.53 mmol) and 1-fluoro-2,4-dinitrobenzene (156 µL, 1.24 mmol). The resulting solution was stirred for 18 hours. Purification by flash column chromatography using a gradient system (pet. ether/EtOAc 20:1 to 10:1) afforded 2-(2,4-dinitrophenyl)-5-(ethylthio)-2*H*-tetrazole (**1e**) (306 mg, 83%) as a yellow solid. R*f* = 0.38 (pet. ether/EtOAc 4:1); IR vmax (solid) 3115, 3092, 2988, 2941, 2876, 1696, 1608, 1556, 1538, 1433, 1411, 1340, 1325, 1190, 1067, 1031, 975, 838, 741, 713 cm^{-1}; ^1H NMR (400 MHz, $CDCl_3$) δ 8.78 (d, *J* = 2.4 Hz, 1H), 8.64 (dd, *J* = 8.9, 2.4 Hz, 1H), 8.26 (d, *J* = 8.9 Hz, 1H), 3.26 (q, *J* = 7.4 Hz, 2H), 1.47 (t, *J* = 7.4 Hz, 3H); ^{13}C NMR (101 MHz, $CDCl_3$) δ 167.7, 147.8, 142.9, 132.5, 127.9, 126.4, 121.2, 26.7, 14.8; HRMS (ESI) m/z: $[M – H]^–$ Calcd for $C_9H_7N_6O_4S$ 295.9739 Found 295.9735

5-(5-Bromothiophen-2-yl)-2-(2,4-dinitrophenyl)-2*H*-tetrazole (1f)

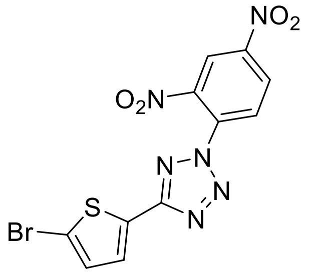

The reaction was performed as described in general procedure B using 5-(5-bromothiophen-2-yl)-1*H*-tetrazole (200 mg, 866 µmol), triethylamine (120 µL, 861 µmol) and 1-fluoro-2,4-dinitrobenzene (98.7 µL, 786 µmol). The resulting solution was stirred for 18 hours. Purification by flash column chromatography using a gradient system (pet. ether/EtOAc 19:1 to 4:1) afforded 5-(5-bromothiophen-2-yl)-2-(2,4-dinitrophenyl)-2*H*-tetrazole (**1f**) (309 mg, 99%) as a

yellow solid. R*f* = 0.47 (pet. ether/EtOAc 4:1); IR vmax (solid) 3115, 3090, 2925, 2856, 1605, 1577, 1541, 1498, 1480, 1353, 1338, 1227, 1208, 1178, 1107, 998, 908, 834, 821, 741 cm^{-1}; ^{1}H NMR (400 MHz, $CDCl_3$) δ 8.84 (d, *J* = 2.4 Hz, 1H), 8.67 (dd, *J* = 8.8, 2.4 Hz, 1H), 8.29 (d, *J* = 8.8 Hz, 1H), 7.68 (d, *J* = 3.9 Hz, 1H), 7.17 (d, *J* = 3.9 Hz, 1H); ^{13}C NMR (101 MHz, $CDCl_3$) δ 161.8, 148.1, 132.7, 131.4, 130.0, 128.7, 128.0, 126.9, 121.4, 117.6; HRMS (ESI) m/z: $[M – H]^{–}$ Calcd for $C_{11}H_4BrN_6O_4S$ 396.9360; Found 396.9365.

5-(4-Chlorophenyl)-2-(2,4-dinitrophenyl)-2*H*-tetrazole (1g)

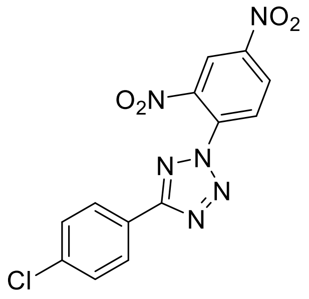

The reaction was performed as described in general procedure B using 5-(4-chlorophenyl)-1*H*-tetrazole (200 mg, 1.11 mmol), triethylamine (154 µL, 1.10 mmol) and 1-fluoro-2,4-dinitrobenzene (125 µL, 995 µmol). The resulting solution was stirred for 18 hours. Recrystallisation from EtOH afforded 5-(4-chlorophenyl)-2-(2,4-dinitrophenyl)-2*H*-tetrazole (**1g**) (331 mg, 96%) as a pale-yellow solid. R*f* = 0.53 (pet. ether/EtOAc 4:1); IR vmax (solid) 3122, 3090, 2917, 1608, 1554, 1536, 1500, 1465, 1350, 1221, 1139, 1097, 1013, 986, 845, 836, 757, 741, 720 cm^{-1}; ^{1}H NMR (400 MHz, D_6-DMSO) δ 9.10 (d, *J* = 2.5 Hz, 1H), 8.83 (dd, *J* = 8.9, 2.5 Hz, 1H), 8.50 (d, *J* = 8.9 Hz, 1H), 8.16 (d, *J* = 8.6 Hz, 2H), 7.72 (d, *J* = 8.6 Hz, 2H); ^{13}C NMR (101 MHz, D_6-DMSO) δ 164.4, 148.4, 142.7, 136.3, 131.4, 129.8 (2 × CH), 129.1, 128.6 (2 × CH), 127.9, 124.3, 121.5; HRMS (ESI) m/z: $[M + H]^{+}$ Calcd for $C_{13}H_8ClN_6O_4$ 347.0290; Found 347.0286.

2-(2,4-Dinitrophenyl)-5-(4-nitrophenyl)-2*H*-tetrazole (1h)

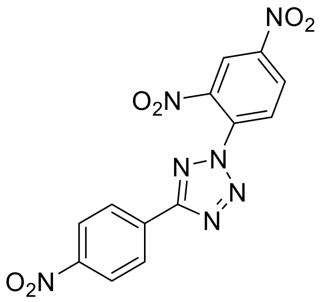

The reaction was performed as described in general procedure B using 5-(4-nitrophenyl)-1*H*-tetrazole (200 mg, 1.05 mmol), triethylamine (146 µL, 1.05 mmol) and 1-fluoro-2,4-dinitrobenzene (120 µL, 956 µmol). The resulting solution was stirred for 18 hours. Recrystallisation from EtOH afforded 2-(2,4-dinitrophenyl)-5-(4-nitrophenyl)-2*H*-tetrazole (**1h**) (321 mg, 94%) as a light brown solid. R*f* = 0.19 (pet. ether/EtOAc 4:1); IR vmax (solid) 3088, 1605, 1541, 1525, 1498, 1350, 1316, 1221, 1094, 1014, 986, 854, 735 cm^{-1}; ^{1}H NMR (400 MHz, $CDCl_3$) δ 8.89 (d, *J* = 2.4 Hz, 1H), 8.72 (dd, *J* = 8.8, 2.4 Hz, 1H), 8.41 (s, 4H), 8.34 (d, *J* = 8.8 Hz, 1H); ^{13}C NMR (101 MHz, $CDCl_3$) δ 164.8, 149.8, 148.4, 143.3, 132.8, 131.7, 128.5 (2 × CH), 128.2, 127.1, 124.6 (2 × CH), 121.4.; HRMS (ESI) m/z: $[M – H]^-$ Calcd for $C_{13}H_6N_7O_6$ 356.0385; Found 356.0379.

5-(2,6-Dichlorophenyl)-2-(2,4-dinitrophenyl)-2*H*-tetrazole (1i)

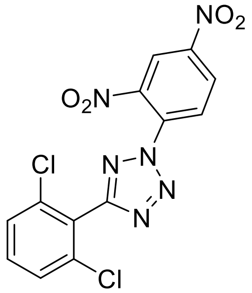

The reaction was performed as described in general procedure B using 5-(2,6-dichlorophenyl)-1*H*-tetrazole (200 mg, 930 µmol), triethylamine (130 µL, 933 µmol) and 1-fluoro-2,4-dinitrobenzene (95.0 µL, 757 µmol). The resulting solution was stirred for 16 hours to afford 5-(2,6-dichlorophenyl)-2-(2,4-dinitrophenyl)-2*H*-tetrazole (**1i**) (223 mg, 77%) as a yellow solid. R*f* = 0.15 (pet. ether/EtOAc 9:1); IR vmax (solid) 3127, 2980, 1754, 1623, 1551, 1496, 1351, 1323, 1266, 1214, 1093, 1070, 1036, 1002, 921, 853, 838, 791, 745, 700 cm^{-1}; ^{1}H-NMR (400 MHz, $CDCl_3$) δ 8.87(d, *J* = 2.4 Hz, 1H), 8.70 (dd, *J* = 8.8, 2.4 Hz, 1H), 8.35 (d, *J* = 8.8 Hz, 1H), 7.56–7.40 (m, 3H); ^{13}C NMR (101 MHz, $CDCl_3$) δ 162.4, 148.4, 143.5, 136.5 (2

× C), 133.0, 132.6, 128.5, (2 × CH), 128.1, 127.5, 125.8, 121.4; HRMS (ESI) m/z: $[M - H]^-$ Calcd for $C_{13}H_5Cl_2N_6O_4$ 380.9900; Found 380.9909.

5-(4-Bromophenyl)-2-(2,4-dinitrophenyl)-2*H*-tetrazole (1j)

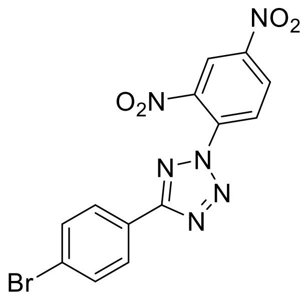

The reaction was performed as described in general procedure B using 5-(4-bromophenyl)-1*H*-tetrazole (250 mg, 1.11 mmol), triethylamine (160 µL, 1.15 mmol) and 1-fluoro-2,4-dinitrobenzene (140 µL, 1.11 mmol). The resulting solution was stirred for 1 hour to afford 5-(4-bromophenyl)-2-(2,4-dinitrophenyl)-2*H*-tetrazole (**1j**) (325 mg, 75%) as a yellow solid. R*f* = 0.13 (pet. ether/EtOAc 9:1); IR vmax (solid) 3090, 1605, 1541, 1498, 1459, 1353, 1217, 1186, 1093, 1072, 1011, 985, 921, 849, 836, 741, 679, 661 cm^{-1}; ^1H NMR (500 MHz, $CDCl_3$) δ 8.84 (d, *J* = 2.3 Hz, 1H), 8.68 (dd, *J* = 8.8, 2.3 Hz, 1H), 8.33 (d, *J* = 8.8 Hz, 1H), 8.09 (d, *J* = 8.5 Hz, 2H), 7.69 (d, *J* = 8.5 Hz, 2H); ^{13}C NMR (126 MHz, $CDCl_3$) δ 166.0, 148.1, 143.1, 132.9, 132.7 (2 × CH), 129.0 (2 × CH), 128.0, 126.8, 126.3, 124.9, 121.3; HRMS (EI) m/z: $[M + H]^+$ Calcd for $C_{13}H_8BrN_6O_4$ 390.9785; Found 390.9790.

5-(3-Bromophenyl)-2-(2,4-dinitrophenyl)-2*H*-tetrazole (1k)

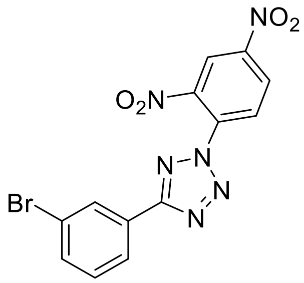

The reaction was performed as described in general procedure B using 5-(3-bromophenyl)-1*H*-tetrazole (100 mg, 444 µmol), triethylamine (65.0 µL, 466 µmol) and 1-fluoro-2,4-dinitrobenzene (56.0 µL, 446 µmol). The resulting solution was stirred for 1 hour. Recrystallisation from EtOH afforded 5-(3-bromophenyl)-2-(2,4-dinitrophenyl)-2*H*-tetrazole (**1k**) (91.0 mg, 52%) as a pale yellow solid. R*f* = 0.15 (pet. ether/EtOAc 9:1); IR vmax (film)

3022, 2173, 1369, 1231, 950, 860, 520 cm^{-1}; ^{1}H NMR (500 MHz, $CDCl_3$) δ 8.85 (d, *J* = 2.4 Hz, 1H), 8.69 (dd, *J* = 8.9, 2.4 Hz, 1H), 8.37 (br s, 1H), 8.33 (d, *J* = 8.9 Hz, 1H), 8.15 (d, *J* = 7.9 Hz, 1H), 7.69 (d, *J* = 7.9 Hz, 1H), 7.45 (t, *J* = 7.9 Hz, 1H); ^{13}C NMR (126 MHz, $CDCl_3$) δ 165.5, 148.1, 143.2, 134.6, 132.9, 130.9, 130.5, 128.0, 127.8, 126.9, 126.1, 123.4, 121.4; HRMS (EI) m/z: $[M + H]^+$ Calcd for $C_{13}H_8BrN_6O_4$ 390.9784; Found 390.9786.

2-(2,4-Dinitrophenyl)-5-methyl-2*H*-tetrazole (1l)

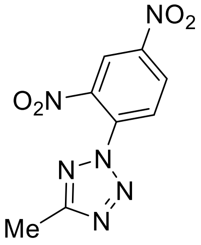

The reaction was performed as described in general procedure B using 5-methyl-1*H*-tetrazole (1.00 g, 11.9 mmol), triethylamine (1.65 mL, 11.8 mmol) and 1-fluoro-2,4-dinitrobenzene (1.35 mL, 10.8 mmol). The resulting solution was stirred for 18 hours. Purification by flash column chromatography using a gradient system (pet. ether/EtOAc 19:1 to 4:1) afforded 2-(2,4-dinitrophenyl)-5-methyl-2*H*-tetrazole (**1l**) (890 mg, 33%) as a light yellow solid. R*f* = 0.26 (pet. ether/EtOAc 4:1); IR vmax (solid) 3118, 3100, 1612, 1551, 1539, 1521, 1350, 1262, 1215, 1098, 1011, 838, 743, 720 cm^{-1}; ^{1}H NMR (400 MHz, $CDCl_3$) δ 8.78 (d, *J* = 2.4 Hz, 1H), 8.64 (dd, *J* = 8.8, 2.4 Hz, 1H), 8.23 (d, *J* = 8.8 Hz, 1H), 2.66 (s, 3H); ^{13}C NMR (101 MHz, $CDCl_3$) δ 164.9, 147.8, 143.0, 132.9, 127.9, 126.7, 121.2, 11.1; HRMS (ESI) m/z: $[M]^+$ Calcd for $C_8H_6N_6O_4$ 250.0456; Found 250.0457.

5-Benzyl-2-(2,4-dinitrophenyl)-2*H*-tetrazole (1m)

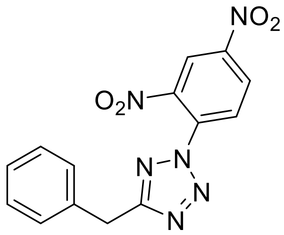

The reaction was performed as described in general procedure B using 5-benzyl-1*H*-tetrazole (200 mg, 1.25 mmol), triethylamine (173 µL, 1.24 mmol) and 1-fluoro-2,4-dinitrobenzene (127 µL, 1.01 mmol). The resulting solution was stirred for 18 hours. Purification by flash column chromatography using a gradient system (pet. ether/EtOAc 20:1 to 10:1) afforded 5-benzyl-2-

(2,4-dinitrophenyl)-2*H*-tetrazole (**1m**) (203 mg, 62%) as a yellow solid. R*f* = 0.28 (pet. ether/EtOAc 4:1); IR vmax (solid) 3304, 3090, 2928, 1713, 1608, 1541, 1512, 1498, 1458, 1428, 1342, 1227, 1098, 990, 835, 743, 703 cm^{-1}; ^{1}H NMR (400 MHz, $CDCl_3$) δ 8.80 (d, *J* = 2.3 Hz, 1H), 8.62 (dd, *J* = 8.9, 2.3 Hz, 1H), 8.22 (d, *J* = 8.9 Hz, 1H), 7.39–7.31 (m, 4H), 7.32–7.27 (m, 1H), 4.37 (s, 2H); ^{13}C NMR (101 MHz, $CDCl_3$) δ 167.5, 147.9, 143.1, 135.6, 133.0, 129.0 (4 × CH), 127.9, 127.4, 126.9, 121.2, 31.9; HRMS (ESI) m/z: $[M + H]^+$ Calcd for $C_{14}H_{11}N_6O_4$ 327.0836; Found 327.0832.

2-(2,4-Dinitrophenyl)-5-pentyl-2*H*-tetrazole (1n)

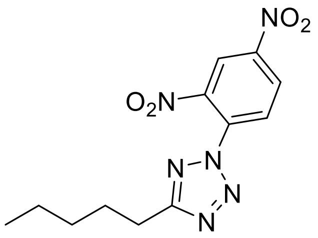

The reaction was performed as described in general procedure B using 5-pentyltetrazole (**S5**) (117 mg, 835 µmol), triethylamine (117 µL, 839 µmol) and 2,4-dinitrofluorobenzene (85.0 µL, 677 µmol). The resulting solution was stirred for 18 hours. Purification by flash column chromatography using a gradient system (pet. ether/EtOAc 100:0 to 95:5) afforded a brown oil. Further purification by flash column chromatography (pet. ether then CH_2Cl_2) afforded 2-(2,4-dinitrophenyl)-5-pentyl-2*H*-tetrazole (**1n**) (58.0 mg, 28%) as an orange oil. R*f* = 0.51 (pet. ether/EtOAc 3:2); IR (solid) 3109, 2943, 1620, 1548, 1520, 1349, 992, 921, 838, 745 cm^{-1}; ^{1}H NMR (500 MHz, $CDCl_3$) δ 8.78 (d, *J* = 2.3 Hz, 1H), 8.63 (dd, *J* = 8.9, 2.3 Hz, 1H), 8.25 (d, *J* = 8.9 Hz, 1H), 2.99 (t, *J* = 7.6 Hz, 2H), 1.84 (quin., *J* = 7.6 Hz, 2H), 1.42–1.34 (m, 4H), 0.91 (t, *J* = 6.8 Hz, 3H); ^{13}C NMR (126 MHz, $CDCl_3$) δ 168.9, 147.8, 143.1, 133.0, 127.9, 126.7, 121.2, 31.2, 27.5, 25.5, 22.4, 14.0; HRMS (ESI) m/z: $[M + H]^+$ Calcd for $C_{12}H_{15}N_6O_4$ 307.1149; Found 307.1144.

5-(3,4-Dimethoxyphenyl)-2-(2,4-dinitrophenyl)-2*H*-tetrazole (1o)

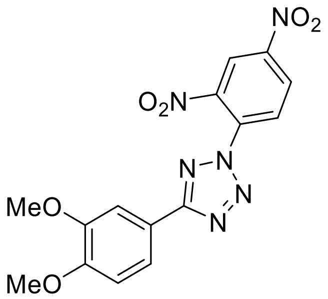

3,4-Dimethoxybenzonitrile (400 mg, 2.45 mmol), sodium azide (175 mg, 2.69 mmol) and ammonium chloride (144 mg, 2.69 mmol) were dissolved in DMF (3 mL) and stirred at 110 °C for 20 h. The reaction mixture was cooled to 0 °C, diluted with water (10 mL) and concentrated HCl (2 mL) was added dropwise. The resulting precipitate was washed with water and dried under high-vacuum and used in the next step without further purification. The residue was dissolved in acetone (15 mL) and triethylamine (215 µL, 1.54 mmol) and 2,4-dinitrofluorobenzene (156 µL, 1.24 mmol) were added. The resulting solution was stirred at room temperature for 16 hours and then concentrated *in vacuo*. Recrystallisation from EtOH afforded 5-(3,4-dimethoxyphenyl)-2-(2,4-dinitrophenyl)-2*H*-tetrazole (**1o**) (203 mg, 44% over two-steps) as an orange solid. R*f* = 0.26 (pet. ether/EtOAc 3:2); IR (solid) 3104, 2859, 1551, 1348, 1264, 1246, 1190, 1132, 1021, 986, 889, 831, 763, 739 cm^{-1}; ^{1}H NMR (500 MHz, $CDCl_3$) δ 8.82 (s, 1H), 8.66 (d, *J* = 8.6 Hz, 1H), 8.33 (d, *J* = 8.6 Hz, 1H), 7.82 (d, *J* = 8.2 Hz, 1H), 7.69 (s, 1H), 7.01 (d, *J* = 8.2 Hz, 1H), 4.00 (s, 3H), 3.97 (s, 3H); ^{13}C NMR (126 MHz, $CDCl_3$) δ 166.6, 152.0, 149.6, 147.8, 143.0, 132.9, 127.9, 126.6, 121.2, 121.0, 118.5, 111.5, 110.0, 56.3, 56.2; HRMS (ESI) m/z: $[M]^+$ Calcd for $C_{15}H_{12}N_6O_6$ 372.0813; Found 372.0806.

5-(2-(2,4-Dinitrophenyl)-2*H*-tetrazol-5-yl)benzo[*c*][1,2,5]oxadiazole (1p)

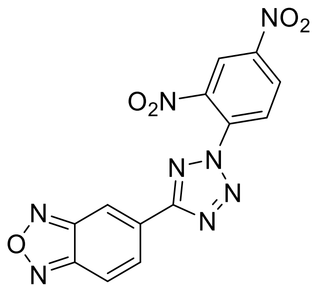

The reaction was performed as described in general procedure B using 5-(2*H*-tetrazol-5-yl)benzo[*c*][1,2,5]oxadiazole (**S6**) (180 mg, 957 µmol), triethylamine (134 µL, 961 µmol) and

2,4-dinitrofluorobenzene (97.0 µL, 772 µmol). The resulting solution was stirred for 18 hours to afford 5-(2-(2,4-dinitrophenyl)-2*H*-tetrazol-5-yl)benzo[*c*][1,2,5]oxadiazole (**1p**) (271 mg, 99%) as a beige solid. R*f* = 0.37 (pet. ether/EtOAc 3:2); IR (solid) 3074, 1544, 1347, 1007, 988, 838, 828, 756, 745 cm^{-1}; ^{1}H NMR (500 MHz, $CDCl_3$) δ 8.90 (s, 1H), 8.83 (s, 1H), 8.73 (d, *J* = 8.1 Hz, 1H), 8.36 (d, *J* = 8.1 Hz, 1H), 8.23 (d, *J* = 8.9 Hz, 1H), 8.06 (d, *J* = 8.9 Hz, 1H); ^{13}C NMR (126 MHz, $CDCl_3$) δ 164.8, 149.4, 149.2, 148.5, 132.7, 129.9, 129.1, 128.2, 127.2, 122.6, 121.5, 118.2, 116.6; HRMS (ESI) m/z: $[M]^+$ Calcd for $C_{13}H_6N_8O_5$ 354.0456; Found 354.0471.

4-(Methoxy-*d*$_3$)benzonitrile (S6)

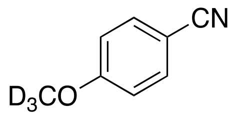

To a stirred solution of 4-cyanophenol (500 mg, 4.20 mmol) in anhydrous DMF (10 mL) was added potassium carbonate (1.16 g, 8.39 mmol). The mixture was cooled to 0 °C and iodomethane-*d*$_3$ (392 µL, 6.30 mmol) was added dropwise. The reaction mixture was warmed to room temperature and stirred for 16 h. The mixture was diluted with ethyl acetate (30 mL) and washed with water (5 × 40 mL) then brine (40 mL). The organic layer was dried over $MgSO_4$, filtered and concentrated *in vacuo* which afforded 4-(methoxy-*d*$_3$)benzonitrile (**S6**) (461 mg, 81%) as a white solid. ^{1}H NMR (400 MHz, $CDCl_3$) δ 7.60–7.55 (m, 2H), 6.96–6.92 (m, 2H); ^{13}C NMR (126 MHz, $CDCl_3$) δ 163.0, 134.1 (2 × CH), 119.3, 114.9 (2 × CH), 104.1. This compound was known in the literature and spectral data are agreed with that of reported values.[6]

2-(2,4-Dinitrophenyl)-5-(4-(methoxy-*d*$_3$)phenyl)-2*H*-tetrazole (1q)

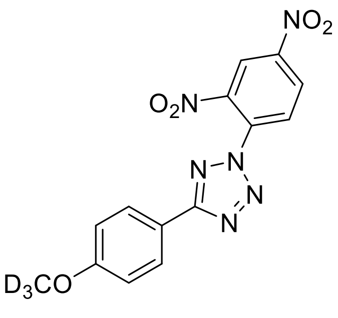

To a stirred solution of 4-(methoxy-*d*$_3$)benzonitrile (**XX**) (200 mg, 1.47 mmol) in DMF (2 mL) was added sodium azide (105 mg, 1.62 mmol) and ammonium chloride (87.0 mg, 1.63 mmol). The resulting solution was heated at 110 °C for 20 hours. The reaction mixture was cooled to

0 °C, diluted with water and concentrated HCl was added dropwise. The resulting precipitate was collected, washed with water, dried under high vacuum and used without further purification. The crude residue was dissolved in acetone (10 mL) and triethylamine (116 µL, 832 µmol) and 2,4-dinitrofluorobenzene (159 µL, 1.27 mmol) were added. The reaction mixture was stirred at room temperature for 16 hours then concentrated *in vacuo*. Trituration with ethanol afforded 2-(2,4-dinitrophenyl)-5-(4-(methoxy-d_3)phenyl)-2*H*-tetrazole (**1q**) (303 mg, 60% over two-steps) as a yellow solid. IR vmax (solid) 3237, 3092, 2926, 2222, 1680, 1603, 1595, 1531, 1499, 1468, 1366, 1263, 1180, 1103, 980, 833, 737 cm^{-1}; ^1H NMR (400 MHz, $CDCl_3$) δ 8.80 (d, *J* = 2.4 Hz, 1H), 8.65 (dd, *J* = 8.9, 2.4 Hz, 1H), 8.34 (d, *J* = 8.9 Hz, 1H), 8.14 (d, *J* = 8.6 Hz, 2H), 7.04 (d, *J* = 8.6 Hz, 2H); ^{13}C NMR (101 MHz, $CDCl_3$) δ 166.6, 162.4, 147.8, 132.9, 129.2 (2 × CH), 127.8, 126.5, 121.2, 118.3, 114.7 (2 × CH); HRMS (ESI) m/z $[M+H]^+$ Calcd for $C_{14}H_8D_3N_6O_5$ 346.0974; Found 346.0961.

2-(2,4-Dinitrophenyl-6-*d*)-5-(4-(methoxy-d_3)phenyl-2,6-d_2)-2*H*-tetrazole (1r)

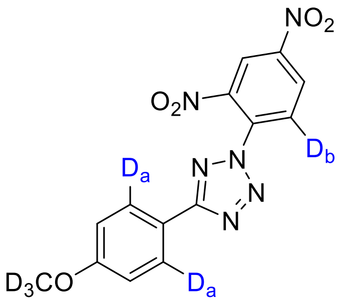

This reaction was performed as according to literature precedent.[7] A flame-dried 100 mL round-bottomed flask bearing two-stopcocks was charged with 2-(2,4-dinitrophenyl)-5-(4-(methoxy-d_3)phenyl)-2*H*-tetrazole (**S6**) (34.5 mg, 100 µmol) and [Ir(COD)(IMes)(PPh_3)][BAr^F_4] (8.50 mg, 5.00 µmol, 5 mol%) and then evacuated and backfilled with argon three times. Anhydrous CH_2Cl_2 (5 mL) was added and the mixture was cooled to –78 °C. The flask was evacuated and refilled with deuterium gas from a balloon, and this vacuum/refill cycle was repeated a further two times. The stopcocks were closed, and the reaction mixture was warmed to room temperature and stirred for 24 h. The reaction mixture was diluted with dichloromethane, transferred to 100 mL round bottomed flask and concentrated *in vacuo*. Purification by flash column chromatography using a gradient system (pet. ether/EtOAc 90:10 to 80:20) afforded 2-(2,4-dinitrophenyl-6-*d*)-5-(4-(methoxy-d_3)phenyl-2,6-d_2)-2*H*-tetrazole (**1r**) (30.2 mg, 87%) as a yellow solid. The product was then analysed directly *via* ^1H NMR spectroscopy and the integrals were calibrated against a signal corresponding to a position

where labelling was not expected to occur. The extent of deuterium incorporation was then calculated according to the following equation:

$$\%D = 100 - \left[100 \times \left(\frac{\textit{residual integral}}{\textit{expected integral}}\right)\right]$$

^{1}H NMR (400 MHz, $CDCl_3$) δ 8.81 (d, J = 2.4 Hz, 1H), 8.66 (d, J = 2.4 Hz, 1H), 7.04 (s, 2H); ^{13}C NMR (101 MHz, $CDCl_3$) δ 162.4, 147.8, 143.0, 132.9, 129.2, 128.9, 128.7, 127.8, 121.2, 118.2, 114.9, 114.6 (2 × CH), OCD_3 quaternary carbon signal not observed .Deuterium Incorporation: D_a = 96%, D_b = 91% (Labelling expected at signals 8.34 ppm and 8.14 ppm, measure against the signal at 8.81 ppm).

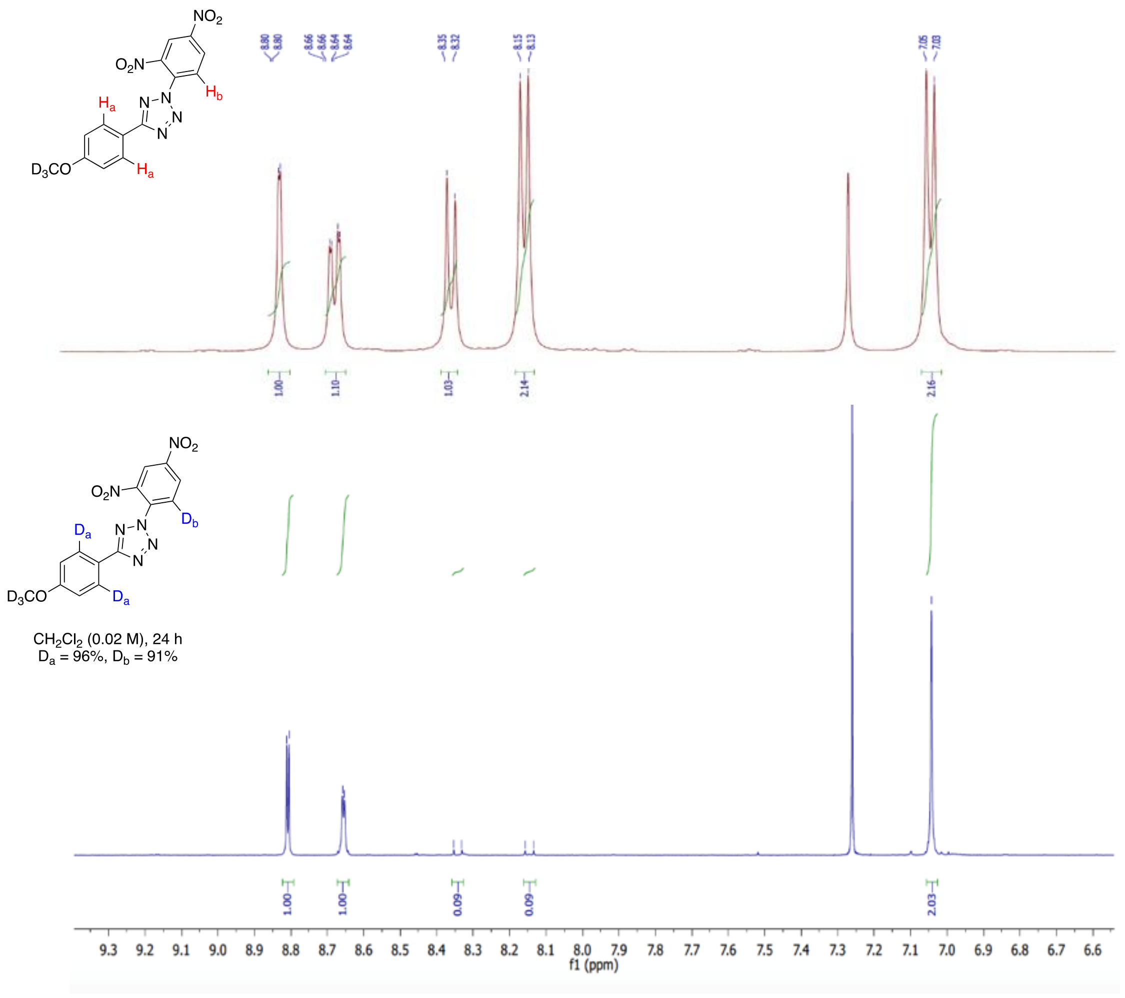

1*H*-Benzo[*d*][1,2,3]triazol-1-yl benzoate (2a)

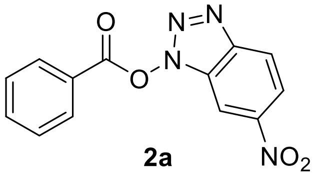

2a

A solution of 2-(2,4-dinitrophenyl)-5-phenyl-2*H*-tetrazole (**1a**) (100 mg, 320 µmol, 1.0 equiv.) in toluene (2 mL) was heated at 110 °C for 2 hours to afford 1*H*-benzo[*d*][1,2,3]triazol-1-yl benzoate (**2a**) (91mg, quant.) as a yellow solid. ^{1}H NMR (500 MHz, D_6-DMSO) δ 8.62 (d, *J* = 1.9 Hz, 1H), 8.24 (d, *J* = 9.1 Hz, 1H), 8.19 (dd, *J* = 9.1, 1.9 Hz, 1H), 7.96–7.91 (m, 2H), 7.64–7.59 (m, 1H), 7.49 (t, *J* = 7.7 Hz, 2H); ^{13}C NMR (126 MHz, D_6-DMSO) δ 167.3, 146.3, 144.7, 132.8, 130.8, 129.2 (2 × CH), 128.5 (2 × CH), 127.0, 120.7, 119.2, 107.4; HRMS (ESI) m/z: $[M + H]^+$ Calcd for $C_{13}H_9N_4O_4$ 285.0618; Found 285.0622.

***N*-Benzyl-2,4-dinitroaniline (2b)**

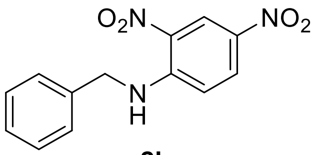

2b

To a solution of 2-(2,4-dinitrophenyl)-5-phenyl-2*H*-tetrazole (**1a**) (100 mg, 320 µmol, 1.0 equiv.) in toluene was added benzylamine (35.0 µL, 320 µmol, 1.0 equiv.) and heated at 110 °C for 5 hours to afford *N*-benzyl-2,4-dinitroaniline as a yellow solid (**2b**) (63%*). ^{1}H NMR (400 MHz, $CDCl_3$) δ 9.16 (d, *J* = 2.6 Hz, 1H), 8.91 (br s, 1H), 8.23 (dd, *J* = 9.6, 2.6 Hz, 1H), 7.46–7.30 (m, 5H), 6.92 (d, *J* = 9.6 Hz, 1H), 4.65 (d, *J* = 5.6 Hz, 2H); ^{13}C NMR (101 MHz, $CDCl_3$) 148.3, 141.1, 135.7, 130.5, 129.4, 128.9, 128.8, 128.5, 127.6, 127.4, 127.2, 126.9, 124.4, 114.5, 47.7, 46.0 (^{13}C NMR spectrum presented as a mixture of rotamers); LCMS (EI) m/z: $[M - H]^-$ Calcd for $C_{13}H_{10}N_3O_4$ 272.3; Found 272.1 at 8.76 mins. This compound was known in the literature and spectral data agreed with that of the reported values.[8]

*Yield determined by qNMR spiked with 1,3,5-trimethoxybenzene as an internal standard. A small sample of the mixture was further purified for characterisation purposes.

This demonstrated that the activation period was necessary to form the active ester before addition of amine.

General Procedure C: Preparation of Amide Products

The following general procedure example is used for reaction representation purposes.

A solution of disubstituted tetrazole (1.0 equiv.) in toluene (5 mL/mmol) was heated at 110 °C for 2 h. The reaction was allowed to cool to 40 °C and the appropriate amine (1.0 equiv.) was then added. The mixture was stirred at 40 °C to room temperature for 0.1–72 hours. The resulting hydroxybenzotriazole precipitate was removed by filtration and the filtrate was diluted with EtOAc and washed with 2 M aqueous HCl. The organic extract was dried over $MgSO_4$, filtered and concentrated *in vacuo*. In some instances, the residue required purification by flash column chromatography and/or recrystallisation to afford the desired product. In the instances where the hydroxybenzotriazole would fail to precipitate, the mixture was concentrated *in vacuo* and the resulting residue was diluted with EtOAc (20 mL) and washed with 2 M aqueous HCl (2 × 20 mL) and 2 M aqueous NaOH (2 × 20 mL). The organic extract was dried over $MgSO_4$, filtered and concentrated *in vacuo*. The residue obtained was purified by flash column chromatography and/or recrystallisation to afford the desired product.

***N*-Benzylbenzamide (3a)**

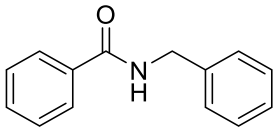

The reaction was performed as described in general procedure C using 2-(2,4-dinitrophenyl)-5-phenyl-2*H*-tetrazole (**1a**) (100 mg, 320 µmol) and benzylamine (35.0 µL, 320 µmol). The resulting solution was stirred for 10 minutes to afford *N*-benzylbenzamide (**3a**) (51.5 mg, 76%) as a yellow solid. R*f* = 0.27 (pet. ether/EtOAc 4:1); ^{1}H NMR (500 MHz, D_6-DMSO) δ 9.03 (t, *J* = 6.1 Hz, 1H), 7.92–7.87 (m, 2H), 7.57–7.50 (m, 1H), 7.50–7.44 (m, 2H), 7.35–7.30 (m, 4H), 7.27–7.21 (m, 1H), 4.48 (d, *J* = 6.1 Hz, 2H); ^{13}C NMR (126 MHz, D_6-DMSO) δ 166.2, 139.7, 134.3, 131.2, 128.3 (2 × CH), 128.2 (2 × CH), 127.2 (2 × CH), 127.2 (2 × CH), 126.7, 42.6; LCMS (EI) m/z: $[M + H]^+$ Calcd for $C_{14}H_{14}NO$ 212.2; Found 212.1 at 7.37 mins. This compound was known in the literature and spectral data are agreed with that of reported values.[9]

Large scale synthesis of *N*-Benzylbenzamide (3a)

The reaction was performed as described in general procedure C using 2-(2,4-dinitrophenyl)-5-phenyl-2*H*-tetrazole (**1a**) (1.04 g, 3.33 mmol) and benzylamine (364 µL, 3.33 mmol). The resulting solution was stirred for 3 hours to afford *N*-benzylbenzamide (**3a**) (564 mg, 80%) as an off-white solid. The characterisation data was as reported above.

***N*-(1-Phenylethyl)benzamide (3b)**

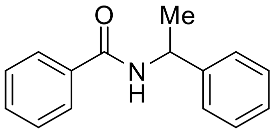

The reaction was performed as described in general procedure C using 2-(2,4-dinitrophenyl)-5-phenyl-2*H*-tetrazole (**1a**) (100 mg, 320 µmol) and (±)-methylbenzylamine (42.0 µL, 326 µmol). The resulting solution was stirred for 10 minutes to afford *N*-(1-phenylethyl)benzamide (**3b**) (64.0 mg, 89%) as a yellow solid. R*f* = 0.30 (pet. ether/EtOAc 4:1); ^{1}H NMR (500 MHz, D_6-DMSO) δ 8.78 (d, *J* = 8.2 Hz, 1H), 7.92–7.86 (m, 2H), 7.55–7.50 (m, 1H), 7.49–7.44 (m, 2H), 7.41–7.37 (m, 2H), 7.35–7.29 (m, 2H), 7.22 (tt, *J* = 7.3, 1.3 Hz, 1H), 5.17 (quin., *J* = 7.1 Hz, 1H), 1.48 (d, *J* = 7.1 Hz, 3H); ^{13}C NMR (126 MHz, D_6-DMSO) δ 165.5, 144.9, 135.5, 131.1, 128.2 (2 × CH), 128.2 (2 × CH), 127.3 (2 × CH), 126.6, 126.0 (2 × CH), 48.4, 22.2; LCMS (EI) m/z: $[M + H]^+$ Calcd for $C_{15}H_{16}NO$ 226.3; Found 226.2 at 7.67 mins. This compound was known in the literature and spectral data are agreed with that of reported values.[10]

***N*-(2-Phenylpropan-2-yl)benzamide (3c)**

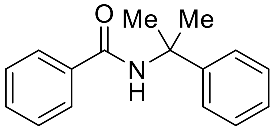

The reaction was performed as described in general procedure C using 2-(2,4-dinitrophenyl)-5-phenyl-2*H*-tetrazole (**1a**) (100 mg, 320 µmol) and 1-methyl-1-phenylethylamine (43.3 mg, 320 µmol). The resulting solution was stirred for 45 minutes to afford *N*-(2-phenylpropan-2-yl)benzamide (**3c**) (75.4 mg, 98%) as a yellow solid. R*f* = 0.38 (pet. ether/EtOAc 4:1); ^{1}H NMR (400 MHz, $CDCl_3$) δ 7.79–7.74 (m, 2H), 7.52–7.40 (m, 5H), 7.39–7.32 (m, 2H), 7.28–7.22 (m, 1H), 6.40 (br s, 1H), 1.84 (s, 6H); ^{13}C NMR (101 MHz, $CDCl_3$) δ 166.6, 147.0, 135.6, 131.5, 128.7 (2 × CH), 128.7 (2 × CH), 127.0 (2 × CH), 126.9, 124.9 (2 × CH), 56.5, 29.3 (2 × CH_3); LCMS (EI) m/z: $[M + H]^+$ Calcd for $C_{16}H_{18}NO$ 240.3; Found 240.2 at 8.04 mins. This compound was known in the literature and spectral data are agreed with that of reported values.[11]

N-(2-(Thiophen-2-yl)ethyl)benzamide (3d)

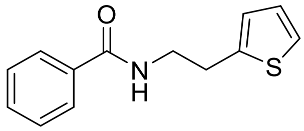

The reaction was performed as described in general procedure C using 2-(2,4-dinitrophenyl)-5-phenyl-2*H*-tetrazole (**1a**) (100 mg, 320 µmol) and 2-thiopheneethylamine (38.0 µL, 325 µmol). The resulting solution was stirred for 16 hours to afford *N*-(2-(thiophen-2-yl)ethyl)benzamide (**3d**) (61.7 mg, 83%) as a brown solid. R*f* = 0.19 (pet. ether/EtOAc 4:1); ^{1}H NMR (400 MHz, $CDCl_3$) δ 7.76–7.69 (m, 2H), 7.53–7.46 (m, 1H), 7.46–7.38 (m, 2H), 7.18 (dd, *J* = 5.1, 1.2 Hz, 1H), 6.97 (dd, *J* = 5.1, 3.4 Hz, 1H), 6.90–6.85 (m, 1H), 6.29 (br s, 1H), 3.74 (app q, *J* = 6.4 Hz, 2H), 3.16 (t, *J* = 6.4 Hz, 2H); ^{13}C NMR (101 MHz, $CDCl_3$) δ 167.6, 141.5, 134.7, 131.6, 128.7 (2 × CH), 127.3, 127.0 (2 × CH), 125.7, 124.2, 41.5, 30.1; LCMS (EI) m/z: $[M + H]^+$ Calcd for $C_{13}H_{14}NOS$ 232.3; Found 232.3 at 7.47 mins. This compound was known in the literature and spectral data are agreed with that of reported values.[12]

***N*-Phenethylbenzamide (3e)**

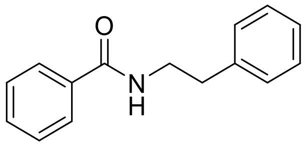

The reaction was performed as described in general procedure C using 2-(2,4-dinitrophenyl)-5-phenyl-2*H*-tetrazole (**1a**) (100 mg, 320 µmol) and phenylethylamine (41.0 µL, 325 µmol). The resulting solution was stirred for 2 hours to afford *N*-phenethylbenzamide (**3e**) (68.0 mg, 94%) as an orange oil. R*f* = 0.46 (pet. ether/EtOAc 3:2); ^{1}H NMR (500 MHz, $CDCl_3$) δ 7.63–7.59 (m, 2H), 7.41–7.36 (m, 1H), 7.33–7.28 (m, 2H), 7.26–7.21 (m, 2H), 7.18–7.12 (m, 3H), 6.21 (br s, 1H), 3.62 (q, *J* = 6.9 Hz, 2H), 2.84 (t, *J* = 6.9 Hz, 2H); ^{13}C NMR (126 MHz, $CDCl_3$) δ 167.6, 139.0, 134.8, 131.5, 128.9 (2 × CH), 128.8 (2 × CH), 128.6 (2 × CH), 126.9 (2 × CH), 126.7, 41.3, 35.8; LCMS (ESI) m/z: $[M + H]^+$ Calcd for $C_{15}H_{16}NO$ 226.1; Found 226.3 at 7.56 mins. This compound was known in the literature and spectral data are agreed with that of reported values.[13]

***N*-(But-3-yn-1-yl)benzamide (3f)**

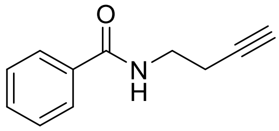

The reaction was performed as described in general procedure C using 2-(2,4-dinitrophenyl)-5-phenyl-2*H*-tetrazole (**1a**) (100 mg, 320 µmol) and 1-amino-3-butyne (26.2 µL, 320 µmol). The resulting solution was stirred for 2 hours. Purification by flash column chromatography using a gradient system (pet. ether/EtOAc 19:1 to 4:1) afforded *N*-(but-3-yn-1-yl)benzamide (**3f**) (27.6 mg, 50%) as a colourless solid. R*f* = 0.13 (pet. ether/EtOAc 4:1); ^{1}H NMR (400 MHz, $CDCl_3$) δ 7.82–7.74 (m, 2H), 7.54–7.47 (m, 1H), 7.47–7.39 (m, 2H), 6.56 (br s, 1H), 3.61 (q, *J* = 6.3 Hz, 2H), 2.52 (td, *J* = 6.3, 2.6 Hz, 2H), 2.04 (t, *J* = 2.6 Hz, 1H); ^{13}C NMR (101 MHz, $CDCl_3$) δ 167.7, 134.6, 131.7, 128.7 (2 × CH), 127.0 (2 × CH), 81.7, 70.3, 38.5, 19.6; LCMS (EI) m/z: $[M + H]^+$ Calcd for $C_{11}H_{12}NO$ 174.2; Found 174.2 at 6.32 mins. This compound was known in the literature and spectral data agreed with that of the reported values.[14]

***N*-Butylbenzamide (3g)**

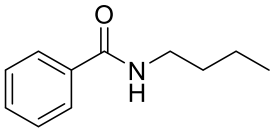

The reaction was performed as described in general procedure C using 2-(2,4-dinitrophenyl)-5-phenyl-2*H*-tetrazole (**1a**) (100 mg, 320 µmol) and butylamine (31.6 µL, 320 µmol). The resulting solution was stirred for 2 hours to afford *N*-butylbenzamide (**3g**) (56.7 mg, quant.) as a pale yellow oil. R*f* = 0.22 (pet. ether/EtOAc 4:1); ^{1}H NMR (400 MHz, $CDCl_3$) δ 7.79–7.72 (m, 2H), 7.51–7.45 (m, 1H), 7.44–7.38 (m, 2H), 6.21 (br s, 1H), 3.45 (td, *J* = 7.3, 5.7 Hz, 2H), 1.65–1.53 (m, 2H), 1.47–1.34 (m, 2H), 0.95 (t, *J* = 7.3 Hz, 3H); ^{13}C NMR (101 MHz, $CDCl_3$) δ 167.7, 135.0, 131.4, 128.7 (2 × CH), 127.0 (2 × CH), 39.9, 31.9, 20.3, 13.9; LCMS (EI) m/z: $[M + H]^+$ Calcd for $C_{11}H_{16}NO$ 178.3; Found 178.2 at 6.58 mins. This compound was known in the literature and spectral data agreed with that of the reported values.[15]

***N*-(2-Methoxyethyl)benzamide (3h)**

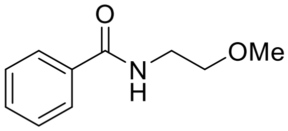

The reaction was performed as described in general procedure C using 2-(2,4-dinitrophenyl)-5-phenyl-2*H*-tetrazole (**1a**) (100 mg, 320 µmol) and 2-methoxyethylamine (27.8 µL, 320 µmol). The resulting solution was stirred for 2 hours to afford *N*-(2-methoxyethyl)benzamide (**3h**) (57.3 mg, quant.) as a pale yellow oil. R*f* = 0.64 (pet. ether/EtOAc 1:3); ^{1}H NMR (400 MHz, $CDCl_3$) δ 7.81–7.73 (m, 2H), 7.51–7.44 (m, 1H), 7.44–7.38 (m, 2H), 6.62 (br s, 1H), 3.64 (app td, *J* = 5.4, 4.3 Hz, 2H), 3.55 (dd, J = 5.4, 4.3 Hz, 2H), 3.37 (s, 3H); ^{13}C NMR (101 MHz, $CDCl_3$) δ 167.6, 134.6, 131.5, 128.6 (2 × CH), 127.1 (2 × CH), 71.3, 58.9, 39.8; LCMS (EI) m/z: $[M + H]^+$ Calcd for $C_{10}H_{14}NO_2$ 180.2; Found 180.2 at 6.18 mins. This compound was known in the literature and spectral data agreed with that of the reported values.[16]

N-(Adamantan-1-yl)benzamide (3i)

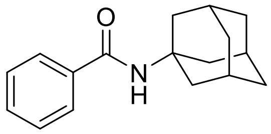

The reaction was performed as described in general procedure C using 2-(2,4-dinitrophenyl)-5-phenyl-2*H*-tetrazole (**1a**) (100 mg, 320 µmol) and adamantylamine (49.0 mg, 324 µmol). The resulting solution was stirred for 18 hours. Purification by flash column chromatograph using a gradient system (pet. ether/Et_2O100:1 to 4:1) afforded *N*-(adamantan-1-yl)benzamide (**3i**) (41.8 mg, 51%) as an off-white solid. R*f* = 0.20 (pet. ether/Et_2O 4:1); ^{1}H NMR (500 MHz, $CDCl_3$) δ 7.73–7.67 (m, 2H), 7.46–7.41 (m, 1H), 7.40–7.34 (m, 2H), 5.84 (br s, 1H), 2.12 (s, 9H), 1.76–1.65 (m, 6H); ^{13}C NMR (126 MHz, $CDCl_3$) δ 166.7, 136.1, 131.1, 128.5 (2 × CH), 126.8 (2 × CH), 52.3, 41.7 (3 × CH), 36.5 (3 × CH_2), 29.6 (3 × CH_2); LCMS (ESI) m/z: $[M + H]^+$ Calcd for $C_{17}H_{22}NO$ 256.2; Found 256.3 at 8.98 mins. This compound was known in the literature and spectral data are agreed with that of reported values.[17]

Benzoyl-*L*-phenylalanine (3j)

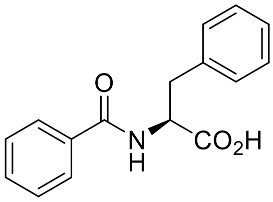

The reaction was performed as described in general procedure C using 2-(2,4-dinitrophenyl)-5-phenyl-2*H*-tetrazole (**1a**) (50.0 mg, 160 µmol), *L*-phenylalanine (26.4 mg, 160 µmol) and triethylamine (25.0 µL,160 µmol) in dioxane. The resulting solution was stirred for 72 hours and then concentrated *in vacuo*. The crude residue was purified by reverse phase column chromatography on a SB-C18 column using a gradient system (H_2O (0.1% TFA)/MeCN (0.1% TFA); 5–95%) to afford benzoyl-*L*-phenylalanine (**3j**) (16.9 mg, 39%) as a colourless solid. R*f* = 0.50 (CH_2Cl_2/MeOH 9:1); ^{1}H NMR (500 MHz, D_6-DMSO) δ 12.74 (br s, 1H), 8.66 (d, *J* = 8.1 Hz, 1H), 7.81–7.75 (m, 2H), 7.52 (t, *J* = 7.3 Hz, 1H), 7.44 (t, *J* = 7.6 Hz, 2H), 7.31 (d, *J* = 7.6 Hz, 2H), 7.26 (t, *J* = 7.5 Hz, 2H), 7.17 (t, *J* = 7.3 Hz, 1H), 4.61 (ddd, *J* = 10.6, 8.1, 4.5 Hz, 1H), 3.19 (dd, *J* = 13.8, 4.5 Hz, 1H), 3.07 (dd, *J* = 13.8, 10.6 Hz, 1H); ^{13}C NMR (126 MHz, D_6-DMSO) δ 173.2, 166.3, 138.2, 133.9, 131.3, 129.0 (2 × CH), 128.2 (2 × CH), 128.1 (2 × CH), 127.3 (2 × CH), 126.3, 54.2, 36.2; LCMS (EI) m/z: $[M + H]^+$ Calcd for $C_{16}H_{16}NO_3$ 270.3; Found

270.2 at 6.05 mins; $[\alpha]_D^{20}$ = –75 (c 0.05, MeOH). This compound was known in the literature and spectral data agreed with that of the reported values.[18]

Benzamide (3k)

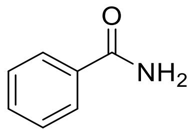

The reaction was performed as described in general procedure C using 2-(2,4-dinitrophenyl)-5-phenyl-2*H*-tetrazole (**1a**) (100 mg, 320 µmol) and ammonia (16.5 µL, 320 µmol, as a 33% aqueous solution). The resulting solution was stirred for 45 minutes to afford benzylamide (**3k**) (20.4 mg, 53%) as a yellow solid. R*f* = 0.15 (pet. ether/EtOAc 2:1); ^{1}H NMR (400 MHz, $CDCl_3$) δ 7.89–7.76 (m, 2H), 7.59–7.49 (m, 1H), 7.49–7.38 (m, 2H), 6.03 (br s, 1H), 5.62 (br s, 1H); ^{13}C NMR (101 MHz, $CDCl_3$) δ 169.5, 132.2, 128.8 (2 × CH), 127.5 (2 × CH); LCMS (EI) m/z: $[M + H]^+$ Calcd for C_7H_8NO 122.2; Found 122.2 at 4.90 mins. This compound was known in the literature and spectral data are agreed with that of reported values.[19]

***N*,*N*-Dimethylbenzamide (3l)**

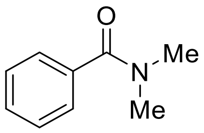

The reaction was performed as described in general procedure C using 2-(2,4-dinitrophenyl)-5-phenyl-2*H*-tetrazole (**1a**) (100 mg, 320 µmol) and using dimethylamine hydrochloride (26.0 mg, 319 µmol). The resulting solution was stirred for 16 hours to afford *N*,*N*-dimethylbenzamide (**3l**) (16.4 mg, 34%) as a brown oil. R*f* = 0.15 (pet. ether/EtOAc 2:1); ^{1}H NMR (500 MHz, D_6-DMSO) δ 7.45–7.41 (m, 3H), 7.41–7.35 (m, 2H), 2.98 (s, 3H), 2.90 (s, 3H); ^{13}C NMR (126 MHz, D_6-DMSO) δ 170.1, 136.5, 129.3, 128.2, 126.8, 34.4 (Methyl peak present under D_6-DMSO solvent peak); LCMS (EI) m/z: $[M + H]^+$ Calcd for $C_9H_{12}NO$ 150.2; Found 150.2 at 5.99 mins. This compound was known in the literature and spectral data are agreed with that of reported values.[20]

***N*-Methoxy-*N*-methylbenzamide (3m)**

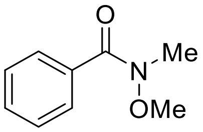

The reaction was performed as described in general procedure C using 2-(2,4-dinitrophenyl)-5-phenyl-2*H*-tetrazole (**1a**) (100 mg, 320 µmol). *N*,*O*-dimethylhydroxylamine hydrochloride (31.2 mg, 320 µmol) and triethylamine (45.0 µL, 323 µmol). The resulting solution was stirred for 2 hours to afford *N*-methoxy-*N*-methylbenzamide (**3m**) (25.3 mg, 48%) as a yellow oil. R*f* = 0.20 (pet. ether/EtOAc 3:2); ^{1}H NMR (500 MHz, $CDCl_3$) δ 7.68–7.64 (m, 2H), 7.47–7.42 (m, 1H), 7.41–7.36 (m, 2H), 3.55 (s, 3H), 3.35 (s, 3H); ^{13}C NMR (126 MHz, $CDCl_3$) δ 170.1, 134.3, 130.7, 128.2 (2 × CH), 128.1 (2 × CH), 61.1, 33.9; LCMS (ESI) m/z: $[M + H]^+$ Calcd for $C_9H_{12}NO_2$ 166.1; Found 166.2 at 6.24 mins. This compound was known in the literature and spectral data are agreed with that of reported values.[21]

***N*-Butyl-*N*-methylbenzamide (3n)**

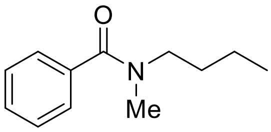

The reaction was performed as described in general procedure C using 2-(2,4-dinitrophenyl)-5-phenyl-2*H*-tetrazole (**1a**) (100 mg, 320 µmol) and *N*-methylbutylamine (38.0 µL, 321 µmol). The resulting solution was stirred for 2 hours to afford *N*-butyl-*N*-methylbenzamide (**3n**) (56.5 mg, 92%) as a yellow oil. R*f* = 0.20 (pet. ether/EtOAc 3:2); ^{1}H NMR (500 MHz, D_6-DMSO, 100 °C) δ 7.44–7.38 (m, 3H), 7.37–7.31 (m, 2H), 3.38–3.27 (m, 2H), 2.92 (s, 3H), 1.59–1.50 (m, 2H), 1.31–1.20 (m, 2H), 0.86 (t, *J* = 6.9 Hz, 3H); ^{13}C NMR (126 MHz, D_6-DMSO, 100 °C) δ 169.8, 136.8, 128.4, 127.6 (2 × CH), 125.9 (2 × CH), 47.8, 42.7, 28.7, 18.7, 12.8; LCMS (ESI) m/z: $[M + H]^+$ Calcd for $C_{12}H_{18}NO$ 192.1; Found 192.3 at 7.58 mins. This compound was known in the literature and spectral data are agreed with that of reported values.[22]

***N*-Benzyl-N-ethylbenzamide (3o)**

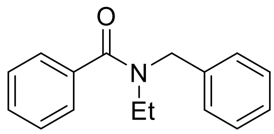

The reaction was performed as described in general procedure C using 2-(2,4-dinitrophenyl)-5-phenyl-2*H*-tetrazole (**1a**) (100 mg, 320 µmol) and (±)-ethylbenzylamine (46.0 µL, 319 µmol). The resulting solution was stirred for 16 hours to afford *N*-benzyl-*N*-ethylbenzamide (**3o**) (47.1 mg, 62%) as a brown oil. R*f* = 0.48 (pet. ether/EtOAc 4:1); IR vmax (film): 3046, 2933, 1636, 1505, 1430, 1372, 1291, 1161, 1079, 1033, 981, 925, 787 cm^{-1}; ^{1}H NMR (500 MHz, D_6-DMSO, 100 °C) δ 7.48–7.38 (m, 5H), 7.38–7.33 (m, 2H), 7.32–7.25 (m, 3H), 4.61 (s, 2H), 3.30 (q, *J* = 7.1 Hz, 2H), 1.06 (t, *J* = 7.1 Hz, 3H); ^{13}C NMR (126 MHz, D_6-DMSO, 100 °C) δ 170.2, 137.3, 136.6, 128.5, 127.9 (2 × CH), 127.8 (2 × CH), 126.8 (2 × CH), 126.5, 125.7 (2 × CH), 48.4, 41.0, 12.4; HRMS (ESI) m/z: $[M + H]^+$ Calcd for $C_{16}H_{18}NO$ 240.1383; Found 240.1385.

Phenyl(piperidin-1-yl)methanone (3p)

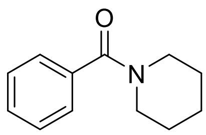

The reaction was performed as described in general procedure C using 2-(2,4-dinitrophenyl)-5-phenyl-2*H*-tetrazole (**1a**) (100 mg, 320 µmol) and piperidine (32.0 µL, 324 µmol). The resulting solution was stirred for 72 hours to afford phenyl(piperidin-1-yl)methanone (**3p**) (56.7 mg, 94%) as a brown oil. R*f* = 0.21 (pet. ether/EtOAc 4:1); ^{1}H NMR (500 MHz, D_6-DMSO) δ 7.46–7.40 (m, 3H), 7.38–7.33 (m, 2H), 3.57 (br s, 2H), 3.24 (br s, 2H), 1.65–1.57 (m, 2H), 1.54 (br s, 2H), 1.45 (br s, 2H); ^{13}C NMR (101 MHz, D_6-DMSO) δ 168.8, 136.6, 129.2, 128,4 (2 × CH), 126.5 (2 × CH), 48.0, 42.3, 25.9, 25.3, 24.0; LCMS (EI) m/z: $[M + H]^+$ Calcd for $C_{12}H_{16}NO$ 190.3; Found 190.3 at 7.12 mins. This compound was known in the literature and spectral data are agreed with that of reported values.[23]

Morpholino(phenyl)methanone (3q)

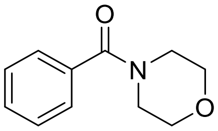

The reaction was performed as described in general procedure C using 2-(2,4-dinitrophenyl)-5-phenyl-2*H*-tetrazole (**1a**) (100 mg, 320 µmol) and morpholine (28.0 µL, 320 µmol). The resulting solution was stirred for 72 hours to afford morpholino(phenyl)methanone (**3q**) (46.3 mg, 76%) as a brown oil. R*f* = 0.22 (pet. ether/EtOAc 4:1); IR vmax (oil): 551, 598, 639, 713, 734, 792, 846, 896, 938, 1020, 1073, 1119, 1161, 1264, 1284, 1307,1371, 1433, 1587, 1637, 2866, 2933 cm^{-1}; ^{1}H NMR (400 MHz, $CDCl_3$) δ 7.49–7.33 (m, 5H), 3.71 (br s, 6H), 3.46 (br s, 2H); ^{13}C NMR (101 MHz, $CDCl_3$) δ 170.6, 135.5, 130.0, 128.7 (2 × CH), 127.7 (2 × CH), 67.0 (2 × CH_2), 48.4, 42.7; LCMS (EI) m/z: $[M + H]^+$ Calcd for $C_{11}H_{14}NO_2$ 192.2; Found 192.2 at 5.85 mins. This compound was known in the literature and spectral data are agreed with that of reported values.[24]

***N*-(4-Methoxyphenyl)benzamide (3r)**

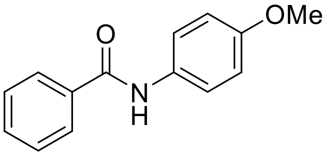

The reaction was performed as described in general procedure C using 2-(2,4-dinitrophenyl)-5-phenyl-2*H*-tetrazole (**1a**) (100 mg, 320 µmol) and 4-methoxyaniline (39.4 mg, 320 µmol). The resulting solution was stirred for 45 minutes to afford *N*-(4-methoxyphenyl)benzamide (**3r**) (67.4 mg, 93%) as a brown solid. R*f* = 0.65 (pet. ether/EtOAc 4:1); ^{1}H NMR (500 MHz, D_6-DMSO) δ 10.11 (s, 1H), 7.97–7.92 (m, 2H), 7.68 (d, *J* = 9.1 Hz, 2H), 7.60–7.49 (m, 3H), 6.93 (d, *J* = 9.1 Hz, 2H), 3.75 (s, 3H); ^{13}C NMR (126 MHz, D_6-DMSO) δ 165.1, 155.5, 135.0, 132.2, 131.3, 128.3 (2 × CH), 127.5 (2 × CH), 121.9 (2 × CH), 113.7 (2 × CH), 55.2; LCMS (EI) m/z: $[M + H]^+$ Calcd for $C_{14}H_{14}NO_2$ 228.3; Found 228.2 at 7.43 mins. This compound was known in the literature and spectral data are agreed with that of reported values.[25]

***N*-Benzyl-4-methoxybenzamide (4a)**

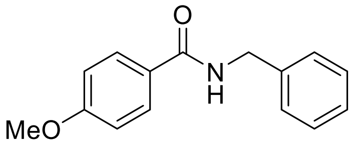

The reaction was performed as described in general procedure C using 2-(2,4-dinitrophenyl)-5-(4-methoxyphenyl)-2*H*-tetrazole (**1b**) (100 mg, 292 µmol) and benzyl amine (32.0 µL, 293 µmol). The resulting solution was stirred for 2 hours. Trituration from Et_2O afforded *N*-benzyl-4-methoxybenzamide (**4a**) (70.4 mg, quant.) as an off-white solid. R*f* = 0.33 (pet. ether/EtOAc 7:3); ^{1}H NMR (500 MHz, $CDCl_3$) δ 7.76 (d, *J* = 8.8 Hz, 2H), 7.36–7.32 (m, 4H), 7.31–7.26 (m, 1H), 6.90 (d, *J* = 8.8 Hz, 2H), 6.43 (br s, 1H), 4.62 (d, *J* = 5.6 Hz, 2H), 3.84 (s, 3H).; ^{13}C NMR (101 MHz, $CDCl_3$) δ (101 MHz, $CDCl_3$) δ 167.1, 162.4, 138.5, 128.9 (2 × CH), 128.9 (2 × CH), 128.0 (2 × CH), 127.7, 126.8, 113.9 (2 × CH), 55.5, 44.2; LCMS (EI) m/z: $[M + H]^+$ Calcd for $C_{15}H_{16}NO_2$ 242.1; Found 242.2 at 7.54 mins. This compound was known in the literature and spectral data are agreed with that of reported values.[26]

***N*-Benzyl-3,4,5-trimethoxybenzamide (4b)**

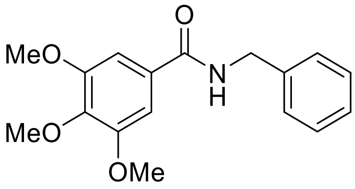

The reaction was performed as described in general procedure C using 2-(2,4-dinitrophenyl)-5-(3,4,5-trimethoxyphenyl)-2*H*-tetrazole (**1c**) (100 mg, 249 µmol) and benzylamine (27.0 µL, 247 µmol). The resulting solution was stirred for 2 hours. Purification by flash column chromatography, using a gradient system (pet. ether/EtOAc 1:1 to 3:7) followed by trituration from Et_2O afforded *N*-benzyl-3,4,5-trimethoxybenzamide (**4b**) (55.0 mg, 74%) as an off-white solid. R*f* = 0.10 (pet. ether/EtOAc 3:2); ^{1}H NMR (400 MHz, $CDCl_3$) δ 7.39–7.33 (m, 4H), 7.33–7.27 (m, 1H), 7.01 (s, 2H), 6.42 (t, *J* = 5.7 Hz 1H), 4.63 (d, *J* = 5.7 Hz, 2H), 3.88 (s, 6H), 3.87 (s, 3H); ^{13}C NMR (101 MHz, $CDCl_3$) δ 167.3, 153.4 (2 × C), 141.2, 138.3, 129.9, 129.0 (2 × CH), 128.1 (2 × CH), 127.8, 104.6 (2 × CH), 61.0, 56.5 (2 × CH_3), 44.4; LCMS (ESI) m/z: $[M + H]^+$ Calcd for $C_{17}H_{20}NO_4$ 302.1; Found 302.3 at 7.61 mins. This compound was known in the literature and spectral data are agreed with that of reported values.[27]

_N_-Benzylbenzo[_d_][1,3]dioxole-5-carboxamide (4c)

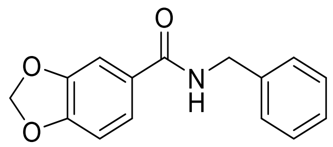

The reaction was performed as described in general procedure C using 5-(benzo[_d_][1,3]dioxol-5-yl)-2-(2,4-dinitrophenyl)-2_H_-tetrazole (**1d**) (100 mg, 281 µmol) and benzylamine (31.0 µL, 284 µmol). The resulting solution was stirred for 2 hours. Triturated from Et_2O afforded _N_-benzylbenzo[_d_][1,3]dioxole-5-carboxamide (**4c**) (52.0 mg, 72%) as an off-white solid. R_f_ = 0.24 (pet. ether/EtOAc 3:2); ^{1}H NMR (400 MHz, $CDCl_3$) δ 7.38–7.31 (m, 4H), 7.31–7.28 (m, 1H), 7.28–7.26 (m, 2H), 6.80 (d, _J_ = 8.0 Hz, 1H), 6.33 (br s, 1H), 6.01 (s, 2H), 4.61 (d, _J_ = 5.7 Hz, 2H); ^{13}C NMR (101 MHz, $CDCl_3$) δ 167.0, 150.6, 148.2, 138.2, 128.9 (2 × CH), 128.6, 128.0 (2 × CH), 127.8, 121.7, 108.2, 107.8, 101.9, 44.4; LCMS (ESI) m/z: $[M + H]^+$ Calcd for $C_{15}H_{14}NO_3$ 256.1; Found 256.2 at 7.31 mins. This compound was known in the literature and spectral data are agreed with that of reported values.[28]

_S_-Ethyl benzylcarbamothioate (4d)

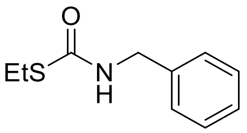

The reaction was performed as described in general procedure C using 2-(2,4-dinitrophenyl)-5-(ethylthio)-2_H_-tetrazole (**1e**) (50.0 mg, 169 µmol) and benzylamine (18.5 µL, 169 µmol). The resulting solution was stirred for 2 hours. Purification by flash column chromatography using a gradient system (pet. ether/Et_2O 9:1 to 2:1) afforded _S_-ethyl benzylcarbamothioate (**4d**) (13.7 mg, 42%) as an off-white solid. R_f_ = 0.55 (pet. ether/EtOAc 2:1); ^{1}H NMR (400 MHz, $CDCl_3$) δ 7.38–7.31 (m, 2H), 7.31–7.26 (m, 3H), 5.60 (br s, 1H), 4.47 (d, _J_ = 5.8 Hz, 2H), 2.95 (q, _J_ = 7.4 Hz, 2H), 1.31 (t, _J_ = 7.4 Hz, 3H); ^{13}C NMR (101 MHz, $CDCl_3$) δ 167.6, 137.9, 128.9 (2 × CH), 127.9 (2 × CH), 127.8, 45.4, 24.6, 15.8; LCMS (EI) m/z: $[M + H]^+$ Calcd for $C_{10}H_{14}NOS$ 196.3; Found 196.4 at 6.84 mins. This compound was known in the literature and spectral data agreed with that of the reported values.[29]

N-Benzyl-5-bromothiophene-2-carboxamide (4e)

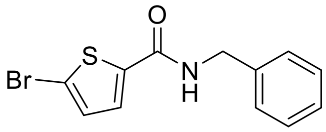

The reaction was performed as described in general procedure C using 2-(2,4-dinitrophenyl)-5-(ethylthio)-2*H*-tetrazole (1f) (100 mg, 252 µmol) and benzylamine (27.6 µL, 253 µmol). The resulting solution was stirred for 2 hours. Purification by flash column chromatography using a gradient system (pet. ether/EtOAc 9:1 to 2:1) afforded *N*-benzyl-5-bromothiophene-2-carboxamide (**4e**) (40.6 mg, 54%) as an off-white solid. R*f* = 0.20 (pet. ether/Et_2O 2:1); ^{1}H NMR (500 MHz, $CDCl_3$) δ 7.39–7.27 (m, 5H), 7.23 (d, *J* = 4.0 Hz, 1H), 7.02 (d, *J* = 4.0 Hz, 1H), 6.26 (br s, 1H), 4.58 (d, *J* = 5.8 Hz, 2H); ^{13}C NMR (126 MHz, $CDCl_3$) δ 160.9, 140.4, 137.9, 130.8, 129.0 (2 × CH), 128.2, 128.1 (2 × CH), 127.9, 118.2, 44.2; HRMS (ESI) m/z: $[M + H]^+$ Calcd for $C_{12}H_{11}BrNOS$ 295.9739; Found 295.9735.

N-Benzyl-4-chlorobenzamide (4f)

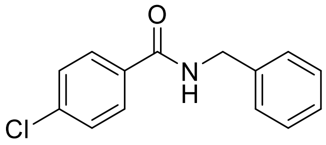

The reaction was performed as described in general procedure C using 5-(4-chlorophenyl)-2-(2,4-dinitrophenyl)-2*H*-tetrazole (**1g**) (100 mg, 288 µmol) and benzylamine (31.5 µL, 288 µmol). The resulting solution was stirred for 2 hours. Trituration from Et_2O afforded *N*-benzyl-4-chlorobenzamide (**4f**) (55.0 mg, 78%) as a light brown solid. R*f* = 0.41 (pet. ether/EtOAc 7:3); ^{1}H NMR (400 MHz, $CDCl_3$) δ 7.73 (d, *J* = 8.6 Hz, 2H), 7.40 (d, *J* = 8.6 Hz, 2H), 7.38–7.28 (m, 5H), 6.36 (br s, 1H), 4.64 (d, *J* = 5.6 Hz, 2H); ^{13}C NMR (101 MHz, $CDCl_3$) δ 166.4, 138.1, 138.0, 132.9, 129.0 (4 × CH), 128.5 (2 × CH), 128.1 (2 × CH), 127.9, 44.4;); LCMS (EI) m/z: $[M + H]^+$ Calcd for $C_{14}H_{13}ClNO$ 246.7; Found 246.2 at 7.06 mins. This compound was known in the literature and spectral data agreed with that of the reported values.[30]

***N*-Benzyl-4-nitrobenzamide (4g)**

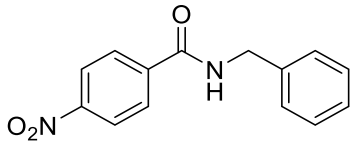

The reaction was performed as described in general procedure C using 2-(2,4-dinitrophenyl)-5-(4-nitrophenyl)-2*H*-tetrazole (**1h**) (100 mg, 280 µmol) and benzylamine (30.6 µL, 280 µmol). The resulting solution was stirred for 2 hours to afford *N*-benzyl-4-nitrobenzamide (**4g**) (56.0 mg, 78%) as a light brown solid. R*f* = 0.47 (pet. ether/EtOAc 7:3); ^{1}H NMR (400 MHz, $CDCl_3$) δ 8.33–8.25 (m, 2H), 7.97–7.93 (m, 2H), 7.41–7.30 (m, 5H), 6.41 (br s, 1H), 4.67 (d, *J* = 5.7 Hz, 2H); ^{13}C NMR (101 MHz, $CDCl_3$) δ165.4, 149.8, 140.1, 137.6, 129.1 (2 × CH), 128.3 (2 × CH), 128.2 (2 × CH), 128.2, 124.0 (2 × CH), 44.7; LCMS (EI) m/z: $[M - H]^-$ Calcd for $C_{14}H_{13}N_2O_3$ 255.1; Found 255.1 at 7.79 mins. This compound was known in the literature and spectral data agreed with that of the reported values.[26]

***N*-Benzyl-2,6-dichlorobenzamide (4h)**

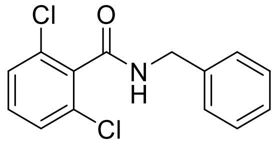

The reaction was performed as described in general procedure C using 5-(2,6-dichlorophenyl)-2-(2,4-dinitrophenyl)-2*H*-tetrazole (**1i**) (50.0 mg, 131 µmol) and benzylamine (15.0 µL, 137 µmol). The resulting solution was stirred for 16 hours. Trituration from Et_2O afforded *N*-benzyl-2,6-dichlorobenzamide (**4h**) (36.7 mg, quant.) as a light brown solid. Rf = 0.35 (pet. ether/EtOAc 7:3); 1H NMR (500 MHz, $CDCl_3$) δ 7.39–7.23 (m, 7H), 7.23–7.18 (m, 1H), 6.05 (br s, 1H), 4.63 (d, *J* = 5.7 Hz, 2H); 13C NMR (126 MHz, $CDCl_3$) δ 164.5, 137.4, 136.0, 132.4 (2 × C), 130.8, 128.8 (2 × CH), 128.2 (2 × CH), 128.2 (2 × CH), 127.9, 44.2; LCMS (EI) m/z: $[M + H]^+$ Calcd for $C_{14}H_{12}Cl_2NO$ 280.1; Found 280.1 at 8.10 mins. This compound was known in the literature and spectral data are agreed with that of reported values.[26]

***N*-Benzyl-4-bromobenzamide (4i)**

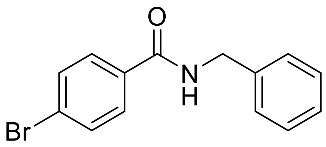

The reaction was performed as described in general procedure C using 5-(4-bromophenyl)-2-(2,4-dinitrophenyl)-2*H*-tetrazole (**1j**) (100 mg, 256 µmol) and benzylamine (28.0 µL, 256 µmol). The resulting solution was stirred for 16 hours to afford *N*-benzyl-4-bromobenzamide (**4i**) (57.8 mg, 78%) as a brown solid; ^{1}H NMR (500 MHz, D_6-DMSO) δ 9.11 (t, *J* = 5.9 Hz, 1H), 7.84 (d, *J* = 8.4 Hz, 2H), 7.69 (d, *J* = 8.4 Hz, 2H), 7.36–7.28 (m, 4H), 7.27–7.19 (m, 1H), 4.47 (d, *J* = 5.9 Hz, 2H); ^{13}C NMR (126 MHz, D_6-DMSO) δ 165.2, 139.4, 133.4, 131.3 (2 × CH), 129.4 (2 × CH), 128.3 (2 × CH), 127.2 (2 × CH), 126.8, 125.0, 42.7; LCMS (EI) m/z: $[M + H]^+$ Calcd for $C_{14}H_{13}BrNO$ 291.2; Found 291.2 at 7.89 mins. This compound was known in the literature and spectral data are agreed with that of reported values.[31]

***N*-Benzyl-3-bromobenzamide (4j)**

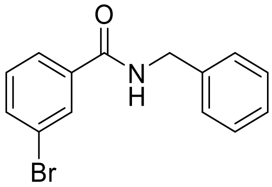

The reaction was performed as described in general procedure C using 5-(3-bromophenyl)-2-(2,4-dinitrophenyl)-2*H*-tetrazole (**1k**) (86.0 mg, 220 µmol) and benzylamine (24.0 µL, 220 µmol). The resulting solution was stirred for 16 hours and then concentrated *in vacuo.* The crude residue was purified by reverse phase column chromatography on a SB-C18 column using a gradient system (H_2O (0.1% TFA)/MeCN (0.1% TFA); 5–95%) to afford *N*-benzyl-3-bromobenzamide (**4j**) (58.0 mg, 91%) as a yellow solid. ^{1}H NMR (500 MHz, $CDCl_3$) δ 7.93 (s, 1H), 7.70 (d, *J* = 7.8 Hz, 1H), 7.62 (d, *J* = 7.8 Hz, 1H), 7.39–7.27 (m, 6H), 6.41 (br s, 1H), 4.63 (d, *J* = 5.6 Hz, 2H); ^{13}C NMR (126 MHz, $CDCl_3$) 166.0, 138.0, 136.5, 134.7, 130.4, 130.3, 129.0 (2 × CH), 128.1 (2 × CH), 127.9, 125.7, 123.0, 44.4; HRMS (ESI) m/z: $[M + Na]^+$ Calcd for $C_{14}H_{12}BrNONa$ 311.9995; Found 311.9998. This compound was known in the literature and spectral data are agreed with that of reported values.[32]

***N*-Benzylacetamide (4k)**

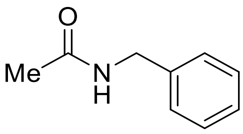

The reaction was performed as described in general procedure C using 2-(2,4-dinitrophenyl)-5-methyl-2*H*-tetrazole (**1l**) (200 mg, 799 µmol) and benzylamine (87.4 µL, 800 µmol). The resulting solution was stirred for 2 hours to afford *N*-benzylacetamide (**4k**) (95.8 mg, 80%) as a pale yellow oil. R*f* = 0.33 (CH_2Cl_2/MeOH 95:5); ^{1}H NMR (400 MHz, $CDCl_3$) δ 7.34–7.28 (m, 2H), 7.28–7.21 (m, 3H), 6.15 (br s, 1H), 4.38 (d, *J* = 5.7 Hz, 2H), 1.97 (s, 3H); ^{13}C NMR (101 MHz, $CDCl_3$) δ 170.2, 138.4, 128.7 (2 × CH), 127.9 (2 × CH), 127.5, 43.8, 23.2; LCMS (EI) m/z: $[M + H]^+$ Calcd for $C_9H_{12}NO$ 150.2; Found 150.2 at 6.52 mins. This compound was known in the literature and spectral data agreed with that of the reported values.[33]

***N*-Benzyl-2-phenylacetamide (4l)**

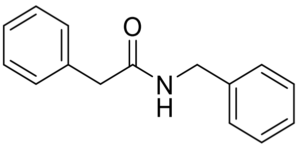

The reaction was performed as described in general procedure C using 5-benzyl-2-(2,4-dinitrophenyl)-2*H*-tetrazole (**1m**) (100 mg, 306 µmol) and benzylamine (33.5 µL, 307 µmol). The resulting solution was stirred for 3 hours. Purification by flash column chromatography using a gradient system (pet. ether/Et_2O 9:1 to 2:1) afforded *N*-benzyl-2-phenylacetamide (**4l**) (20.8 mg, 30%) as an off-white solid. R*f* = 0.71 (pet. ether/EtOAc 1:1); ^{1}H NMR (400 MHz, $CDCl_3$) δ 7.37–7.26 (m, 8H), 7.21–7.15 (m, 2H), 5.66 (br s, 1H), 4.42 (d, *J* = 5.8 Hz, 2H), 3.64 (s, 2H); ^{13}C NMR (101 MHz, $CDCl_3$) δ 171.0, 138.3, 134.9, 129.6 (2 × CH), 129.2 (2 × CH), 128.8 (2 × CH), 127.6 (2 × CH), 127.6 (2 × CH), 44.0, 43.8; LCMS (EI) m/z: $[M + H]^+$ Calcd for $C_{15}H_{16}NO$ 226.3; Found 226.2 at 6.67 mins. This compound was known in the literature and spectral data agreed with that of the reported values.[34]

***N*-Benzylhexanamide (4m)**

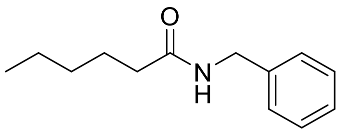

The reaction was performed as described in general procedure C using 2-(2,4-dinitrophenyl)-5-pentyl-2*H*-tetrazole (**1n**) (40.5 mg, 132 µmol) and benzylamine (15.0 µL, 137 µmol). The resulting solution was stirred for 1 hours to afford *N*-benzylhexanamide (**4m**) (23.8 mg, 88%) as an orange oil. R*f* = 0.20 (pet. ether/EtOAc, 3:2); ^{1}H NMR (500 MHz, $CDCl_3$) δ 7.28–7.23 (m, 2H), 7.22–7.17 (m, 3H), 5.77 (br s, 1H), 4.35 (d, *J* = 5.7 Hz, 2H), 2.13 (t, *J* = 7.7 Hz, 2H), 1.58 (quin., *J* = 7.7 Hz, 2H), 1.28–1.21 (m, 4H), 0.82 (t, *J* = 6.8 Hz, 3H); ^{13}C NMR (126 MHz, $CDCl_3$) δ 173.1, 138.6, 128.8 (2 × CH), 127.9 (2 × CH), 127.6, 43.7, 36.9, 31.6, 25.6, 22.5, 14.0; LCMS (ESI) m/z: $[M + H]^+$ Calcd for $C_{13}H_{20}NO$ 206.1; Found 206.3 at 7.01 mins. This compound was known in the literature and spectral data are agreed with that of reported values.[35]

4-Chloro-*N*-(2-morpholinoethyl)benzamide, moclobemide (5a)

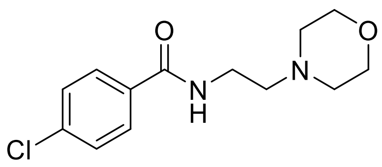

The reaction was performed as described in general procedure C using 5-(4-chlorophenyl)-2-(2,4-dinitrophenyl)-2*H*-tetrazole (**1g**) (100 mg, 288 µmol) and 4-(2-aminoethyl)morpholine (37.9 µL, 289 µmol). The resulting solution was stirred for 2 hours. Purification by flash column chromatography (EtOAc/MeOH 9:1) afforded 4-chloro-*N*-(2-morpholinoethyl)benzamide, moclobemide (**5a**) (56.8 mg, 73%) as an off-white solid. R*f* = 0.26 (EtOAc/MeOH 9:1); ^{1}H NMR (500 MHz, $CDCl_3$) δ 7.70 (d, *J* = 8.4 Hz, 2H), 7.39 (d, *J* = 8.4 Hz, 2H), 6.79 (br s, 1H), 3.70 (t, *J* = 4.5 Hz, 4H), 3.52 (app q, *J* = 5.7 Hz, 2H), 2.58 (t, *J* = 6.0 Hz, 2H), 2.48 (t, *J* = 4.3, 4H); ^{13}C NMR (126 MHz, $CDCl_3$) δ 166.4, 137.7, 133.1, 128.9 (2 × CH), 128.5 (2 × CH), 67.1 (2 × CH_2), 56.9, 53.4 (2 × CH_2), 36.2;); LCMS (EI) m/z: $[M + H]^+$ Calcd for $C_{13}H_{18}ClN_2O_2$ 269.1; Found 269.2 at 6.49 mins. This compound was known in the literature and spectral data agreed with that of the reported values.[26]

***N*-(2-(Diethylamino)ethyl)-4-nitrobenzamide (5b)**

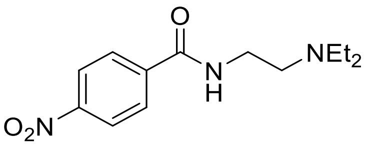

The reaction was performed as described in general procedure C using 2-(2,4-dinitrophenyl)-5-(4-nitrophenyl)-2*H*-tetrazole (**1h**) (100 mg, 280 µmol) and *N*,*N*-diethylethylenediamine (39.0 µL, 278 µmol). The resulting solution was stirred for 2 hours. Purification by flash column chromatography (CH_2Cl_2/MeOH 9:1) afforded *N*-(2-(diethylamino)ethyl)-4-nitrobenzamide (**5b**) (47.8 mg, 65%) as a viscous pale yellow oil. R*f* = 0.33 (CH_2Cl_2/MeOH 9:1); ^{1}H NMR (400 MHz, $CDCl_3$) δ 8.28 (d, *J* = 8.8 Hz, 2H), 7.99 (d, *J* = 8.8 Hz, 2H), 7.49 (br s, 1H), 3.57 (app q, *J* = 5.4 Hz, 2H), 2.79 (t, *J* = 5.4 Hz, 2H), 2.69 (q, *J* = 7.2 Hz, 4H), 1.11 (t, *J* = 7.2 Hz, 6H); ^{13}C NMR (101 MHz, $CDCl_3$) δ 165.4, 149.7, 140.2, 128.4 (2 × CH), 123.9 (2 × CH), 51.5, 47.2 (2 × CH_2), 37.2, 11.5 (2 × CH_3); LCMS (EI) m/z: $[M + H]^+$ Calcd for $C_{13}H_{20}N_3O_3$ 266.3; Found 266.2 at 5.54 mins. This compound was known in the literature and spectral data agreed with that of the reported values.[26]

***N*-(4-(2-(Dimethylamino)ethoxy)benzyl)-3,4-dimethoxybenzamide, Itopride (5c)**

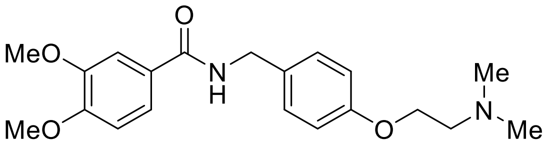

The reaction was performed as described in general procedure C using 5-(3,4-dimethoxyphenyl)-2-(2,4-dinitrophenyl)-2*H*-tetrazole (**1o**) (100 mg, 269 µmol) and 4-(2-dimethylamino)-ethoxybenzylamine (52.0 mg, 268 µmol). The resulting solution was stirred for 18 hours. The reaction mixture was diluted with EtOAc (30 mL) and washed with 2 M aqueous HCl (3 × 25 mL). The combined aqueous layer was adjusted to approximately pH 12 by the addition of 4 M aqueous NaOH and extracted with EtOAc (3 × 50 mL), dried over $MgSO_4$, filtered and concentrated *in vacuo.* Trituration from Et_2O afforded *N*-(4-(2-(dimethylamino)ethoxy)benzyl)-3,4-dimethoxybenzamide, Itopride (**5c**) (68.9 mg, 72%) as a beige solid. *Rf* = 0.10 (CH_2Cl_2/MeOH, 9:1); ^{1}H NMR (500 MHz, $CDCl_3$) δ 7.37 (d, *J* = 1.8 Hz, 1H), 7.23–7.16 (m, 3H), 6.81 (d, *J* = 8.6 Hz, 2H), 6.75 (d, *J* = 8.4 Hz, 1H), 6.38 (t, *J* = 5.7 Hz, 1H), 4.47 (d, *J* = 5.7 Hz, 2H), 3.98 (t, *J* = 5.7 Hz, 2H), 3.83 (s, 3H), 3.82 (s, 3H), 2.66 (t, *J* = 5.7 Hz, 2H), 2.26 (s, 6H); ^{13}C NMR (126 MHz, $CDCl_3$) δ 166.9, 158.4, 151.8, 149.1, 130.7,

129.3 (2 × CH), 128.5, 127.2, 119.4, 114.9 (2 × CH), 110.8, 110.4, 66.1, 58.4, 56.1, 56.1, 46.0, 43.7; LCMS (ESI) m/z: $[M + H]^+$ Calcd for $C_{20}H_{27}N_2O_4$ 359.2; Found 359.4 at 5.45 mins. This compound was known in the literature and spectral data are agreed with that of reported values.[36]

Indolin-1-yl(3,4,5-trimethoxyphenyl)methanone (5d)

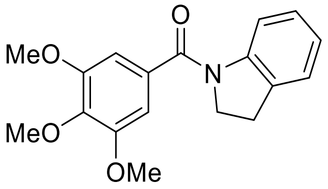

The reaction was performed as described in general procedure **C** using 2-(2,4-dinitrophenyl)-5-(3,4,5-trimethoxyphenyl)-2*H*-tetrazole (**1c**) (100 mg, 249 µmol) and indoline (28.0 µL, 250 µmol). The resulting solution was stirred for 18 hours. Purification by flash column chromatography using a gradient system (pet. Ether/EtOAc 4:1 to 3:2) afforded indolin-1-yl(3,4,5-trimethoxyphenyl)methanone (**5d**) (41.6 mg, 53%) as an off-white solid. R*f* = 0.28 (pet. ether/EtOAc, 1:1); ^{1}H NMR (500 MHz, $CDCl_3$) δ 7.89 (br s, 1H), 7.21 (d, *J* = 7.5 Hz, 1H), 7.11 (br s, 1H), 7.01 (t, *J* = 7.3 Hz, 1H), 6.77 (s, 2H), 4.11 (t, *J* = 8.3 Hz, 2H), 3.89 (s, 3H), 3.85 (s, 6H), 3.12 (t, *J* = 8.3 Hz, 2H); ^{13}C NMR (126 MHz, $CDCl_3$) δ 168.7, 153.5 (2 × C), 142.6, 139.9, 132.7, 132.2, 127.3, 125.1, 124.1, 117.0, 104.6 (2 × CH), 61.1, 56.4 (2 × CH_3), 50.7, 28.1; LCMS (ESI) m/z: $[M + H]^+$ Calcd for $C_{18}H_{20}NO_4$ 314.1; Found 314.2 at 7.83 mins. This compound was known in the literature and spectral data are agreed with that of reported values.[37]

Benzo[*c*][1,2,5]oxadiazol-5-yl(piperidin-1-yl)methanone, Farampator (5e)

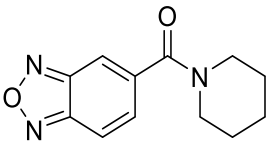

The reaction was performed as described in general procedure C using 5-(2-(2,4-dinitrophenyl)-2*H*-tetrazol-5-yl)benzo[*c*][1,2,5]oxadiazole (**1p**) (100 mg, 282 µmol) and piperidine (28.0 µL, 283 µmol). The resulting solution was stirred for 18 hours. Purification by flash column chromatography using a gradient system (CH_2Cl_2/MeOH 100:0 to 19:1). Further purification by flash column chromatography (pet. Ether/(EtOAc/EtOH 3:1) 4:1) afforded

benzo[*c*][1,2,5]oxadiazol-5-yl(piperidin-1-yl)methanone, Farampator (**5e**) (39.3 mg, 60%) as an orange solid. R*f* = 0.15 (pet. ether/EtOAc, 3:2); ^{1}H NMR (400 MHz, $CDCl_3$) δ 7.86 (dd, *J* = 9.2, 1.2 Hz, 1H), 7.80 (t, *J* = 1.2 Hz, 1H), 7.40 (dd, *J* = 9.2, 1.2 Hz, 1H), 3.71 (br s, 2H), 3.37 (br s, 2H), 1.77–1.47 (m, 6H); ^{13}C NMR (101 MHz, $CDCl_3$) δ 167.5, 148.7, 148.6, 139.6, 130.8, 117.4, 114.5, 48.8, 43.4, 26.6, 25.5, 24.4; HRMS (ESI) m/z: $[M + H]^+$ Calcd for $C_{12}H_{14}N_3O_2$ 232.1081; Found 232.1084.

***N*-(Benzo[*d*][1,3]dioxol-5-ylmethyl)-2,6-dichlorobenzamide, ALDA-1 (5f)**

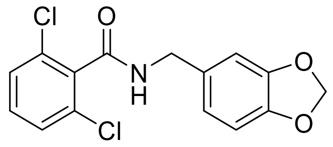

The reaction was performed as described in general procedure C using 5-(2,6-dichlorophenyl)-2-(2,4-dinitrophenyl)-2*H*-tetrazole (**1i**) (100 mg, 262 µmol) and 3,4-(methylenedioxy)benzylamine (33.0 µL, 265 µmol). The resulting solution was stirred for 18 hours. Trituration from Et_2O afforded *N*-(benzo[*d*][1,3]dioxol-5-ylmethyl)-2,6-dichlorobenzamide, ALDA-1 (**5f**) (65.9 mg, 78%) as a beige solid. R*f* = 0.28 (pet. ether/EtOAc, 3:2); ^{1}H NMR (400 MHz, $CDCl_3$) δ 7.37–7.32 (m, 2H), 7.29 (dd, *J* = 8.4, 2.5 Hz, 1H), 6.94 (d, *J* = 1.7 Hz, 1H), 6.88 (dd, *J* = 7.9, 1.7 Hz, 1H), 6.81 (d, *J* = 7.9 Hz, 1H), 6.02 (br s, 1H), 5.99 (s, 2H), 4.62 (d, *J* = 5.8 Hz, 2H); ^{13}C NMR (101 MHz, $CDCl_3$) δ 164.4, 148.1, 147.3, 136.0, 132.5, 131.2 (2 × C), 130.8, 128.2 (2 × CH), 121.6, 108.8, 108.5, 101.3, 44.0; LCMS (ESI) m/z: $[M + H]^+$ Calcd for $C_{15}H_{12}Cl_2NO_3$ 324.0; Found 324.1 at 7.65 mins. This compound was known in the literature and spectral data are agreed with that of reported values.[38]

3. General Experimental for Peptide Synthesis

All amino acids and coupling reagents were obtained from commercial sources and used as received without further purification. *N,N'*-dimethylformamide, dichloromethane, piperidine and acetic anhydride were obtained from commercial suppliers and used without further purification.

Peptides were synthesised manually using a Merrifield bubbler, attached to a vacuum line, a nitrogen line and a round-bottom flask for waste.

Experimental Procedures for Peptides

All peptides were assembled onto Wang resin. The first amino acid residue was converted to the active, symmetrical amino acid anhydride by dissolving the amino acid (1 equiv.) in anhydrous DMF (2 mL/mmol). DIC (1 equiv.) dissolved in minimal anhydrous DMF was added to the flask and the mixture stirred at 0 °C for 10 minutes. The reaction mixture was then allowed to warm to room temperature and stirred for a further 10 minutes. The anhydride formed was manually loaded onto the resin using a Merrifield bubbler, attached to a vacuum line, a nitrogen line and a round bottom flask for waste (Table 1).

Table 1: Procedure for the manual attachment of the first amino acid residue onto Wang resin

Step	Solvent/Reagents	Volume (mL)	Time (min)	Mixing	Iterations
**Swell**	DCM	9	30	-	-
**Wash**	DMF	9	0.5	N_2 bubbling	5
**Esterification**	Amino acid anhydride (5 equiv.), DMAP (5 equiv.), DMF	9	120	N_2 bubbling	-
**Wash**	DMF	9	0.5	N_2 bubbling	5
**Wash**	DCM	9	0.5	N_2 bubbling	5
**Wash**	Diethyl ether	9	0.5	N_2 bubbling	5
**Vacuum Dry**	-	-	30	-	-
**Loading Test**	20 % piperidine in DMF	10	15	Sonication	2
**Swell**	DCM	9	30	-	-

**Wash**	DMF	9	0.5	N_2 bubbling	5
**Capping**	15% (v/v) acetic anhydride in DMF + 1 M pyridine	9	30	N_2 bubbling	1
**Wash**	DMF	9	0.5	N_2 bubbling	3
**Wash**	DCM	9	0.5	N_2 bubbling	3
**Wash**	Diethyl ether	9	0.5	N_2 bubbling	5
**Vacuum Dry**	-	-	30	-	1

Loading test

The manual loading of each amino acid residue was assessed by means of a Fmoc loading test. The resin (~10 mg) was added to 10 mL volumetric flask (x2) and dissolved in 20% (v/v) piperidine in DMF solution (10 mL) and sonicated for 15 minutes. The sample absorption was then monitored by UV at 302 nm against a blank solution of 20% (v/v) piperidine in DMF. The loading was then calculated using a derived equation from the Beer-Lambert law and an average was taken of the two calculated values.

$$L = \frac{A\ x\ 10}{m\ x\ 7.8}$$

where, L = loading; A = absorbance at 302 nm; m = mass of resin used

The loading value determined was used to calculate the required masses for the subsequent amino acid residues for assembly of the desired peptide, which was carried out through manual process (Tables 2–4).

Table 2: Manual addition of second amino acid residue

Step	Solvent/Reagents	Volume (mL)	Time (min)	Mixing	Iterations
**Swell**	DCM	9	20	N_2 bubbling	-
**Wash**	DMF	9	1	N_2 bubbling	3
**Deprotection**	20 % (v/v) piperidine in DMF	9	15	N_2 bubbling	2
**Wash**	DMF	9	1	N_2 bubbling	3
**Coupling**	Amino acid (3 mL), DIC (3 mL), OxymaPure® (3 mL) in DMF	9	240	N_2 bubbling	-
**Wash**	DMF	9	1	N_2 bubbling	1
**Capping**	15 % (v/v) acetic anhydride in DMF	9	15	N_2 bubbling	1
**Wash**	DMF	9	1	N_2 bubbling	3

Table 3: Manual procedure for coupling of third amino acid residue

Step	Solvent/Reagents	Volume (mL)	Time (min)	Mixing	Iterations
**Deprotection**	20 % (v/v) piperidine in DMF	9	15	N_2 bubbling	2
**Wash**	DMF	9	1	N_2 bubbling	1
**Coupling**	Amino acid (3 mL), DIC (3 mL), OxymaPure® (3 mL) in DMF	9	240	N_2 bubbling	-
**Wash**	DMF	9	1	N_2 bubbling	1
**Wash**	DMF	9	1	N_2 bubbling	3
**Capping**	15 % (v/v) acetic anhydride in DMF	9	15	N_2 bubbling	1
**Wash**	DMF	9	1	N_2 bubbling	3
**Wash**	DCM	9	1	N_2 bubbling	3
**Wash**	Diethyl ether	9	1	N_2 bubbling	5
**Vacuum dry**	-	-	30	-	1

Table 4: Manual coupling of fourth amino acid residue

Step	Solvent/Reagents	Volume (mL)	Time (min)	Mixing	Iterations
**Swell**	DCM	9	20	N_2 bubbling	-
**Wash**	DMF	9	1	N_2 bubbling	3
**Deprotection**	20 % (v/v) piperidine in DMF	9	15	N_2 bubbling	2
**Wash**	DMF	9	1	N_2 bubbling	3
**Coupling**	Amino acid (3 mL), DIC (3 mL), OxymaPure® (3 mL) in DMF	9	240	N_2 bubbling	-
**Wash**	DMF	9	1	N_2 bubbling	1
**Capping**	15 % (v/v) acetic anhydride in DMF	9	15	N_2 bubbling	1
**Wash**	DMF	9	1	N_2 bubbling	3
**Deprotection**	20 % (v/v) piperidine in DMF	9	15	N_2 bubbling	2
**Wash**	DMF	9	1	N_2 bubbling	3
**Wash**	DCM	9	1	N_2 bubbling	3
**Wash**	Diethyl ether	9	1	N_2 bubbling	5
**Vacuum dry**	-	-	30	-	1

Table 5: Automated coupling of second amino acid residue

Step	Solvent/Reagents	Volume (mL)	Time (min)	Mixing	Iterations
**Swell (Top wash)**	DCM	6	20	N_2 bubbling and shaking	1
**Wash**	DCM	3	1	N_2 bubbling and shaking	3
**Top Wash**	DMF	6	1	N_2 bubbling and shaking	1
**Wash**	DMF	6	1	N_2 bubbling and shaking	3
**Deprotection**	20% (v/v) piperidine in DMF	6	15	N_2 bubbling and shaking	3
**Top Wash**	DMF	6	1	N_2 bubbling and shaking	1
**Wash**	DMF	6	1	N_2 bubbling and shaking	3
**Pre-Activation**	Amino acid (2 mL), DIC (2 mL)in DMF	4	30	N_2 bubbling and shaking	-
**Pre-Activation**	OxymaPure® (2 mL) in DMF	2	30	N_2 bubbling and shaking	-
**PV to RV**	-	6	-	-	-
**Mix**	Amino acid (2 mL), DIC (2 mL), OxymaPure® (2 mL) in DMF	6	240	N_2 bubbling and shaking	1
**Top Wash**	DMF	6	1	N_2 bubbling and shaking	1
**Wash**	DMF	6	2	N_2 bubbling and shaking	3
**Capping**	15% (v/v) piperidine in DMF	6	30	N_2 bubbling and shaking	1
**Top Wash**	DMF	6	1	N_2 bubbling and shaking	1
**Wash**	DMF	6	1	N_2 bubbling and shaking	3

Table 6: Automated coupling of amino acid residues

Step	Solvent/Reagents	Volume (mL)	Time (min)	Mixing	Iterations
**Deprotection**	20% (v/v) piperidine in DMF	6	15	N_2 bubbling and shaking	3
**Top Wash**	DMF	6	1	N_2 bubbling and shaking	1
**Wash**	DMF	6	1	N_2 bubbling and shaking	3
**Pre-Activation**	Amino acid (2 mL), DIC (2 mL)in DMF	4	30	N_2 bubbling and shaking	-
**Pre-Activation**	OxymaPure® (2 mL) in DMF	2	30	N_2 bubbling and shaking	-
**PV to RV**	-	6	-	-	-
**Mix**	Amino acid (2 mL), DIC (2 mL), OxymaPure® (2 mL) in DMF	6	240	N_2 bubbling and shaking	1
**Top Wash**	DMF	6	1	N_2 bubbling and shaking	1
**Wash**	DMF	6	2	N_2 bubbling and shaking	3
**Capping**	15% (v/v) piperidine in DMF	6	30	N_2 bubbling and shaking	1
**Top Wash**	DMF	6	1	N_2 bubbling and shaking	1
**Wash**	DMF	6	1	N_2 bubbling and shaking	3

Following from the SPPS, a trial cleave is conducted for each peptide in order to assess if the synthesis had been successful. 10 mg of each resin was added to 250 μL of the cleaving solution TFA/H_2O/TIS (95:2.5:2.5 v/v) or TFA/H_2O/phenol/thioanisole/DODT (82.5/5/5/5/2.5 v/v) for sulfur-containing peptides. The solution containing the resin was stirred at room temperature for 1 hour and then decanted into cold Et_2O in order to precipitate the peptide. The suspension was then centrifuged for 3 minutes, and the liquid was discarded, and this was repeated 3 times. The solid peptide remaining with then dissolved in a MeCN/H_2O solvent mixture to by analysed by HPLC and LCMS.

Capping of Peptides with Active Esters

A solution of tetrazole (1 equiv.) in toluene was heated at 110 °C for 2 hours and concentrated *in vacuo*. The resultant benzoate was then dissolved in minimal amounts of DMF before addition to the resin.

Table 7: Manual capping with 1*H*-Benzo[*d*][1,2,3]triazol-1-yl benzoate

Step	Solvent/Reagents	Volume (mL)	Time (min)	Mixing	Iterations
**Swell**	DCM	3	20	-	-
**Wash**	DMF	3	1	N_2 bubbling	3
**Deprotection**	20% (v/v) piperidine in DMF	3	15	N_2 bubbling	2
**Wash**	DMF	3	1	N_2 bubbling	3
**Coupling**	Active ester (1 eq.), DMF	3	240	N_2 bubbling	-
**Wash**	DMF	3	1	N_2 bubbling	3
**Wash**	DCM	3	1	N_2 bubbling	3
**Wash**	Diethyl ether	3	1	N_2 bubbling	5
**Vacuum Dry**	-	-	30	-	-

A trial cleave was then conducted as previously described. The crude peptide was analysed by HPLC with a gradient of 5-95% MeCN/H_2O, both spiked with 0.1% TFA, over 31 minutes at a flow rate of 1 mL/min.

Benzoyl-Leu-Enkephalin (8a)

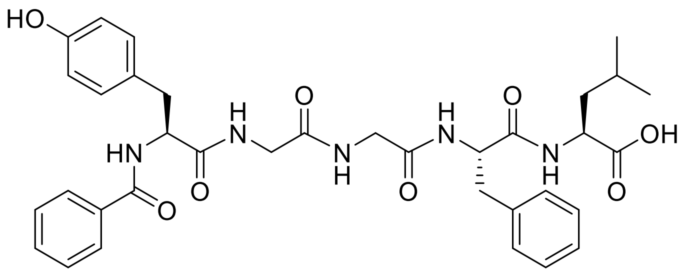

Using resin loading value 0.50 mmol/g, Leu-loaded Wang resin (100 mg, 0.05 mmol) was added to a Merrifield bubbler and the title peptide was synthesised according to Tables 1-4. The peptide was then capped with 1*H*-Benzo[*d*][1,2,3]triazol-1-yl benzoate (**2a**) according to Table 7 from 2-(2,4-dinitrophenyl)-5-phenyl-2*H*-tetrazole (**1a**). Cleavage with TFA/TIS/H_2O (95/2.5/2.5 v/v). HRMS (ESI) m/z: $[M + Na]^+$ calcd for $C_{35}H_{41}N_5O_8Na$ 682.2853; found 682.2850.

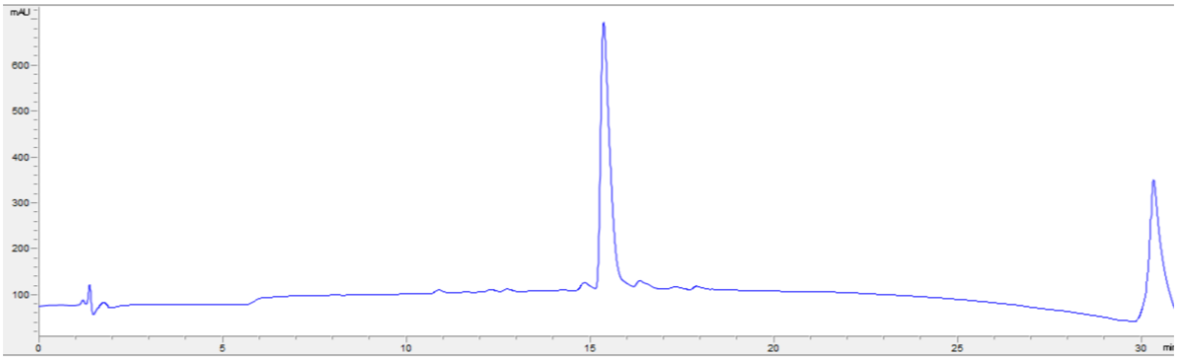

Crude purity 91% at 15.351 minutes

(4-Methoxy-d_3)benzoyl-2,6-d_2-Leu-Enkephalin (8b) and (4-methoxy)benzoyl-Leu-Enkephalin (8c)

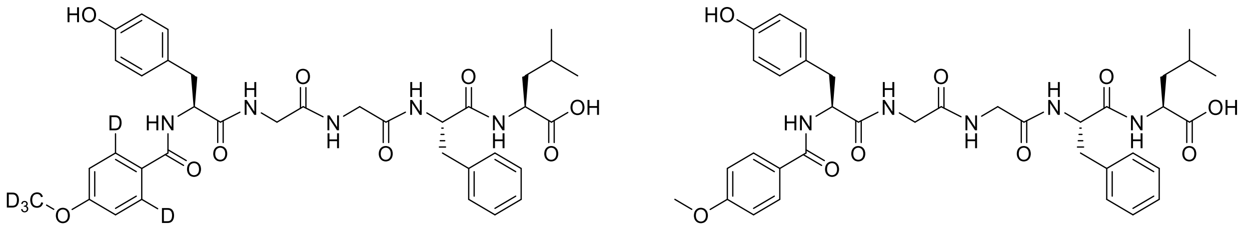

Using resin loading value 0.50 mmol/g, Leu-loaded Wang resin (100 mg, 0.05 mmol) was added to a Merrifield bubbler and the title peptide was synthesised according to Tables 1-4. The peptide was then capped with 6-nitro-1*H*-benzo[*d*][1,2,3]triazol-1-yl-4-*d* 4-methoxy-d_3) benzoate-2,6-d_2 according to Table 7 from 2-(2,4-dinitrophenyl-6-*d*)-5-(4-methoxy-d_3)phenyl-2,6-d_2-2*H*-tetrazole (**1r**), in a 1:1 mixture with 2-(2,4-dinitrophenyl)-5-(4-methoxy)phenyl-2*H*-tetrazole (**1b**). Cleavage with TFA/TIS/H_2O (95/2.5/2.5 v/v). HRMS (ESI) m/z: $[M + Na]^+$ calcd for $C_{36}H_{38}D_5N_5O_9Na$ 717.3272; found 717.3248, $[M + Na]^+$ calcd for $C_{36}H_{43}N_5O_9Na$ 712.2958; found 712.2945.

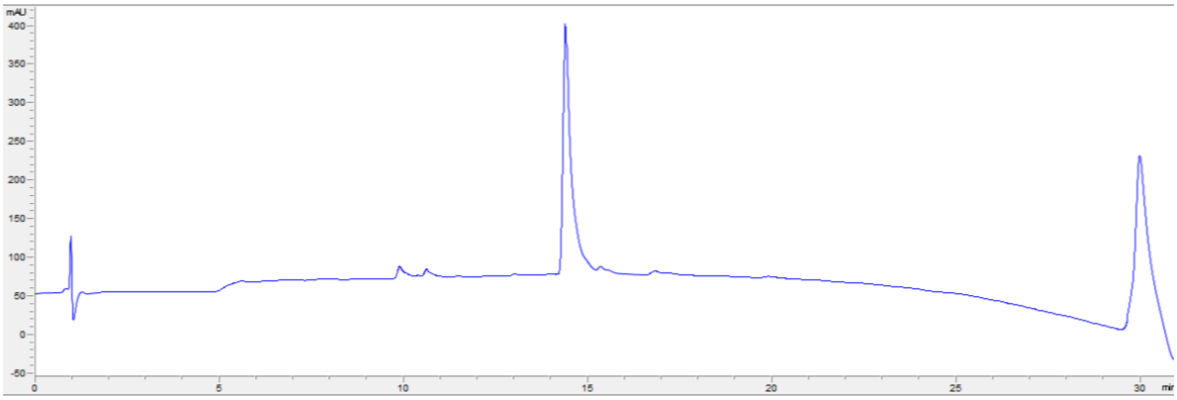

Crude purity 92% at 14.389 minutes

Benzoyl-Substance P (9)

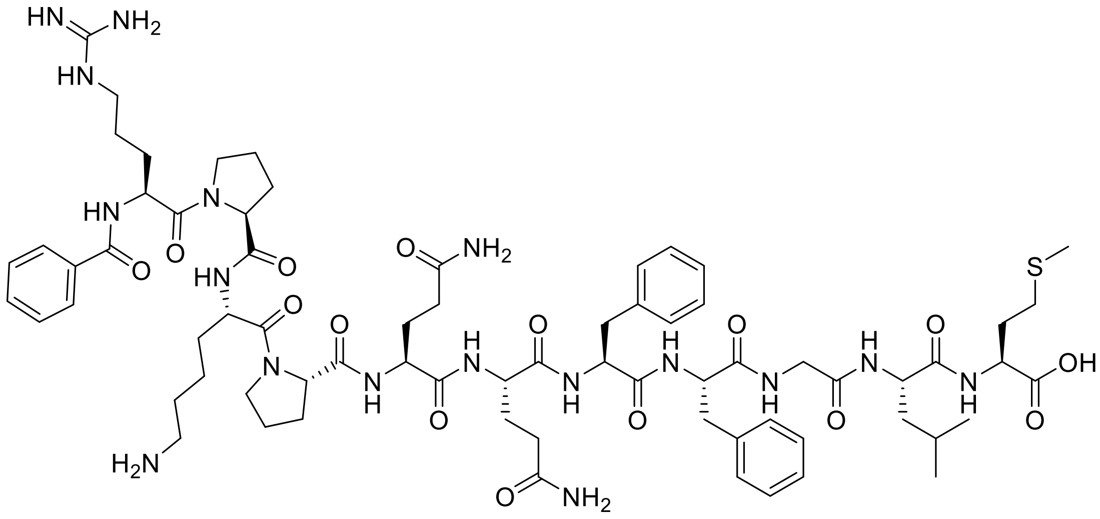

Using resin loading value 0.42 mmol/g, Met-loaded Wang resin (476 mg, 0.2 mmol) the title peptide was synthesised according to Tables 1, 5, and 6. The peptide was then capped with 1*H*-Benzo[*d*][1,2,3]triazol-1-yl benzoate (**2a**) according to Table 7 from 2-(2,4-dinitrophenyl)-5-phenyl-2*H*-tetrazole (**1a**). Cleavage with TFA/H_2O/phenol/thioanisole/DODT (82.5/5/5/5/2.5 v/v). HRMS (ESI) m/z: $[M + H]^+$ calcd for $C_{70}H_{102}N_{17}O_{15}S$ 1452.7462; found 1452.7444.

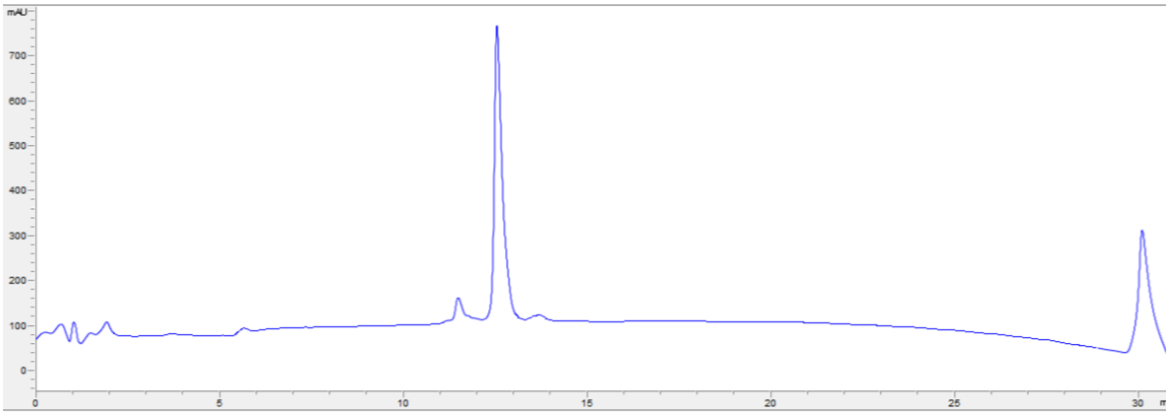

Crude purity 95% at 12.324 minutes.

4. References

[1] J. R. Maxwell, D. A. Wasdahl, A. C. Wolfson, V. I. Stenberg, *J. Med. Chem.* **1984**, *27*, 1565–1570.

[2] N. Iqbal, J. Hashim, S. A. Ali, M. al-Rashida, R. D. Alharthy, S. Ahmad, K. M. Khan, F. Z. Basha, S. T. Moin, A. Hameed, *RSC Adv.* **2015**, *5*, 95061–95072.

[3] M. R. Bhosle, D. S. Shaikh, L. D. Khillare, A. R. Deshmukh, R. A. Mane, *Synth. Commun.* **2017**, *47*, 695–703.

[4] E. Kobayashi, H. Togo, *Tetrahedron* **2018**, *74*, 4226–4235.

[5] S. J. Wittenberger, B. G. Donner, *J. Org. Chem.* **1993**, *58*, 4139–4141.

[6] A. Vijeta, C. Casadevall, S. Roy, E. Reisner, **2021**, 8494–8499.

[7] W. J. Kerr, D. M. Lindsay, P. K. Owens, M. Reid, T. Tuttle, S. Campos, *ACS Catal.* **2017**, *7*, 7182–7186.

[8] C. A. Wilhelmsen, A. D. C. Dixon, J. D. Chisholm, D. A. Clark, *J. Org. Chem.* **2018**, *83*, 1634–1642.

[9] B. R. Kim, H. G. Lee, S. B. Kang, G. H. Sung, J. J. Kim, J. K. Park, S. G. Lee, Y. J. Yoon, *Synthesis (Stuttg).* **2012**, *44*, 42–50.

[10] S. Cheruku, S. C. Nagarakere, M. P. Sunilkumar, Y. Narayana, K. N. Manikyanally, K. S. Rangappa, K. Mantelingu, *New J. Chem.* **2022**, *46*, 4421–4426.

[11] A. (Gus) Bakhoda, Q. Jiang, Y. M. Badiei, J. A. Bertke, T. R. Cundari, T. H. Warren, *Angew. Chemie - Int. Ed.* **2019**, *58*, 3421–3425.

[12] B. Zoller, J. Zapp, P. H. Huy, *Chem. - A Eur. J.* **2020**, *26*, 9632–9638.

[13] K. Singha, S. C. Ghosh, A. B. Panda, *European J. Org. Chem.* **2021**, 657–662.

[14] J. Iley, R. Tolando, *J. Chem. Soc. Perkin Trans. 2* **2000**, 2328–2336.

[15] T. Kaicharla, M. Thangaraj, A. T. Biju, *Org. Lett.* **2014**, *16*, 1728–1731.

[16] S.-Y. Lu, S. S. Badsara, Y.-C. Wu, D. M. Reddy, C.-F. Lee, *Tetrahedron Lett.* **2016**, *57*, 633–636.

[17] A. Drageset, H.-R. Bjørsvik, *European J. Org. Chem.* **2018**, 4436–4445.

[18] T.-J. Zhang, S.-Y. Li, W. Y. Yuan, Y. Zhang, F.-H. Meng, *Chem. Biol. Drug Des.* **2018**, *91*, 893–901.

[19] B. Xu, Q. Jiang, A. Zhao, J. Jia, Q. Liu, W. Luo, C. Guo, *Chem. Commun.* **2015**, *51*, 11264–11267.

[20] J. Wang, H. Hou, Y. Hu, J. Lin, M. Wu, Z. Zheng, X. Xu, *Tetrahedron Lett.* **2021**, *65*, 152801.

[21] G. Li, M. Szostak, *Org. Biomol. Chem.* **2020**, *18*, 3827–3831.

[22] C. G. McPherson, N. Caldwell, C. Jamieson, I. Simpson, A. J. B. Watson, *Org. Biomol. Chem.* **2017**, *15*, 3507–3518.

[23] Y.-J. Wang, G.-Y. Zhang, A. Shoberu, J.-P. Zou, *Tetrahedron Lett.* **2021**, *80*, 153316.

[24] J. Zhu, Y. Zhang, F. Shi, Y. Deng, *Tetrahedron Lett.* **2012**, *53*, 3178–3180.

[25] J. Liu, Q. Liu, H. Yi, C. Qin, R. Bai, X. Qi, Y. Lan, A. Lei, *Angew. Chemie - Int. Ed.* **2014**, *53*, 502–506.

[26] M. Boyle, K. Livingstone, M. C. Henry, J. M. L. Elwood, J. D. Lopez-Fernandez, C. Jamieson, *Org. Lett.* **2022**, *24*, 334–338.

[27] R. A. Green, D. Pletcher, S. G. Leach, R. C. D. Brown, *Org. Lett.* **2016**, *18*, 1198–1201.

[28] S. S. Kulkarni, X. Hu, R. Manetsch, *Chem. Commun.* **2013**, *49*, 1193–1195.

[29] M. M. Hamed, A. M. Abdalla, M. A. Ghareeb, S. A. Saleh, *Int. J. Pharm. Pharm. Sci.* **2017**, *9*, 240–247.

[30] B. Tan, N. Toda, C. F. Barbas, *Angew. Chemie - Int. Ed.* **2012**, *51*, 12538–12541.

[31] A. Wang, Y. Xie, J. Wang, D. Shi, H. Yu, *Chem. Commun.* **2022**, *58*, 1127–1130.

[32] C. Feng, G. Yin, B. Yan, J. Chen, M. Ji, *J. Chem. Res.* **2018**, *42*, 383–386.

[33] C. Wan, Y. Feng, Z. Hou, C. Lian, L. Zhang, Y. An, J. Sun, D. Yang, C. Jiang, F. Yin, R. Wang, Z. Li, *Org. Lett.* **2022**, *24*, 581–586.

[34] Y. H. Wang, J. L. Ye, A. E. Wang, P. Q. Huang, *Org. Biomol. Chem.* **2012**, *10*, 6504–6511.

[35] A. Kumar, N. A. Espinosa-Jalapa, G. Leitus, Y. Diskin-Posner, L. Avram, D. Milstein, *Angew. Chemie - Int. Ed.* **2017**, *56*, 14992–14996.

[36] S. D. Nielsen, G. Smith, M. Begtrup, J. L. Kristensen, *Chem. - A Eur. J.* **2010**, *16*, 4557–4566.

[37] Z. Fu, X. Wang, S. Tao, Q. Bu, D. Wei, N. Liu, *J. Org. Chem.* **2021**, *86*, 2339–2358.

[38] M.-C. Cheng, W.-C. Lo, Y.-W. Chang, S.-S. Lee, C.-C. Chang, *Bioorg. Chem.* **2020**, *104*, 104166.

5. ^{1}H and ^{13}C NMR Spectra

^{1}H NMR: 500 MHz,
D_6-DMSO

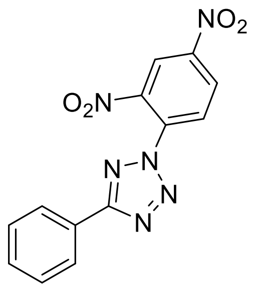

1a

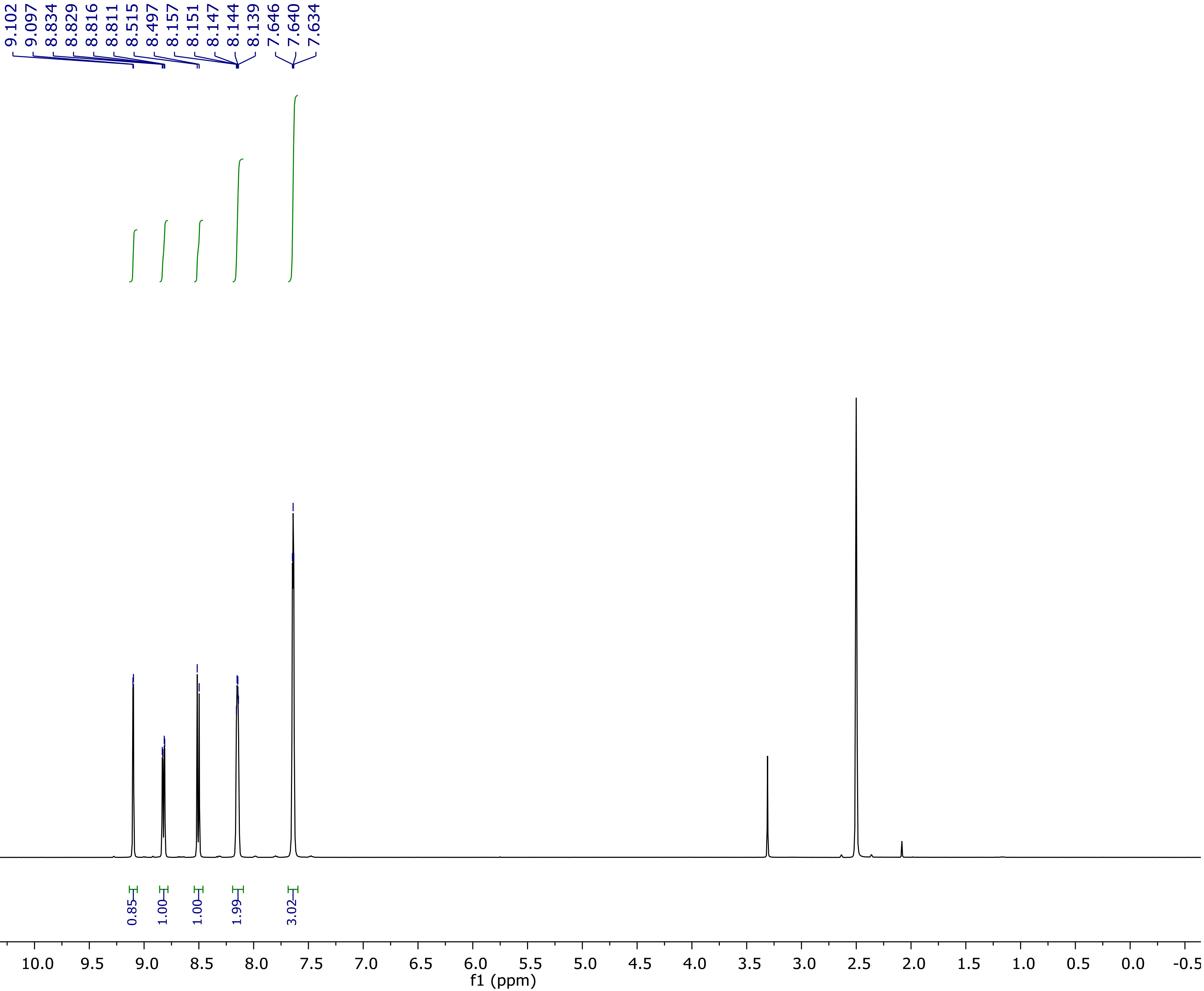

^{13}C NMR: 126 MHz, D_6-DMSO

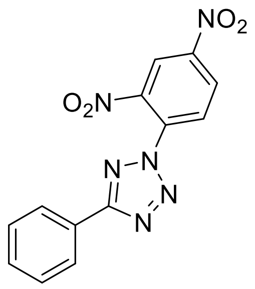

1a

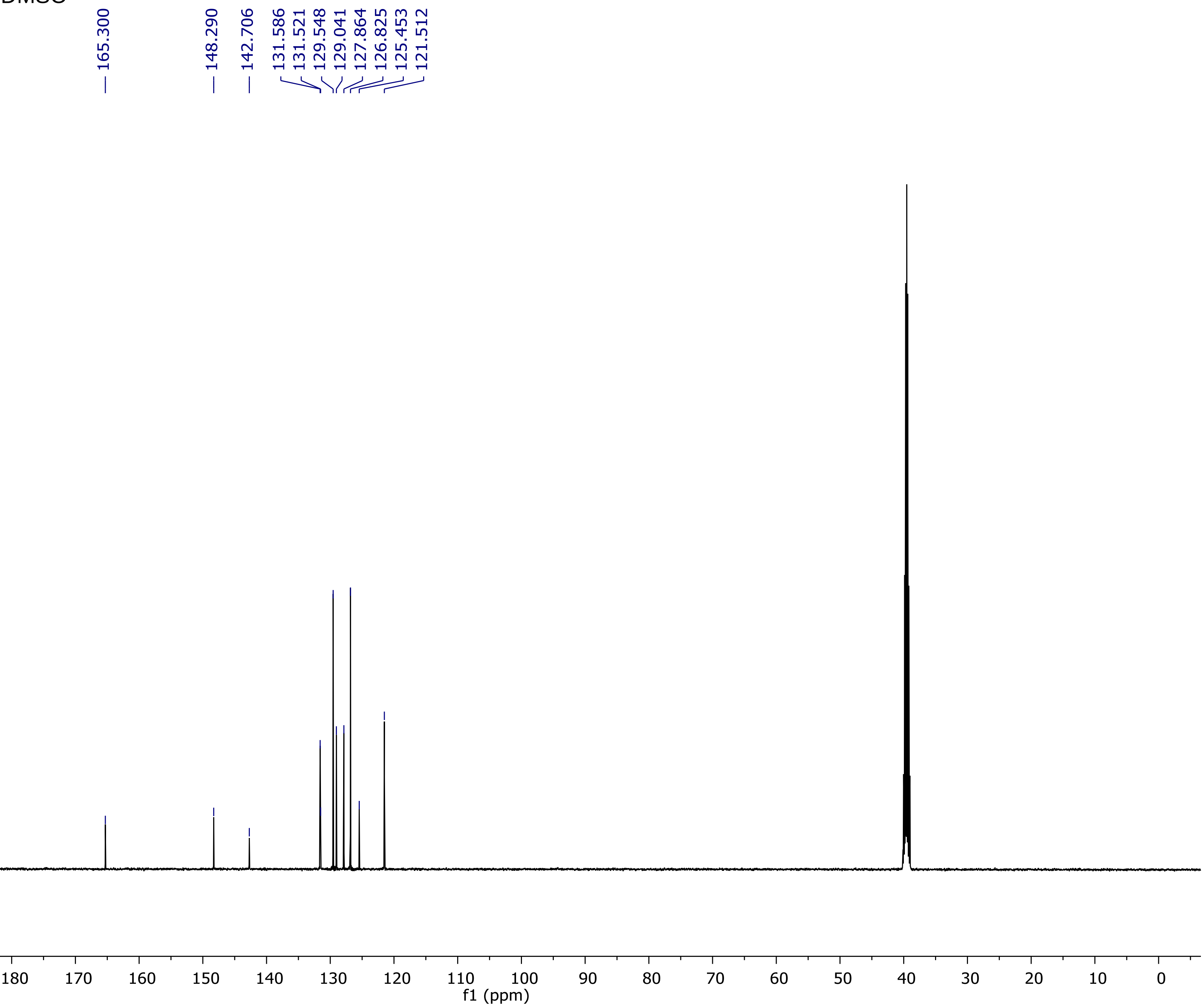

^{1}H NMR: 400 MHz, $CDCl_3$

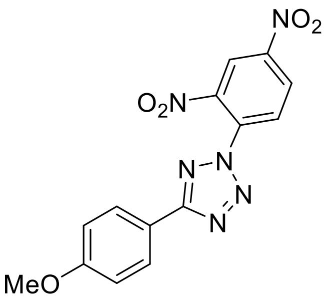

1b

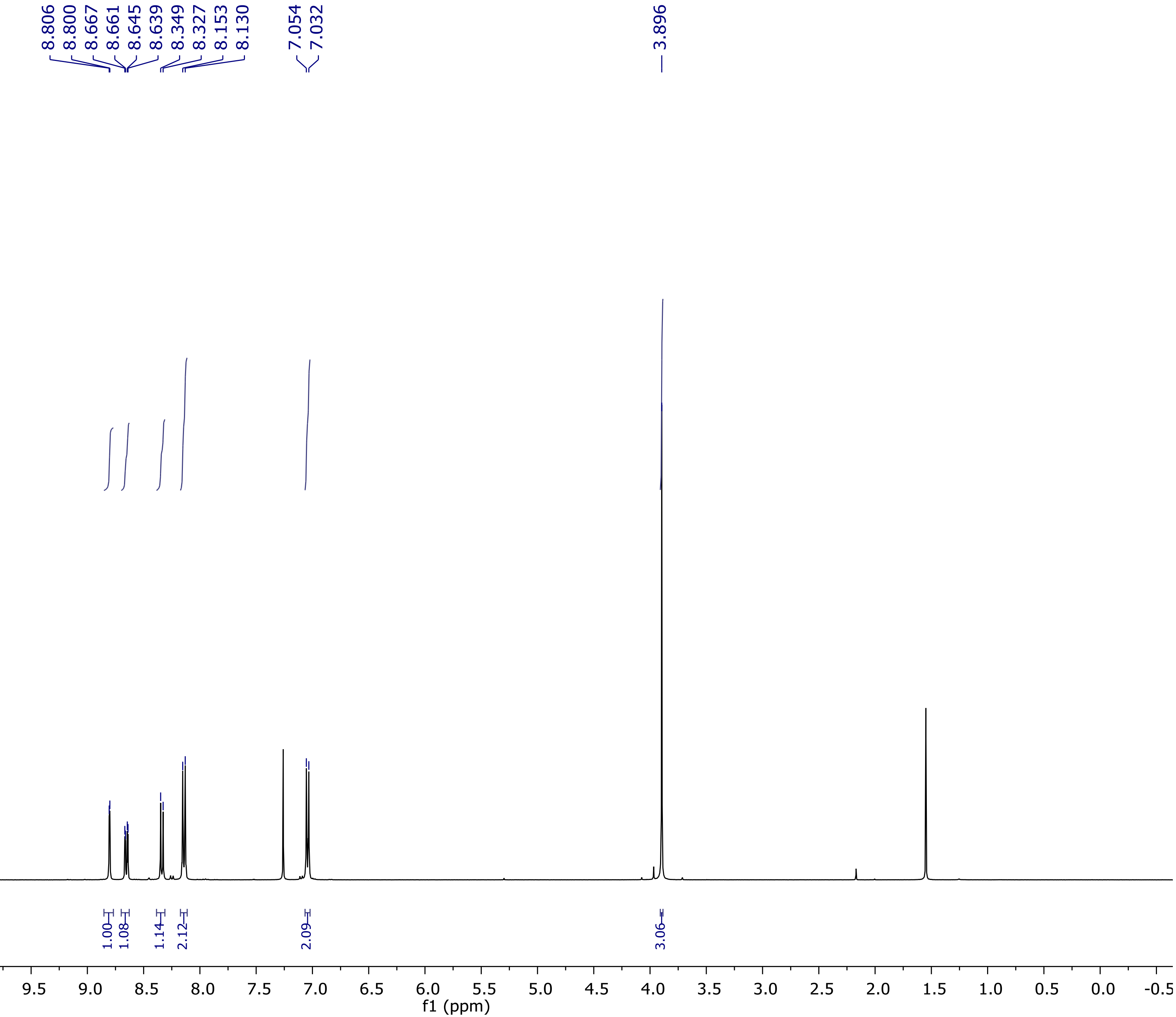

^{13}C NMR: 101 MHz, $CDCl_3$

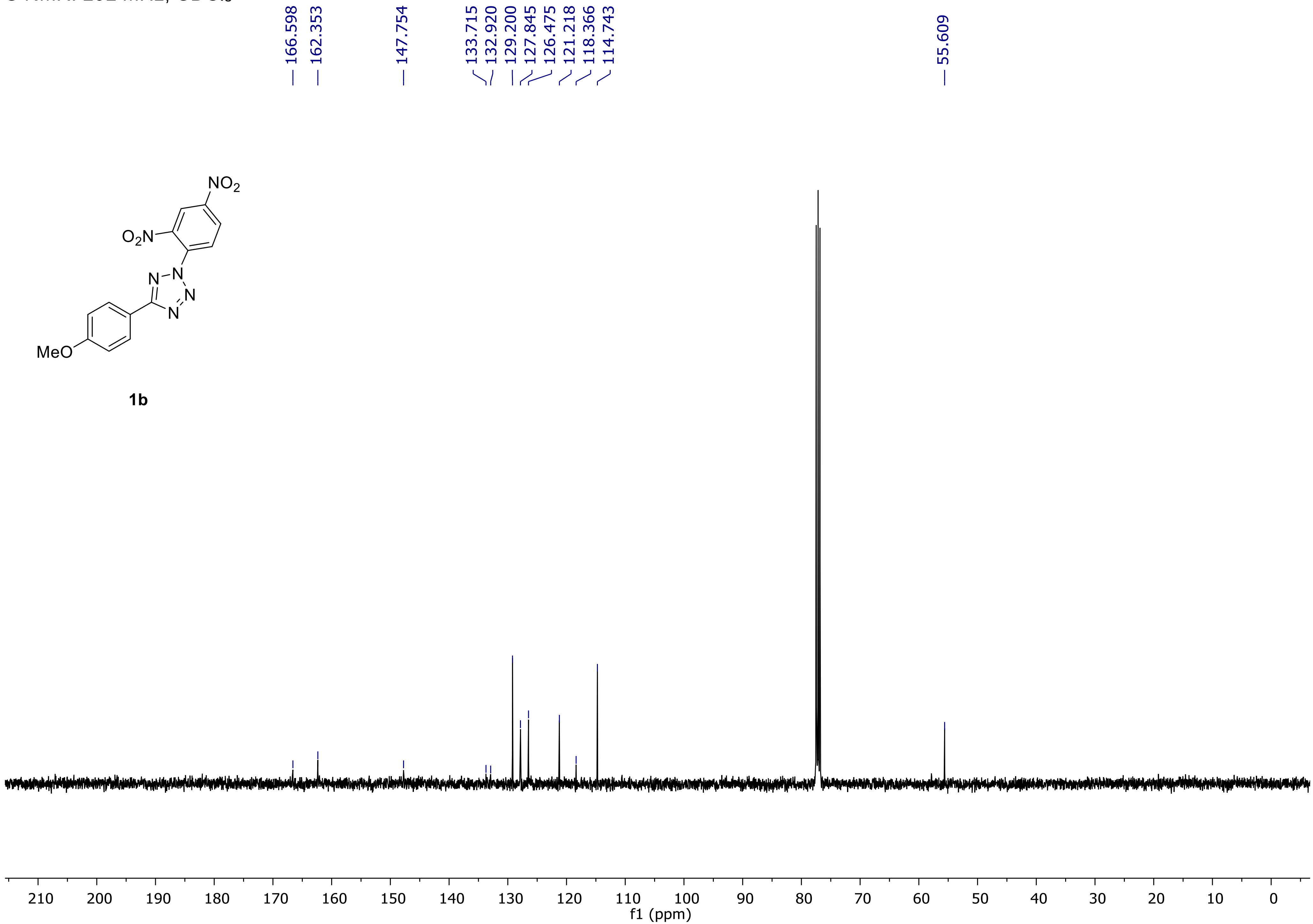

^1H NMR: 400 MHz, $CDCl_3$

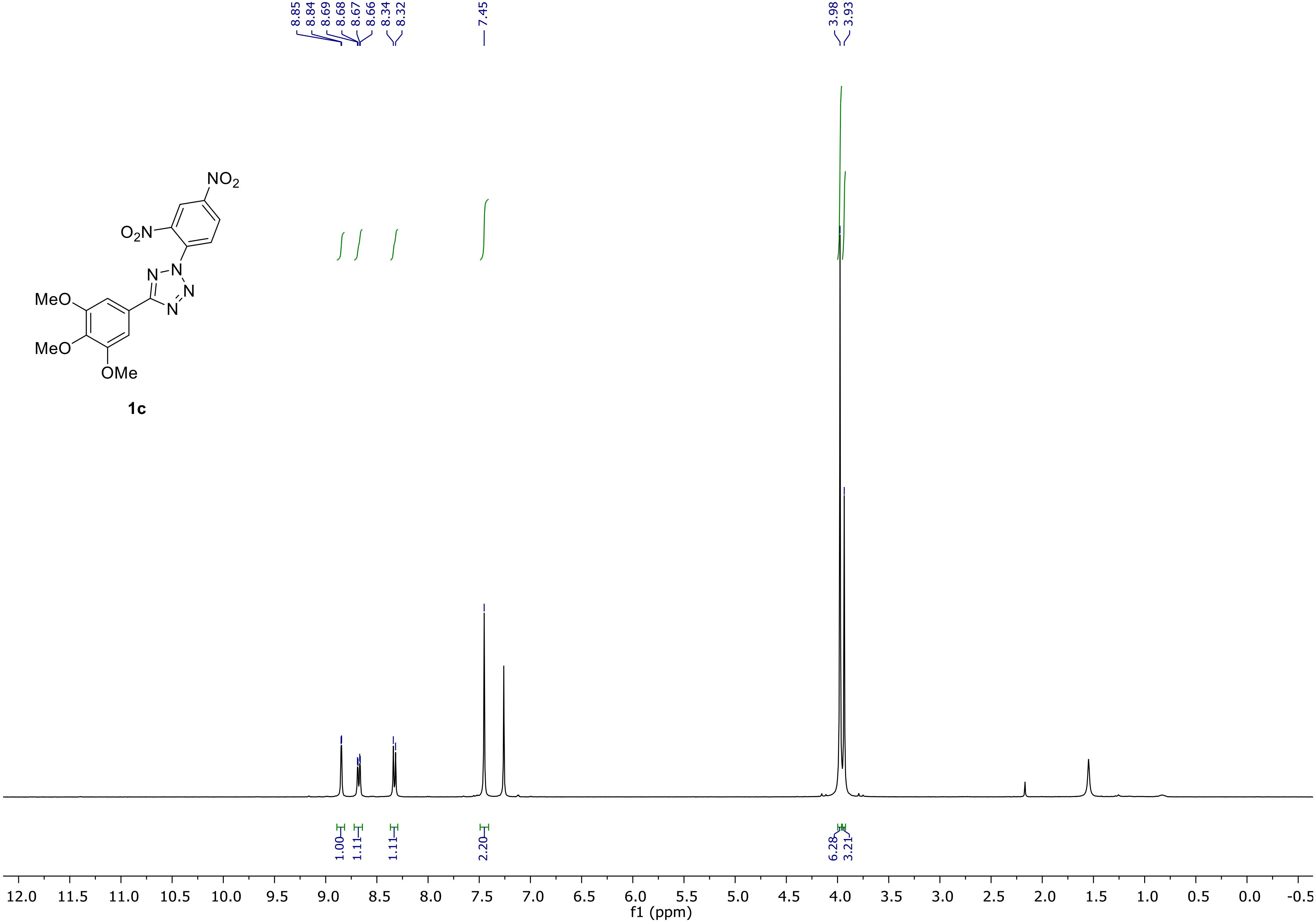

1c

^{13}C NMR: 126 MHz, $CDCl_3$

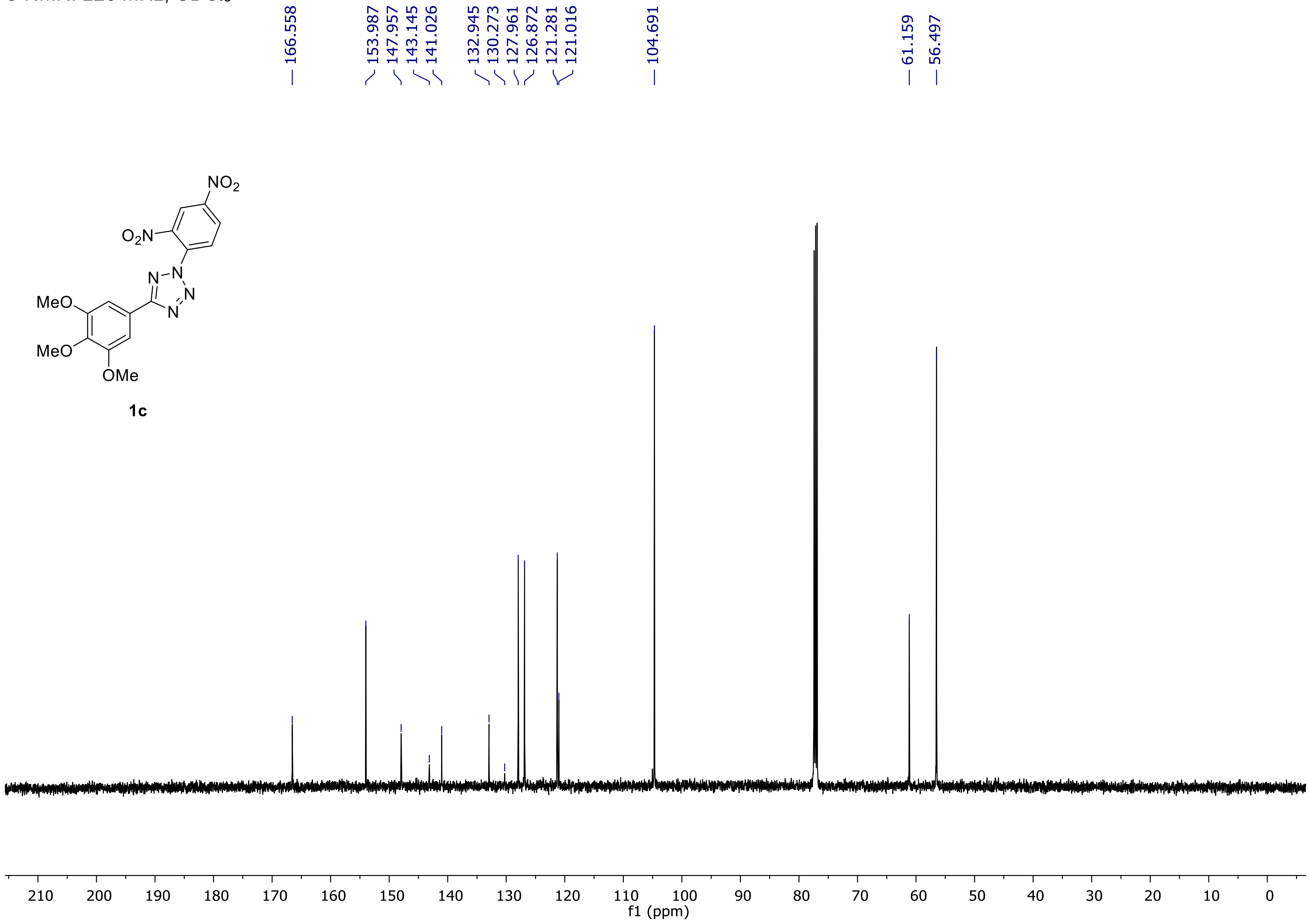

^{1}H NMR: 400 MHz, $CDCl_3$

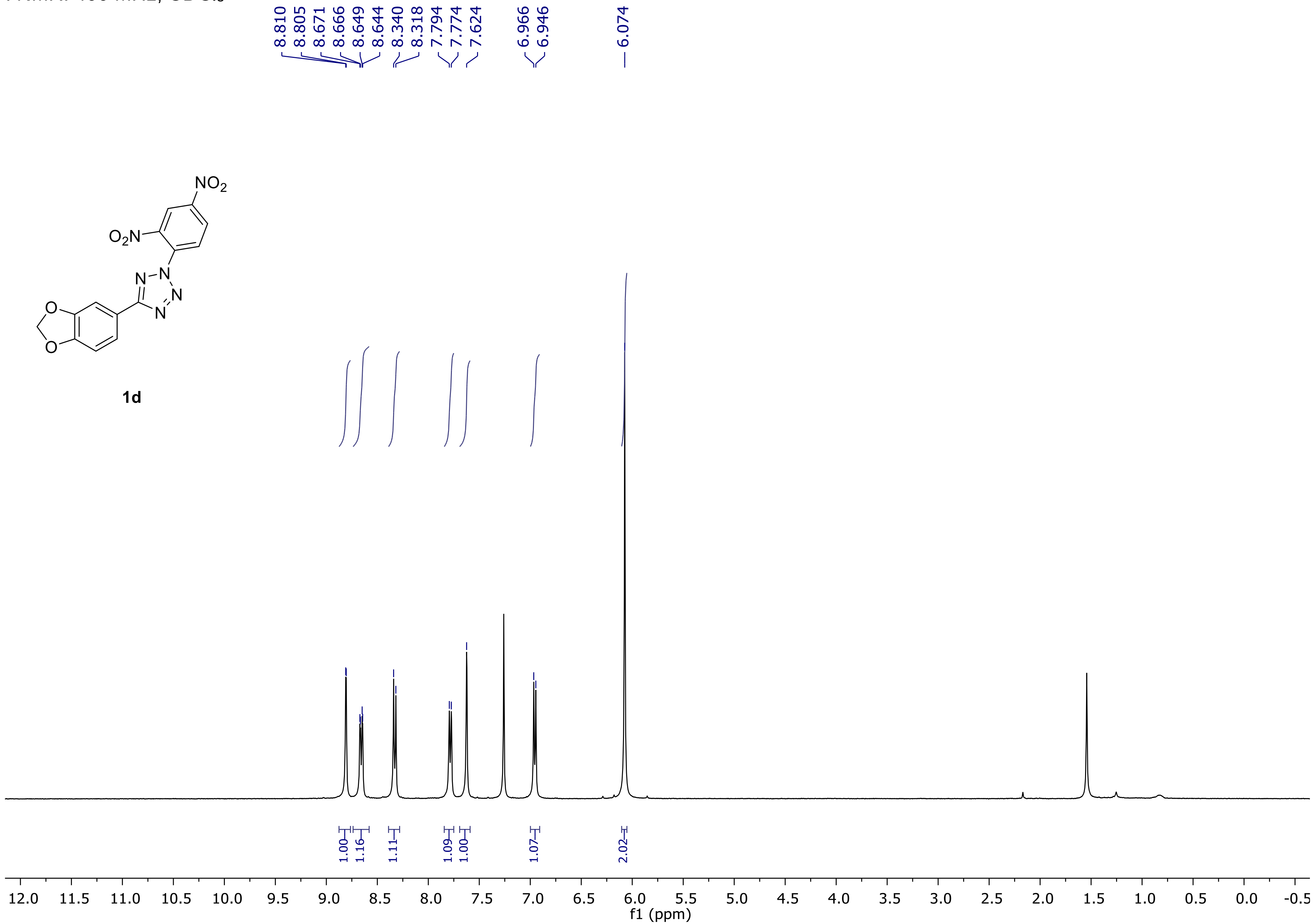

^{13}C NMR: 126 MHz, $CDCl_3$

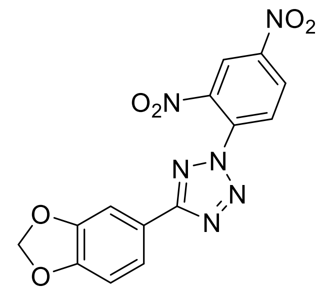

1d

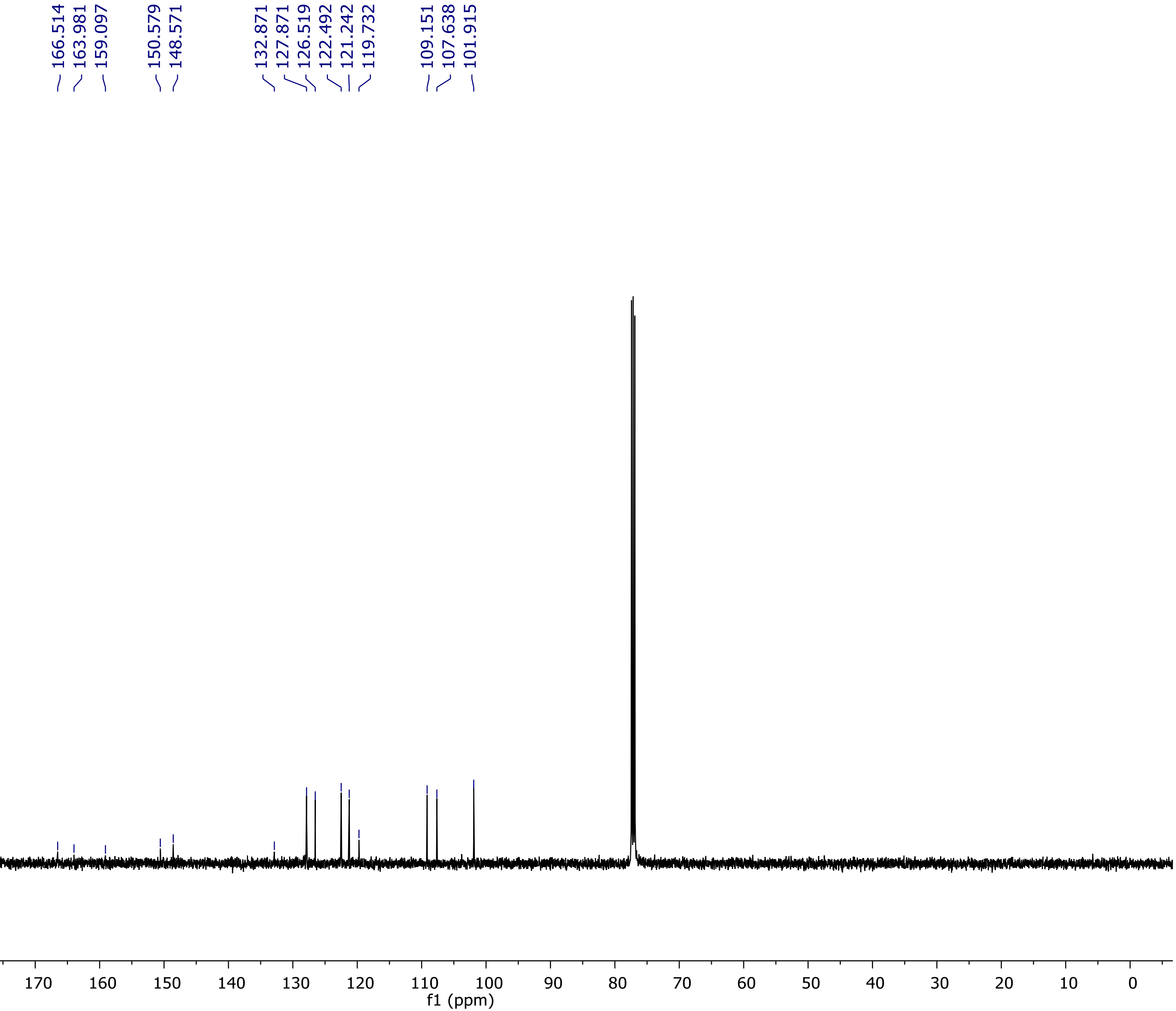

^1H NMR: 400 MHz, $CDCl_3$

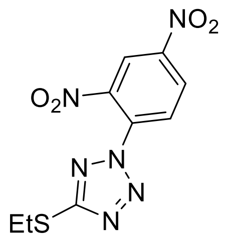

1e

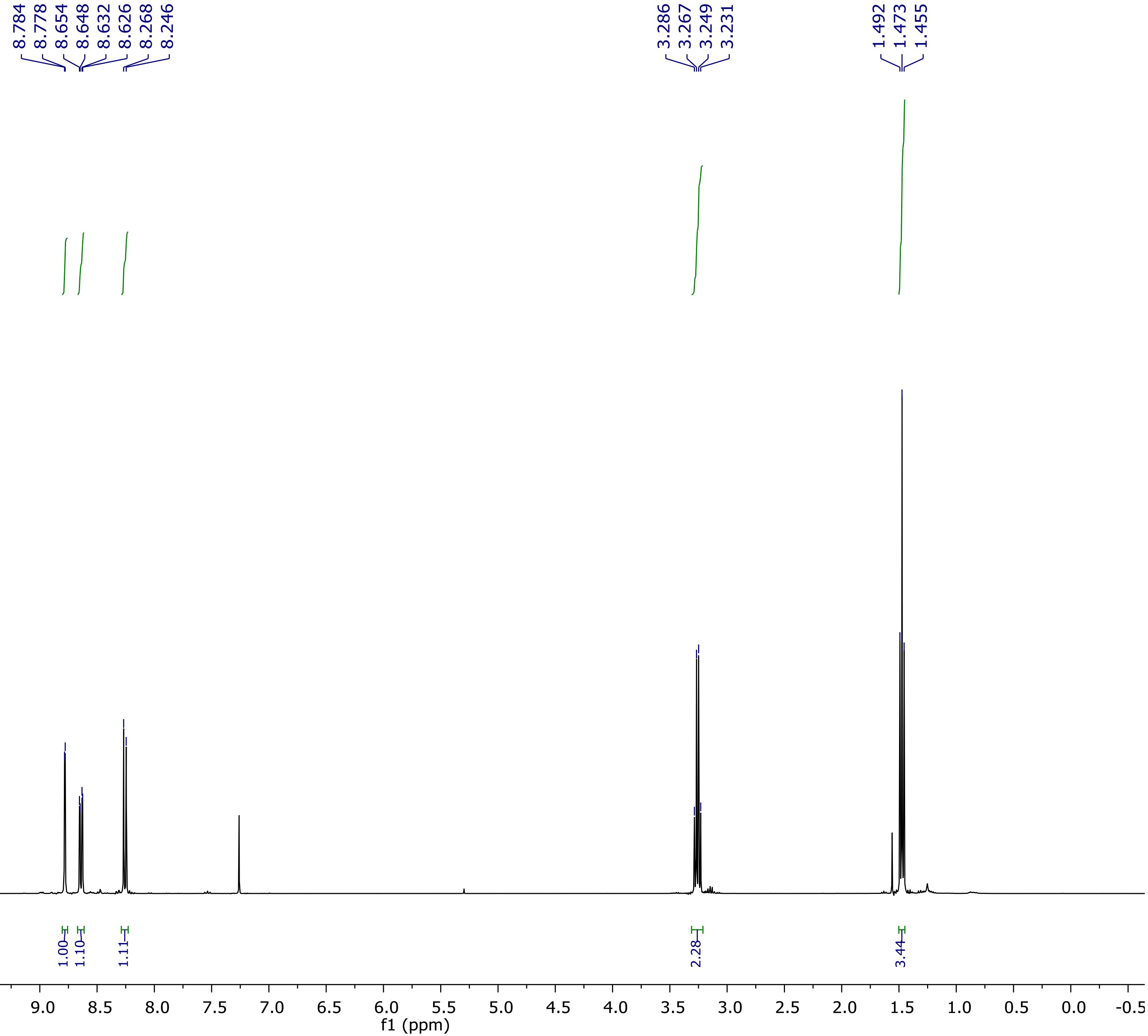

^{13}C NMR: 101 MHz, $CDCl_3$

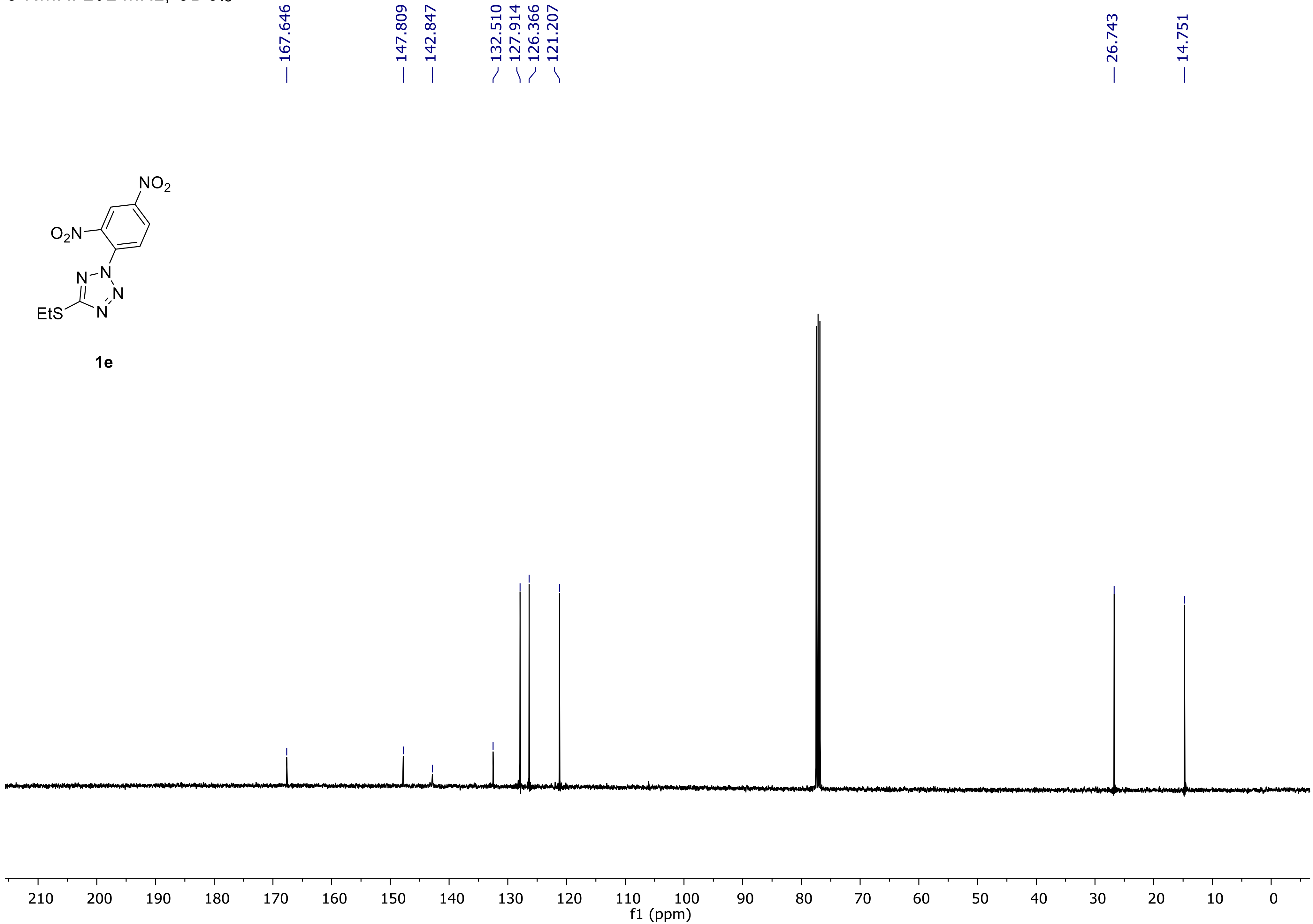

1e

^{1}H NMR: 400 MHz, $CDCl_3$

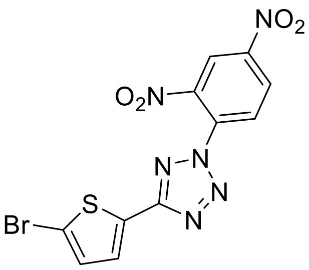

1f

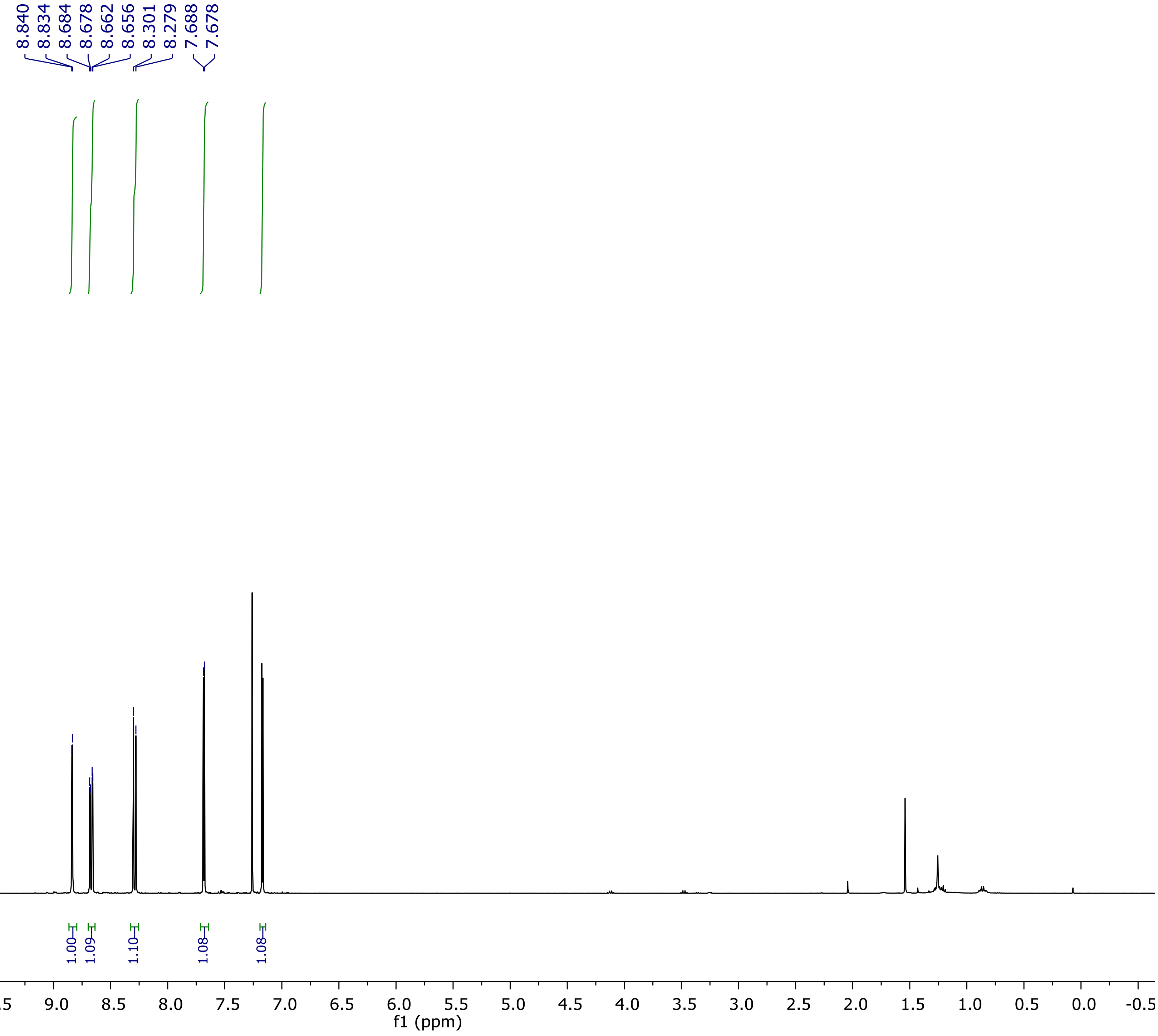

^{13}C NMR: 101 MHz, $CDCl_3$

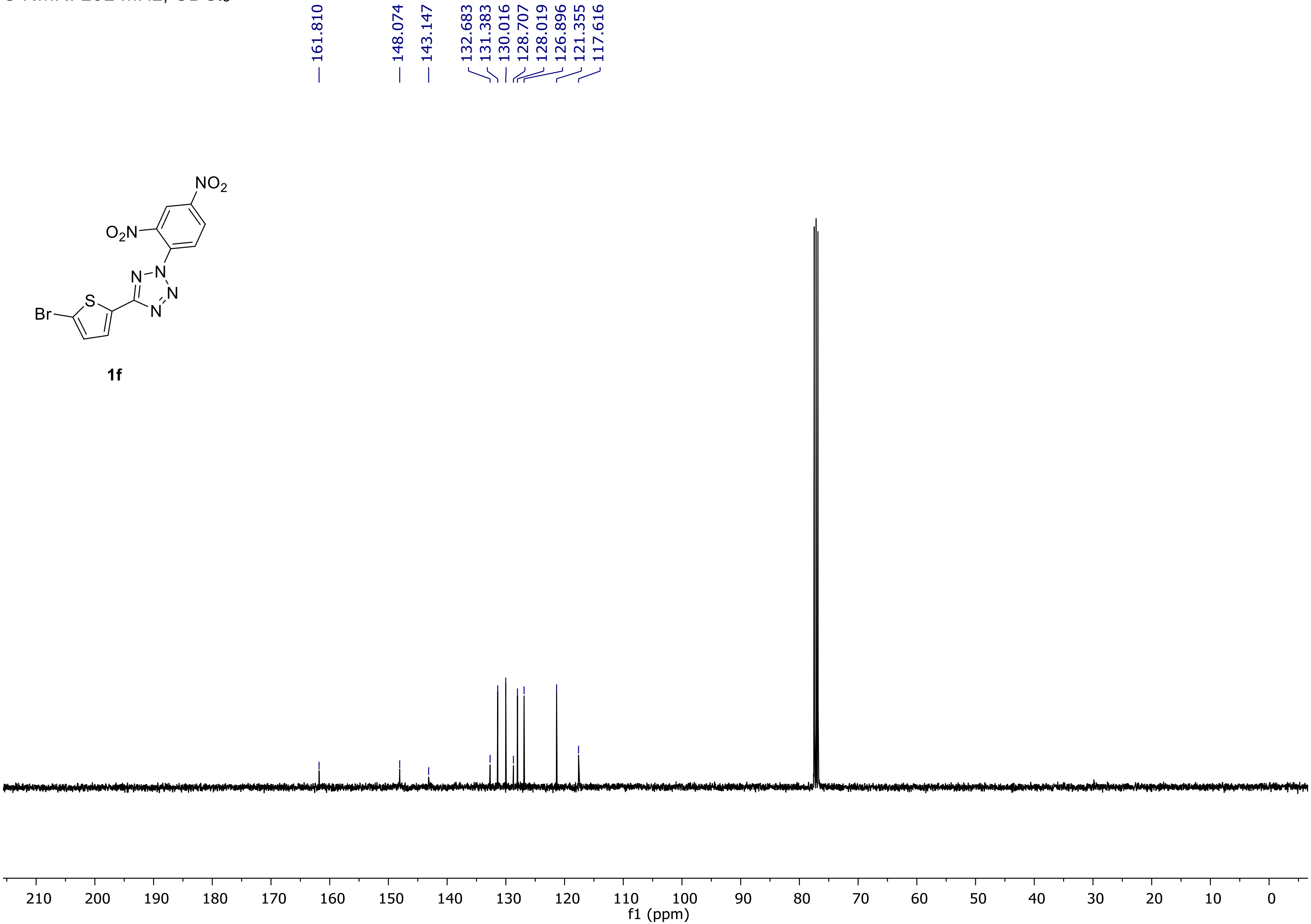

^{1}H NMR: 400 MHz,
D_6-DMSO

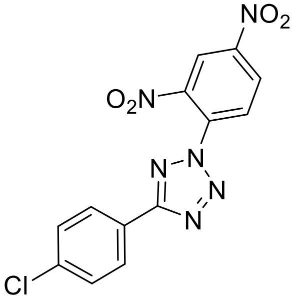

1g

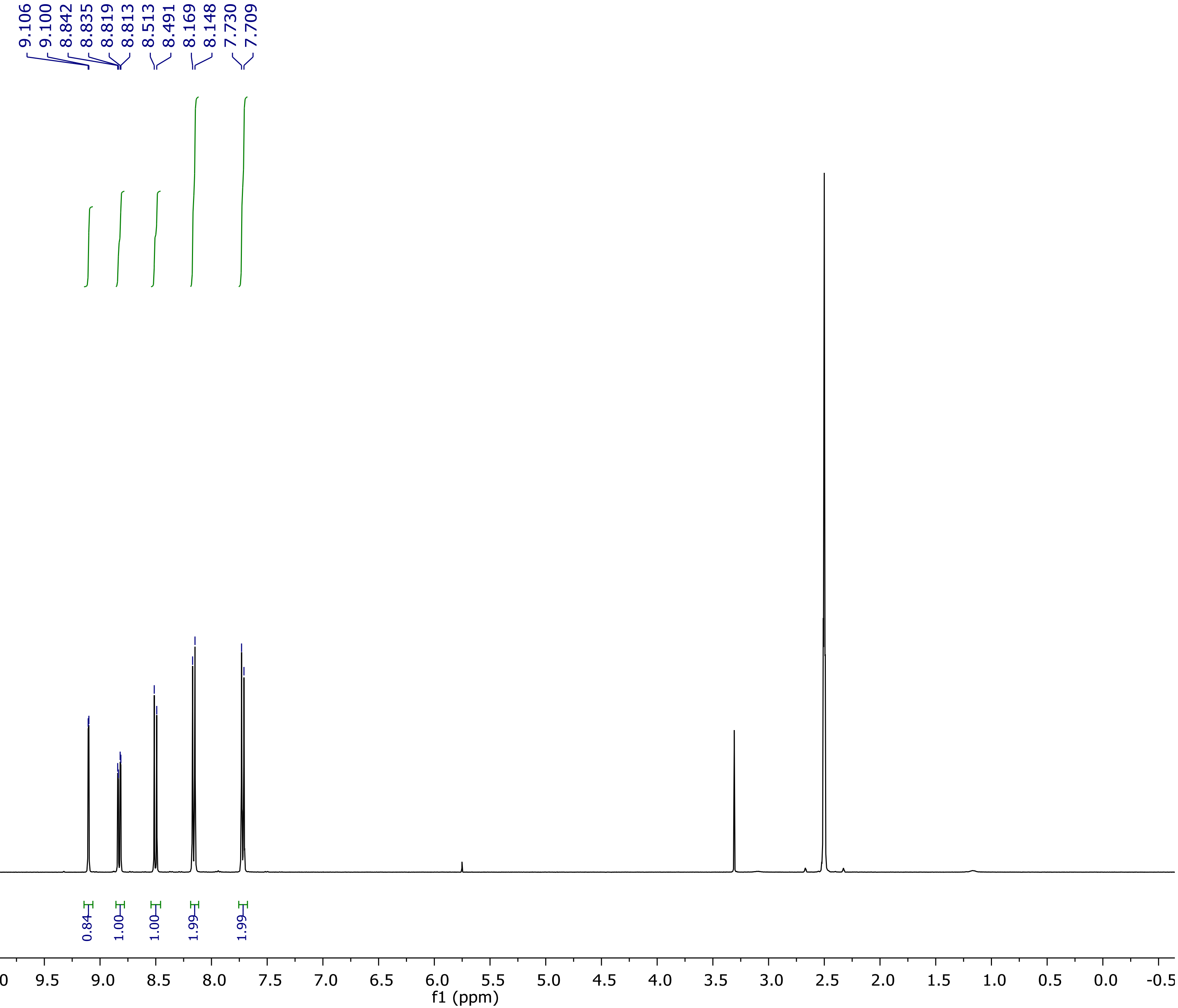

^{13}C NMR: 101 MHz, D_6-DMSO

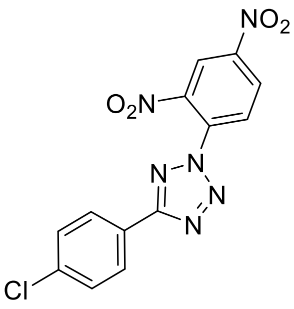

1g

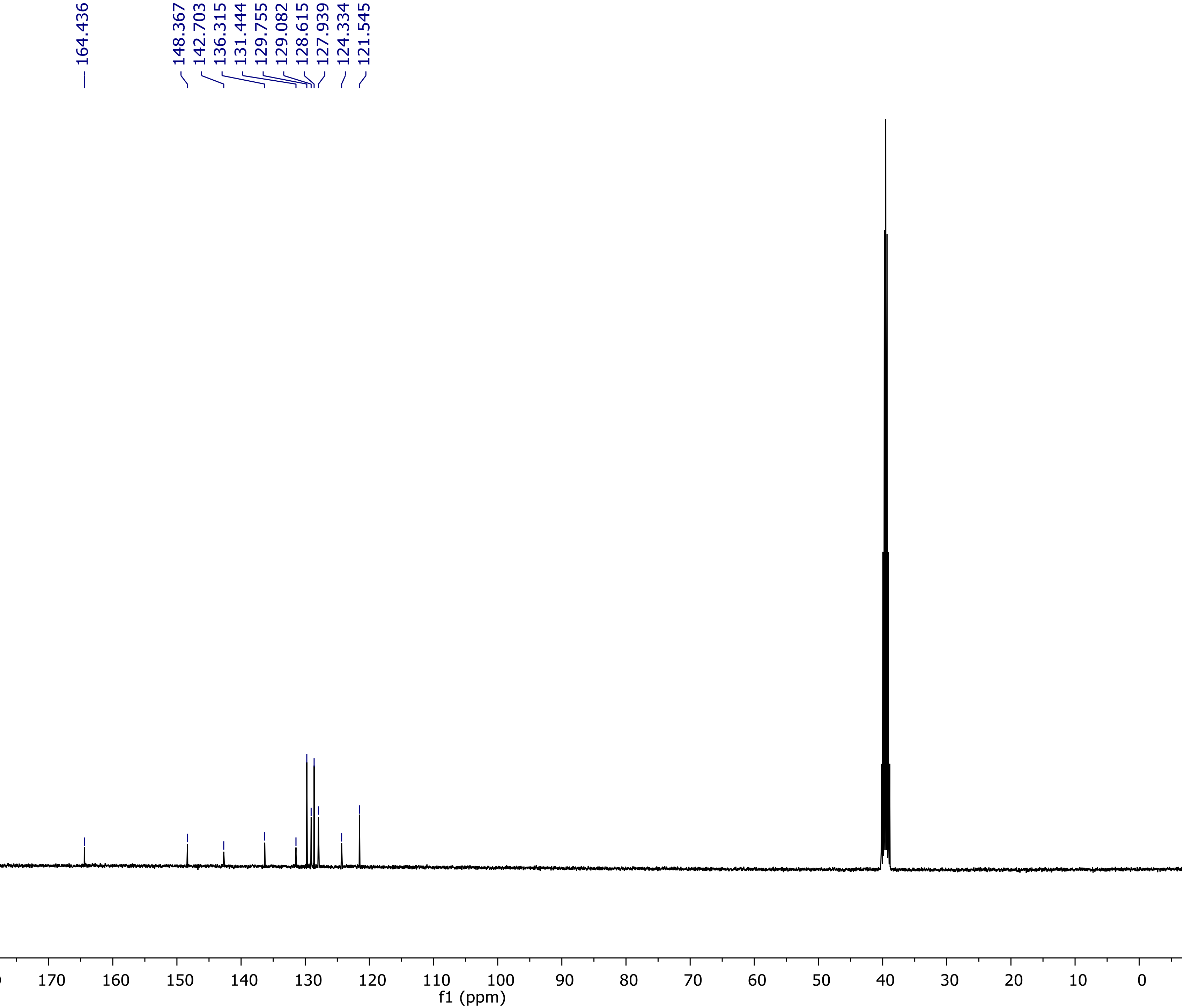

^{1}H NMR: 400 MHz, $CDCl_3$

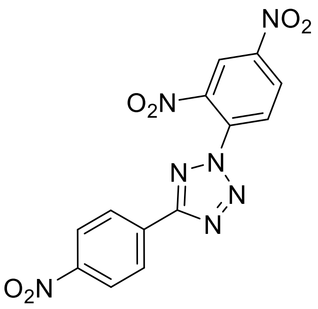

1h

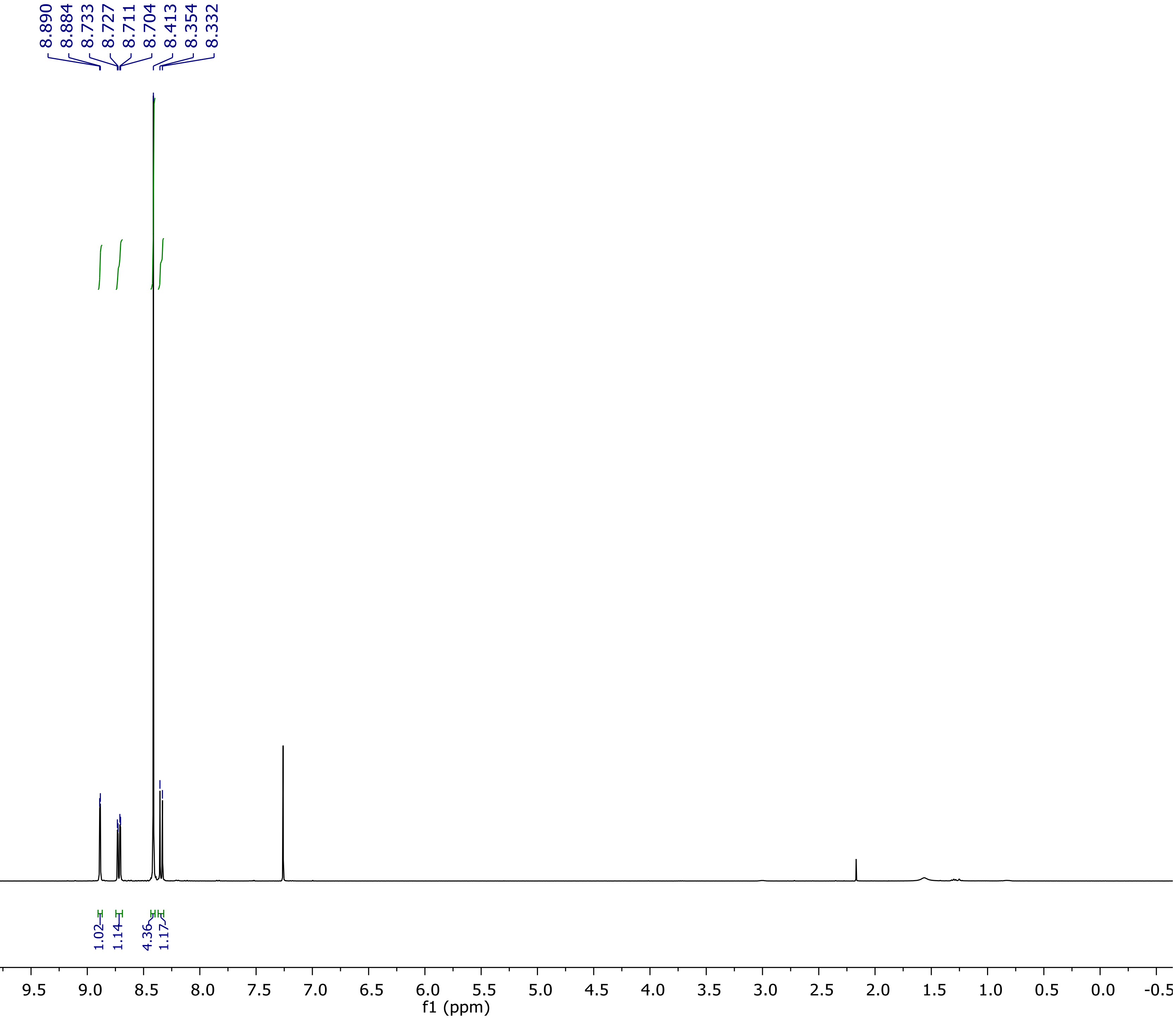

^{13}C NMR: 101 MHz, $CDCl_3$

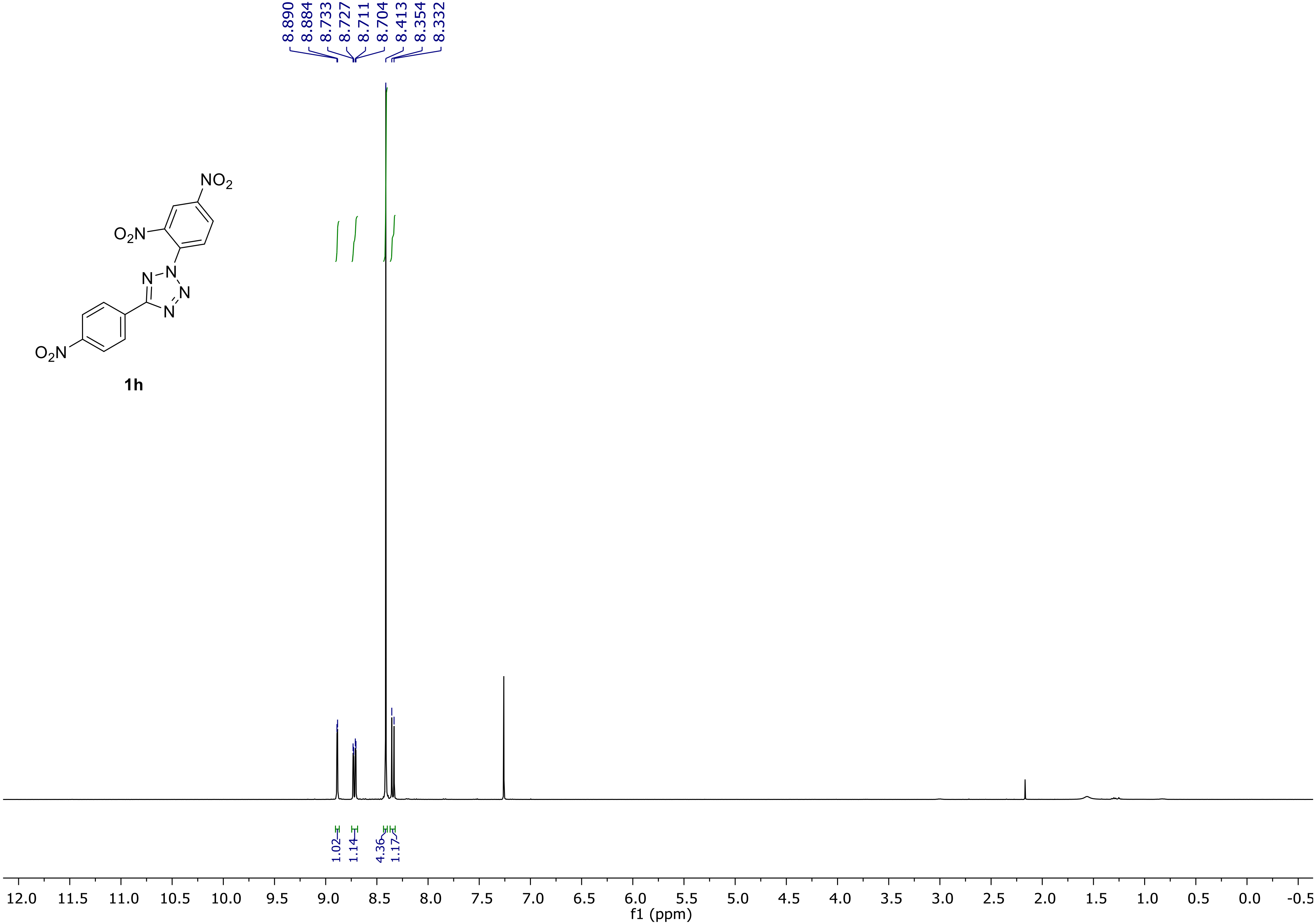

^{1}H NMR: 400 MHz, $CDCl_3$

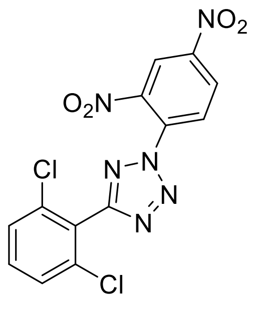

1i

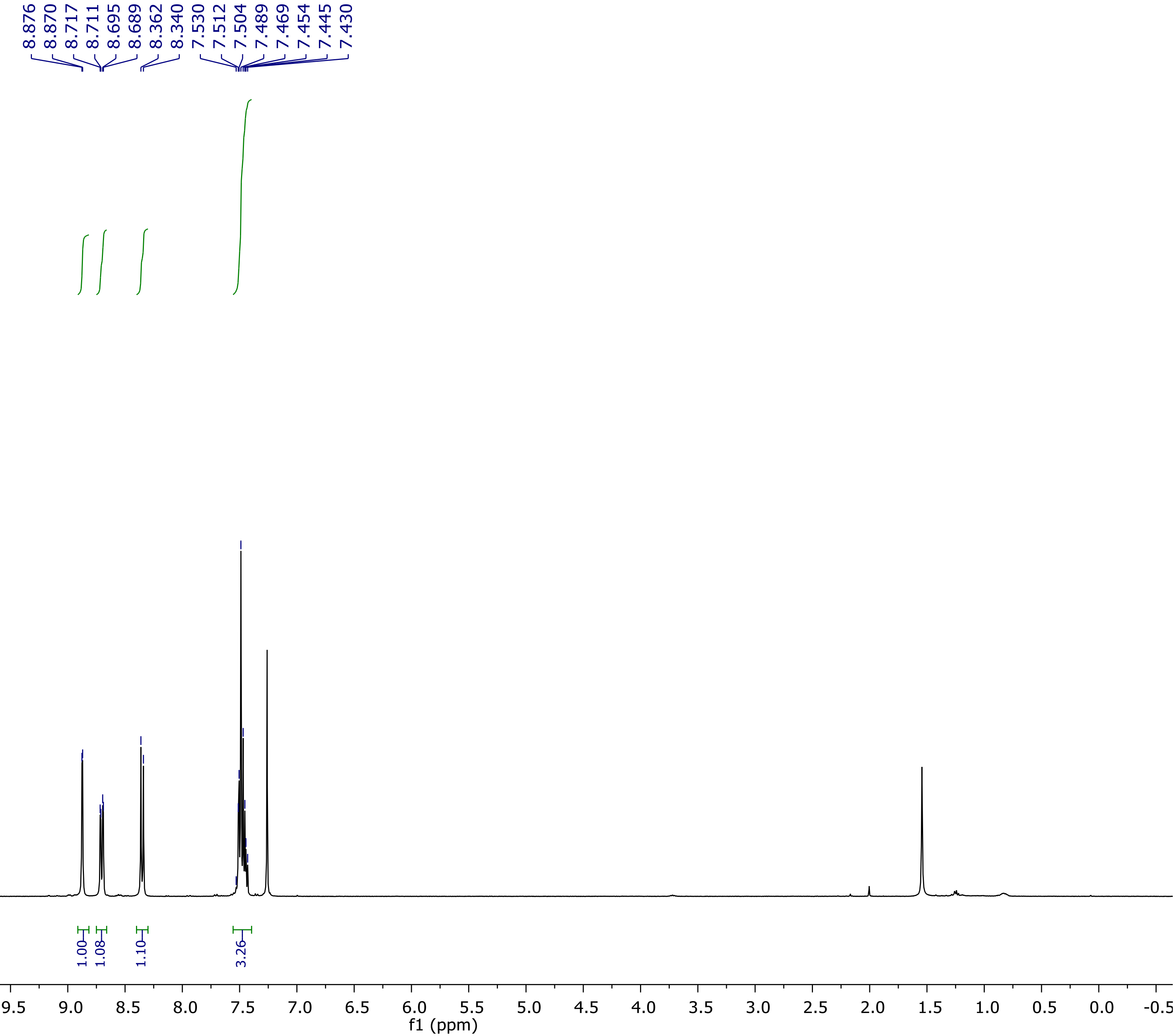

^{13}C NMR: 101 MHz, $CDCl_3$

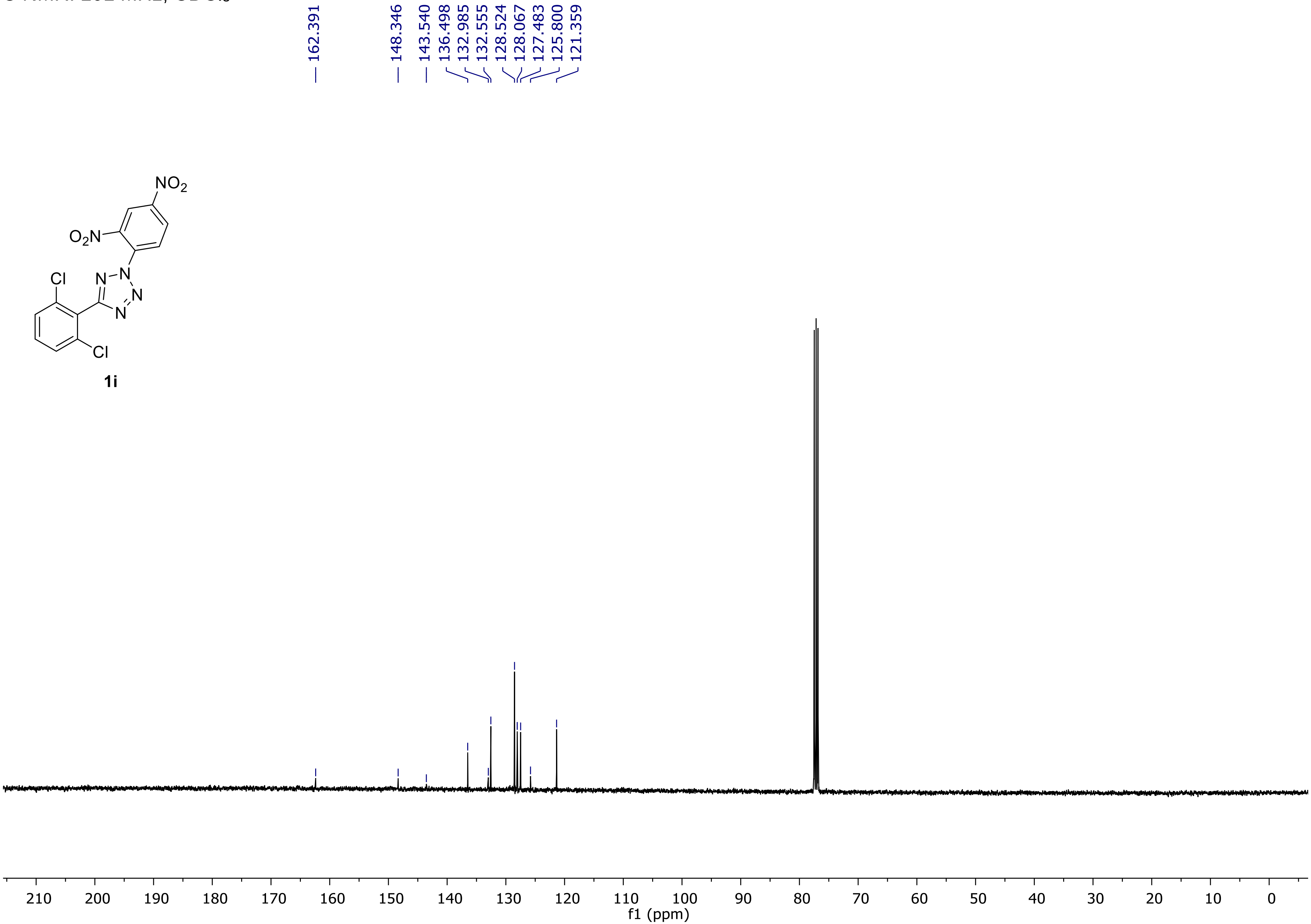

^{1}H NMR: 500 MHz, $CDCl_3$

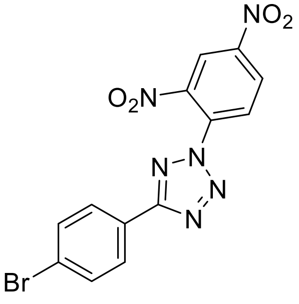

1j

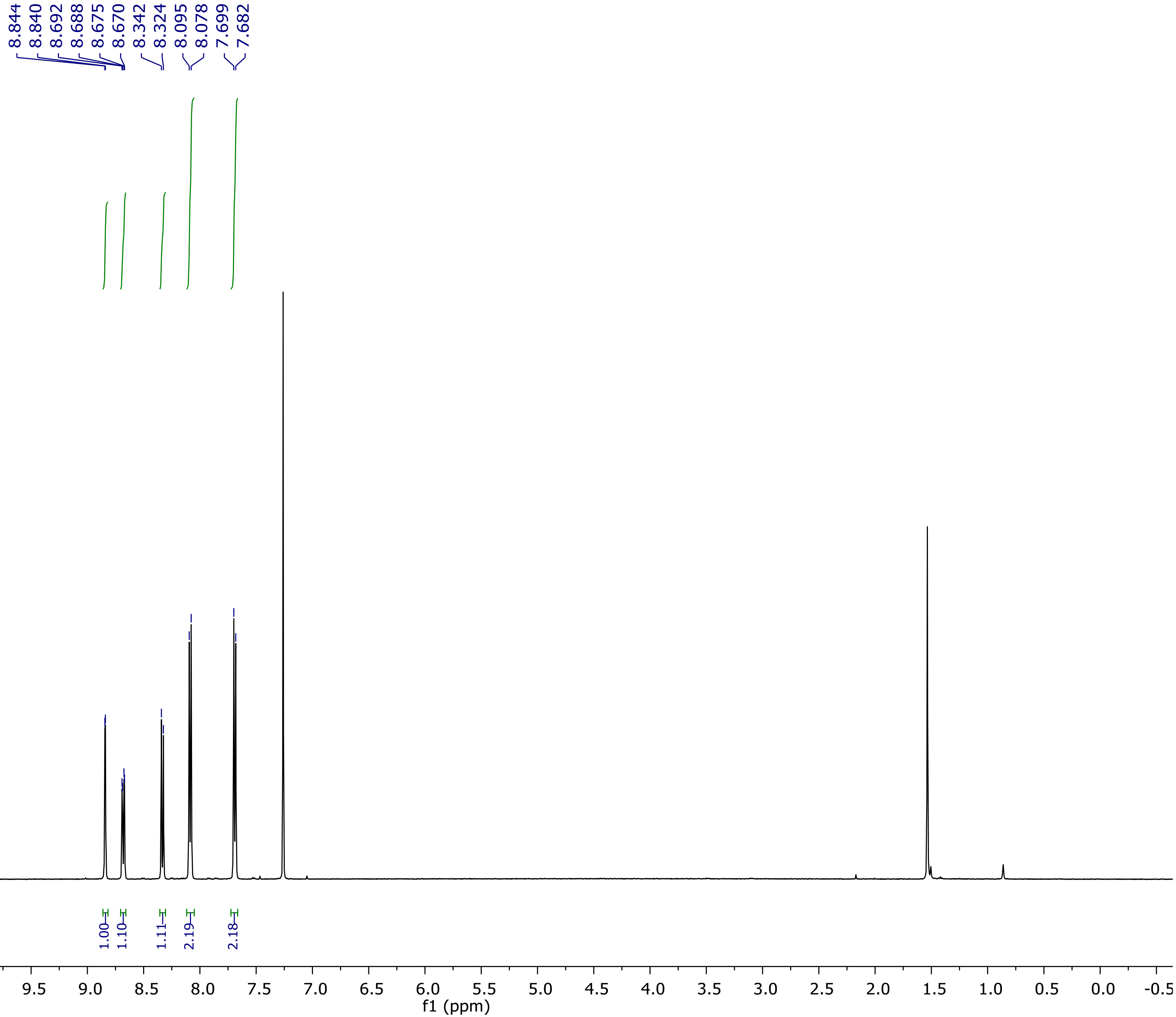

^{13}C NMR: 126 MHz, $CDCl_3$

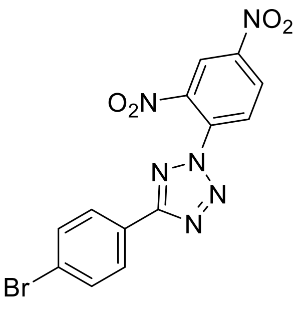

1j

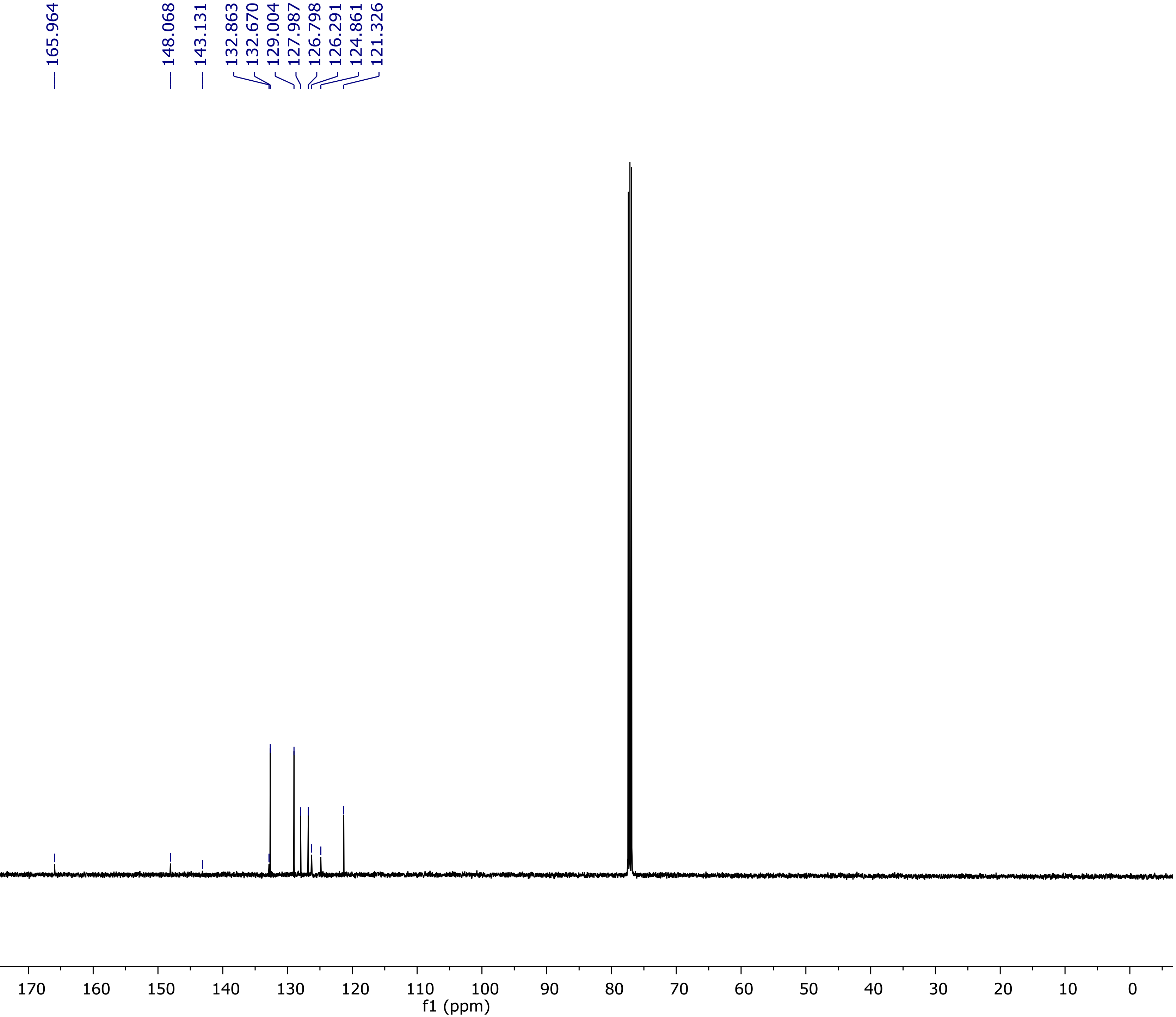

^1H NMR: 50 MHz, $CDCl_3$

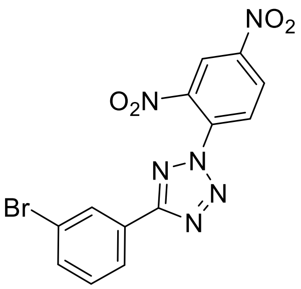

1k

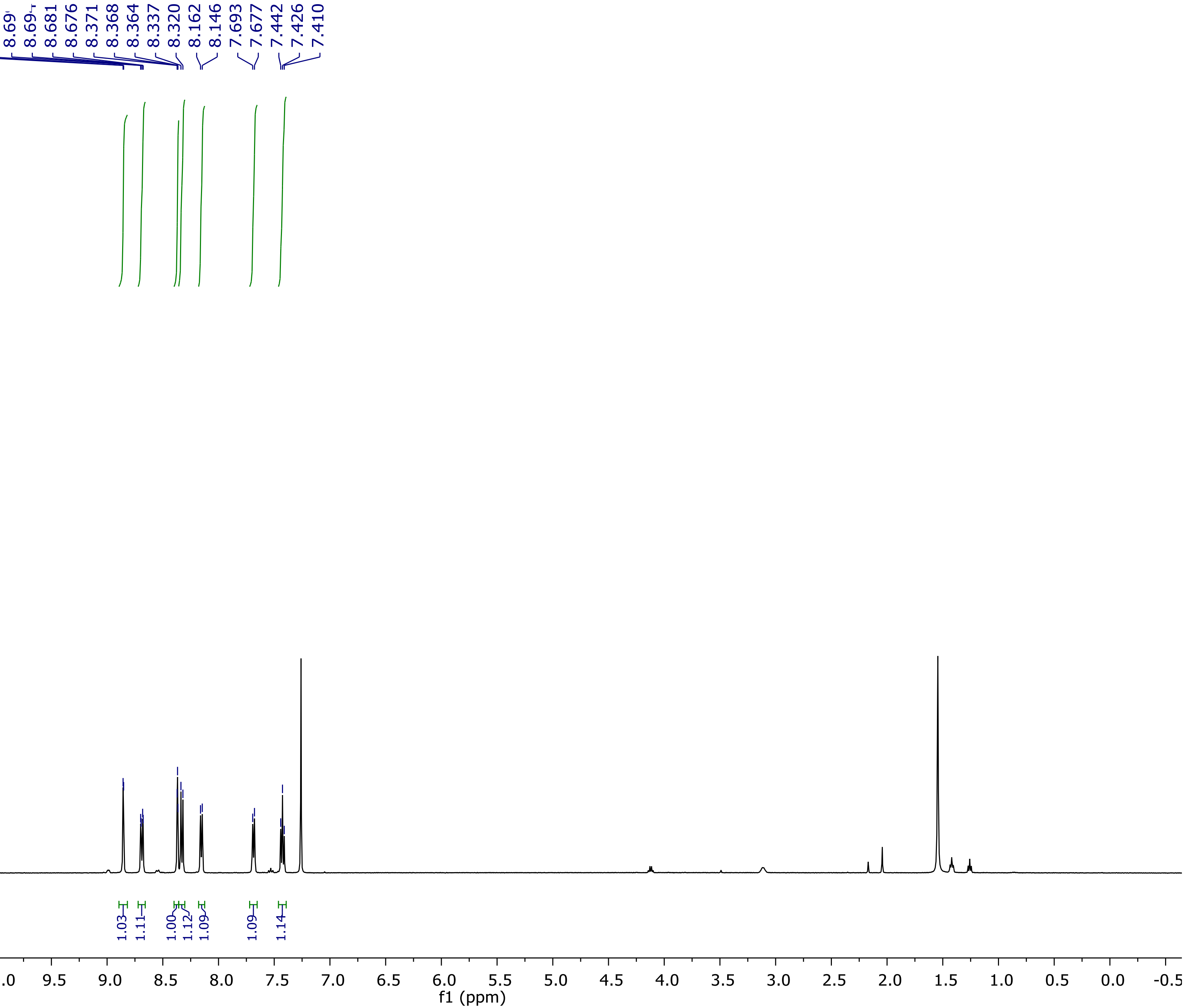

^{13}C NMR: 126 MHz, $CDCl_3$

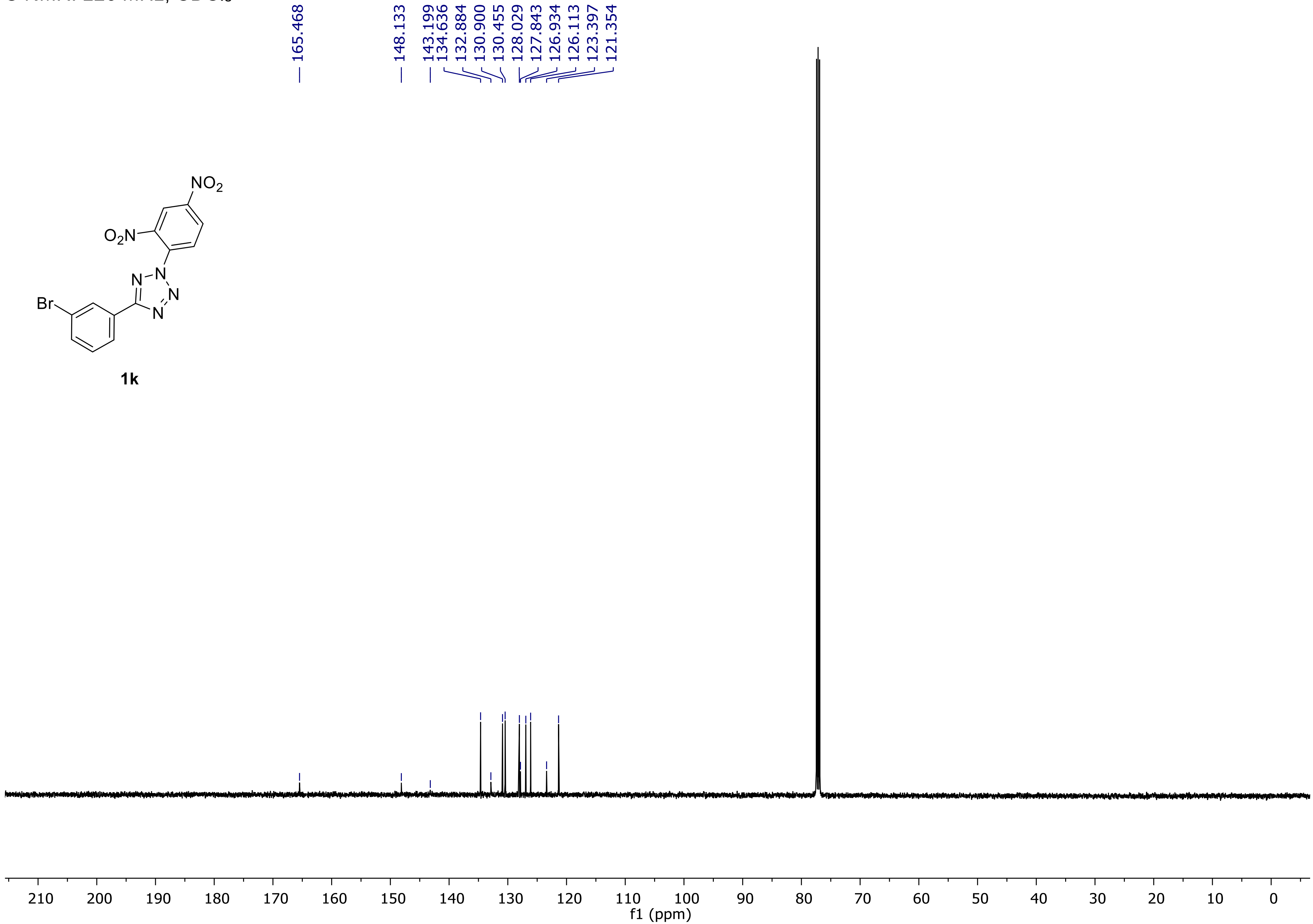

1k

^1H NMR: 400 MHz, $CDCl_3$

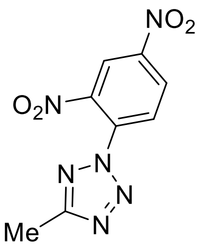

1l

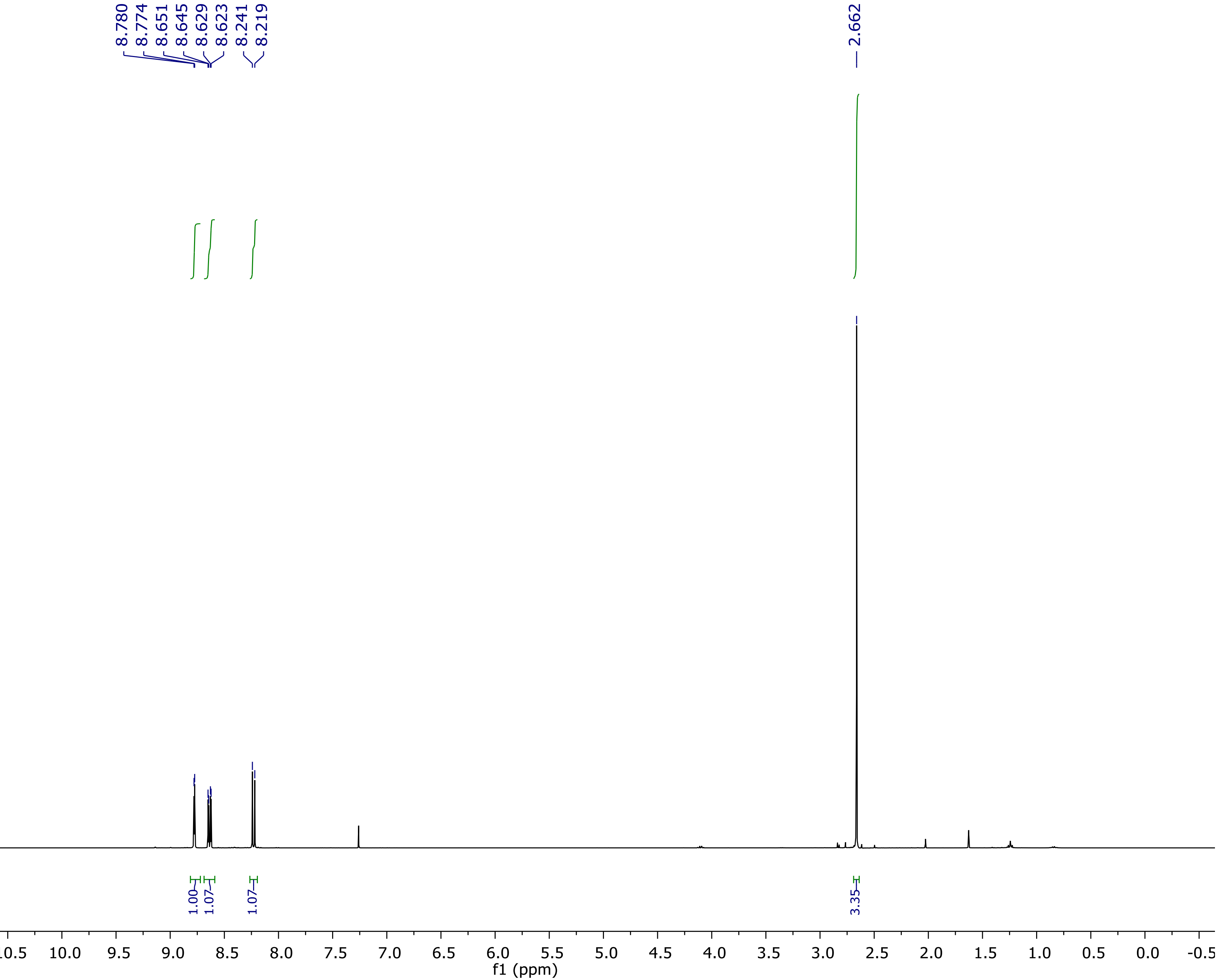

^{13}C NMR: 101 MHz, $CDCl_3$

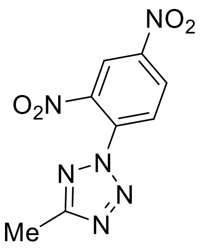

1l

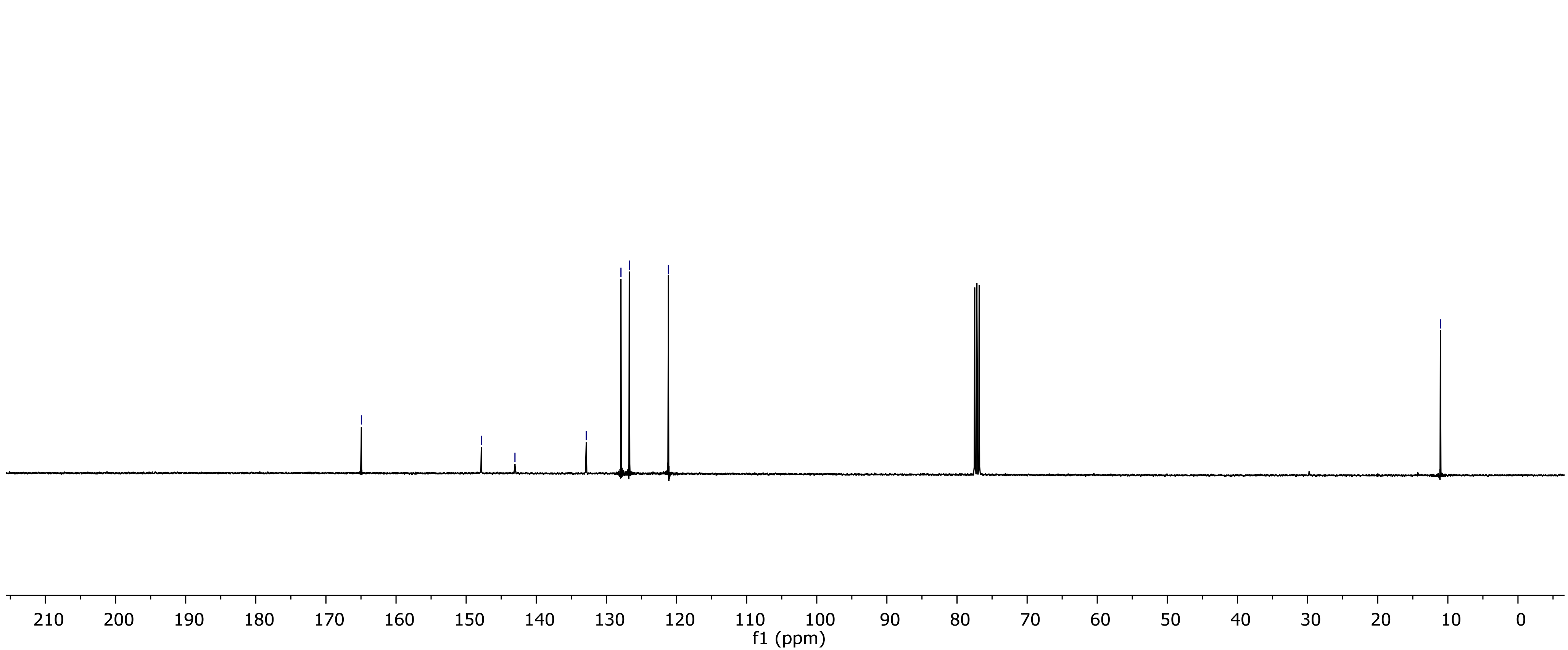

^{1}H NMR: 400 MHz, $CDCl_3$

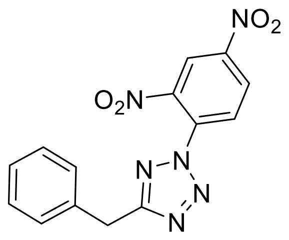

1m

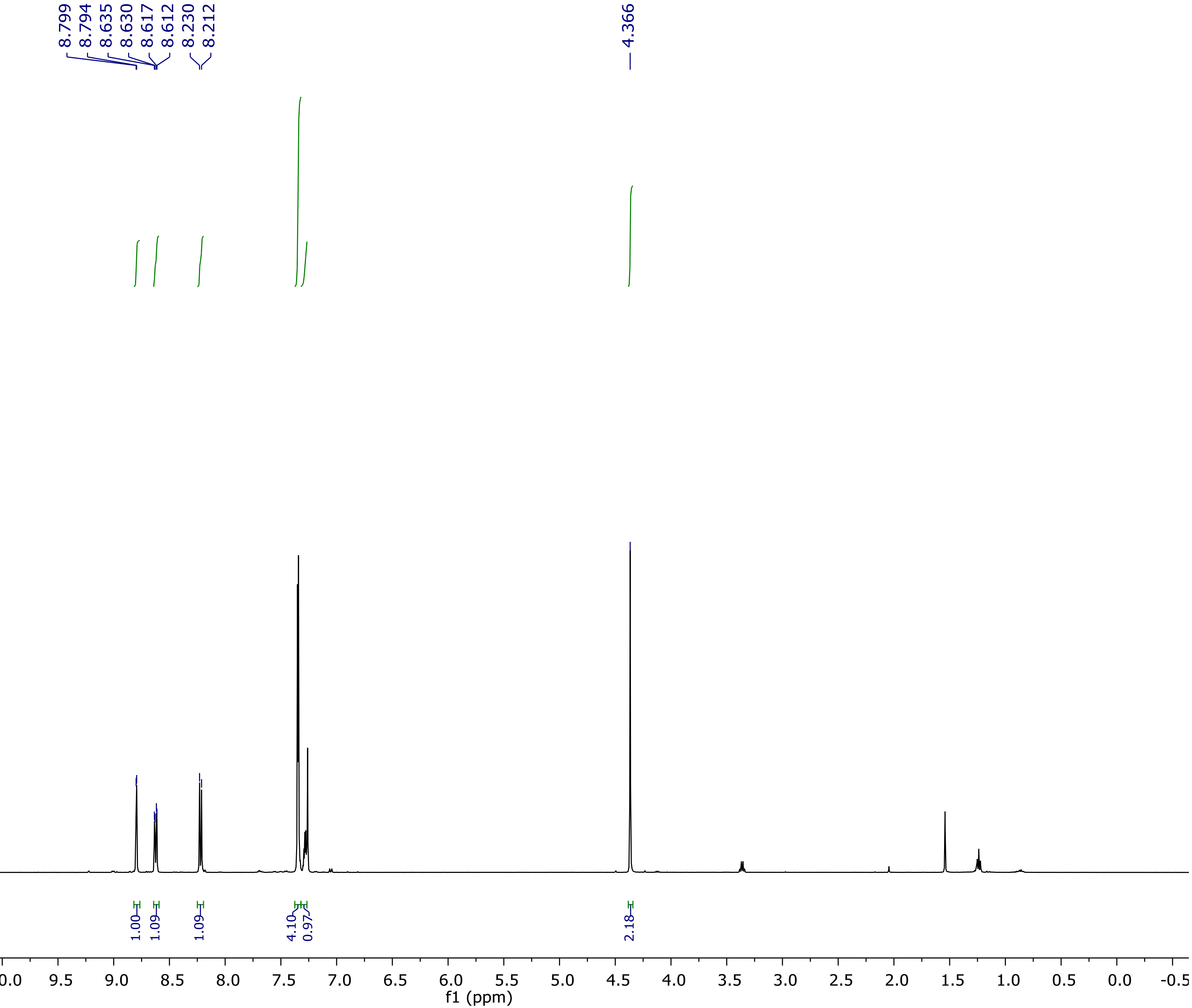

^{13}C NMR: 101 MHz, $CDCl_3$

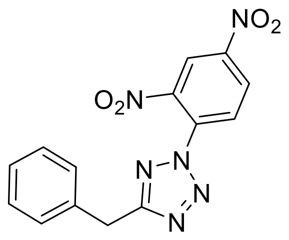

1m

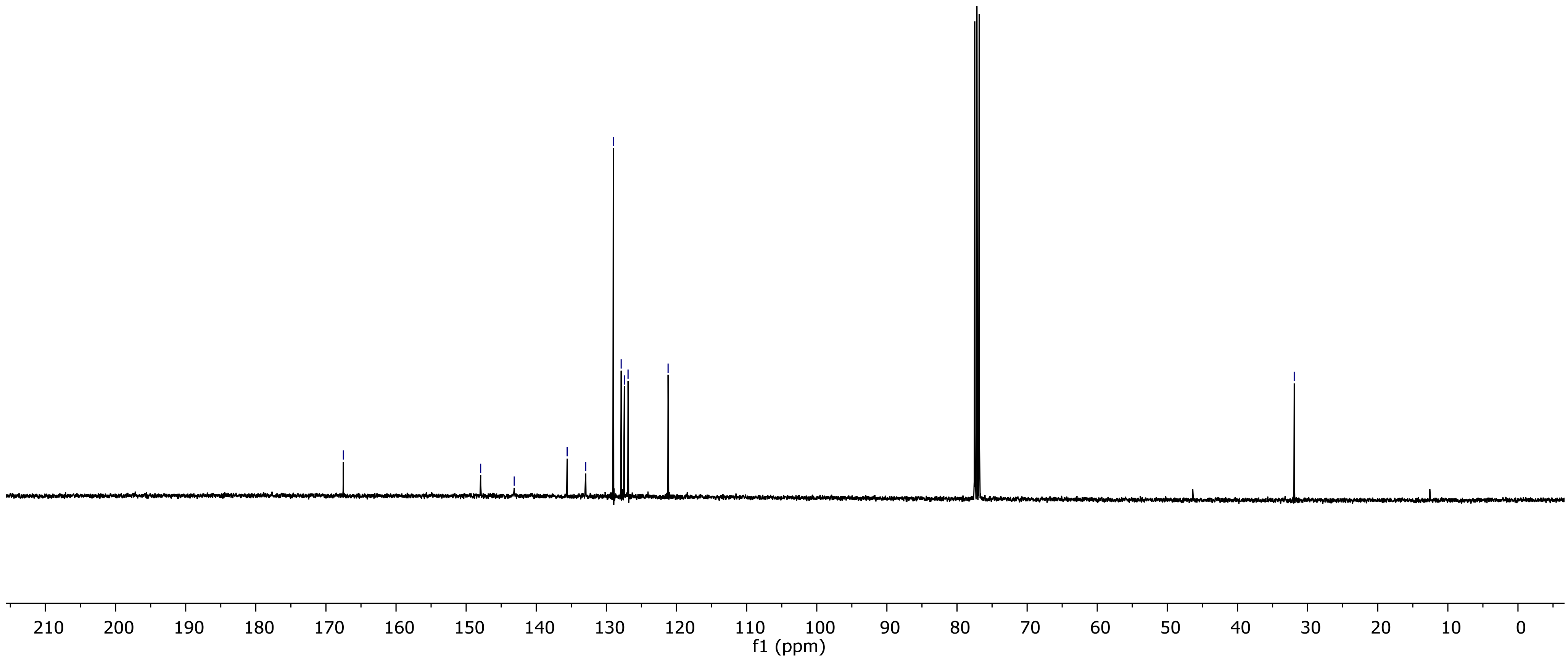

^1H NMR: 500 MHz, $CDCl_3$

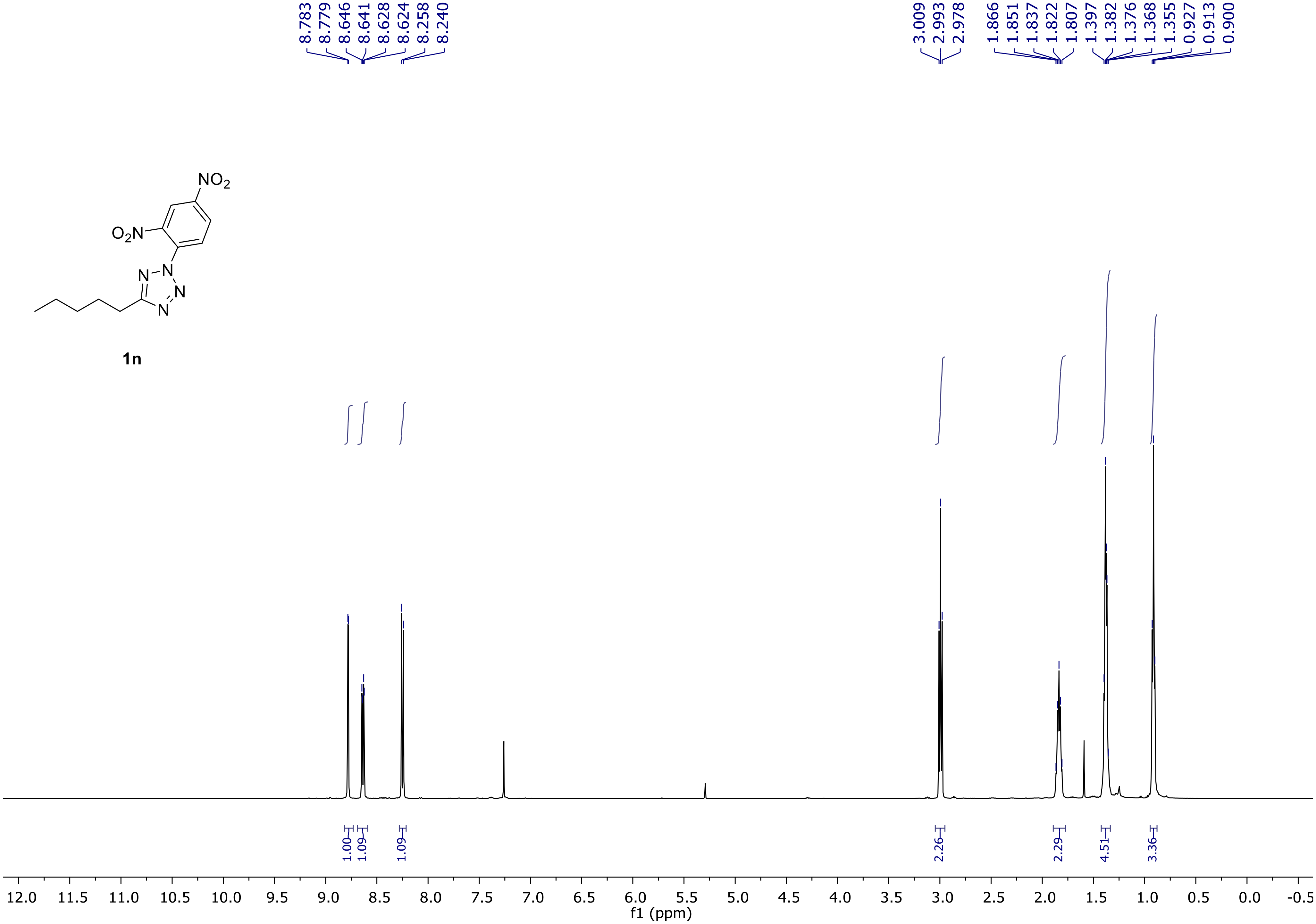

^{13}C NMR: 126 MHz, $CDCl_3$

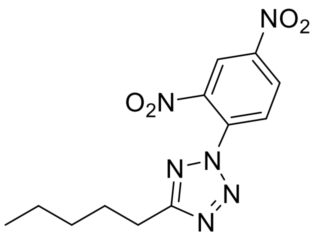

1n

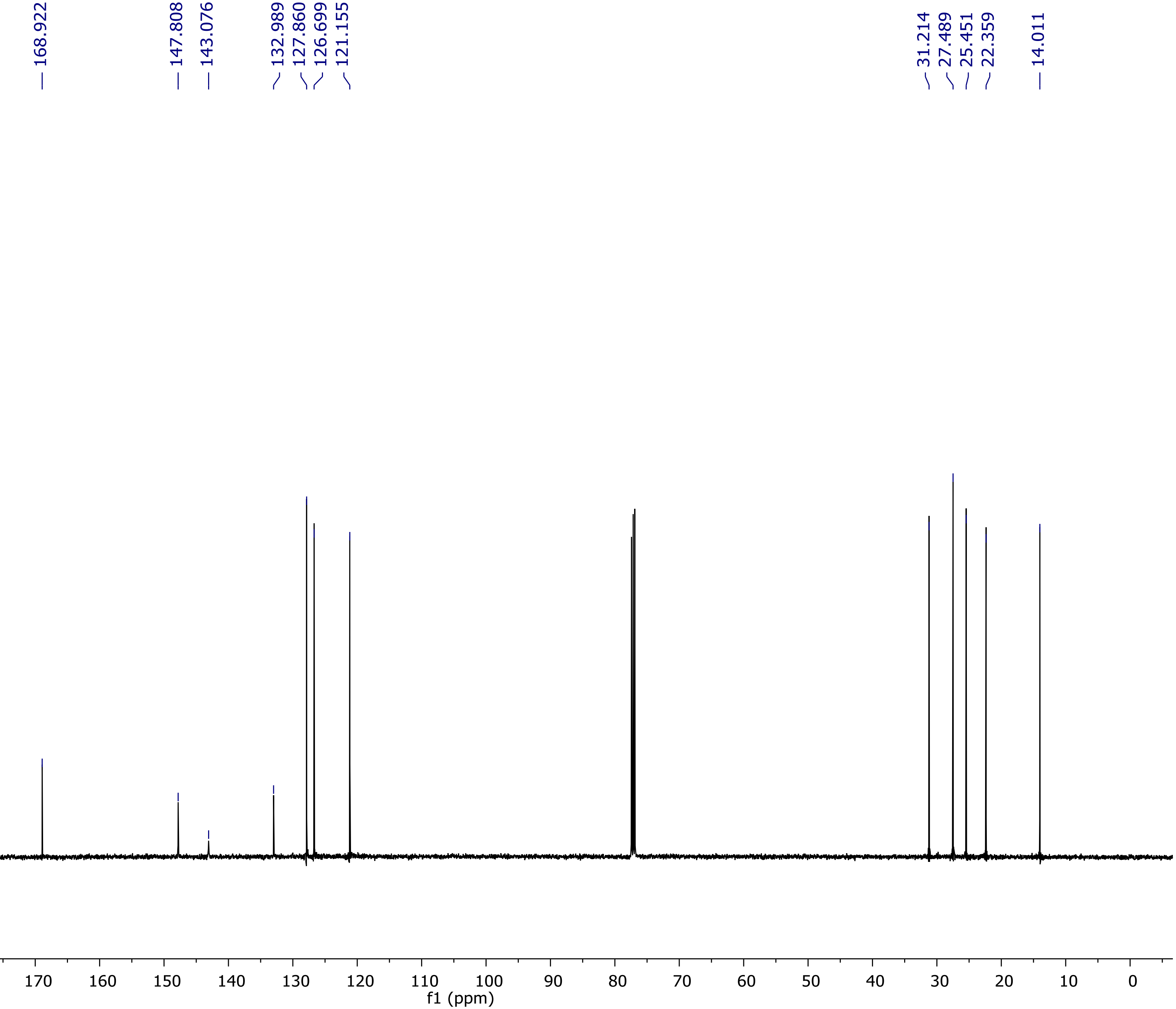

^{1}H NMR: 500 MHz, $CDCl_3$

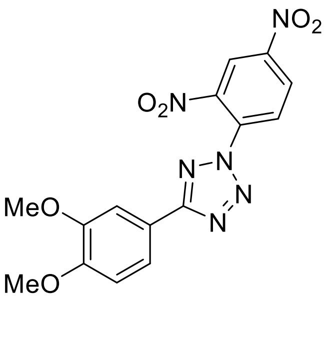

1o

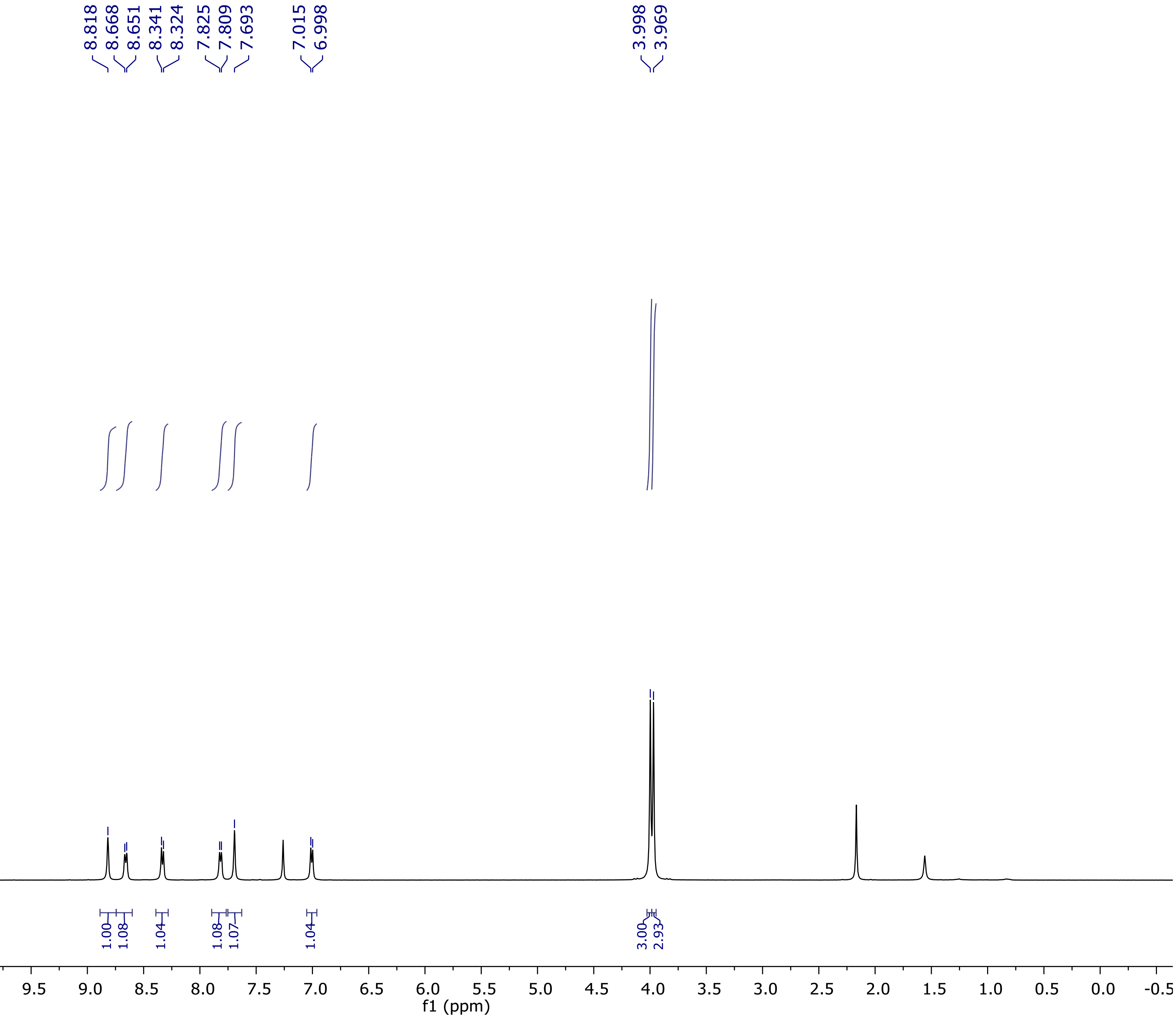

^{13}C NMR: 126 MHz, $CDCl_3$

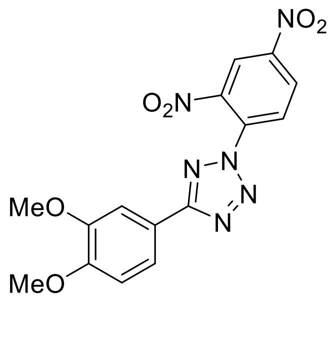

1o

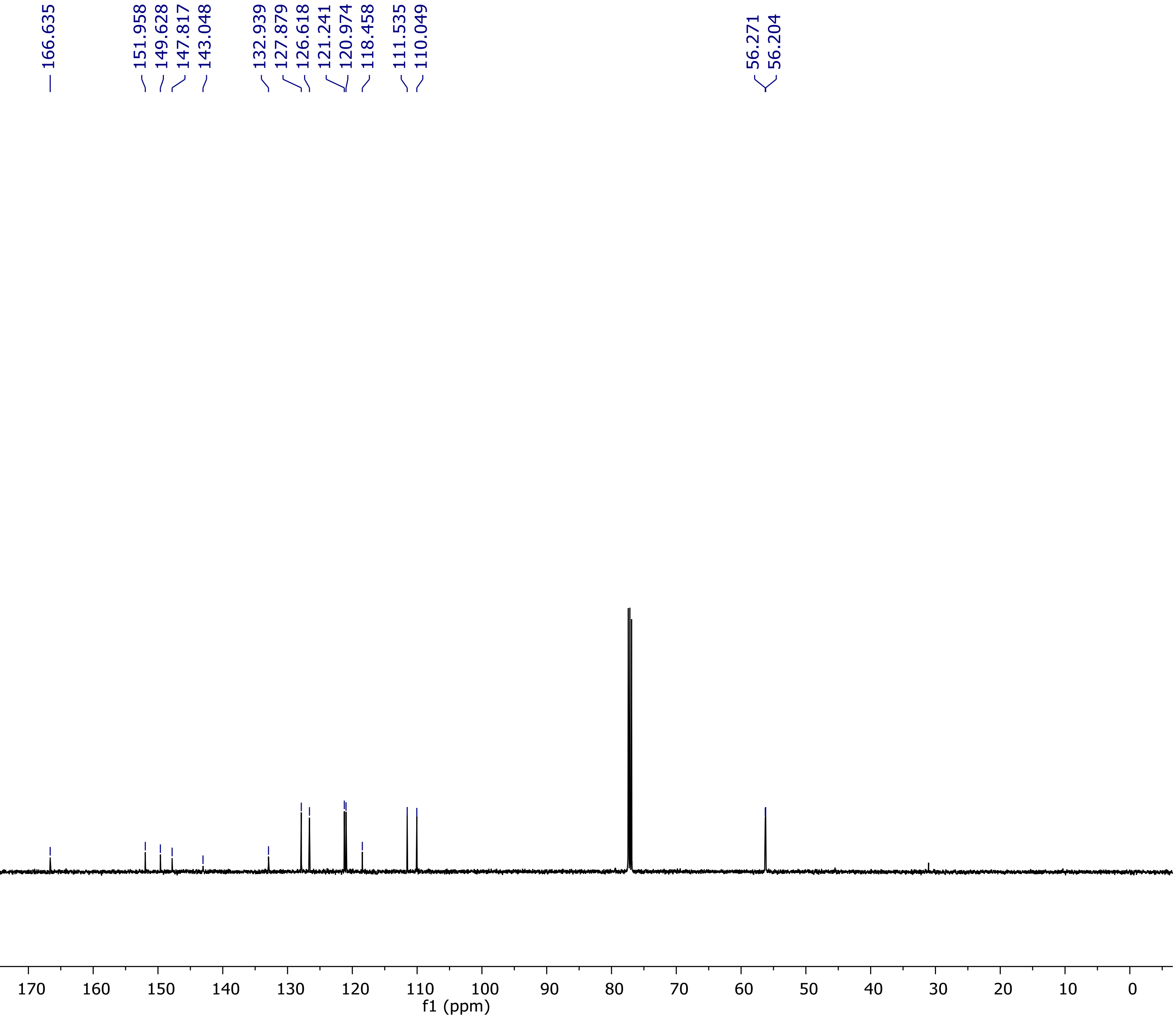

^{1}H NMR: 500 MHz, $CDCl_3$

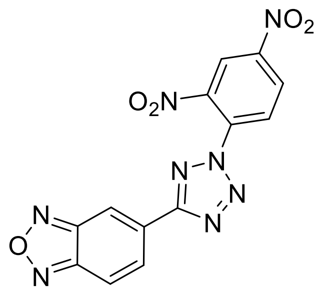

1p

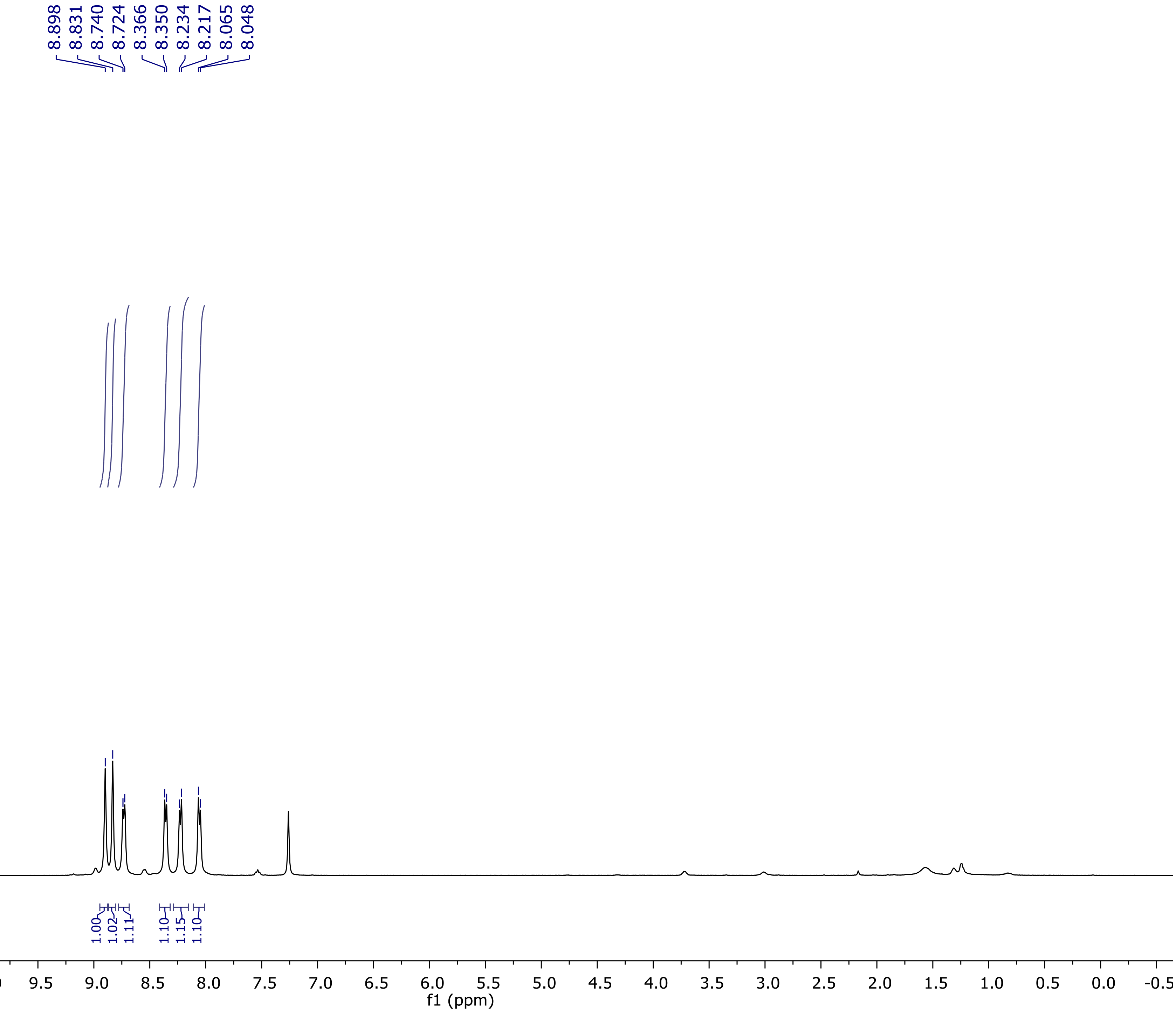

^{13}C NMR: 126 MHz, $CDCl_3$

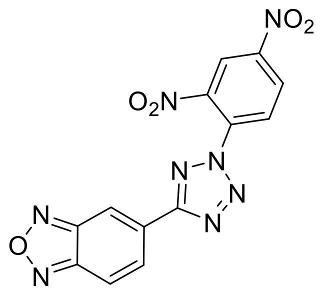

1p

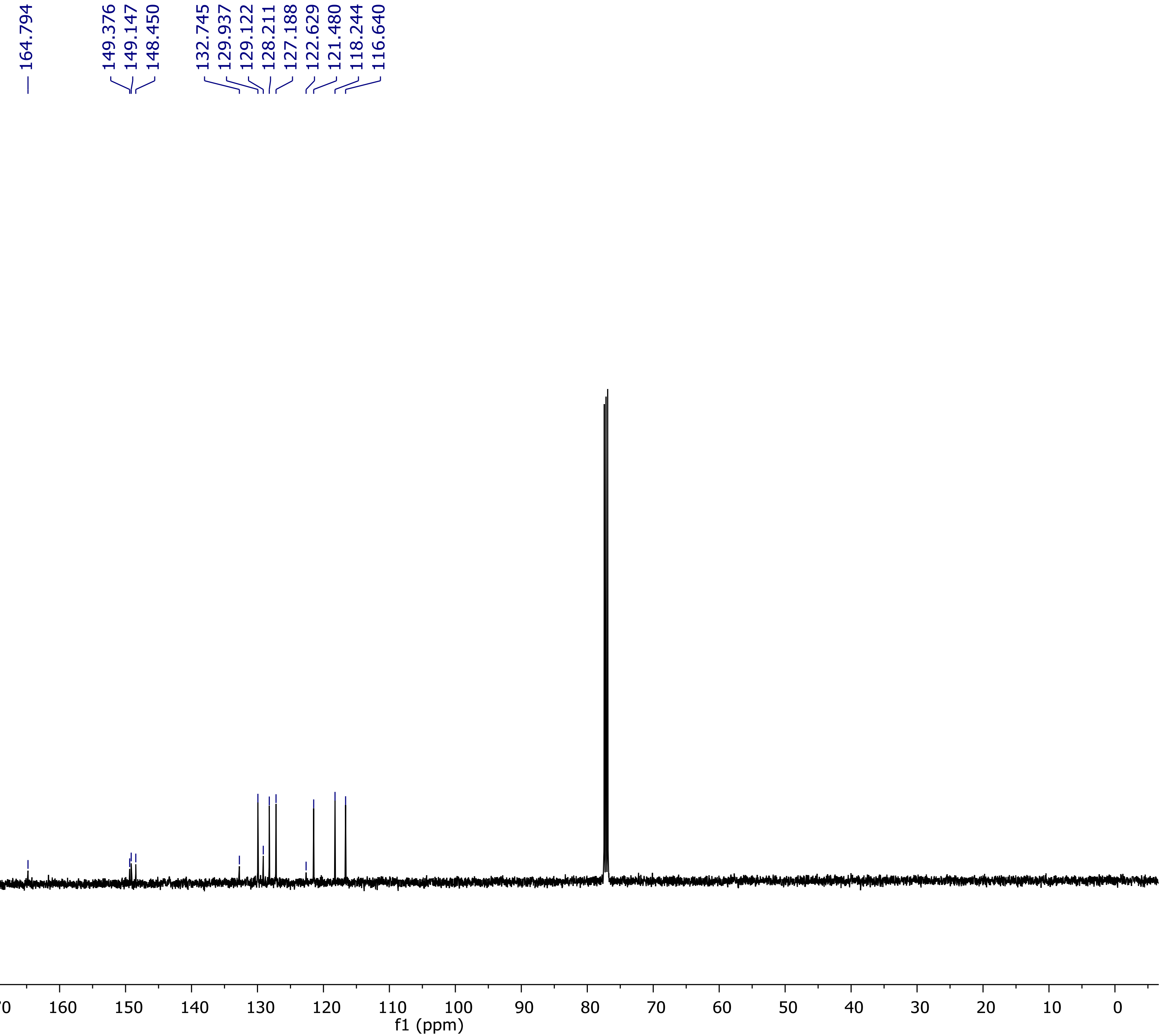

^{1}H NMR: 400 MHz, CDCl3

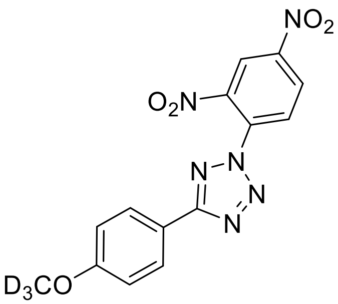

1q

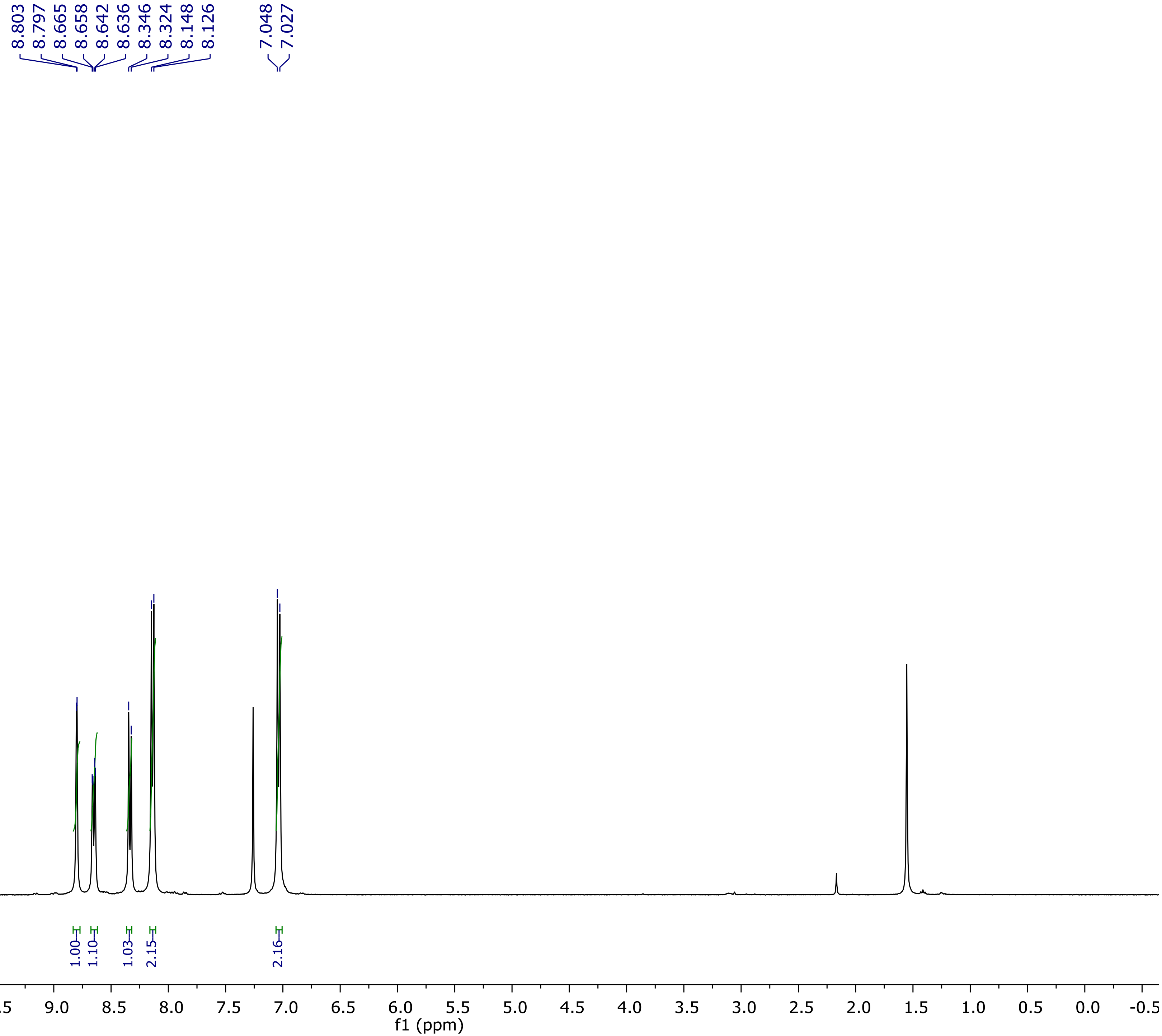

^{13}C NMR: 101 MHz, $CDCl_3$

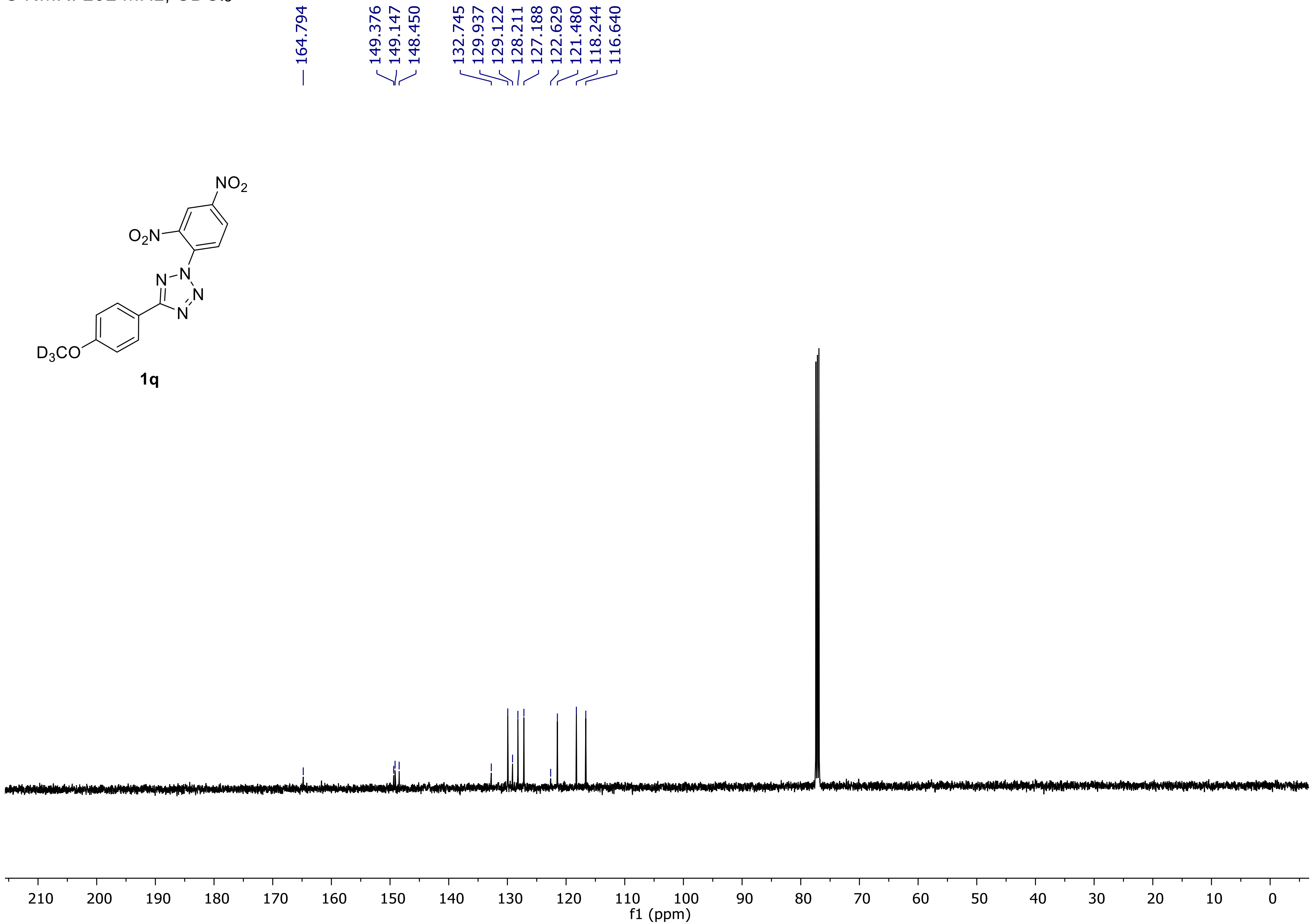

1q

^{1}H NMR: 400 MHz, $CDCl_3$

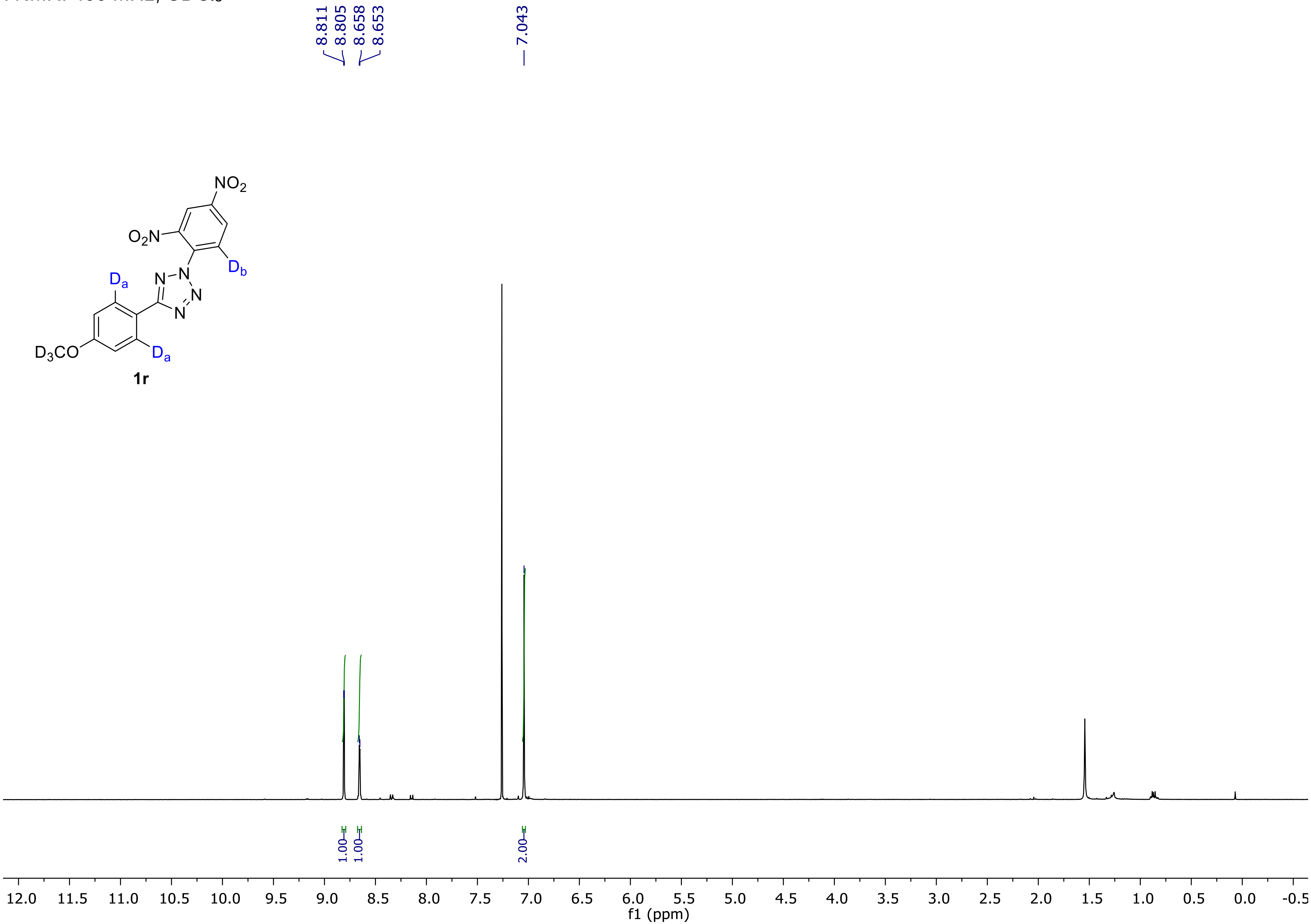

^{13}C NMR: 101 MHz, $CDCl_3$

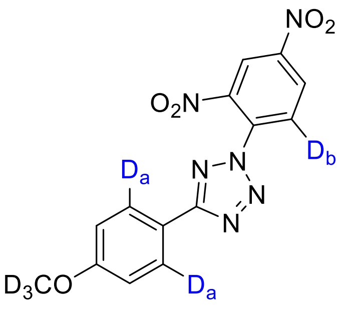

1r

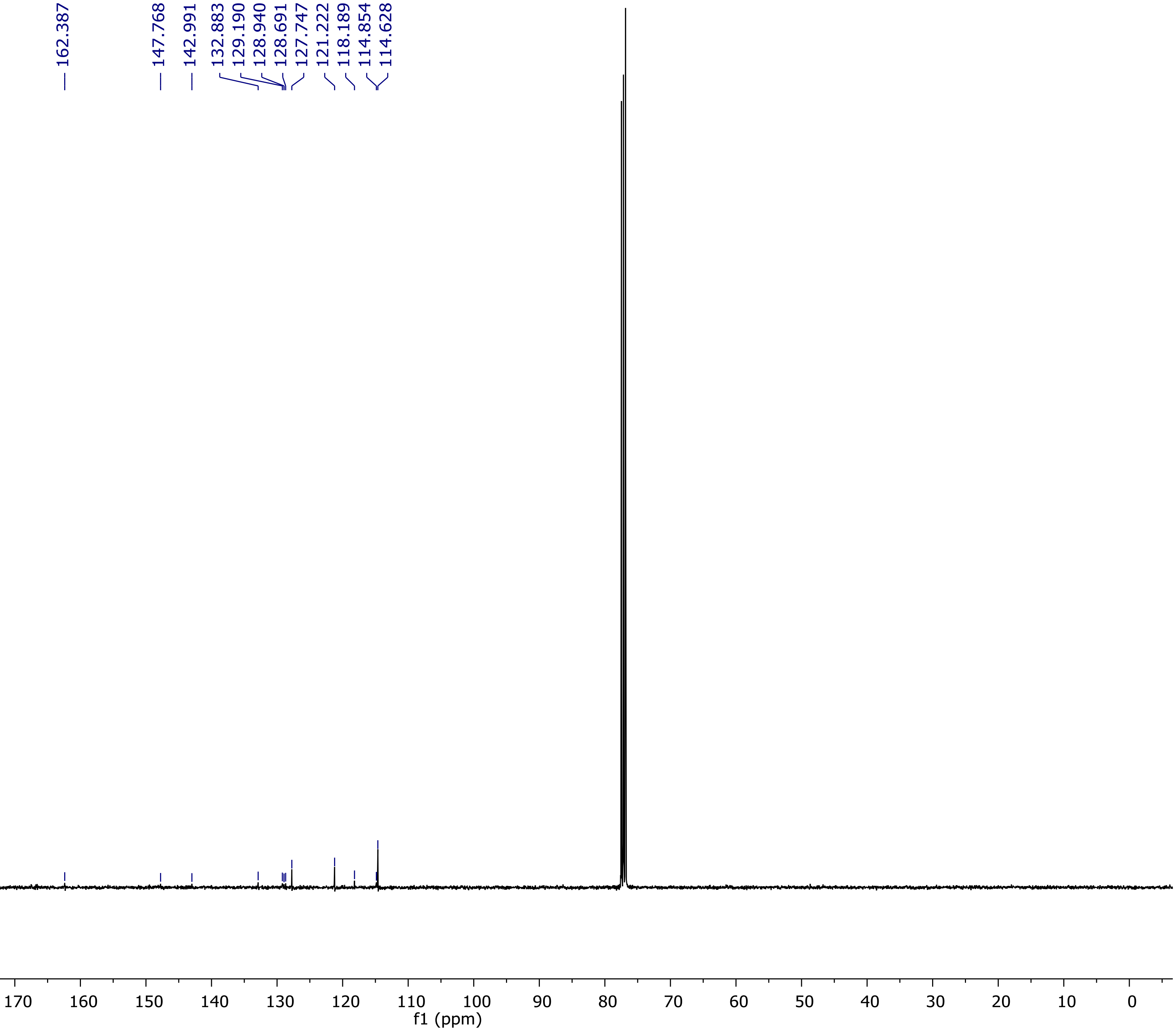

^{1}H NMR: 500 MHz, D_6-DMSO

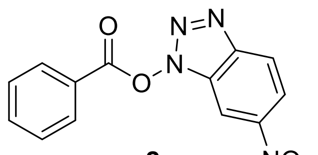

2a

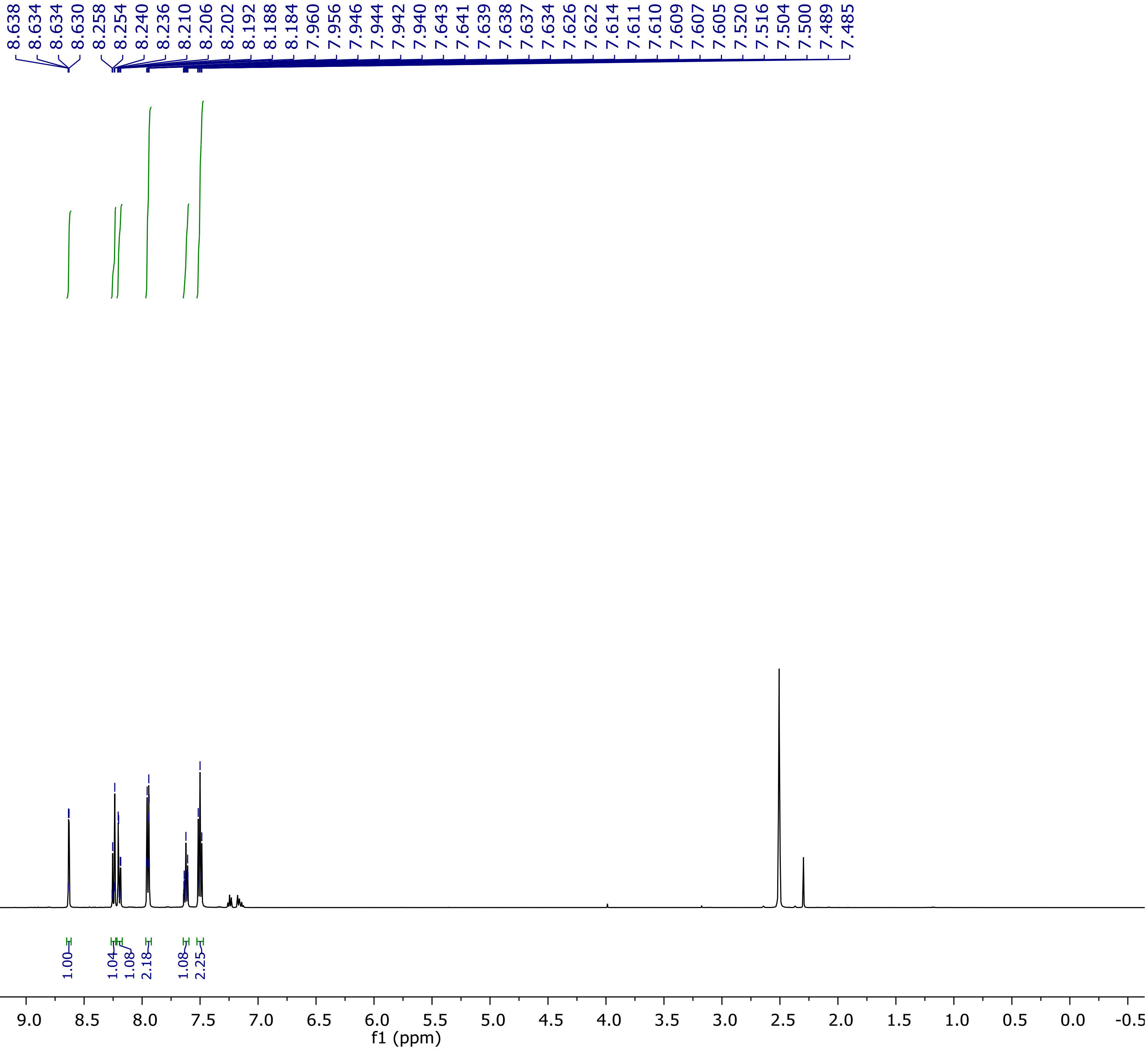

^{13}C NMR: 126 MHz, D_6-DMSO

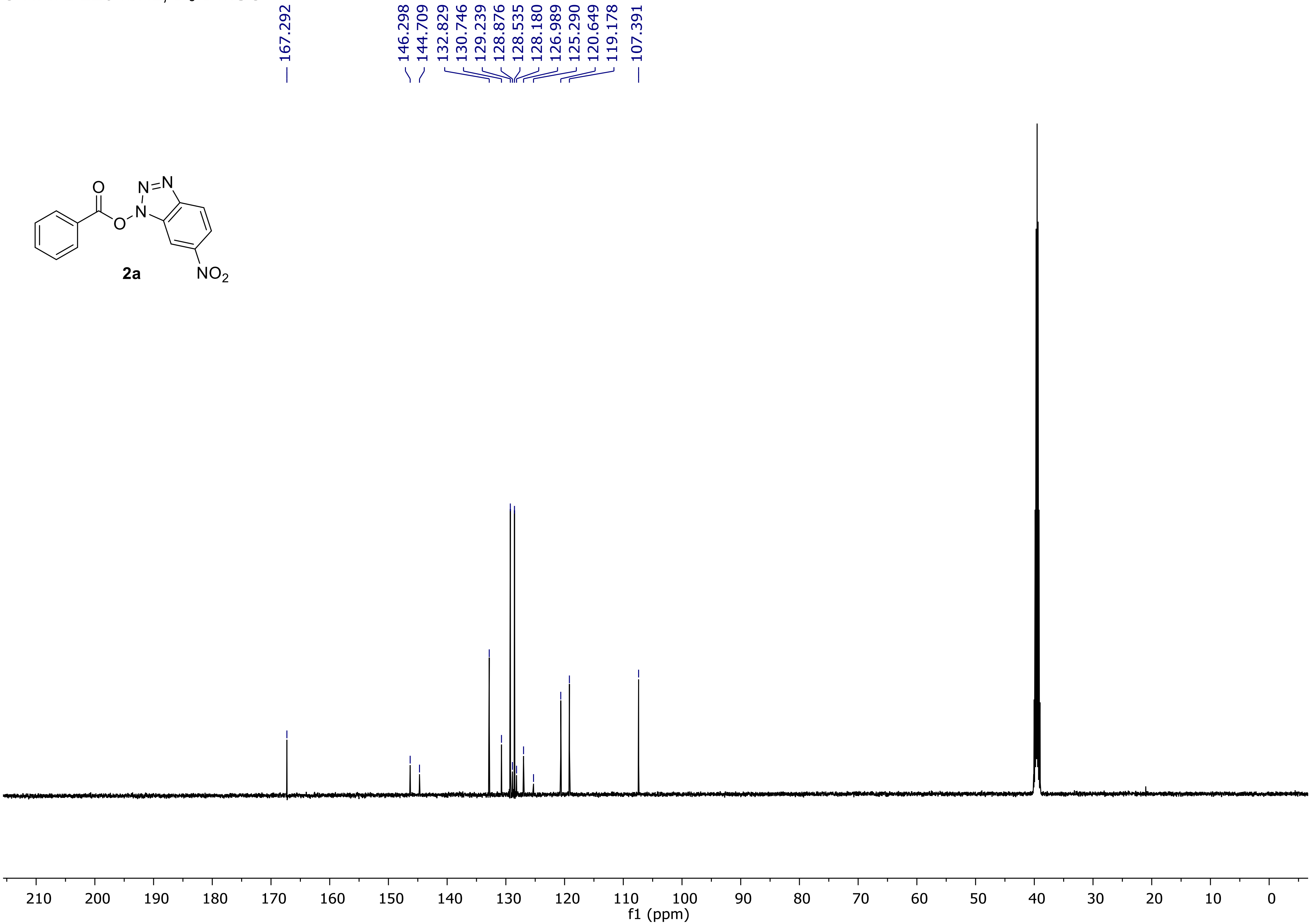

^{1}H NMR: 400 MHz, $CDCl_3$

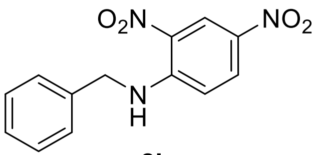

2b

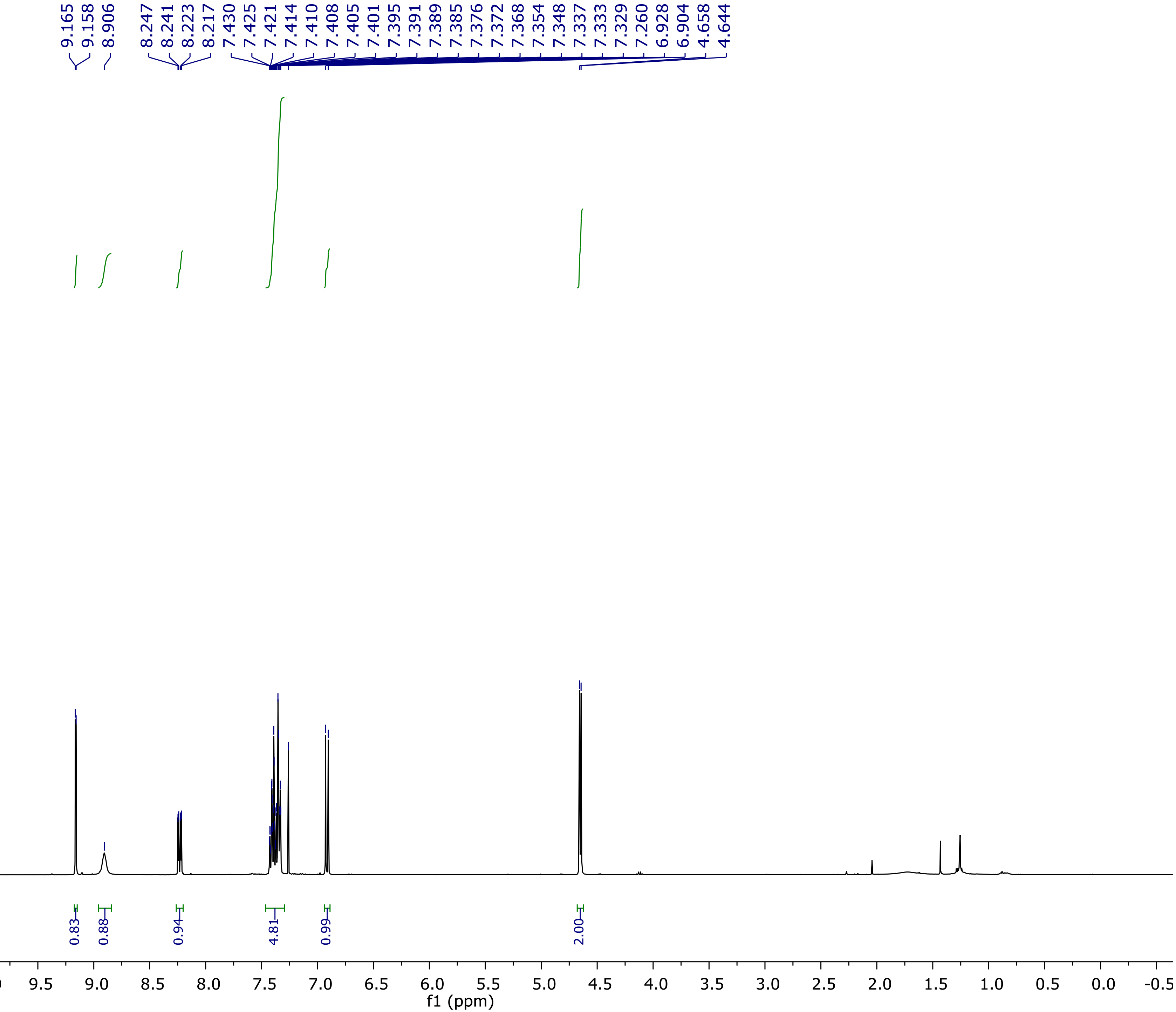

^{13}C NMR: 101 MHz, $CDCl_3$

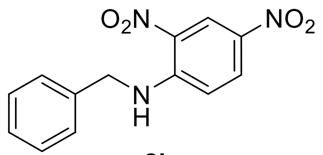

2b

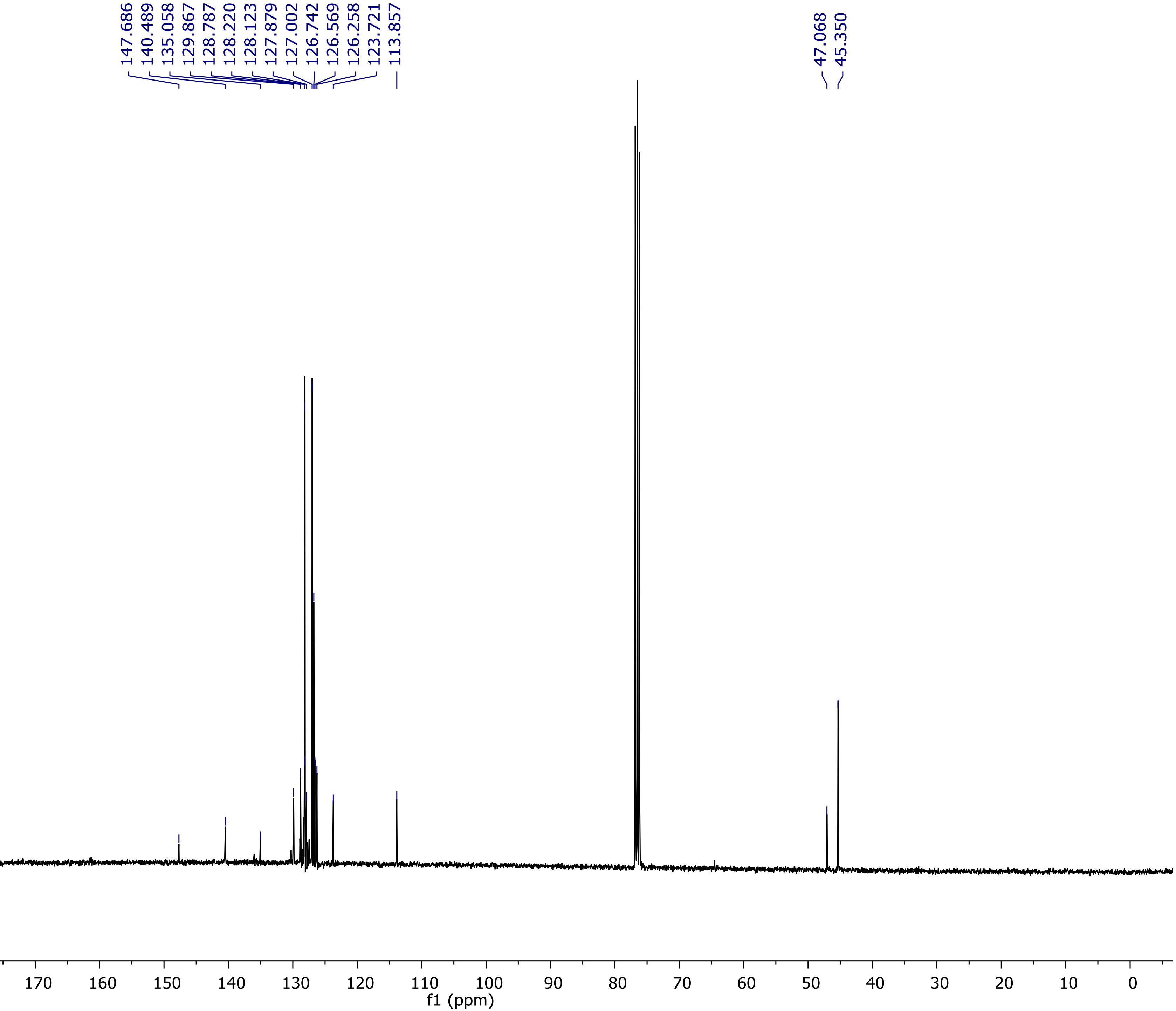

^{1}H NMR: 500 MHz, D_6-DMSO

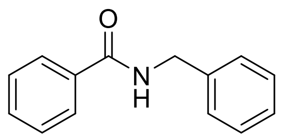

3a

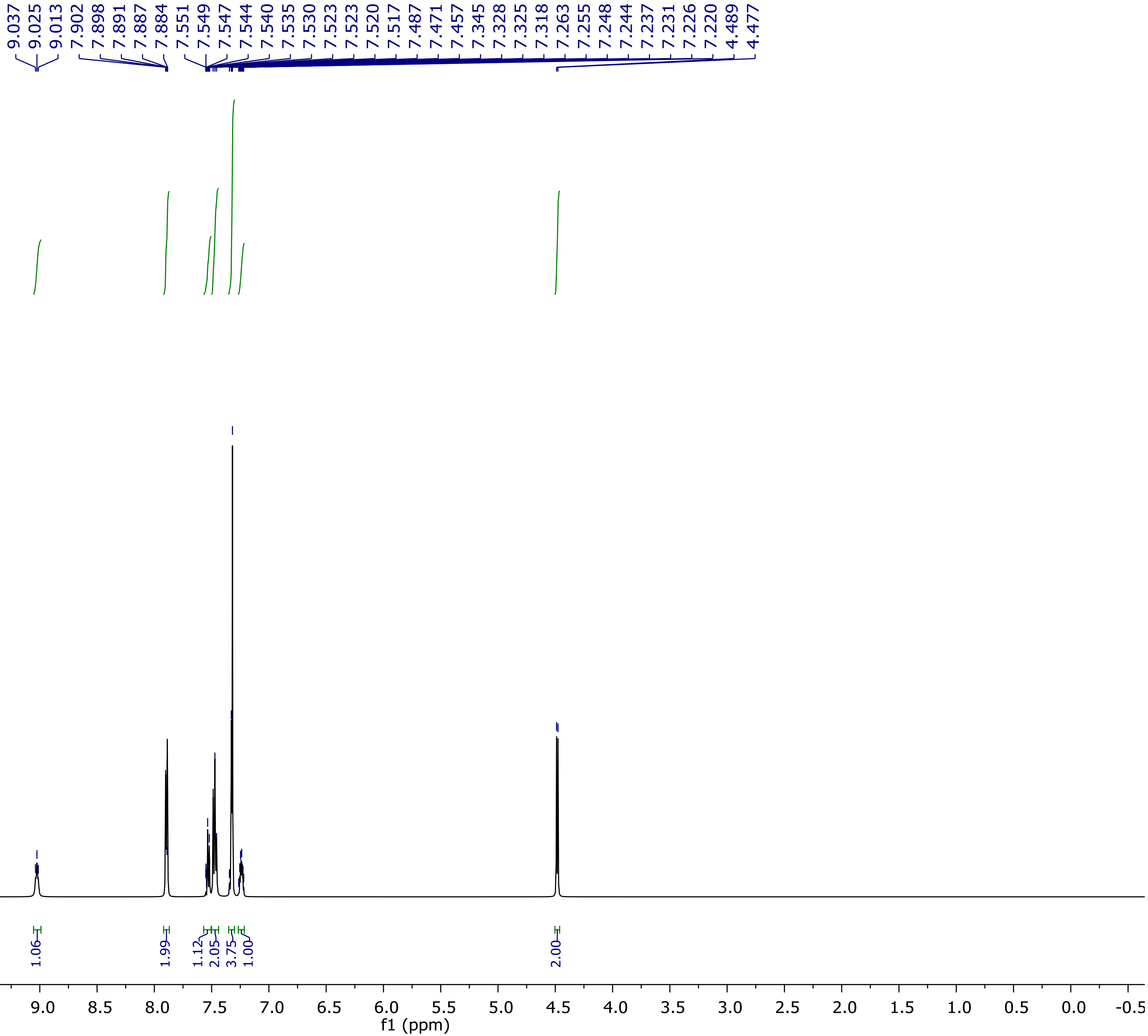

^{13}C NMR: 126 MHz, D_6-DMSO

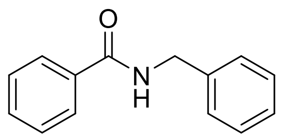

3a

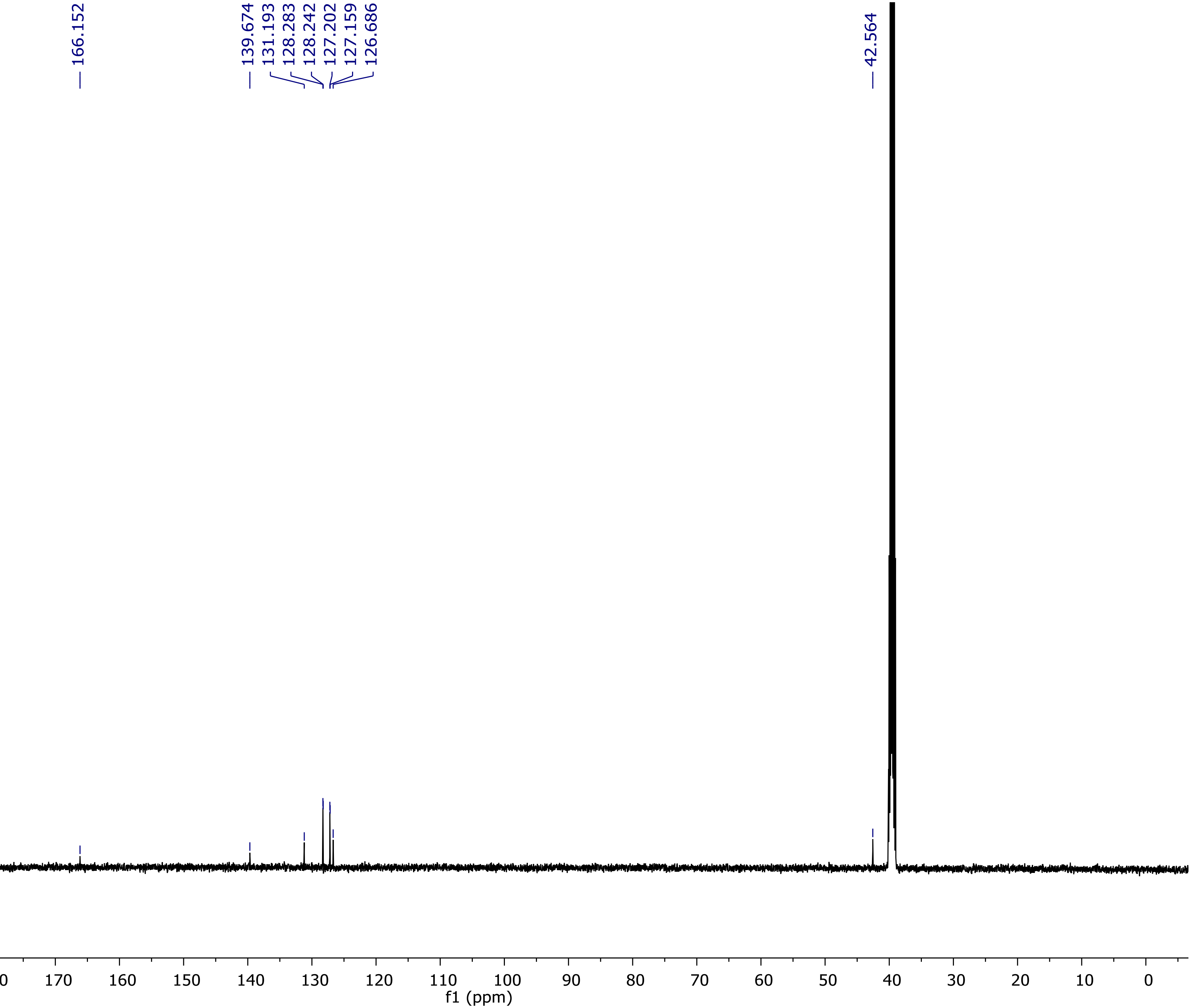

^{1}H NMR: 500 MHz, D_6-DMSO

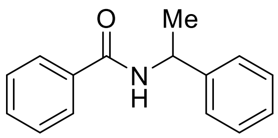

3b

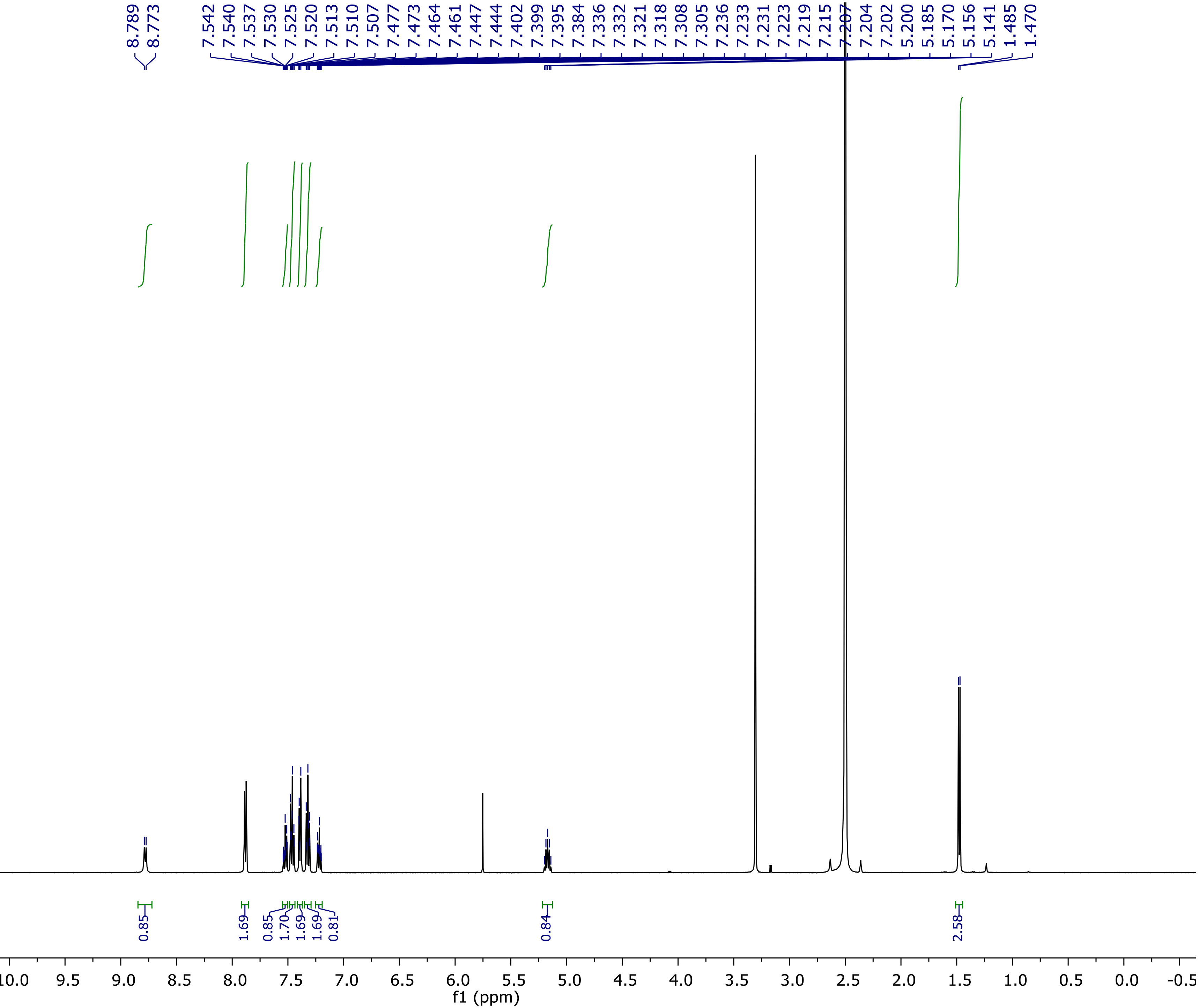

^{13}C NMR: 126 MHz, D_6-DMSO

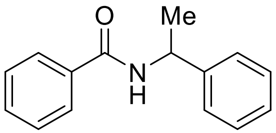

3b

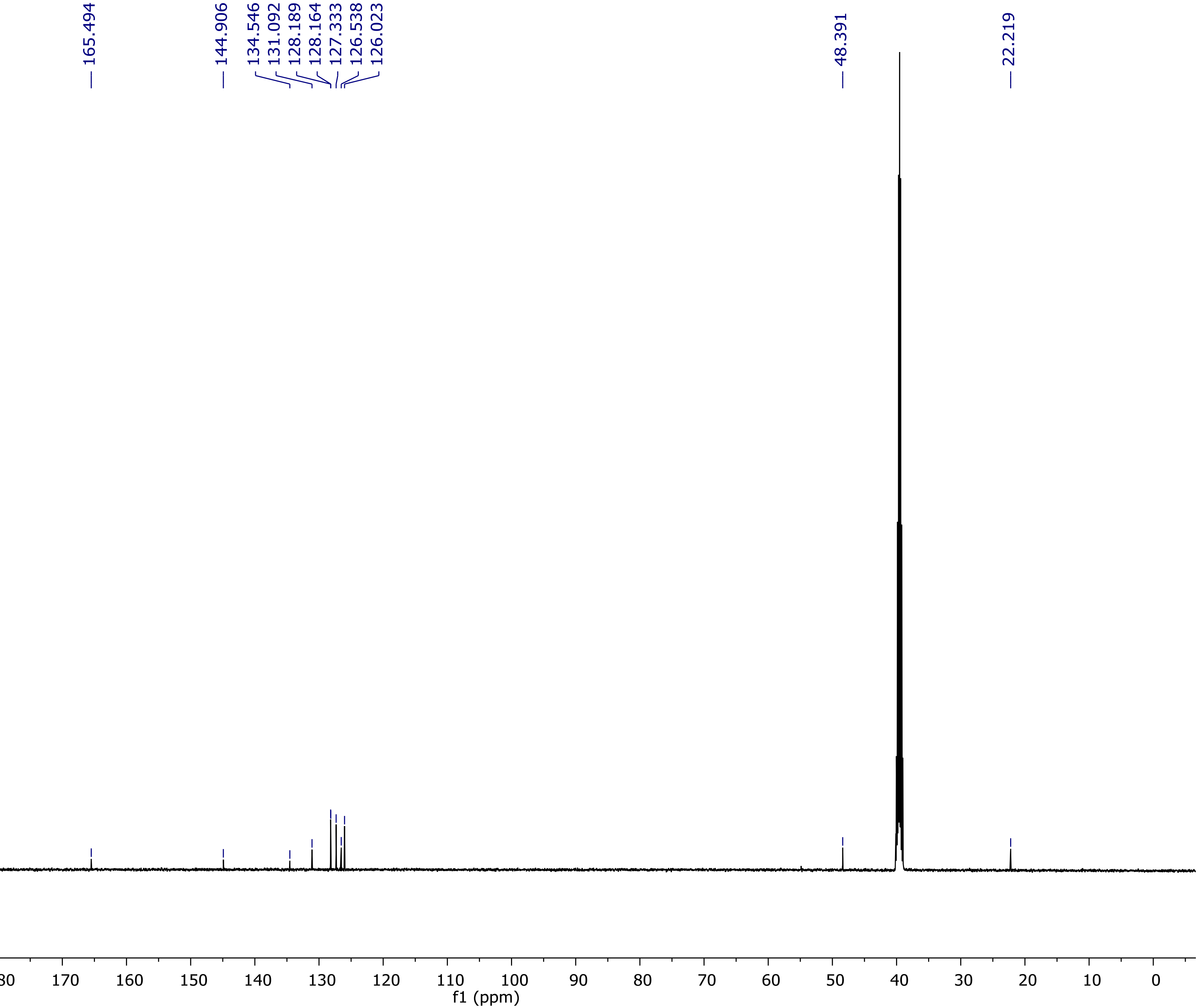

^1H NMR: 400 MHz, $CDCl_3$

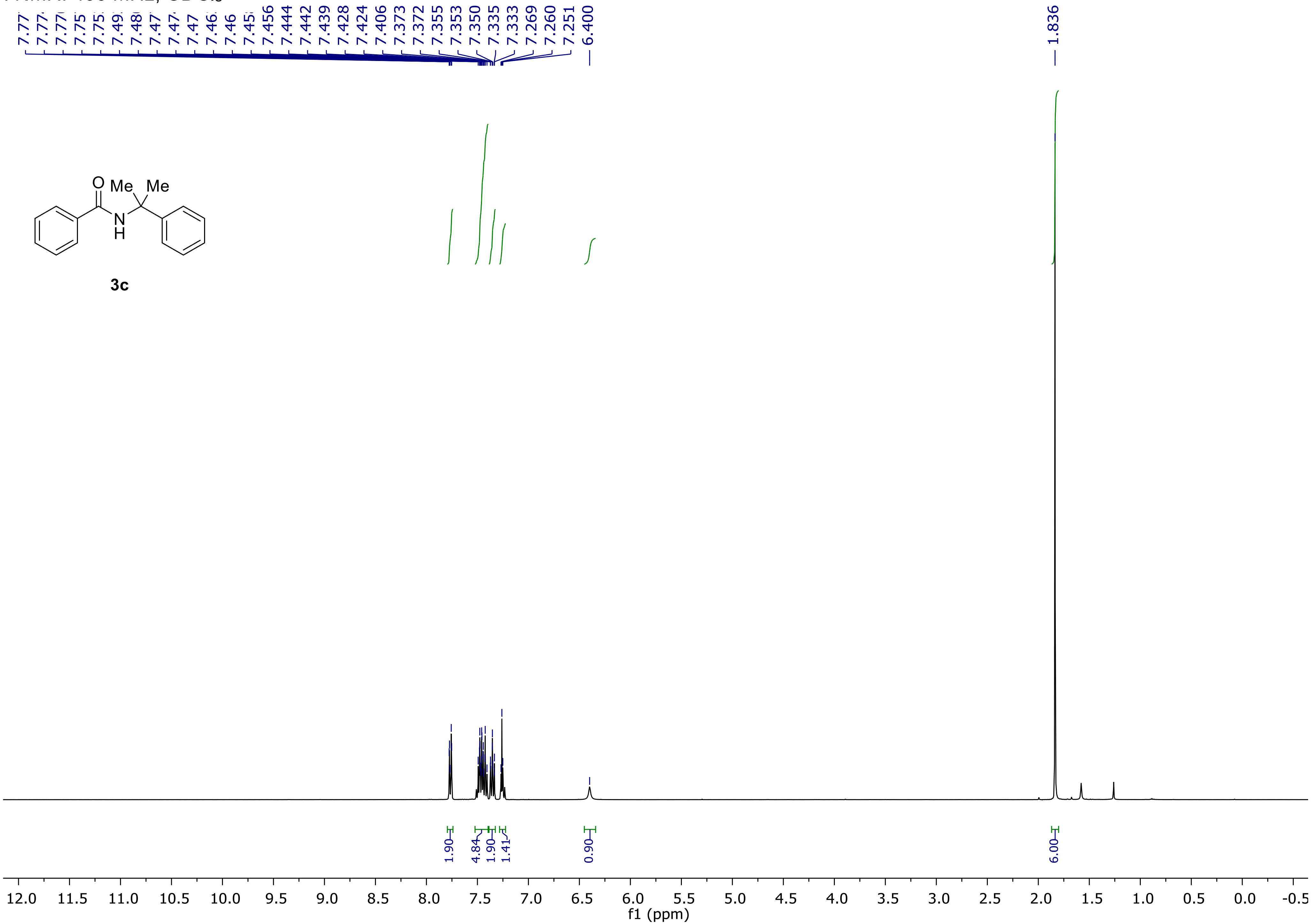

^{13}C NMR: 101 MHz, $CDCl_3$

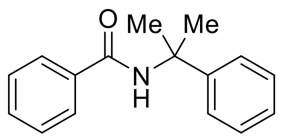

3c

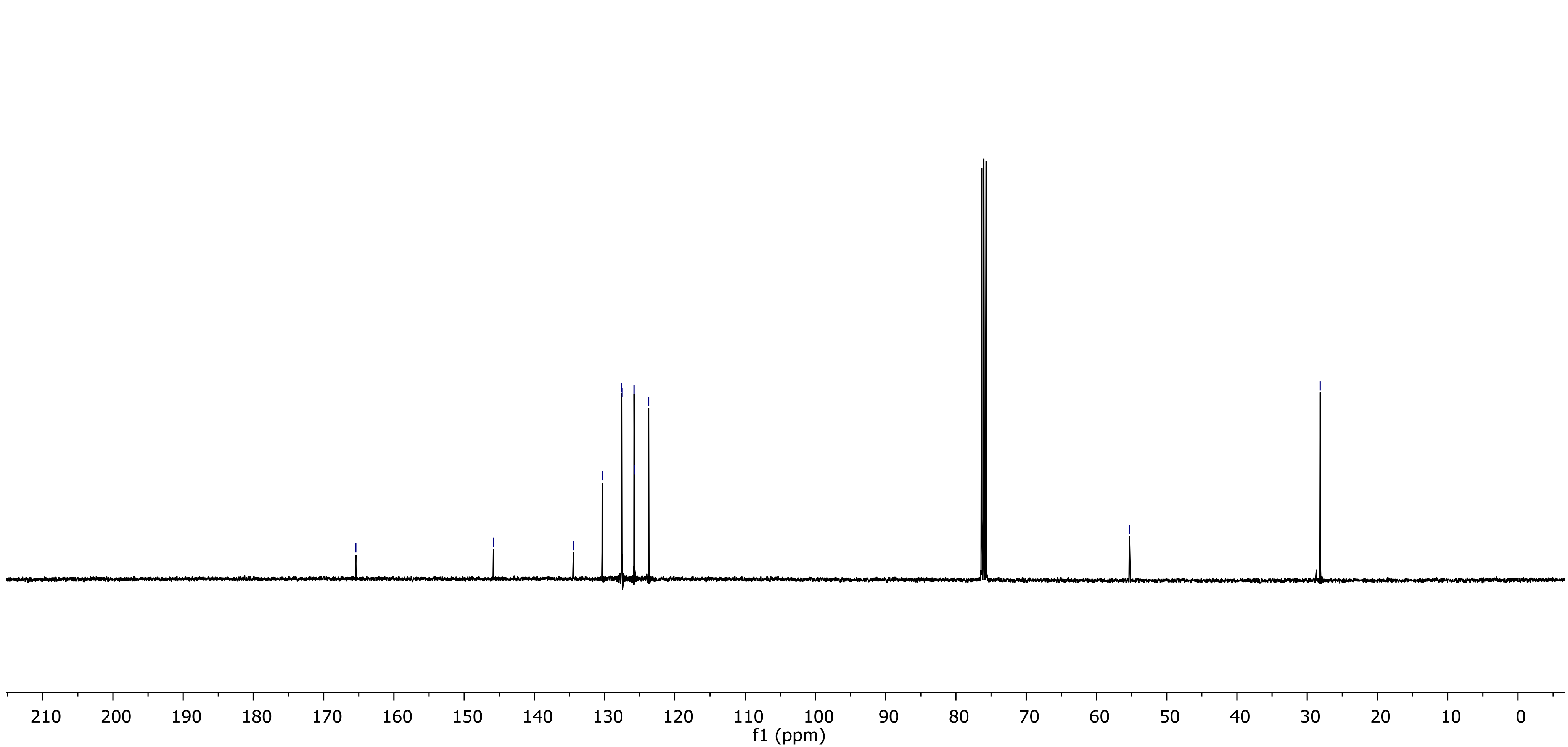

^1H NMR: 400 MHz, $CDCl_3$

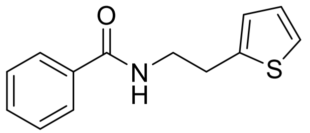

3d

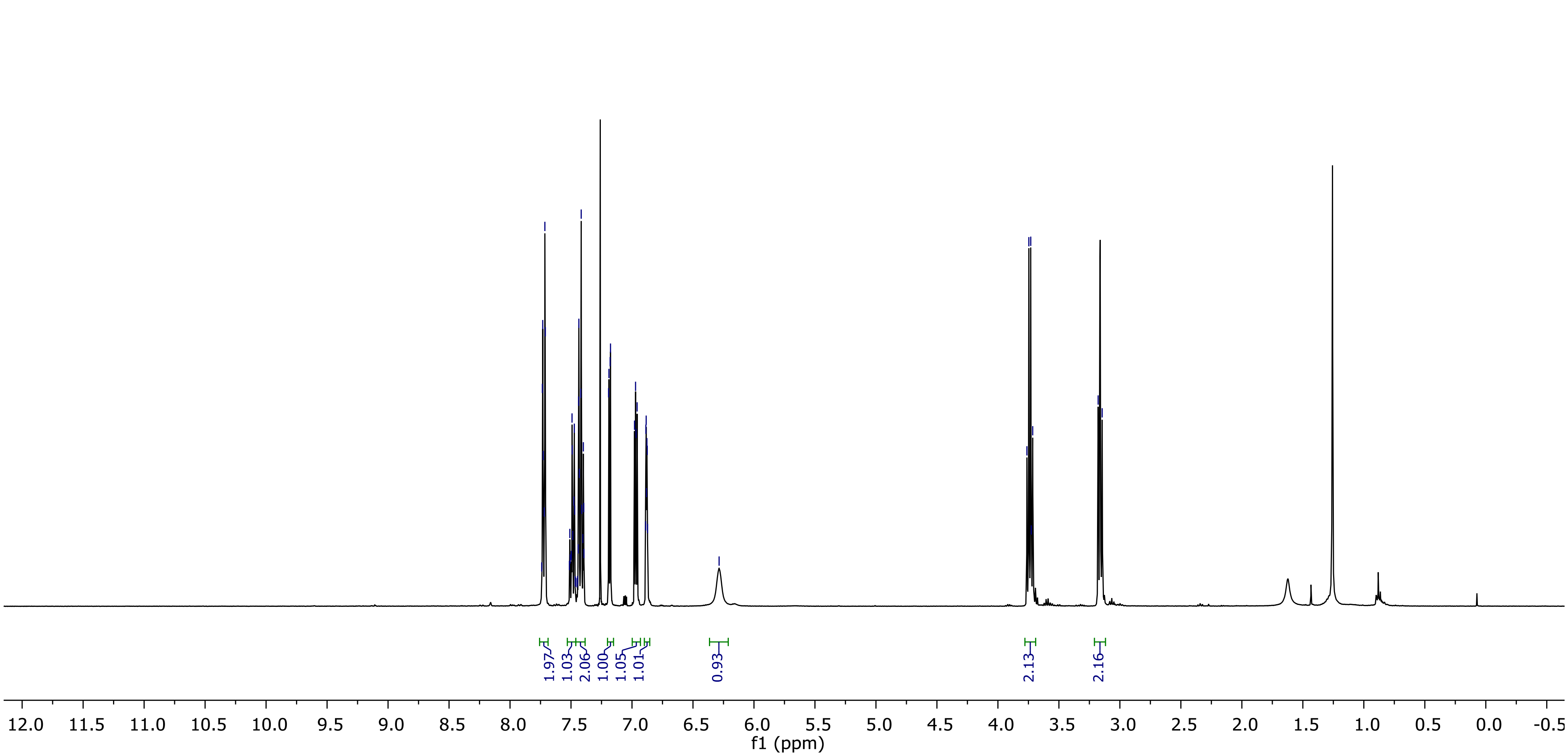

^{13}C NMR: 101 MHz, $CDCl_3$

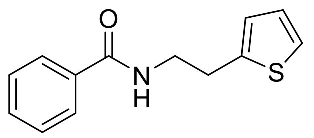

3d

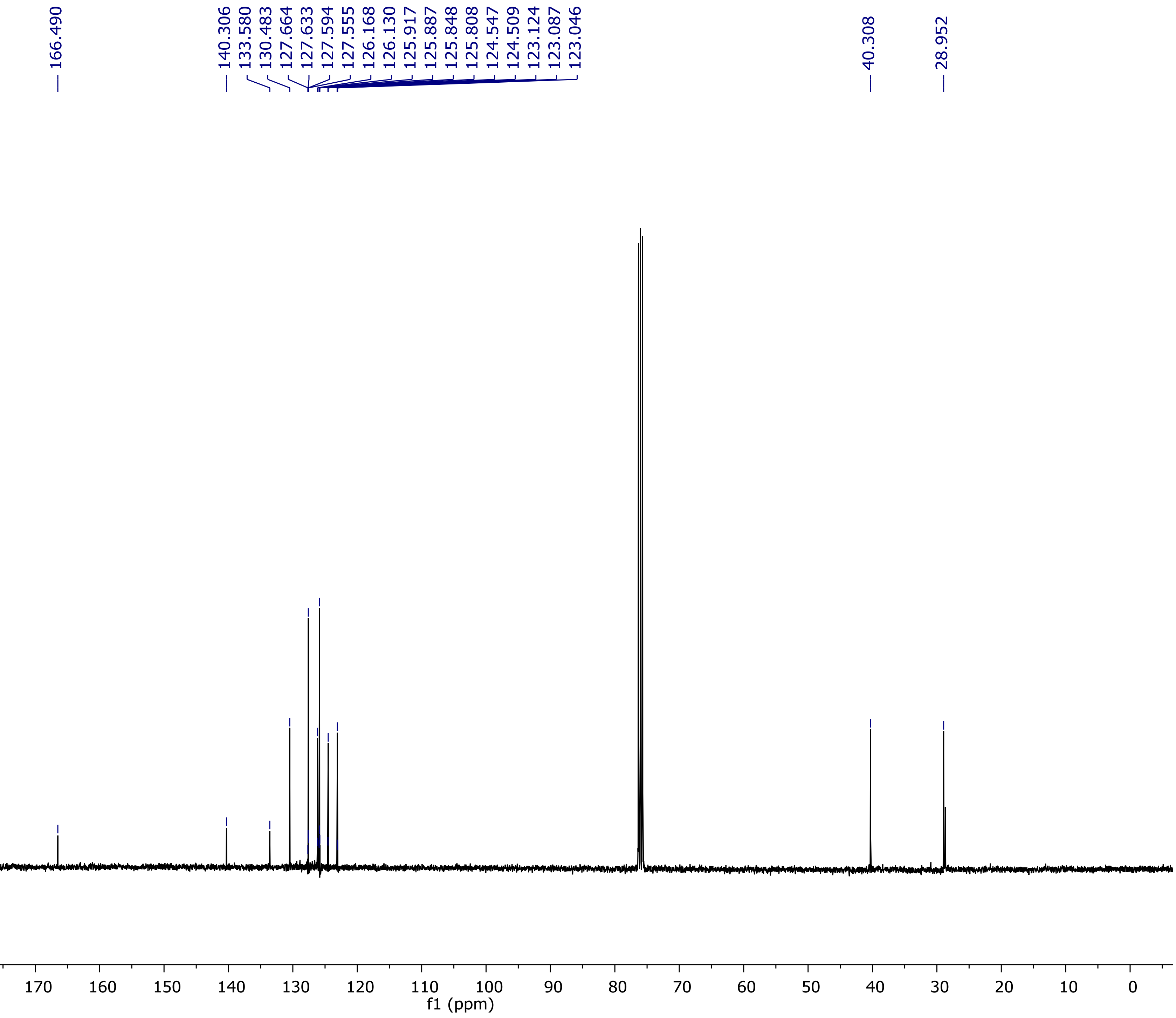

^{1}H NMR: 500 MHz, $CDCl_3$

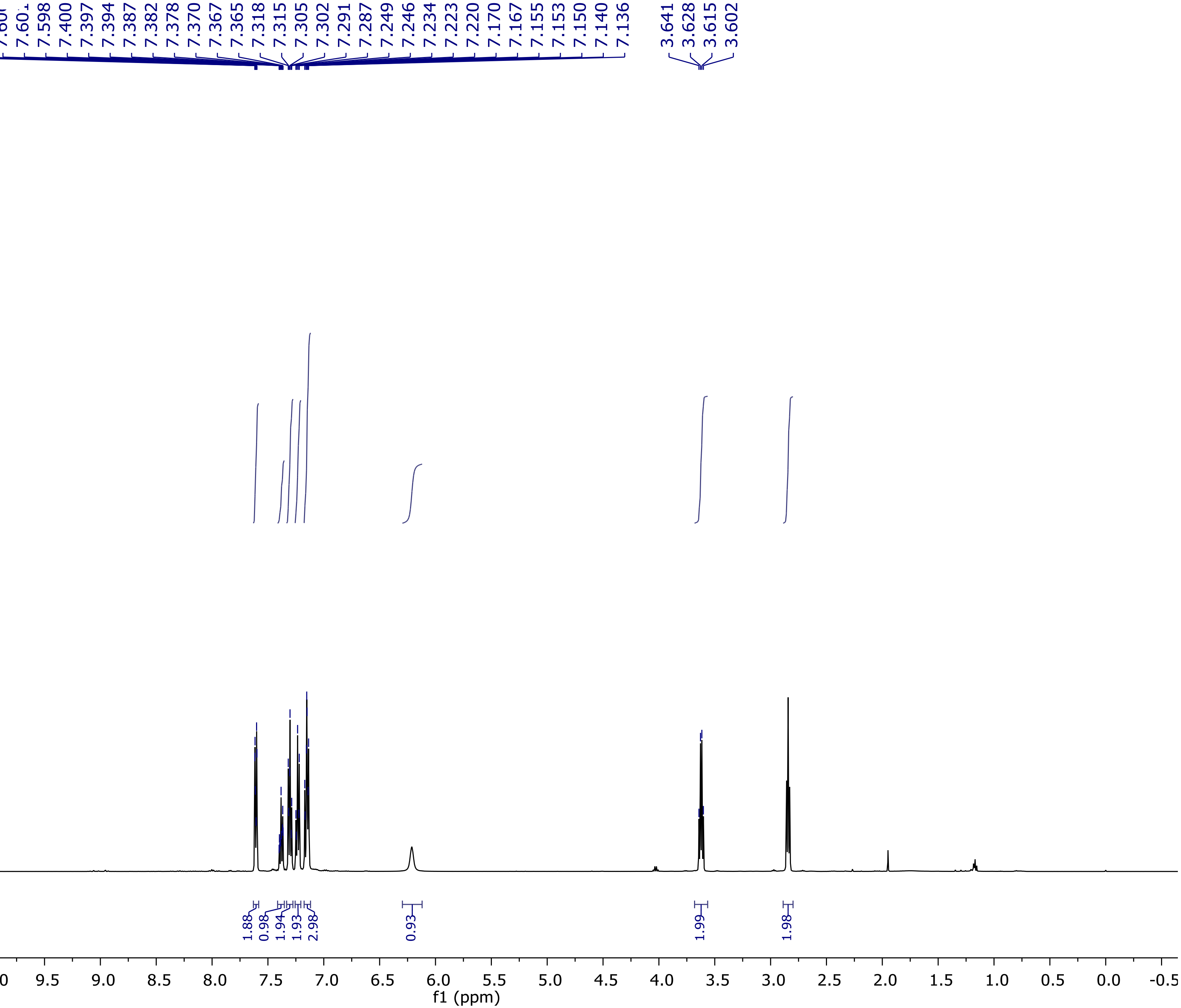

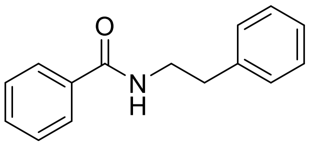

3e

^{13}C NMR: 126 MHz, $CDCl_3$

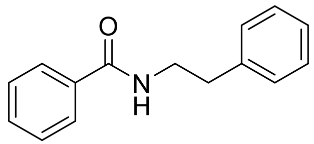

3e

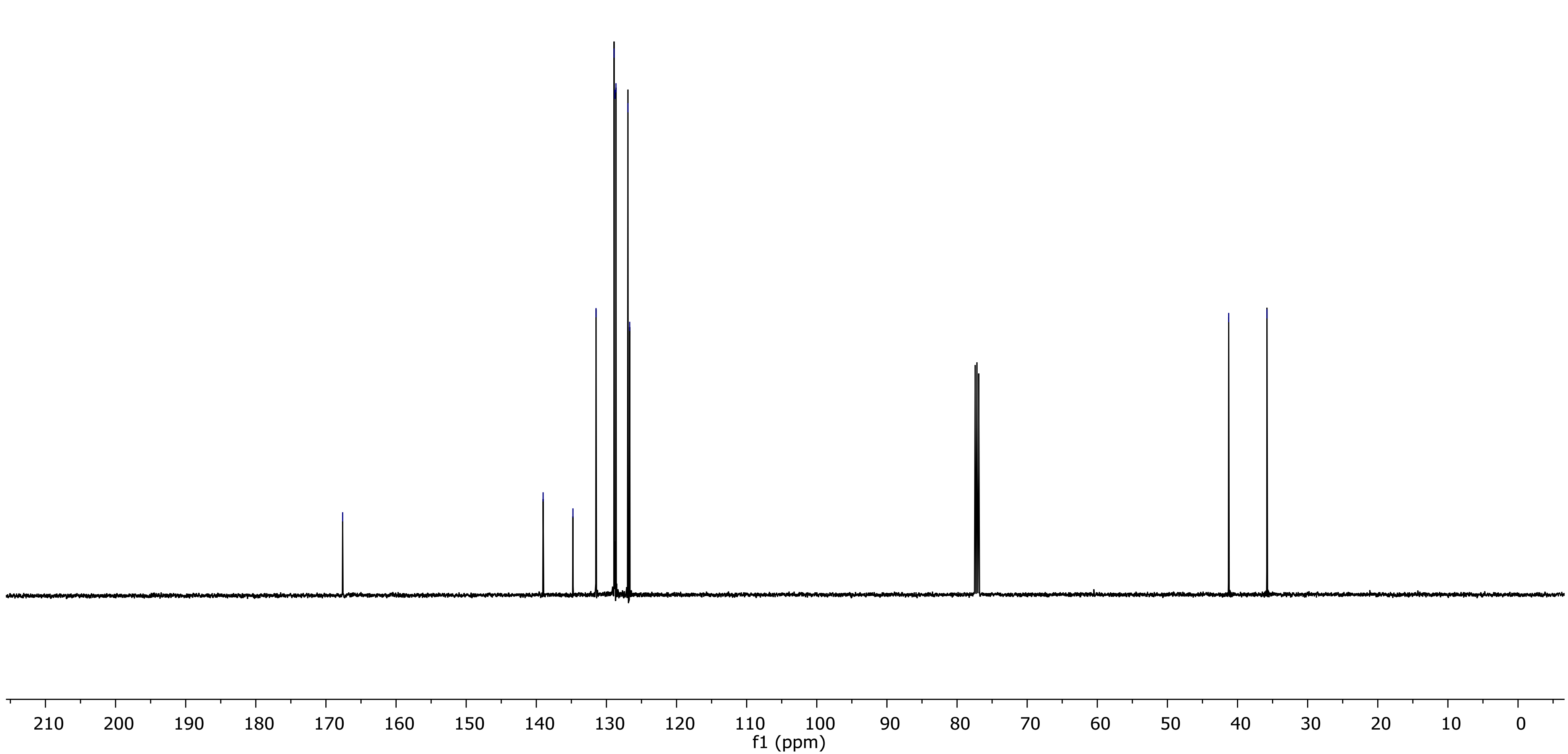

^{1}H NMR: 400 MHz, $CDCl_3$

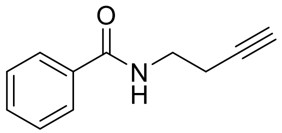

3f

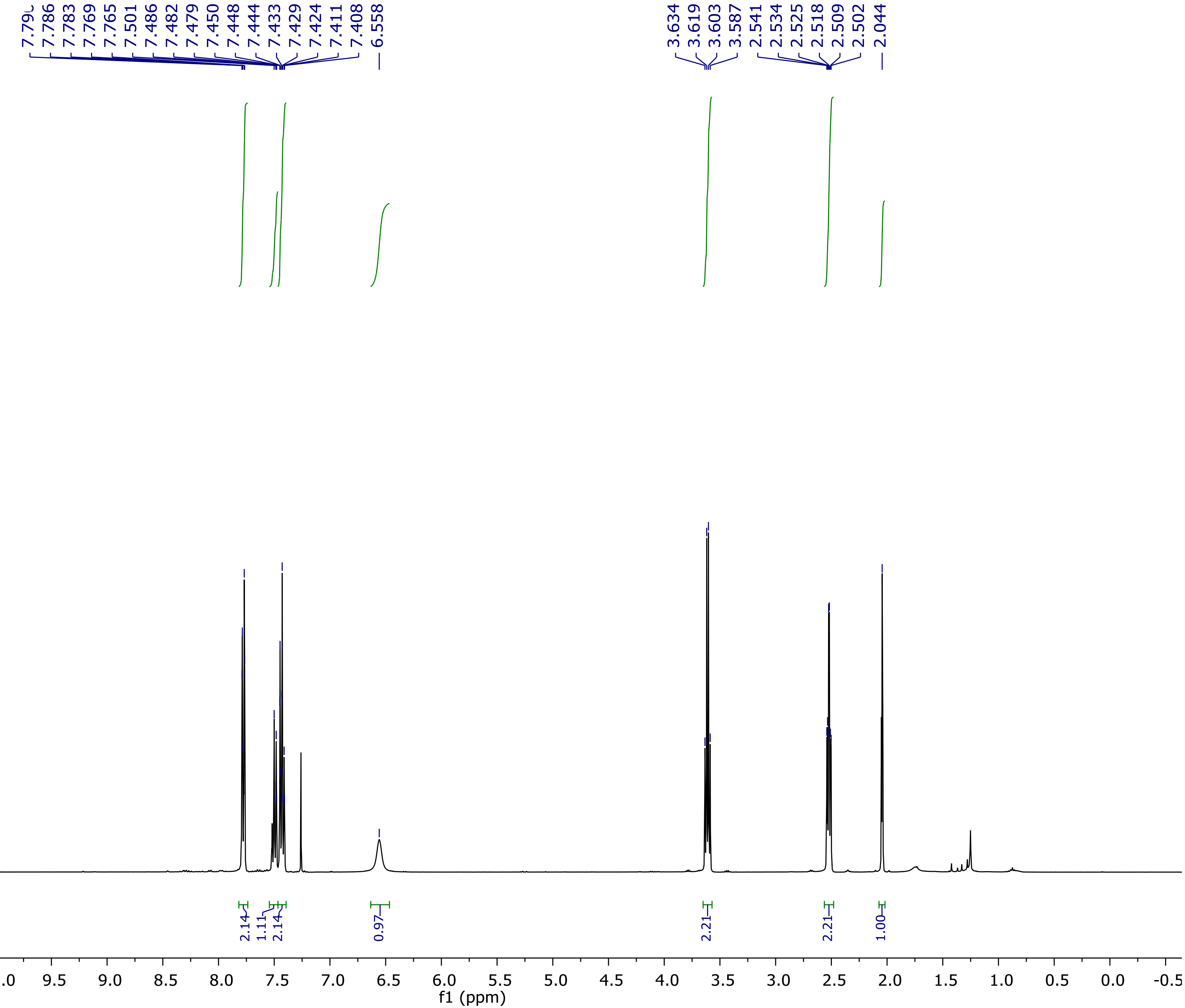

^{13}C NMR: 101 MHz, $CDCl_3$

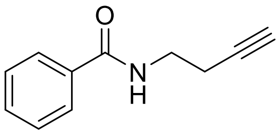

3f

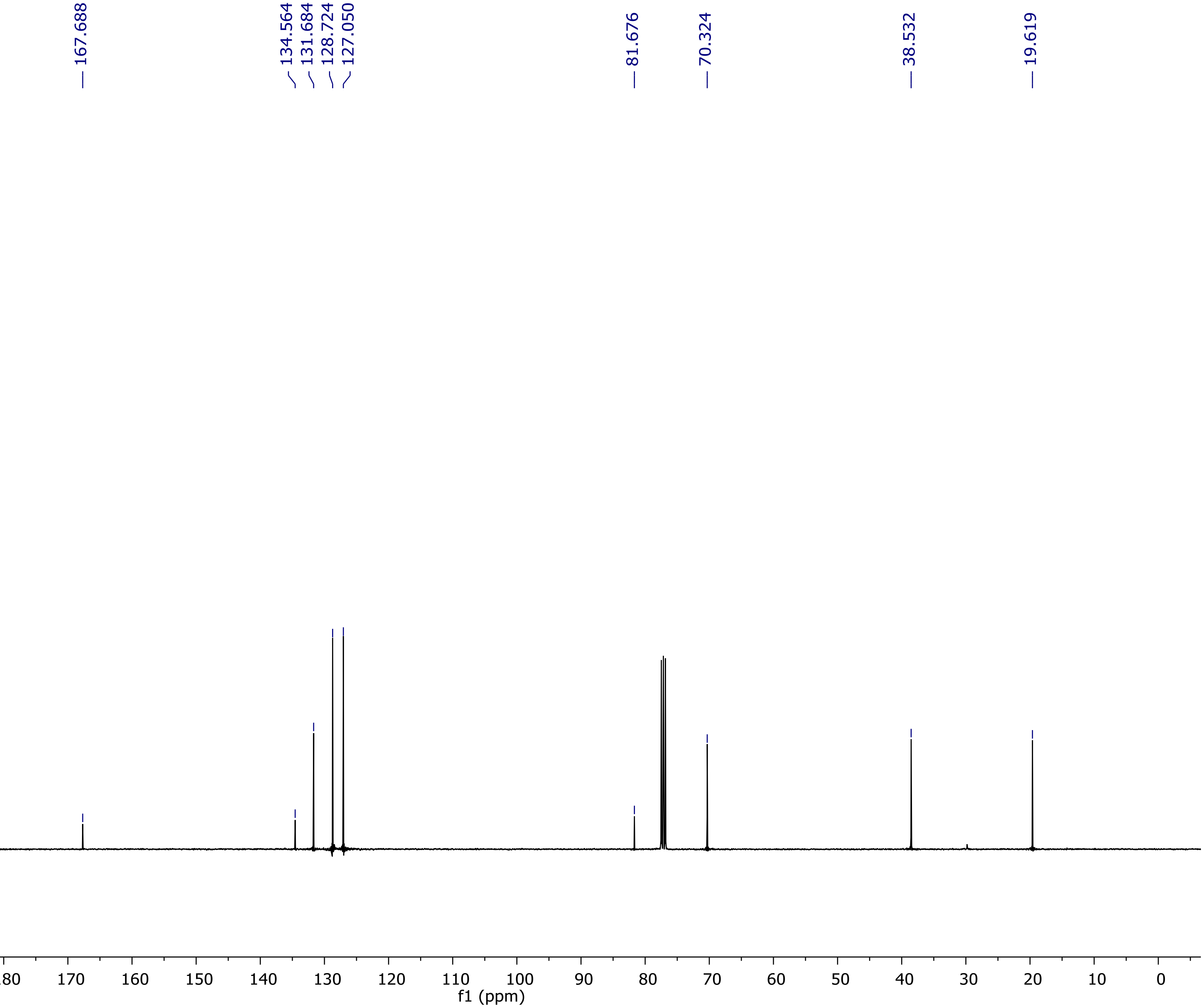

^1H NMR: 400 MHz, $CDCl_3$

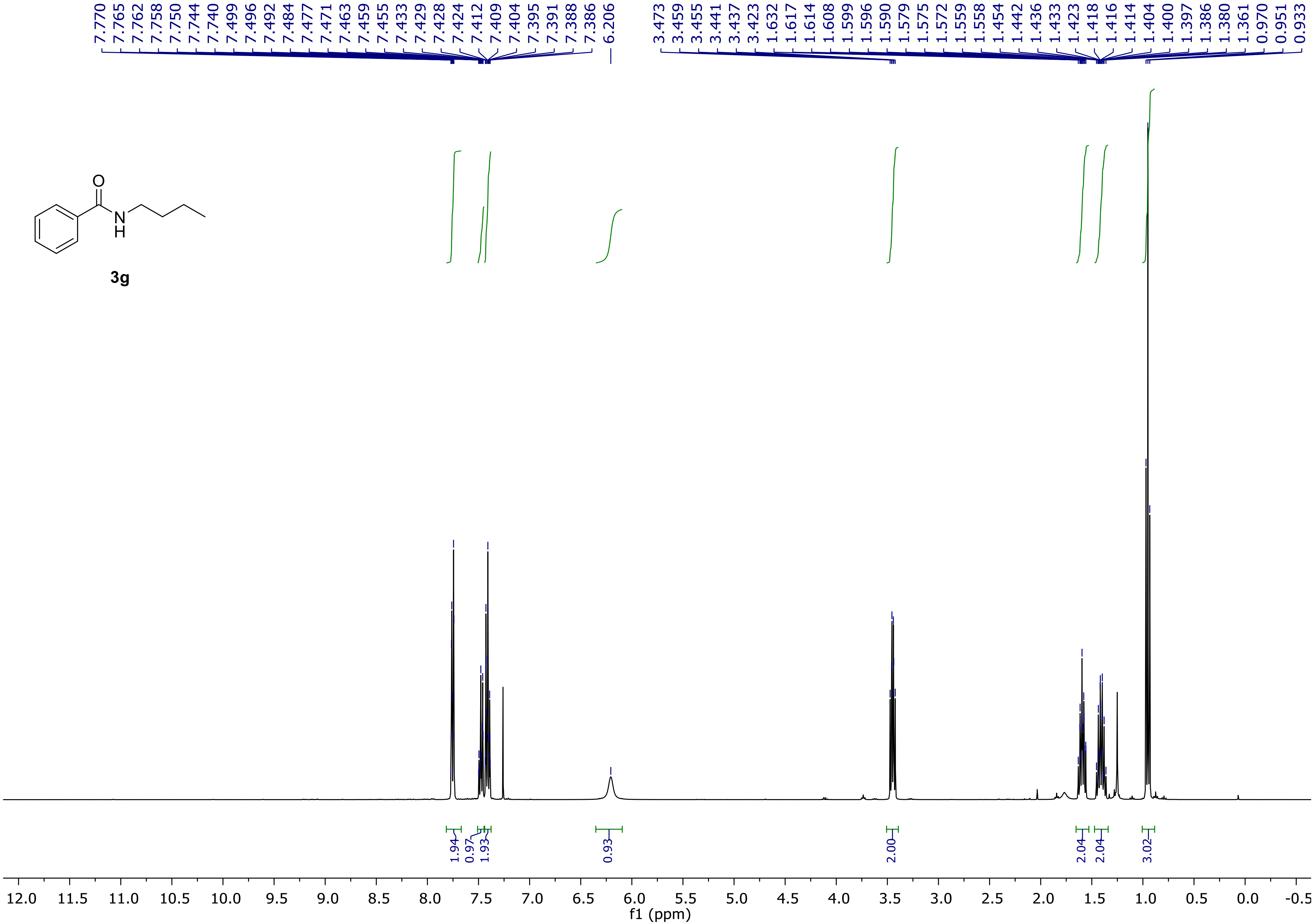

^{13}C NMR: 101 MHz, $CDCl_3$

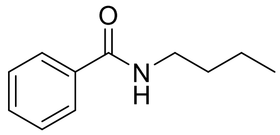

3g

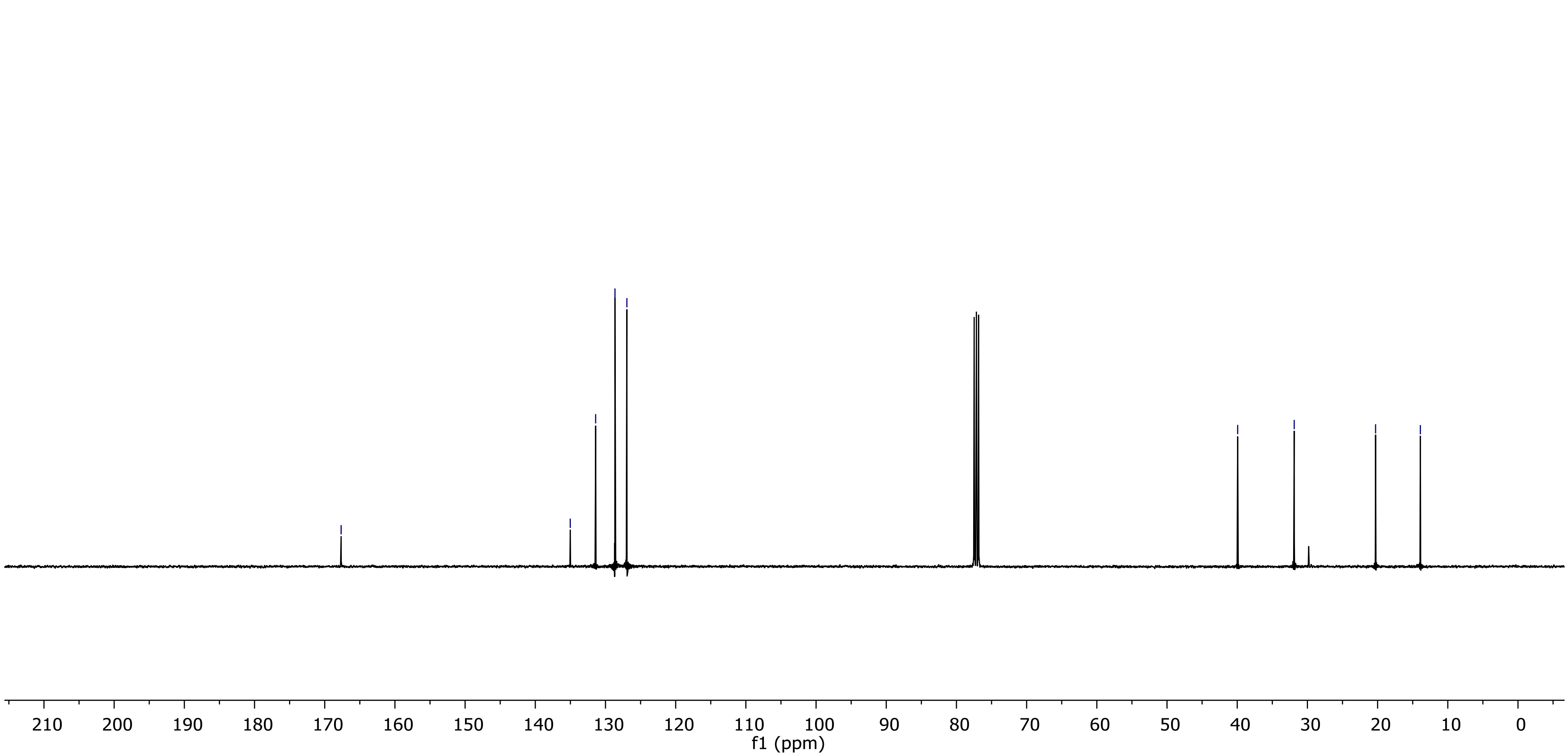

^1H NMR: 400 MHz, $CDCl_3$

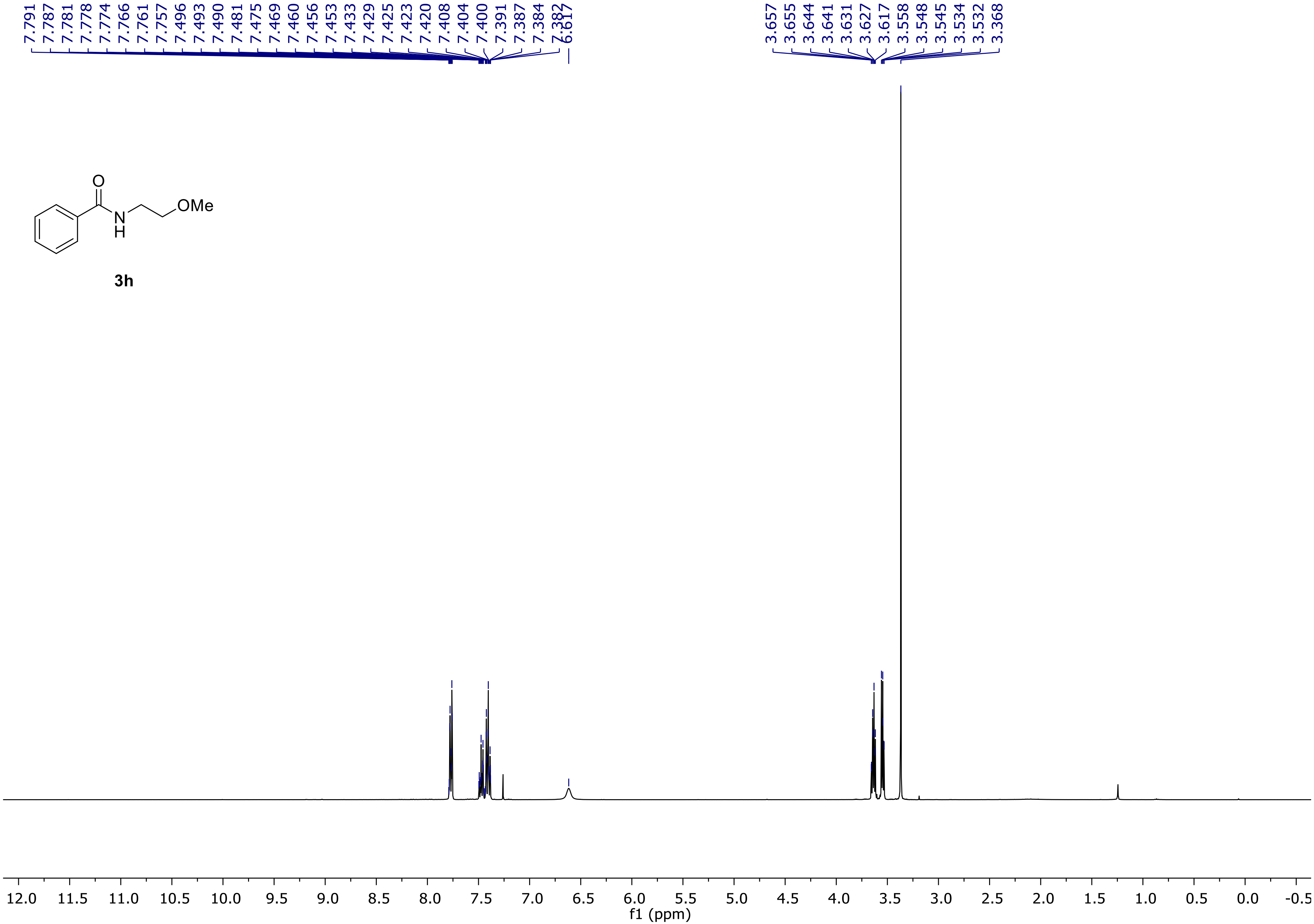

3h

^{13}C NMR: 101 MHz, $CDCl_3$

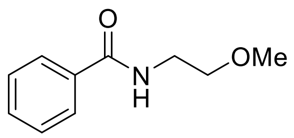

3h

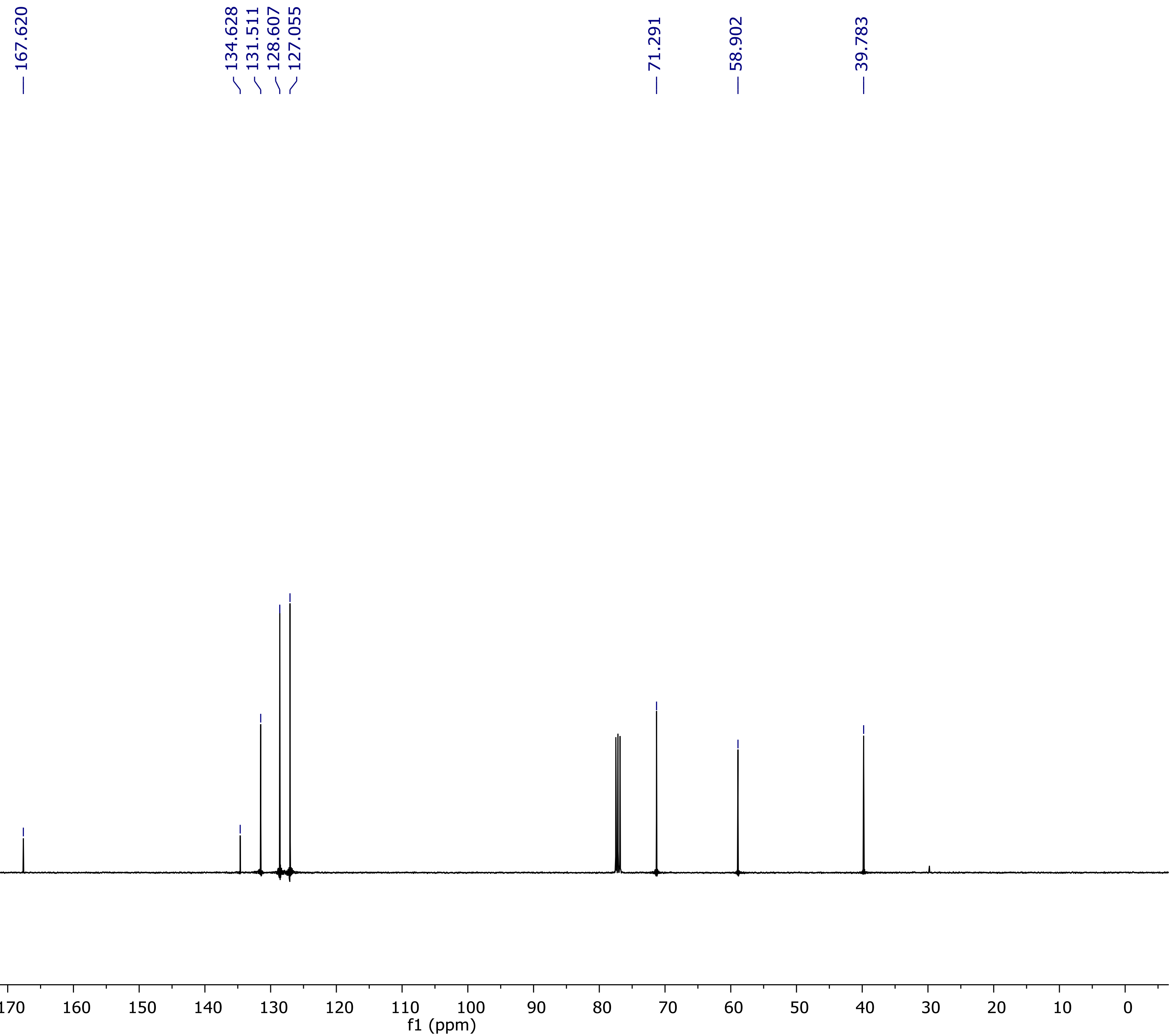

^1H NMR: 500 MHz, $CDCl_3$

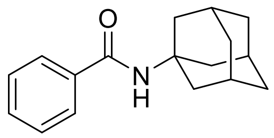

3i

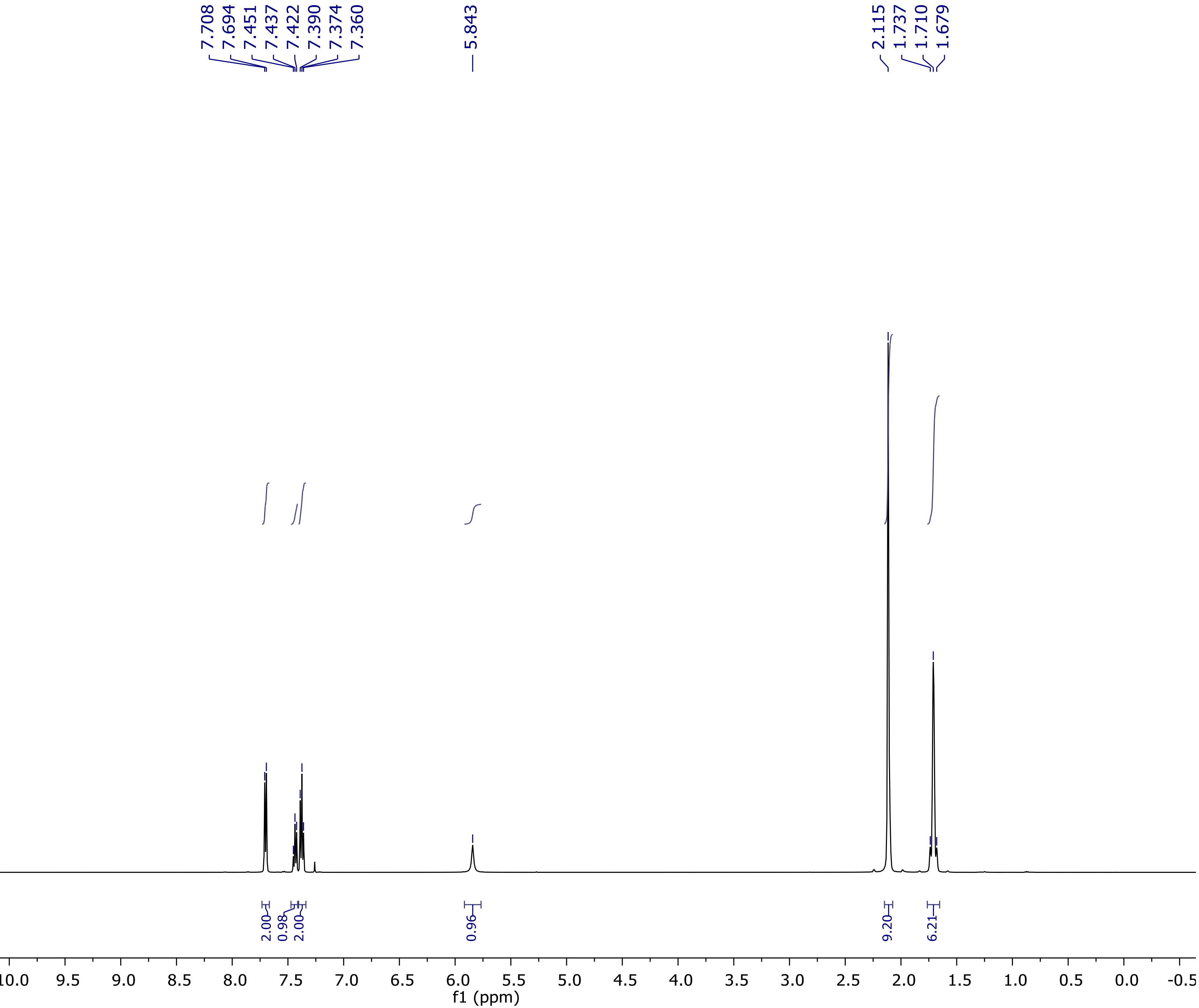

^{13}C NMR: 126 MHz, $CDCl_3$

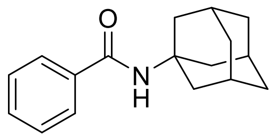

3i

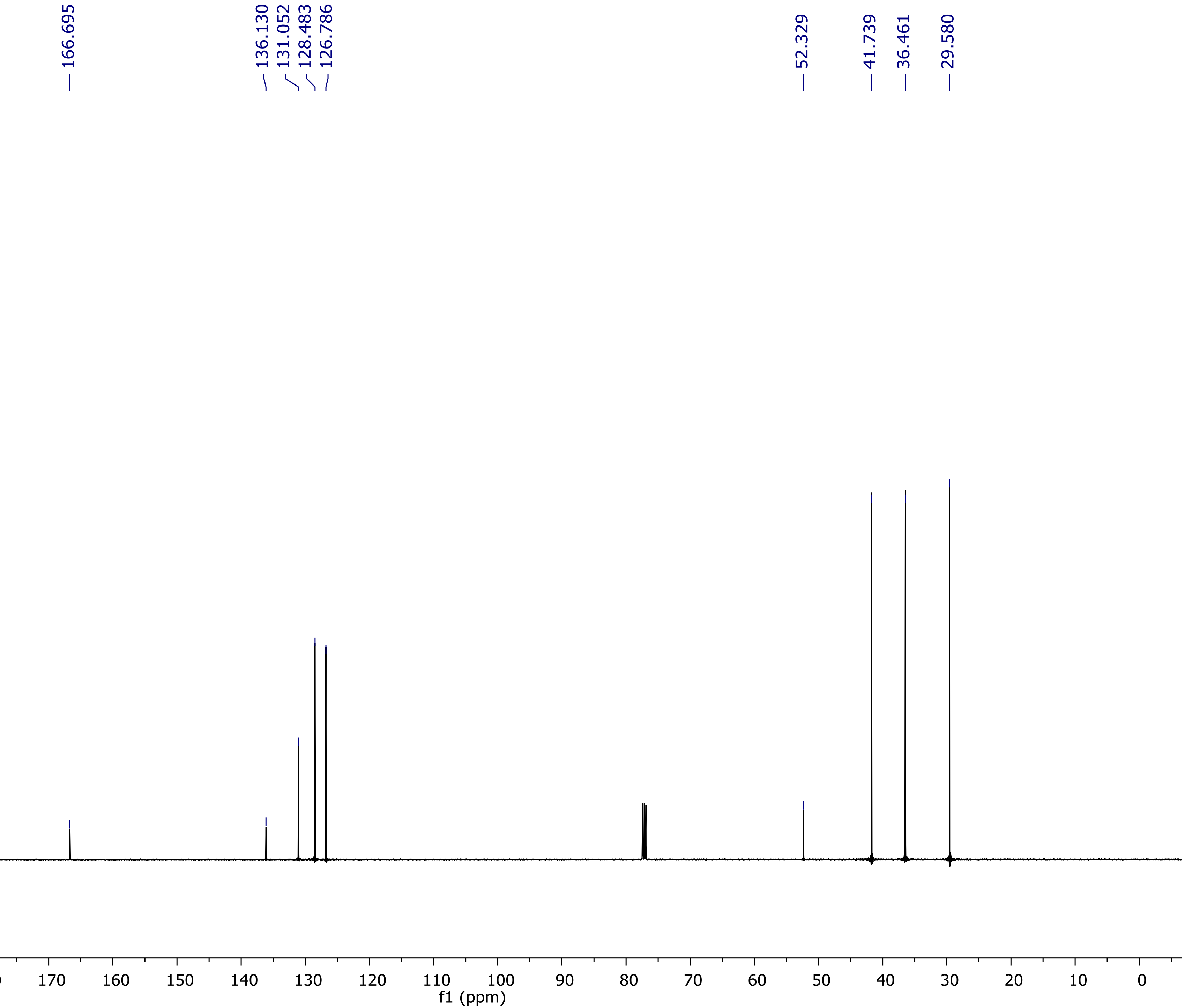

^1H NMR: 500 MHz, D_6-DMSO

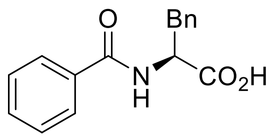

3j

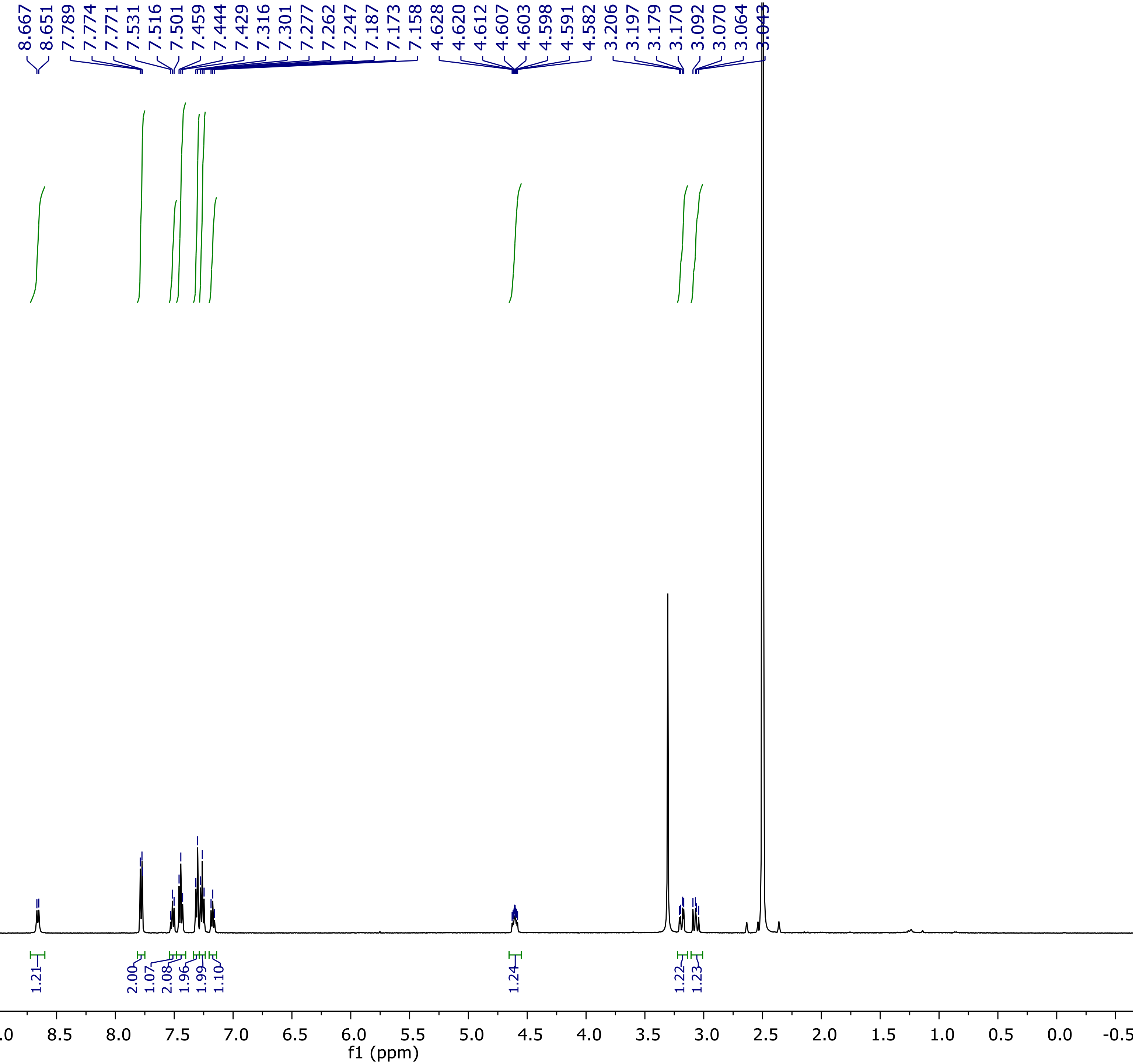

^{13}C NMR: 126 MHz,
D_6-DMSO

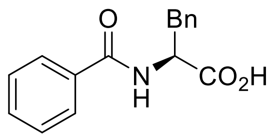

3j

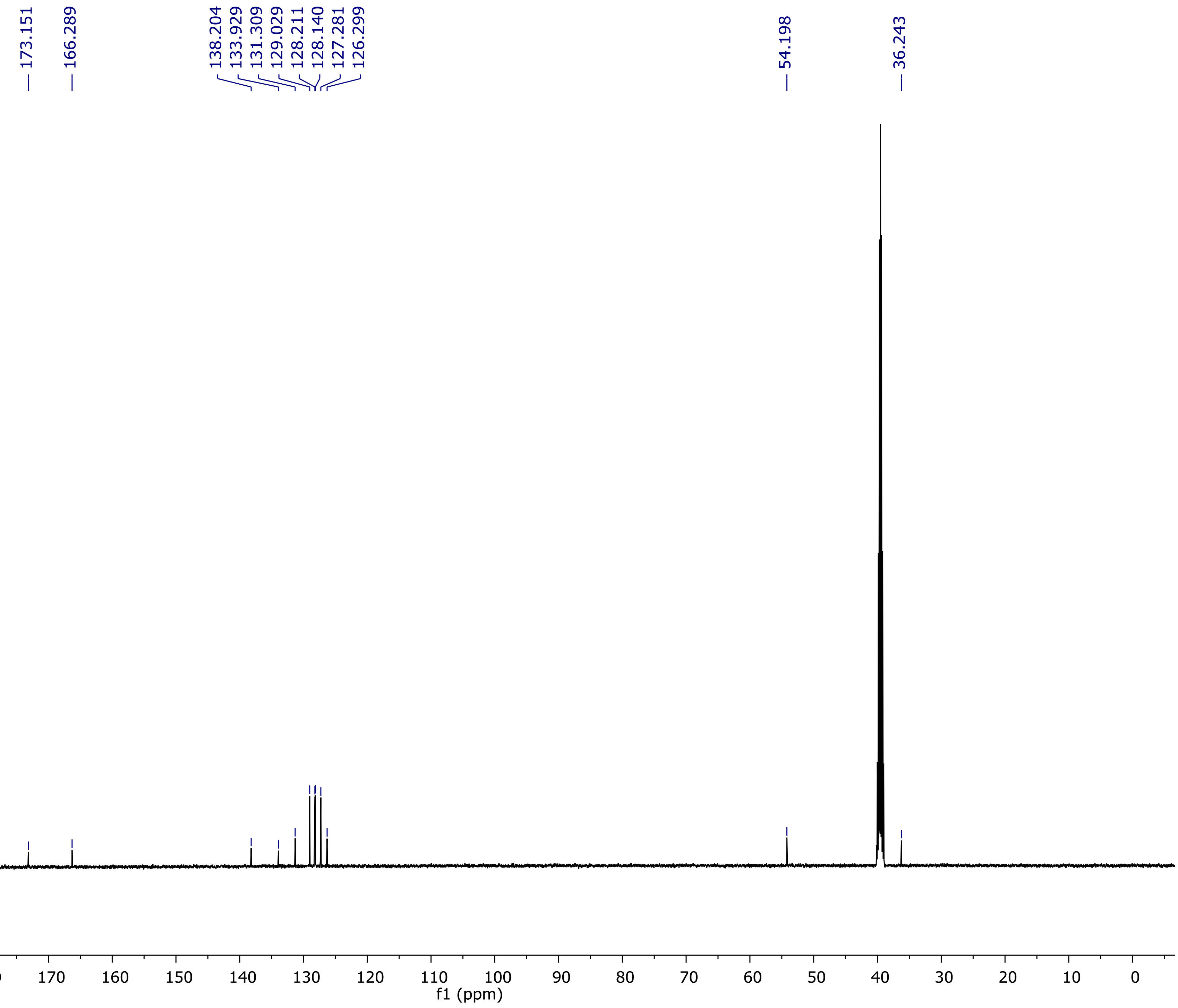

^{1}H NMR: 400 MHz, $CDCl_3$

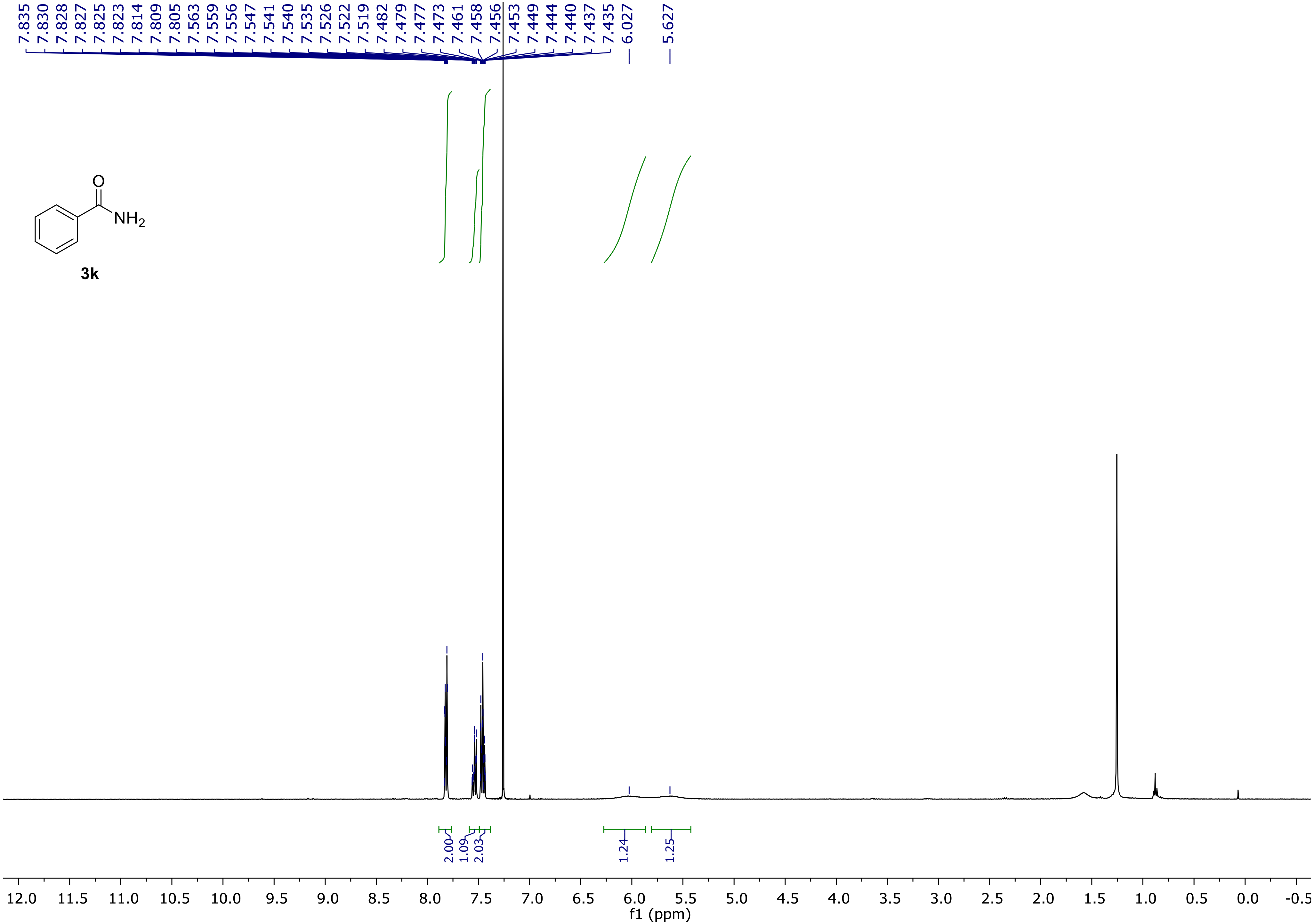

^{13}C NMR: 101 MHz, $CDCl_3$

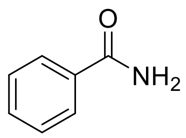

3k

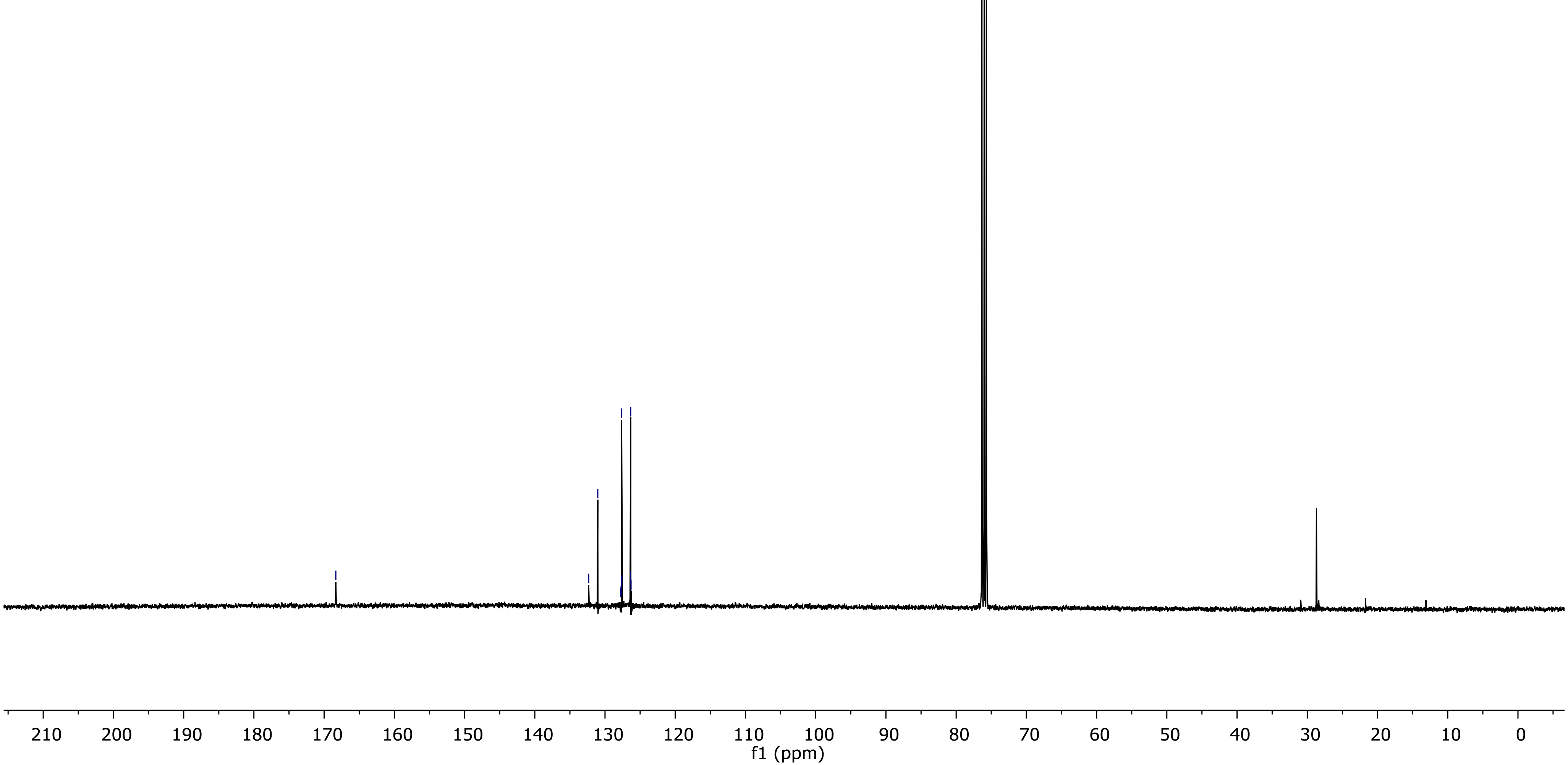

^{1}H NMR: 500 MHz, D_6-DMSO

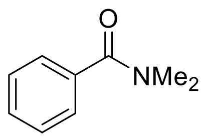

3l

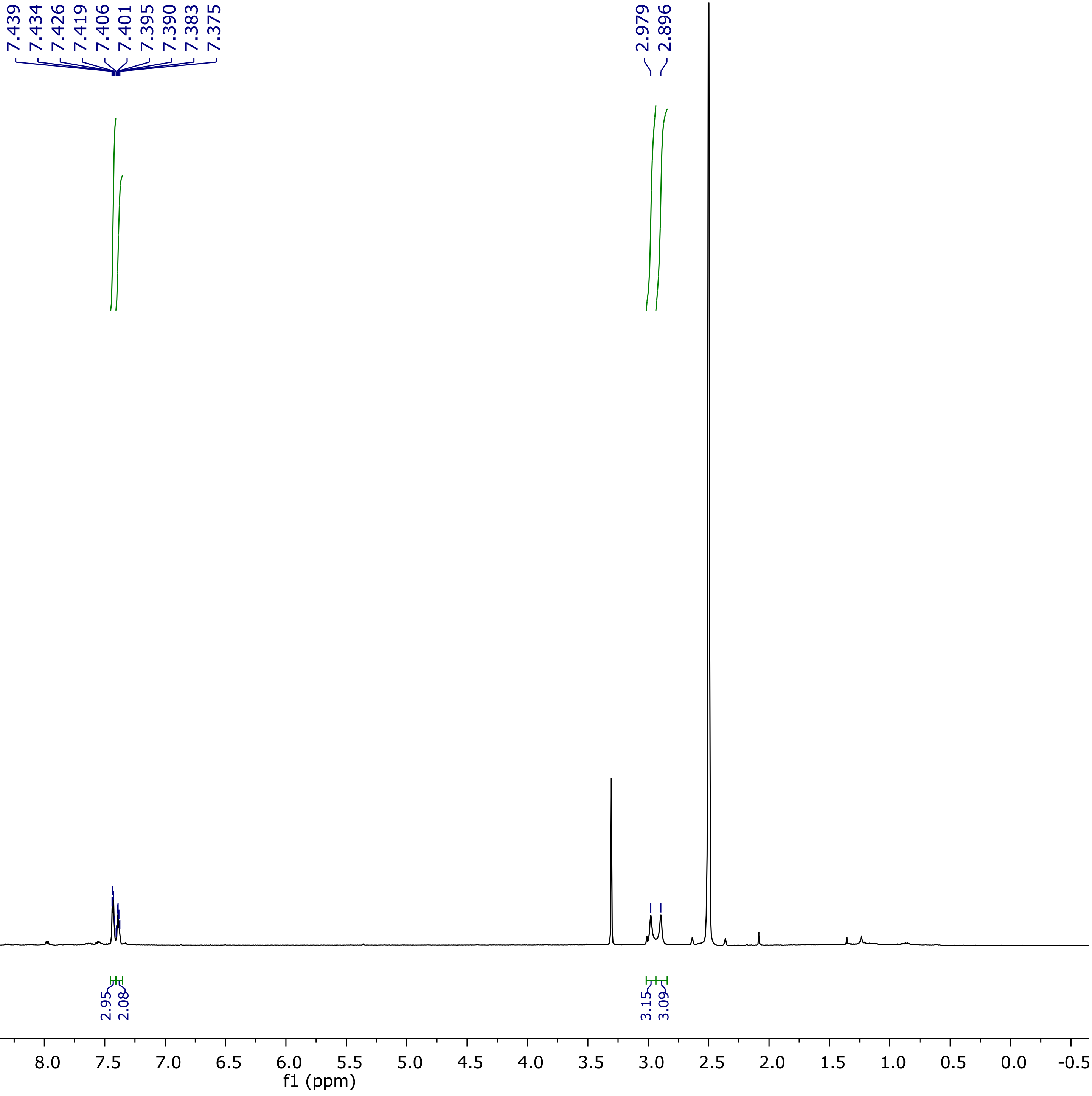

^{13}C NMR: 126 MHz, D_6-DMSO

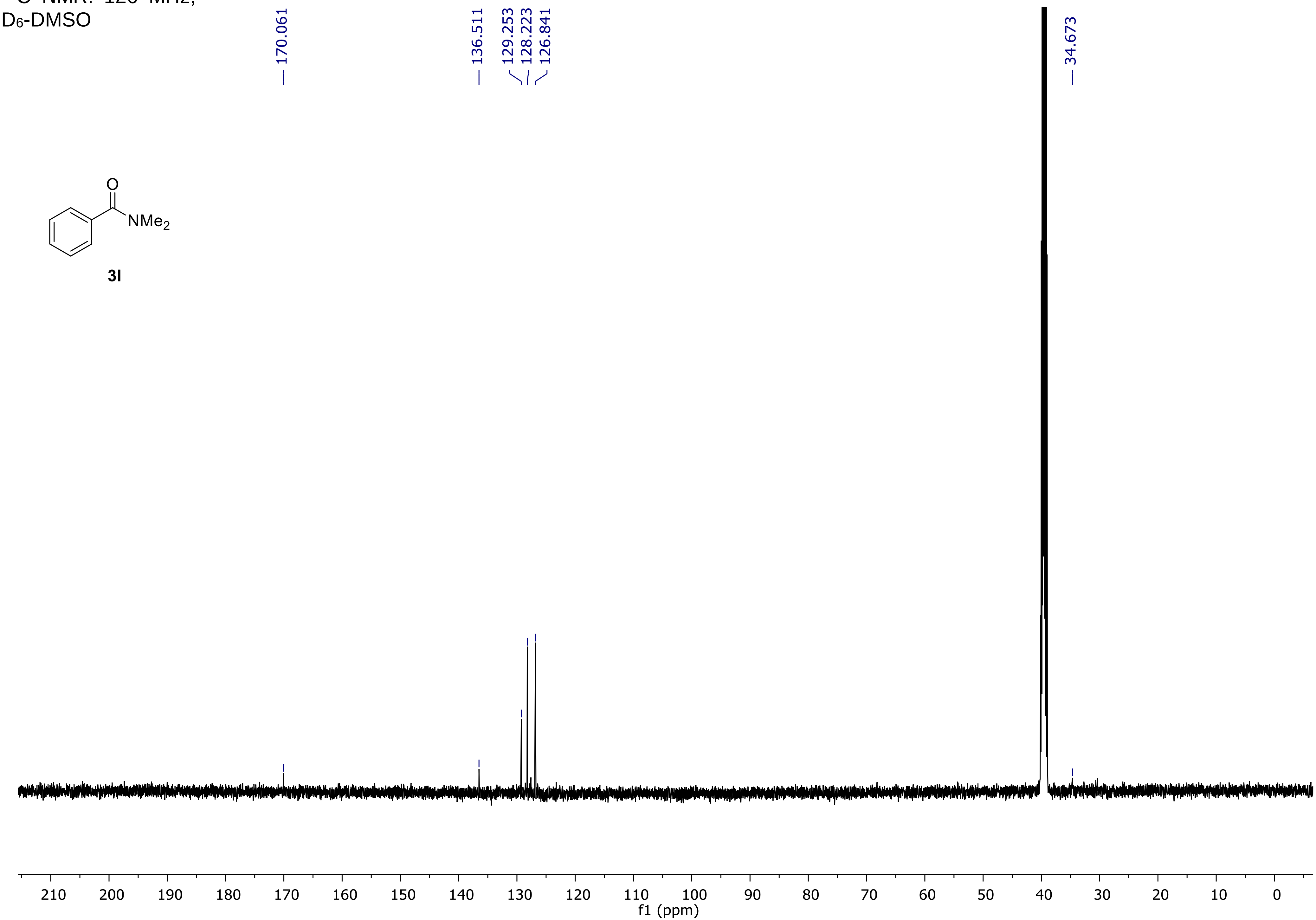

^{1}H NMR: 500 MHz, $CDCl_3$

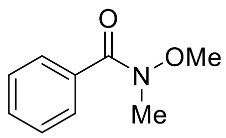

3m

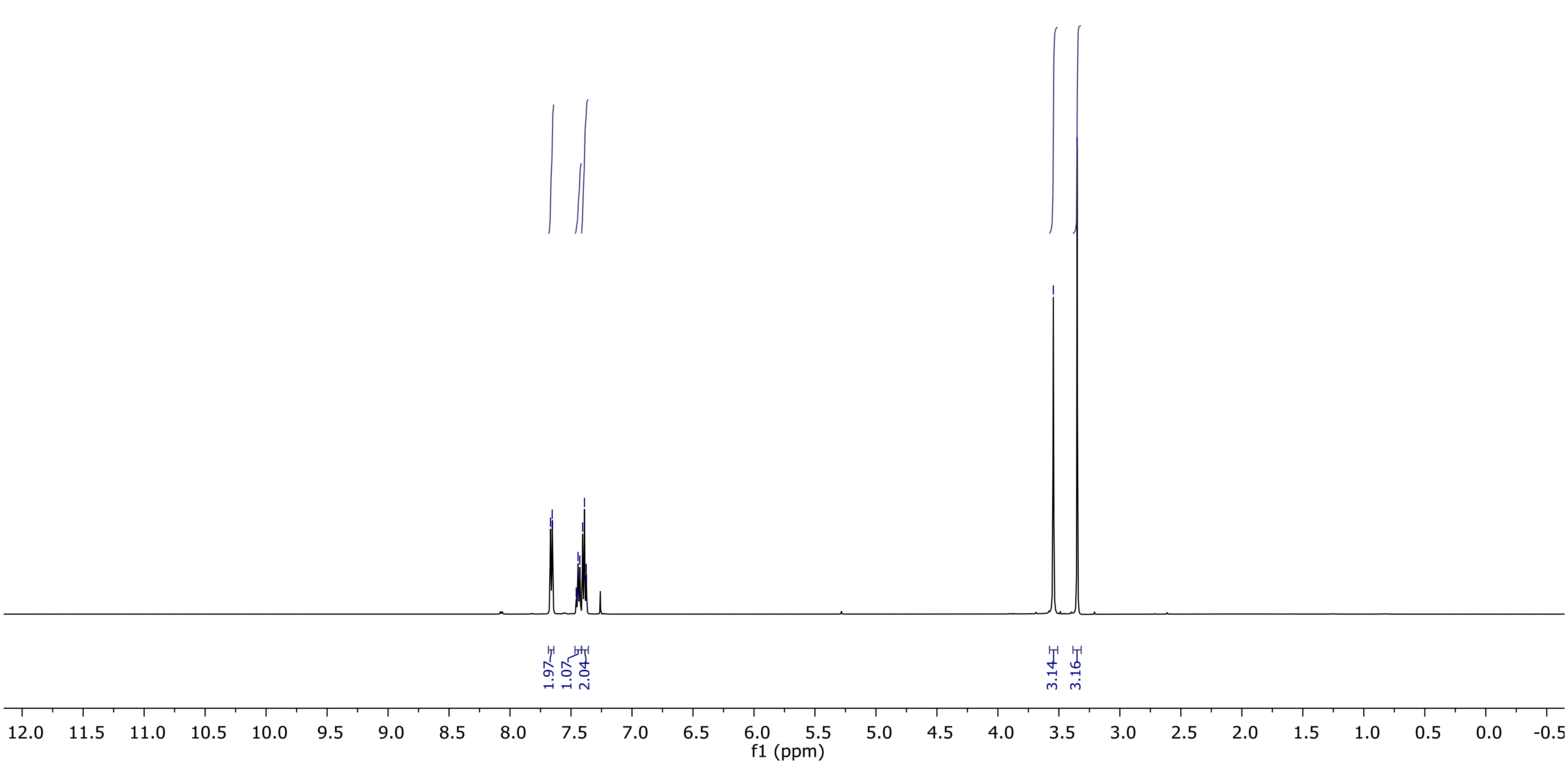

^{13}C NMR: 126 MHz, $CDCl_3$

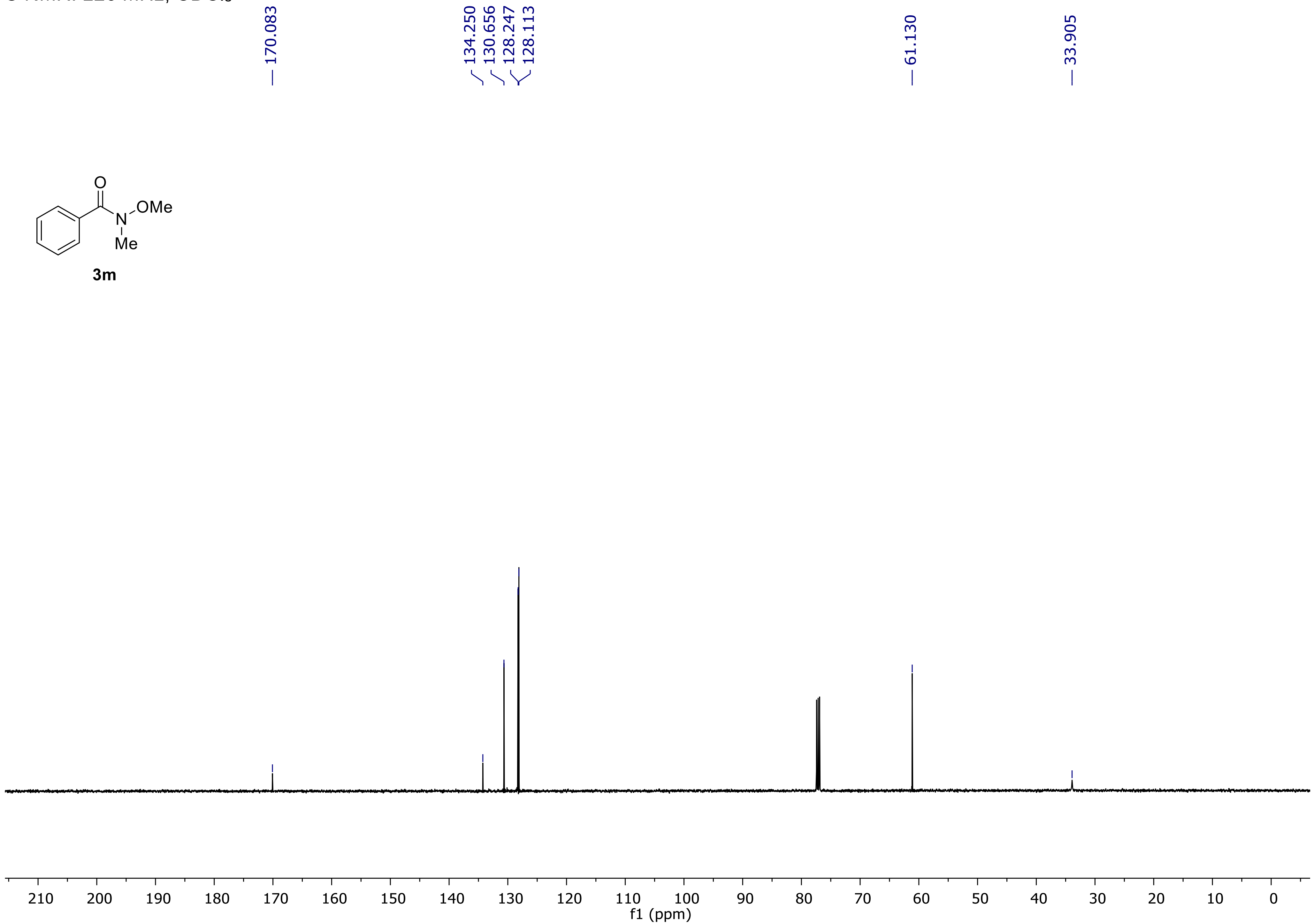

^1H NMR: 500 MHz, D_6-DMSO, 100 °C

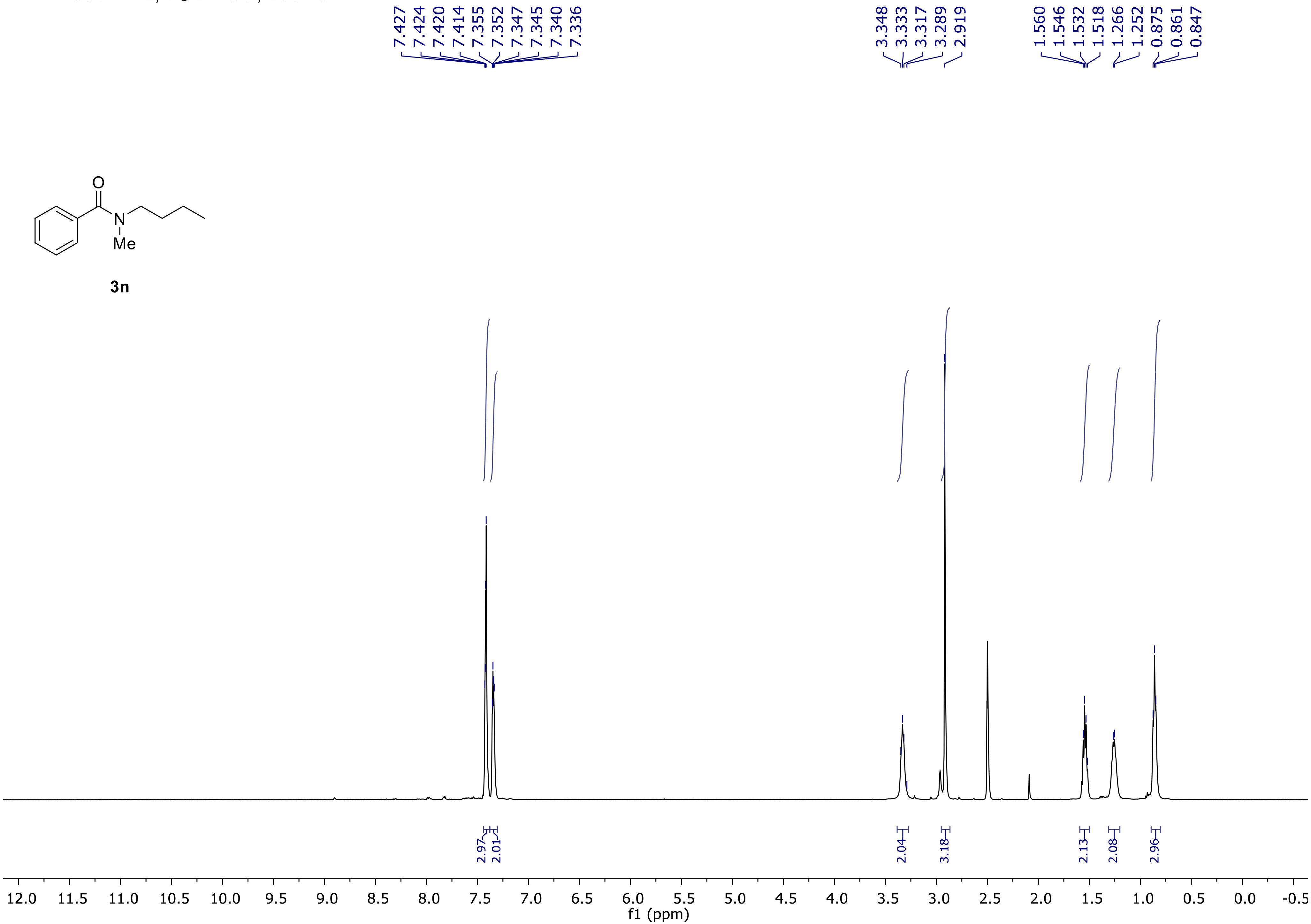

3n

^{13}C NMR: 126 MHz,
D_6-DMSO, 100 °C

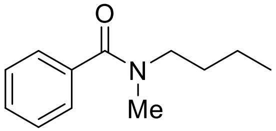

3n

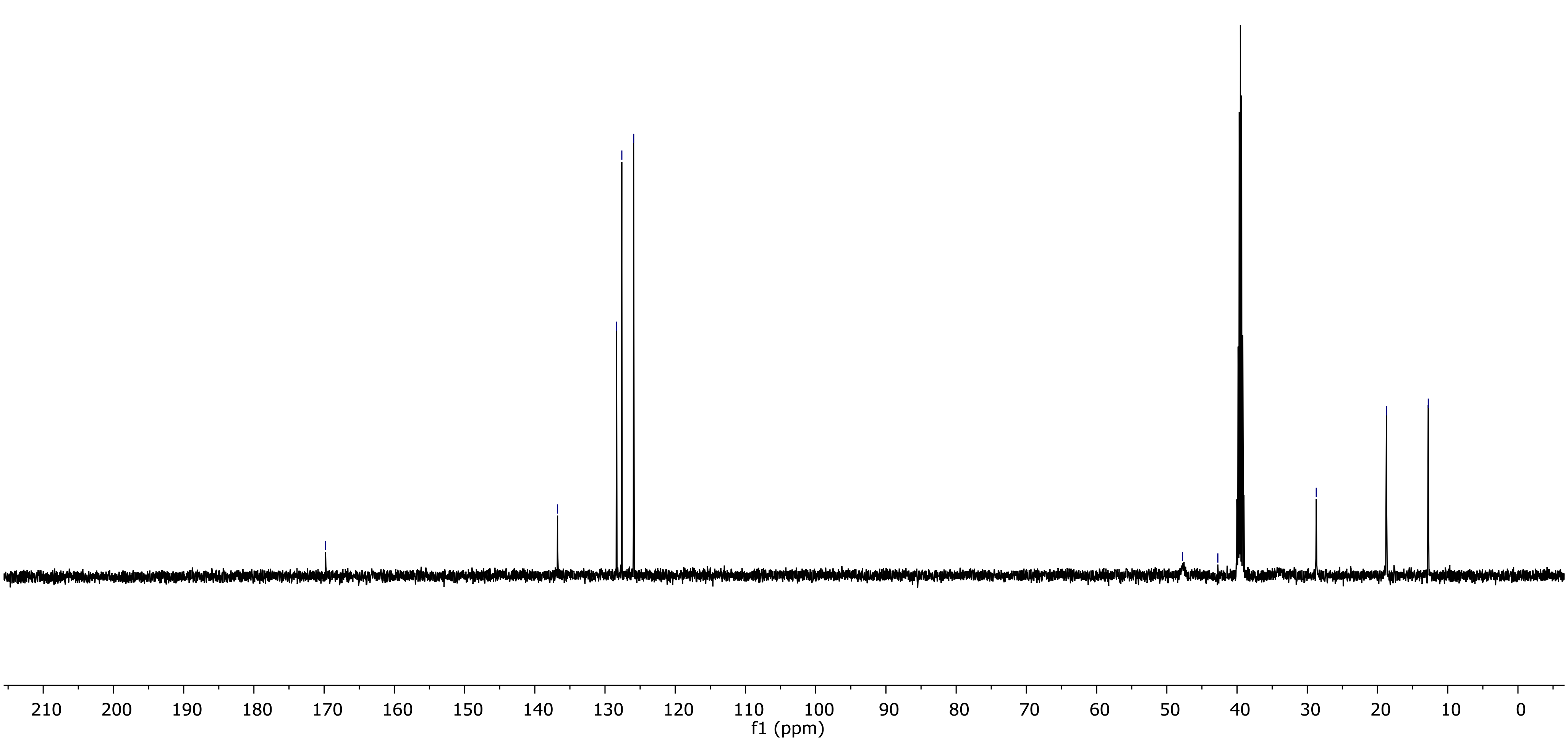

^{1}H NMR: 500 MHz, D_6-DMSO, 100 °C

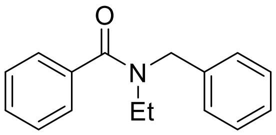

3o

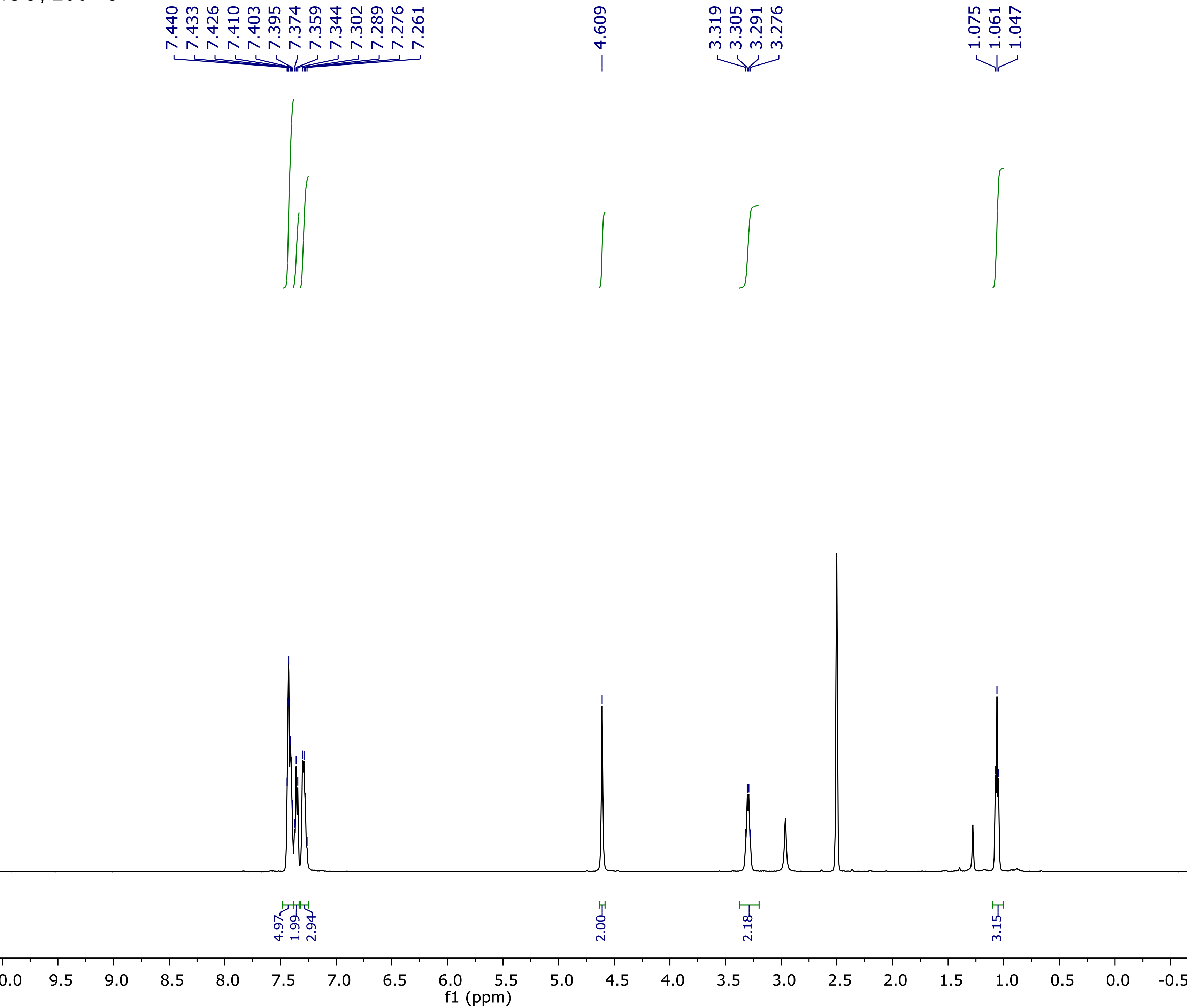

^{13}C NMR: 126 MHz,
D_6-DMSO, 100 °C

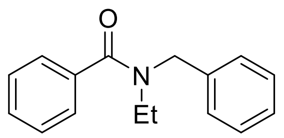

3o

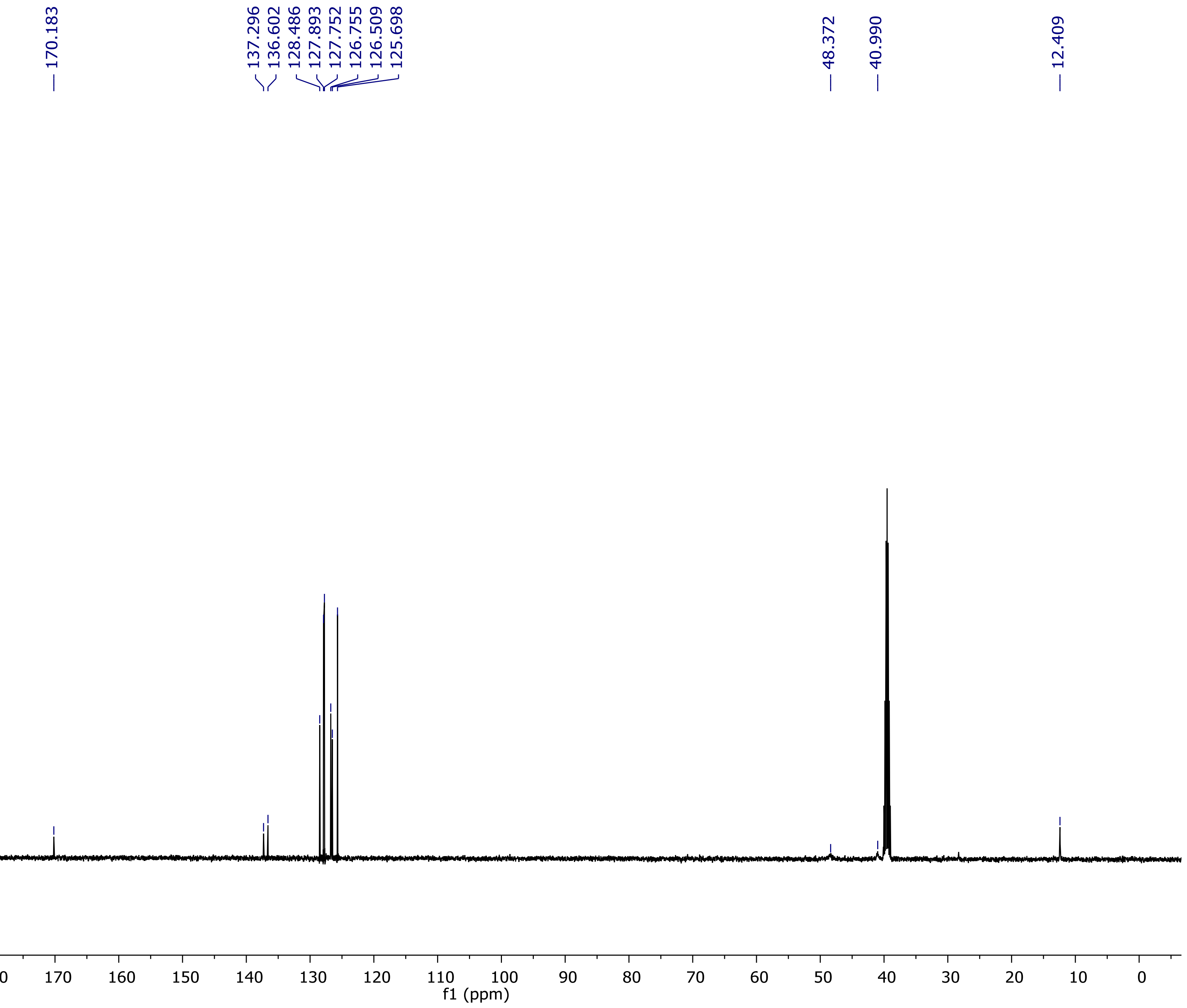

^{1}H NMR: 500 MHz, D6-DMSO

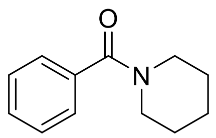

3p

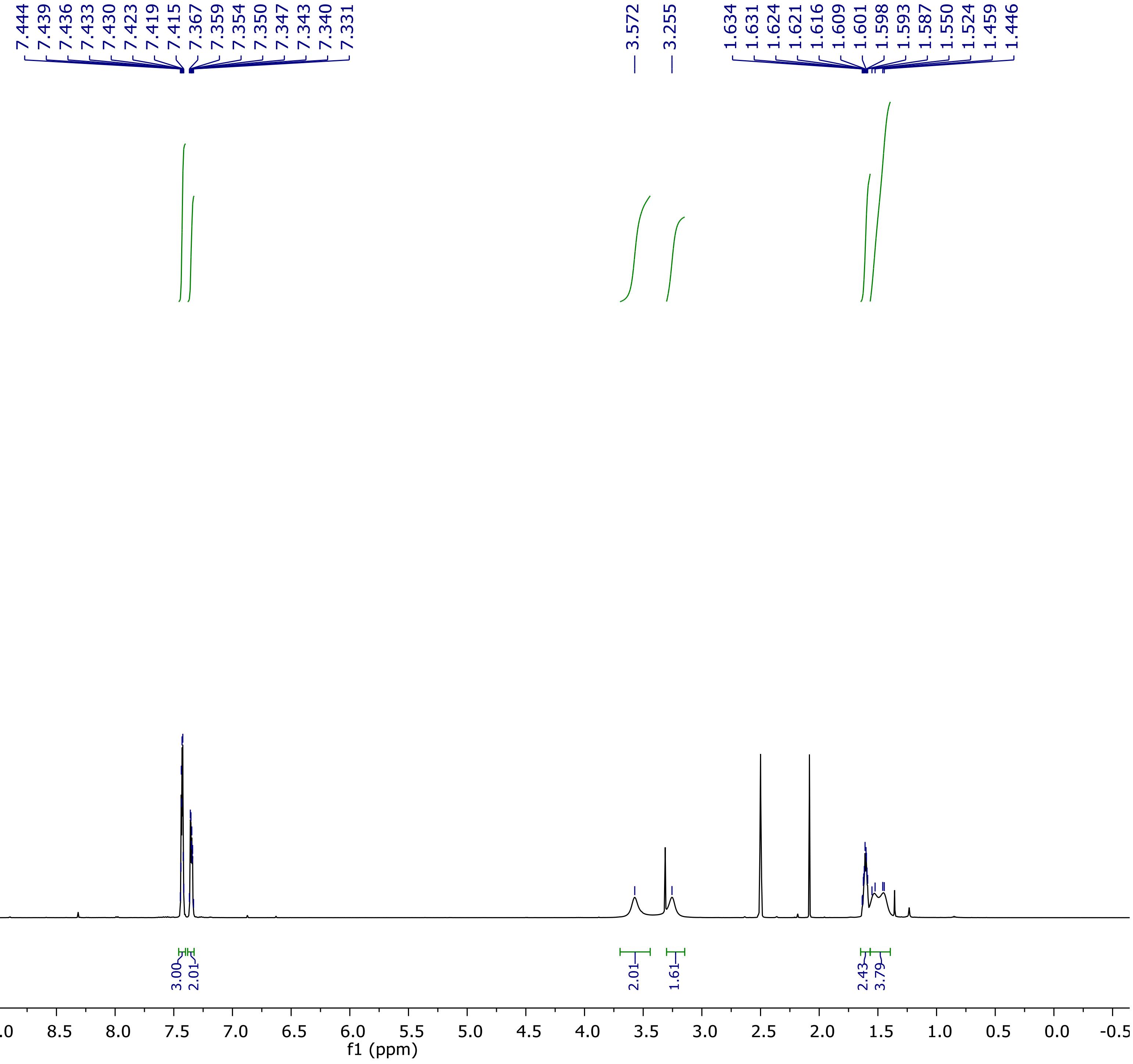

^{13}C NMR: 101 MHz, D_6-DMSO

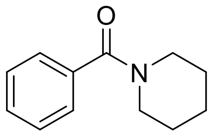

3p

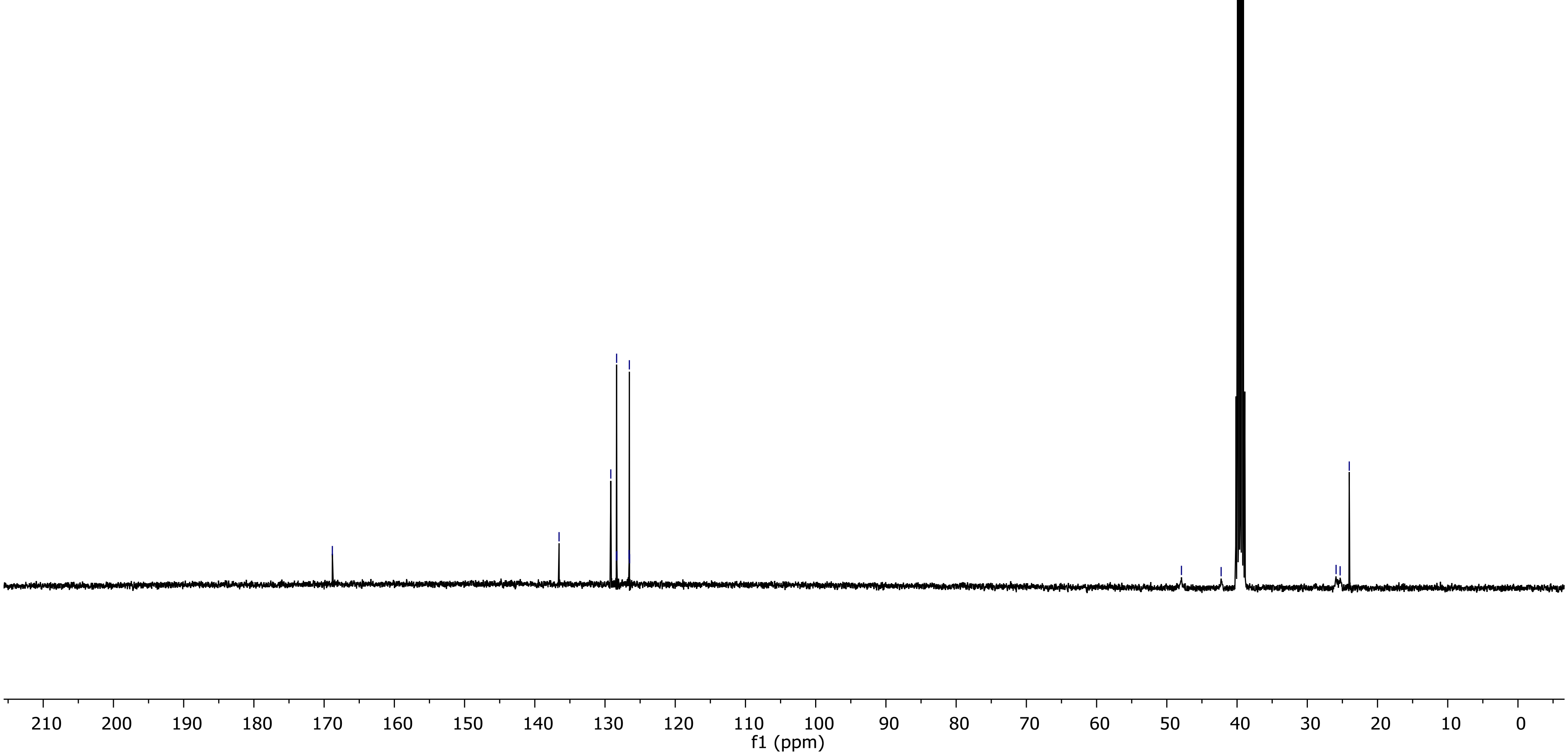

^{1}H NMR: 400 MHz, $CDCl_3$

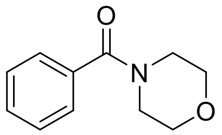

3q

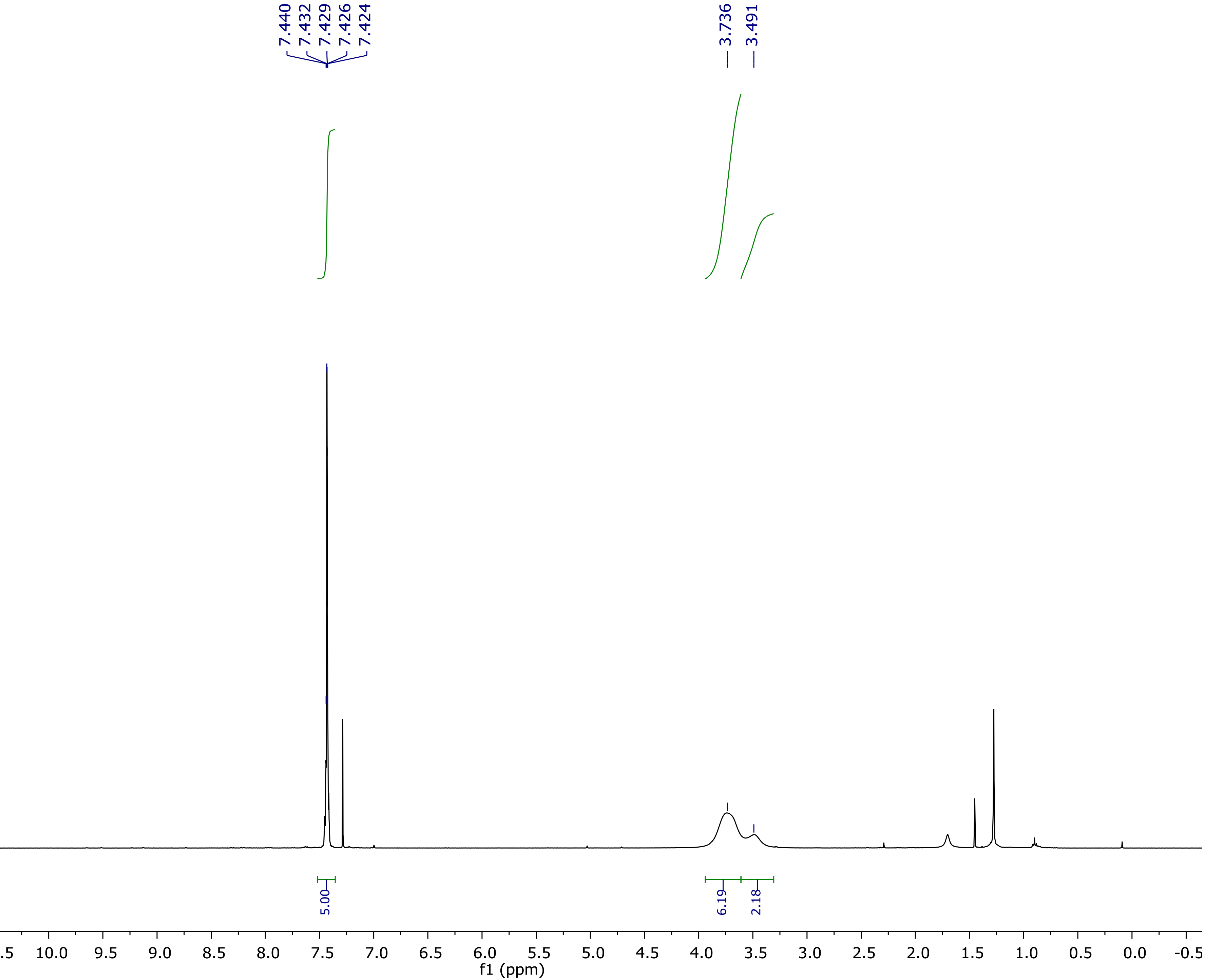

^{13}C NMR: 101 MHz, $CDCl_3$

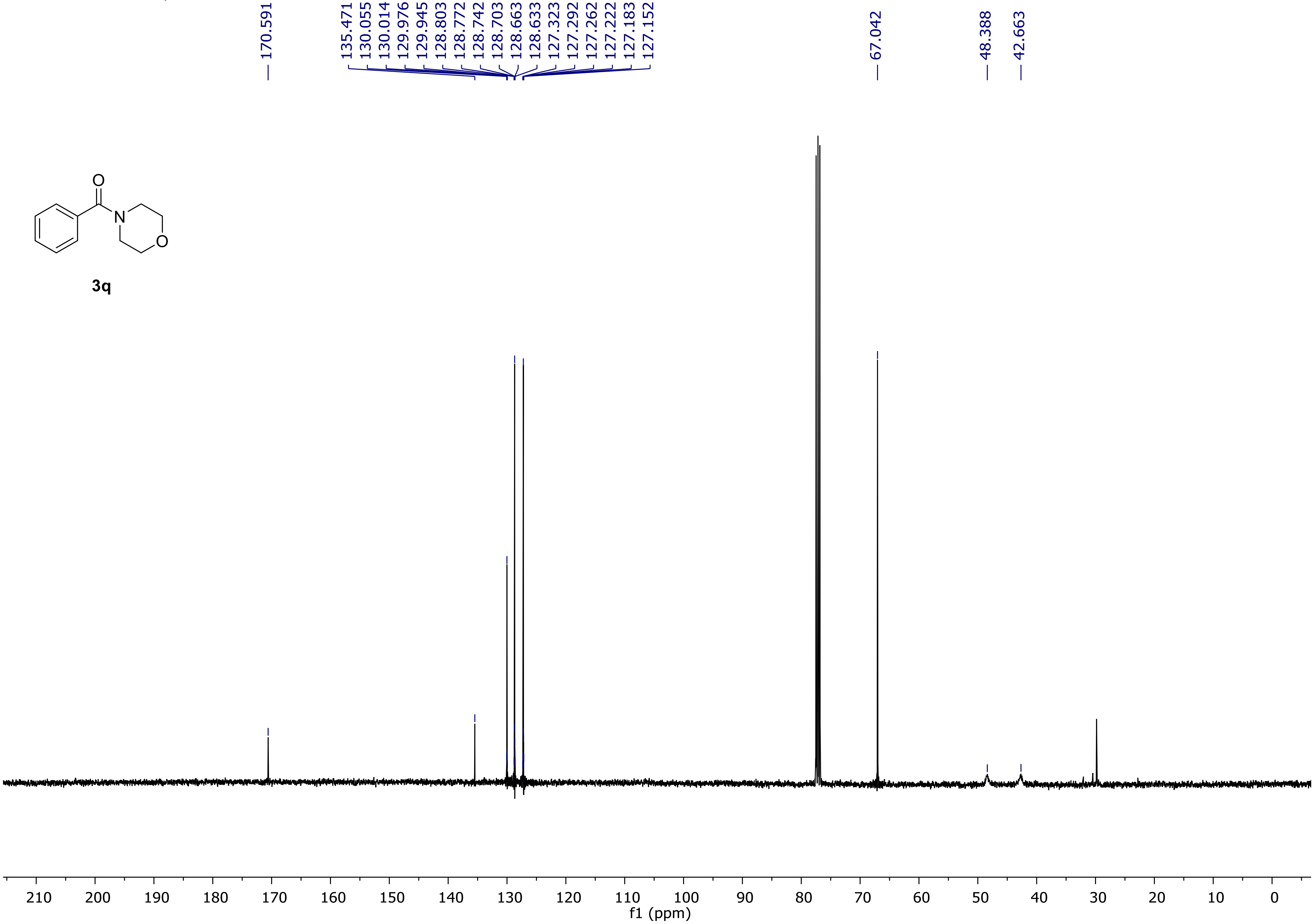

^{1}H NMR: 500 MHz, D_6-DMSO

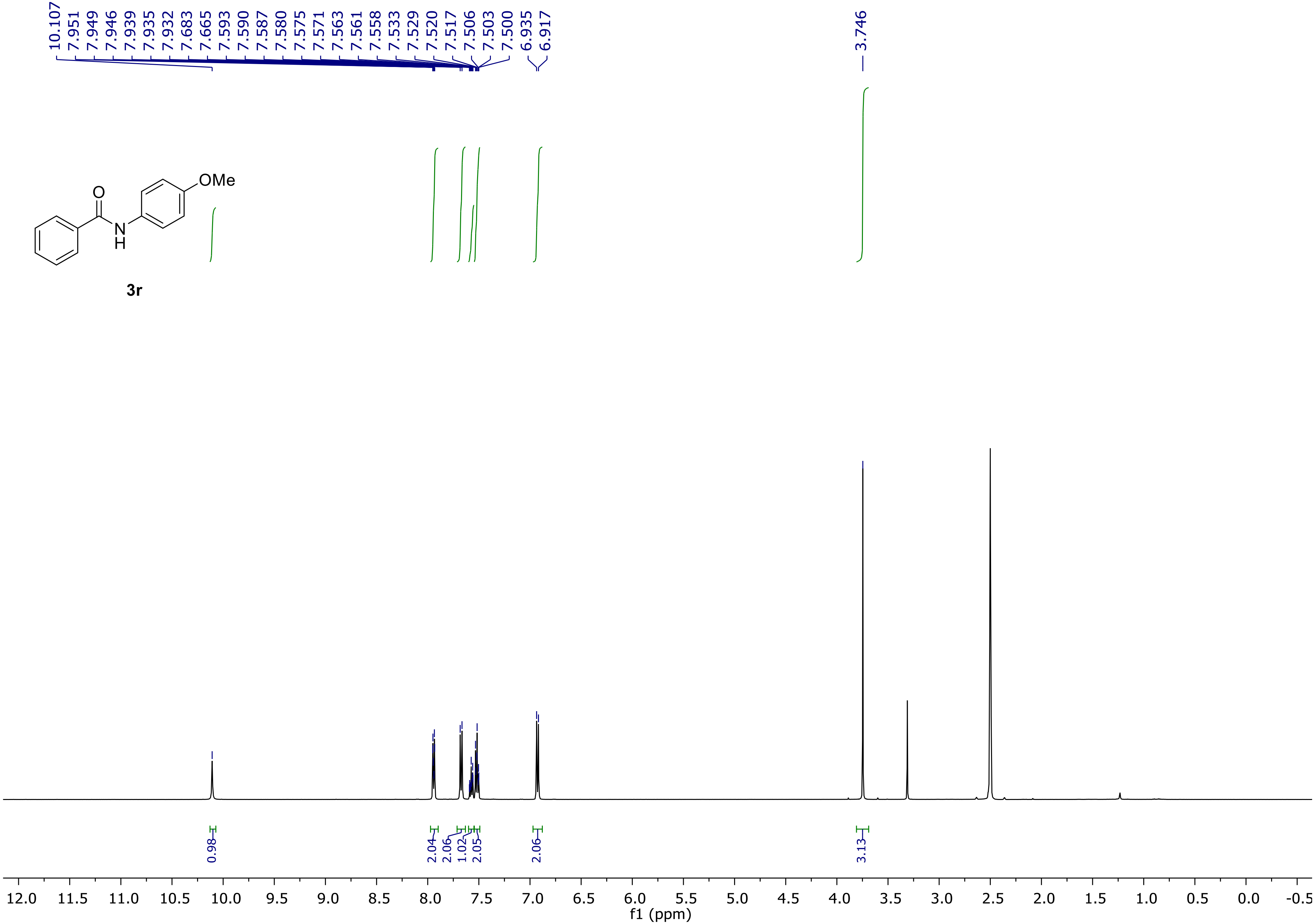

3r

^{13}C NMR: 126 MHz,
D_6-DMSO

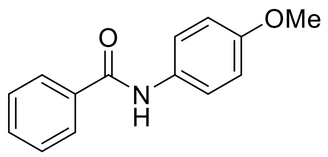

3r

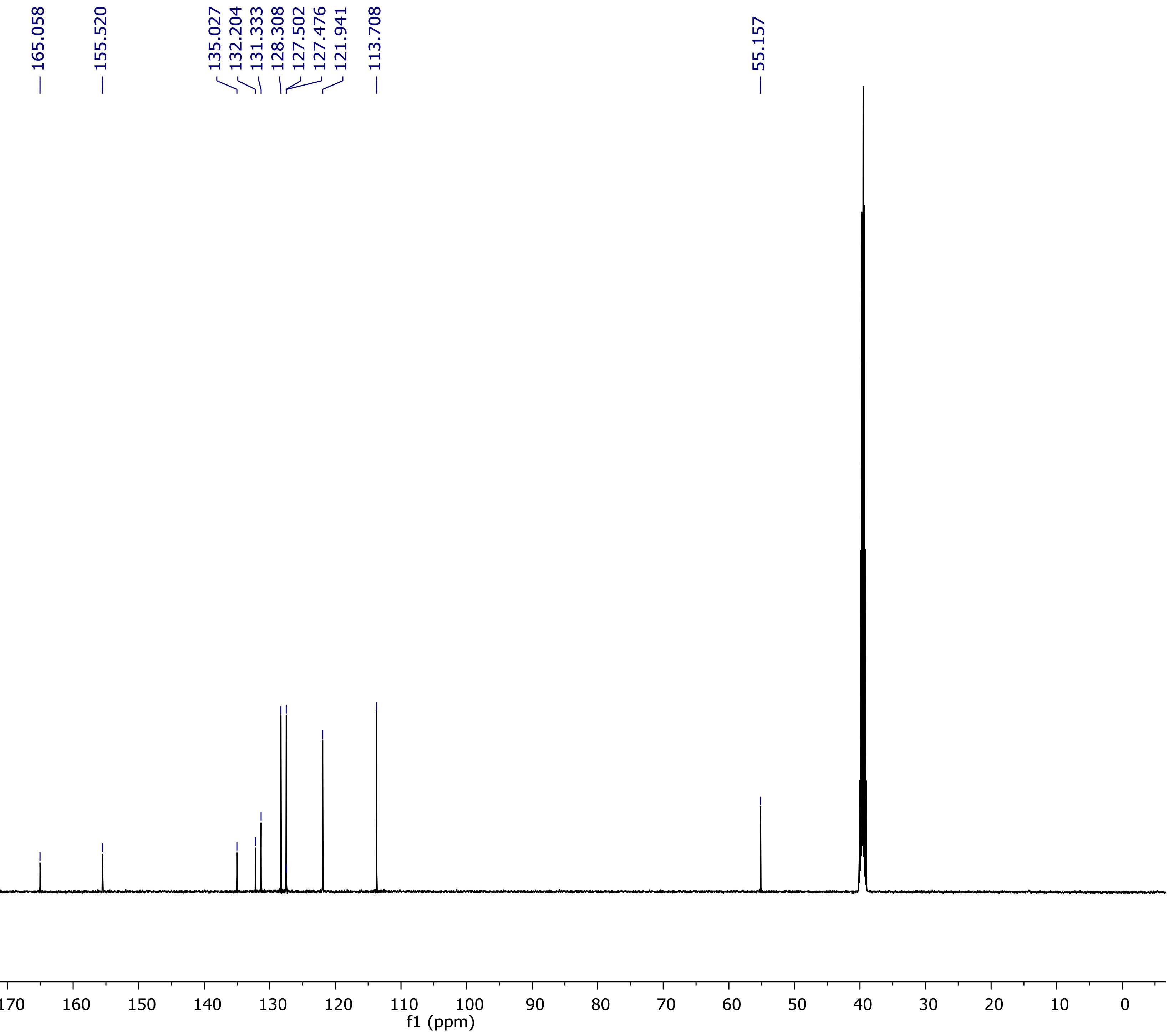

^{1}H NMR: 500 MHz, $CDCl_3$

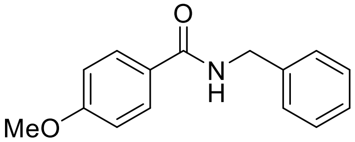

4a

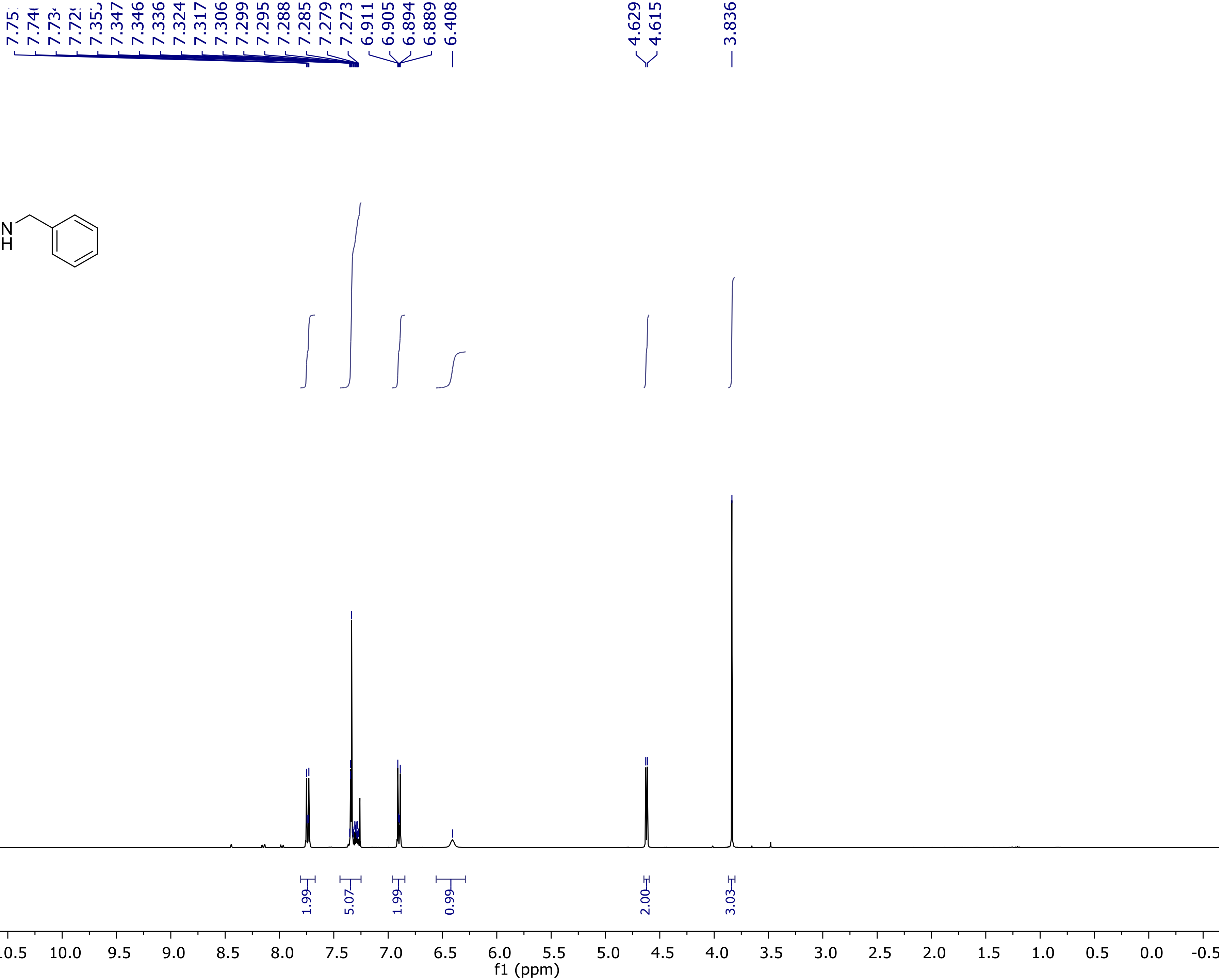

^{13}C NMR: 101 MHz, $CDCl_3$

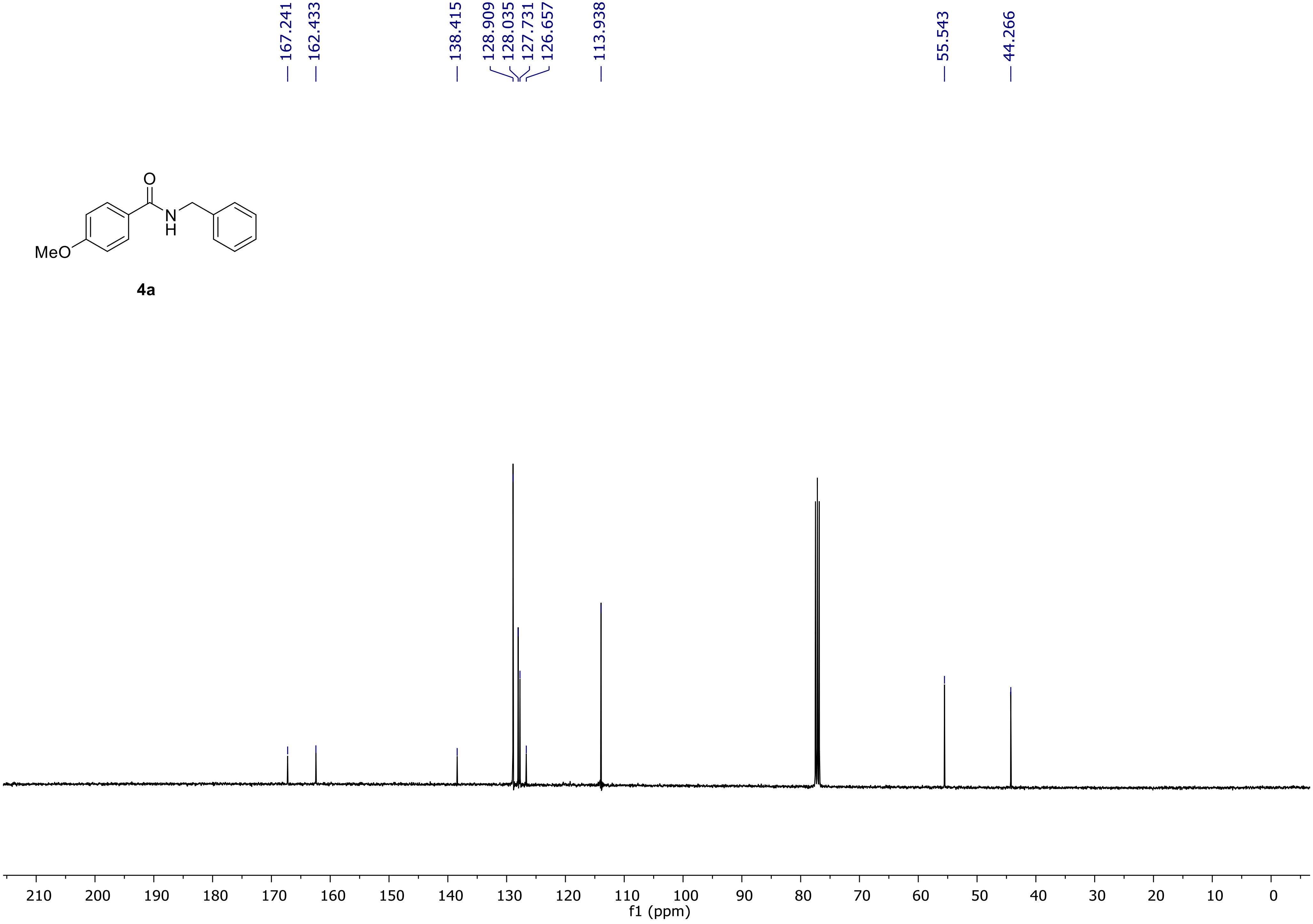

4a

¹H NMR: 400 MHz, $CDCl_3$

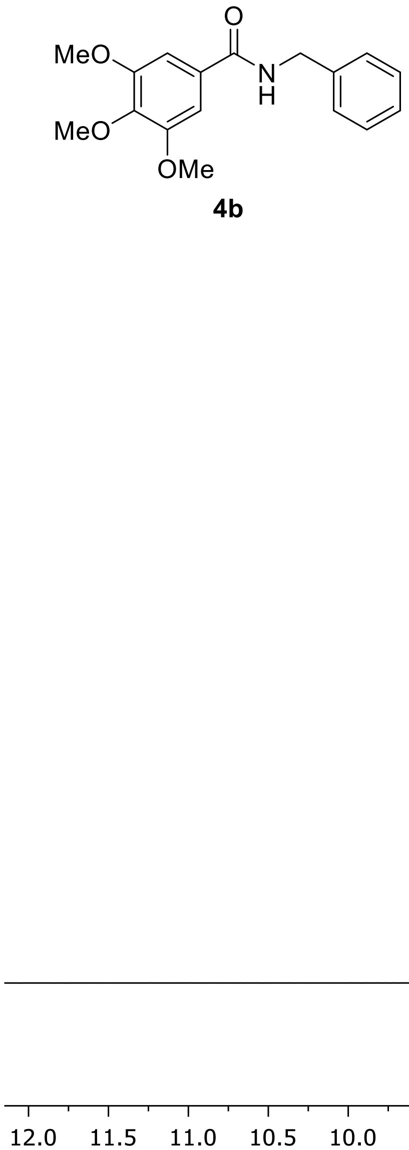

4b

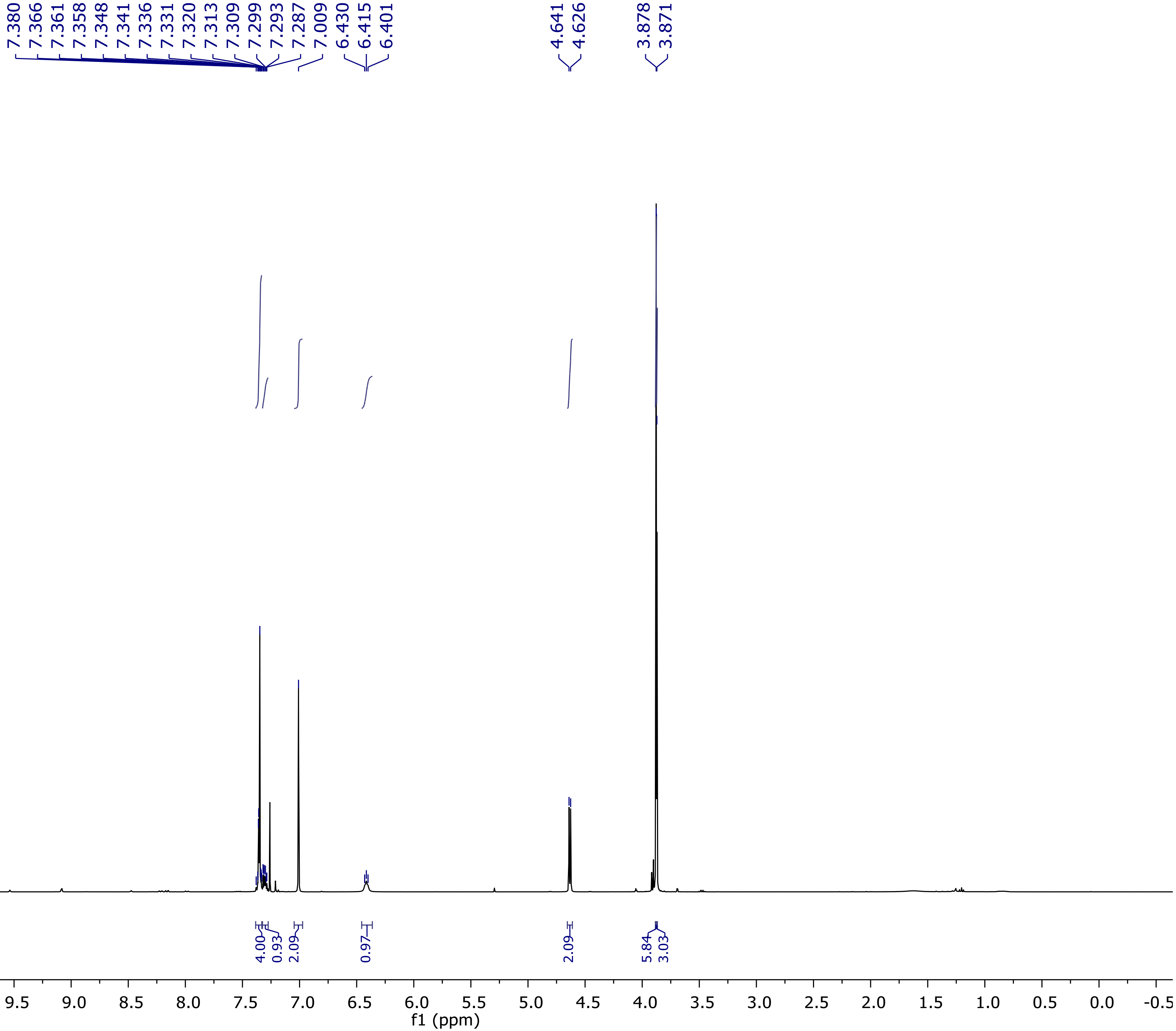

^{13}C NMR: 101 MHz, $CDCl_3$

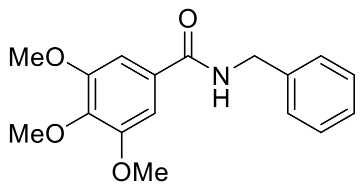

4b

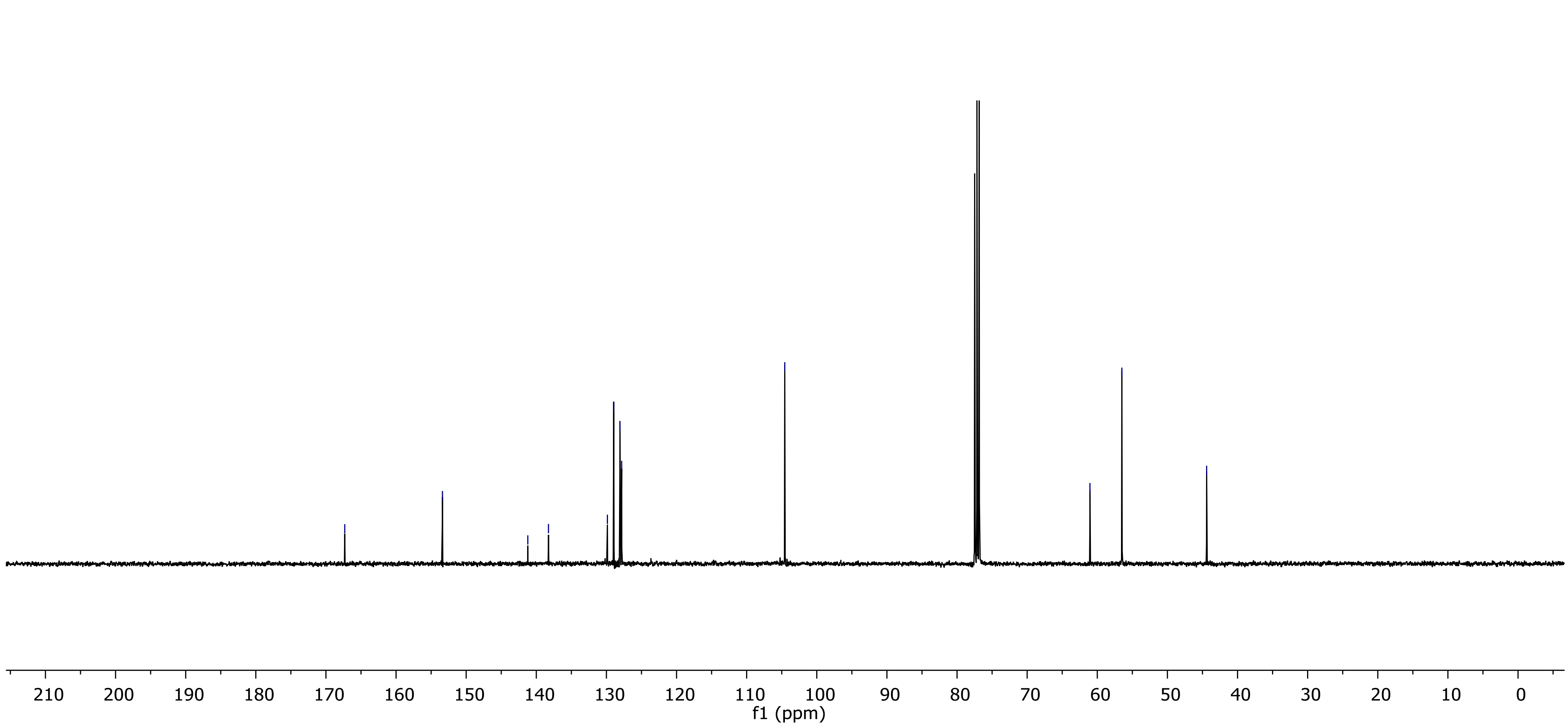

^{1}H NMR: 400 MHz, $CDCl_3$

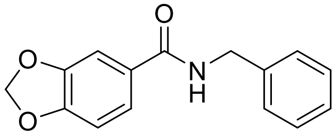

4c

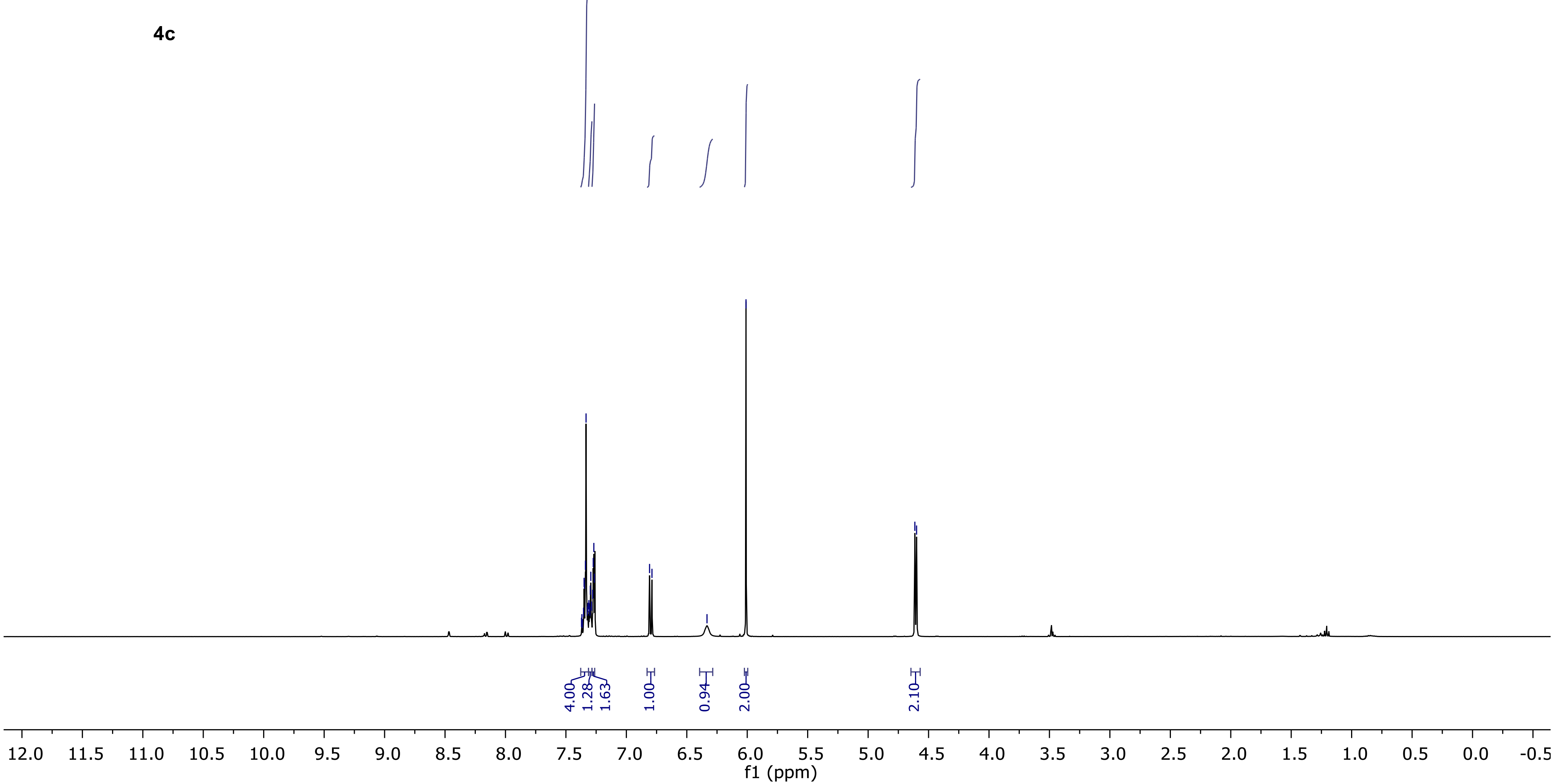

^{13}C NMR: 101 MHz, $CDCl_3$

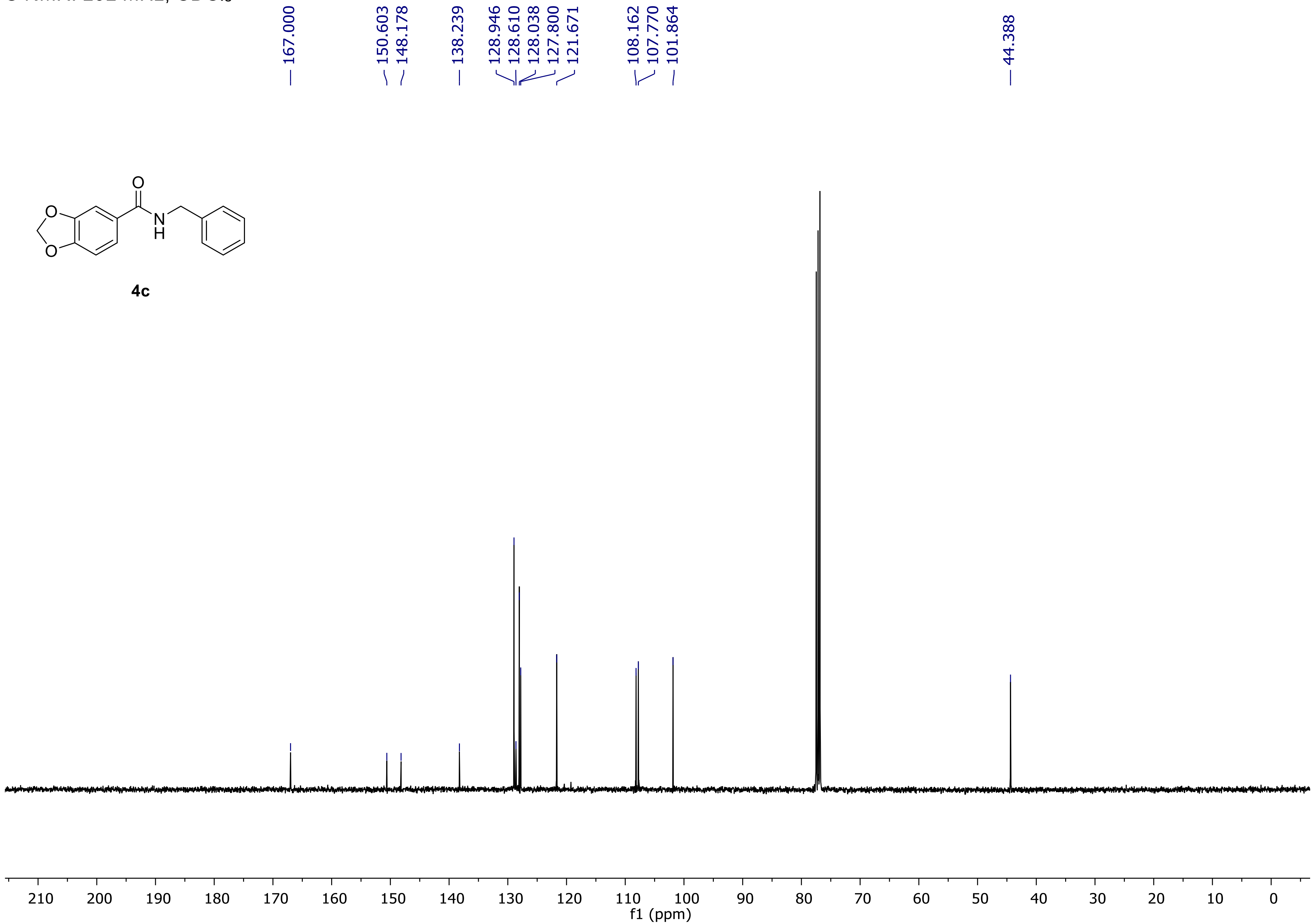

4c

^{1}H NMR: 400 MHz, $CDCl_3$

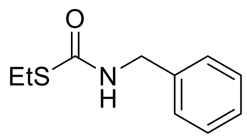

4d

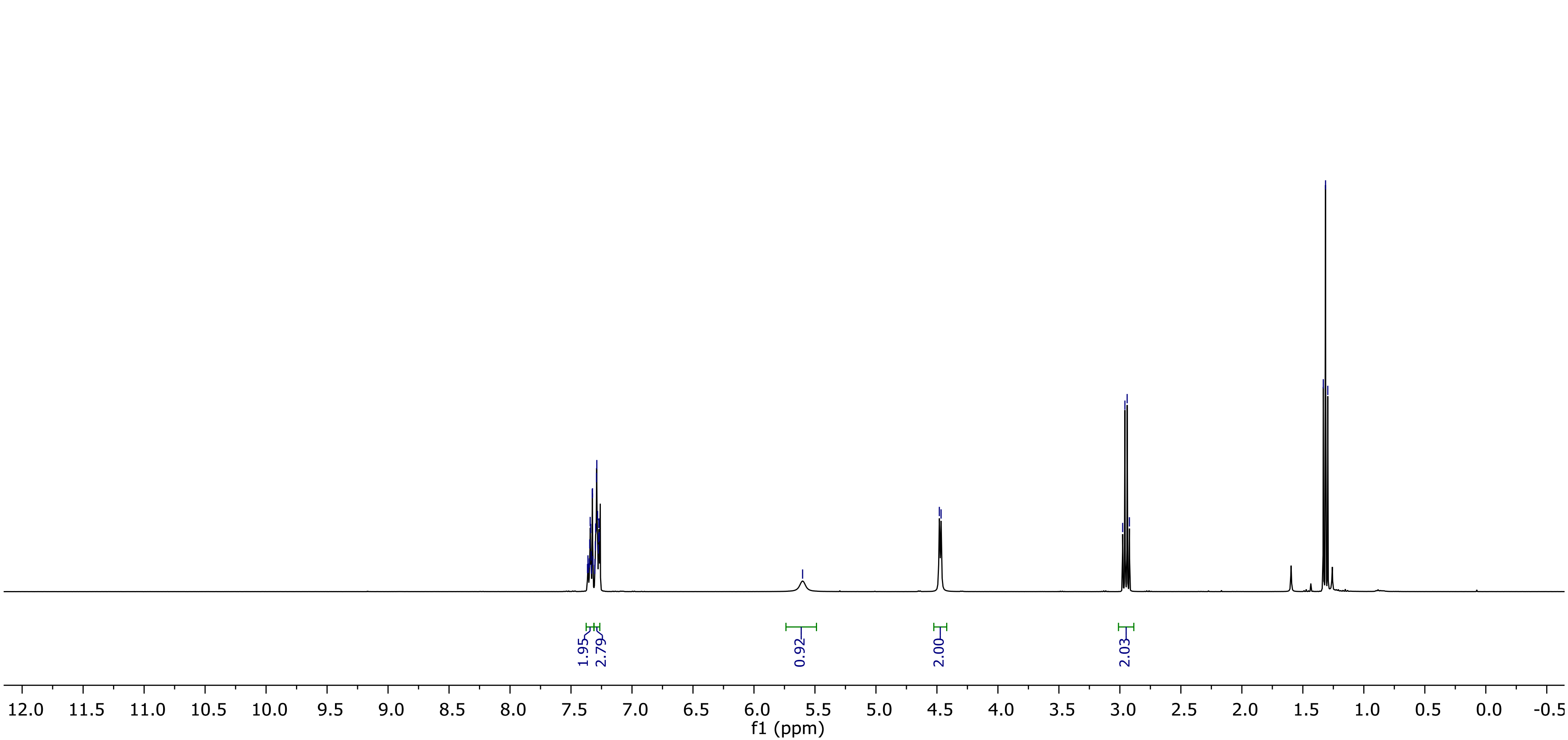

^{13}C NMR: 101 MHz, $CDCl_3$

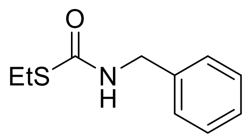

4d

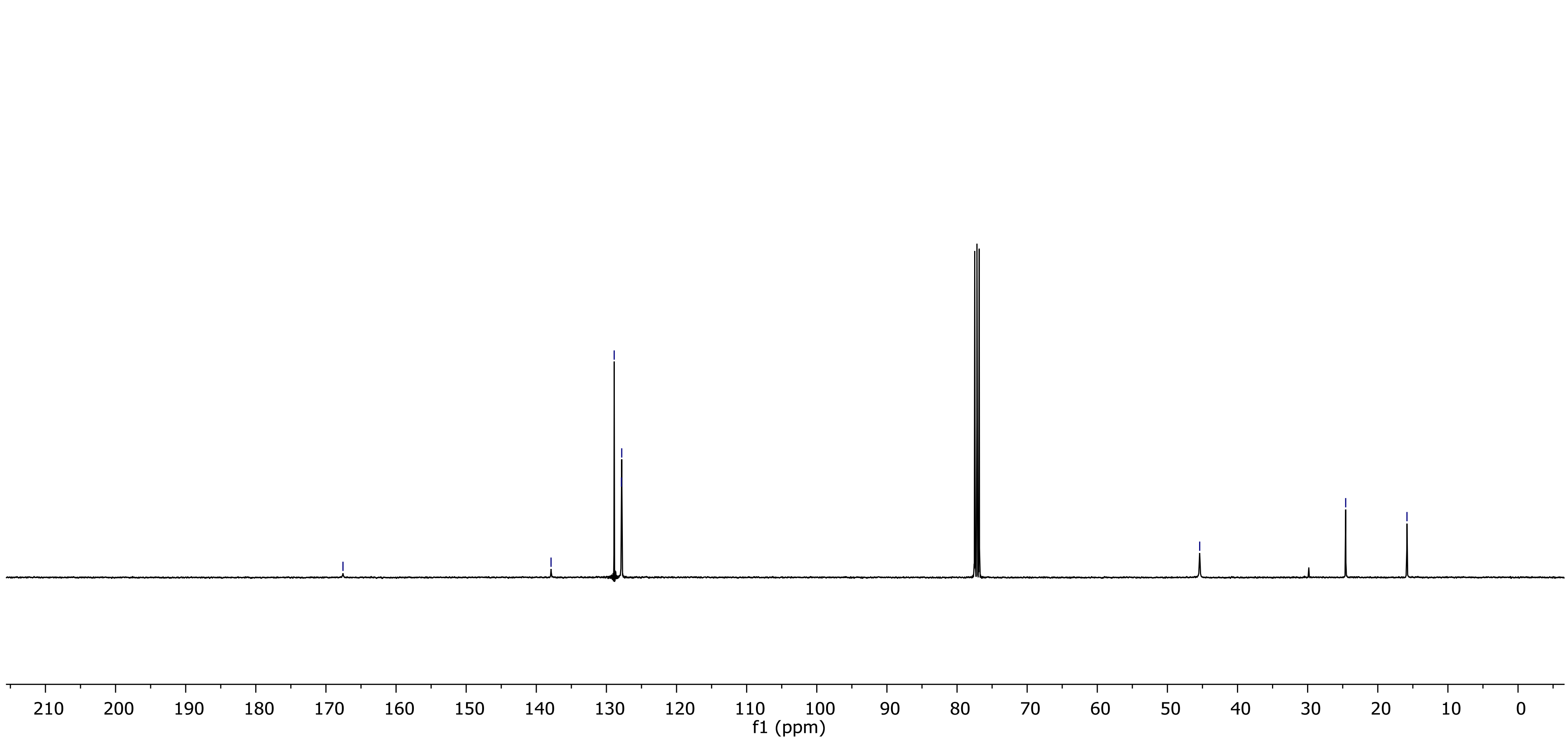

^{1}H NMR: 500 MHz, $CDCl_3$

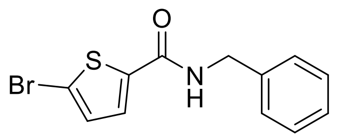

4e

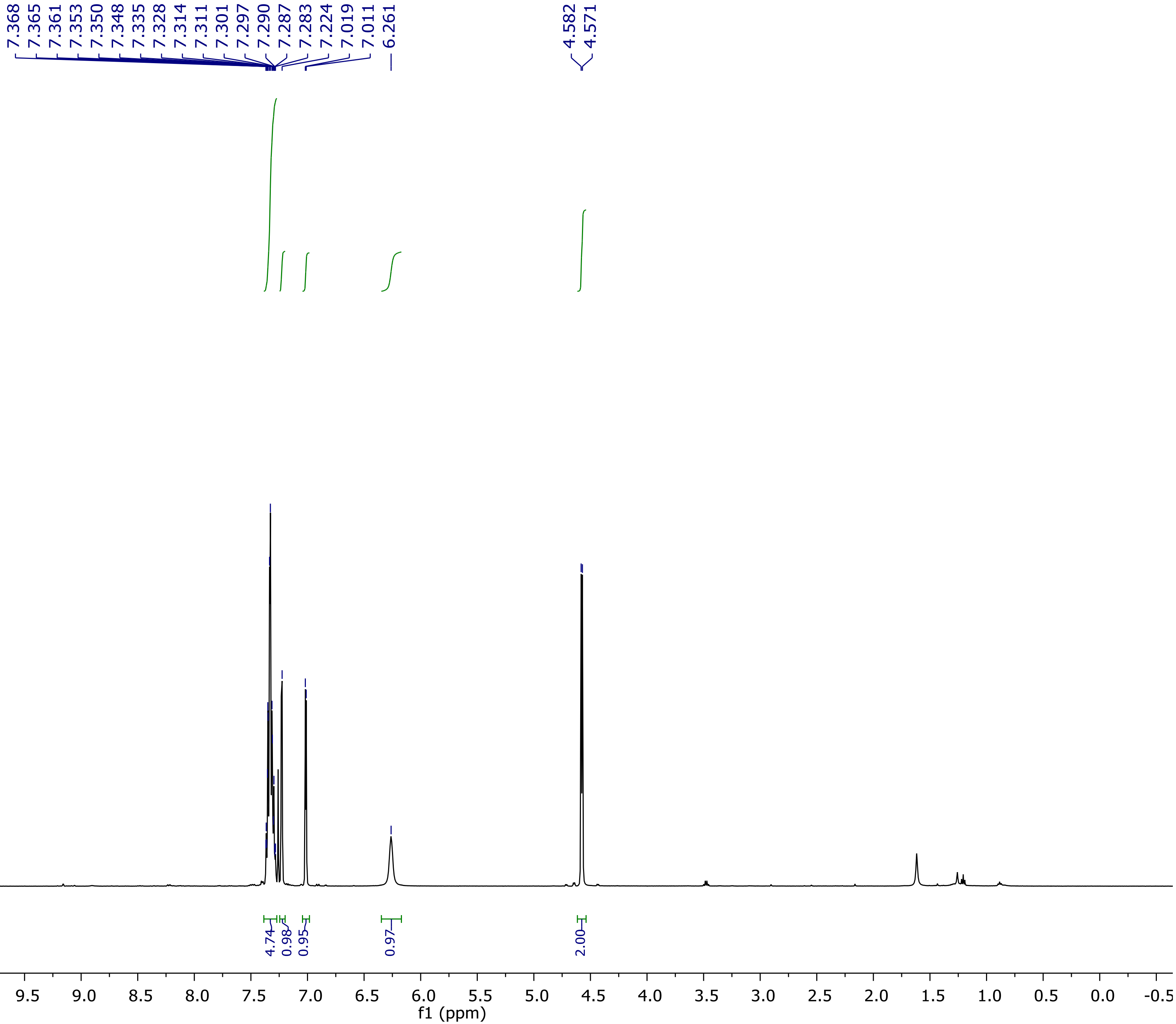

^{13}C NMR: 126 MHz, $CDCl_3$

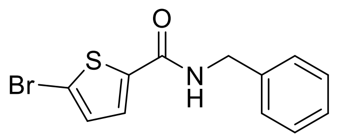

4e

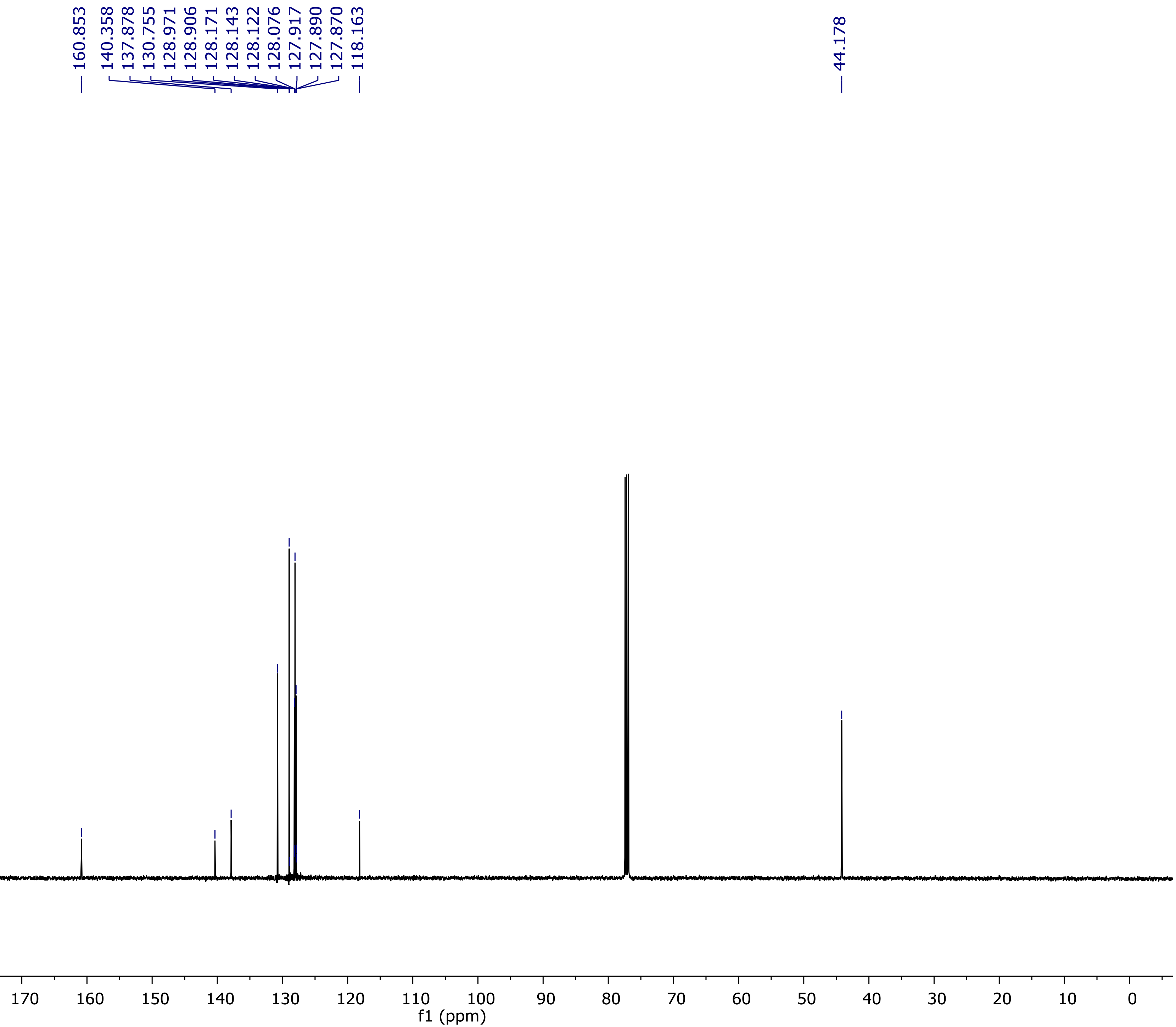

¹H NMR: 400 MHz, $CDCl_3$

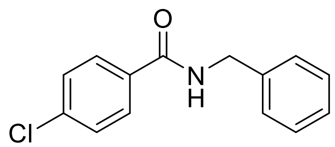

4f

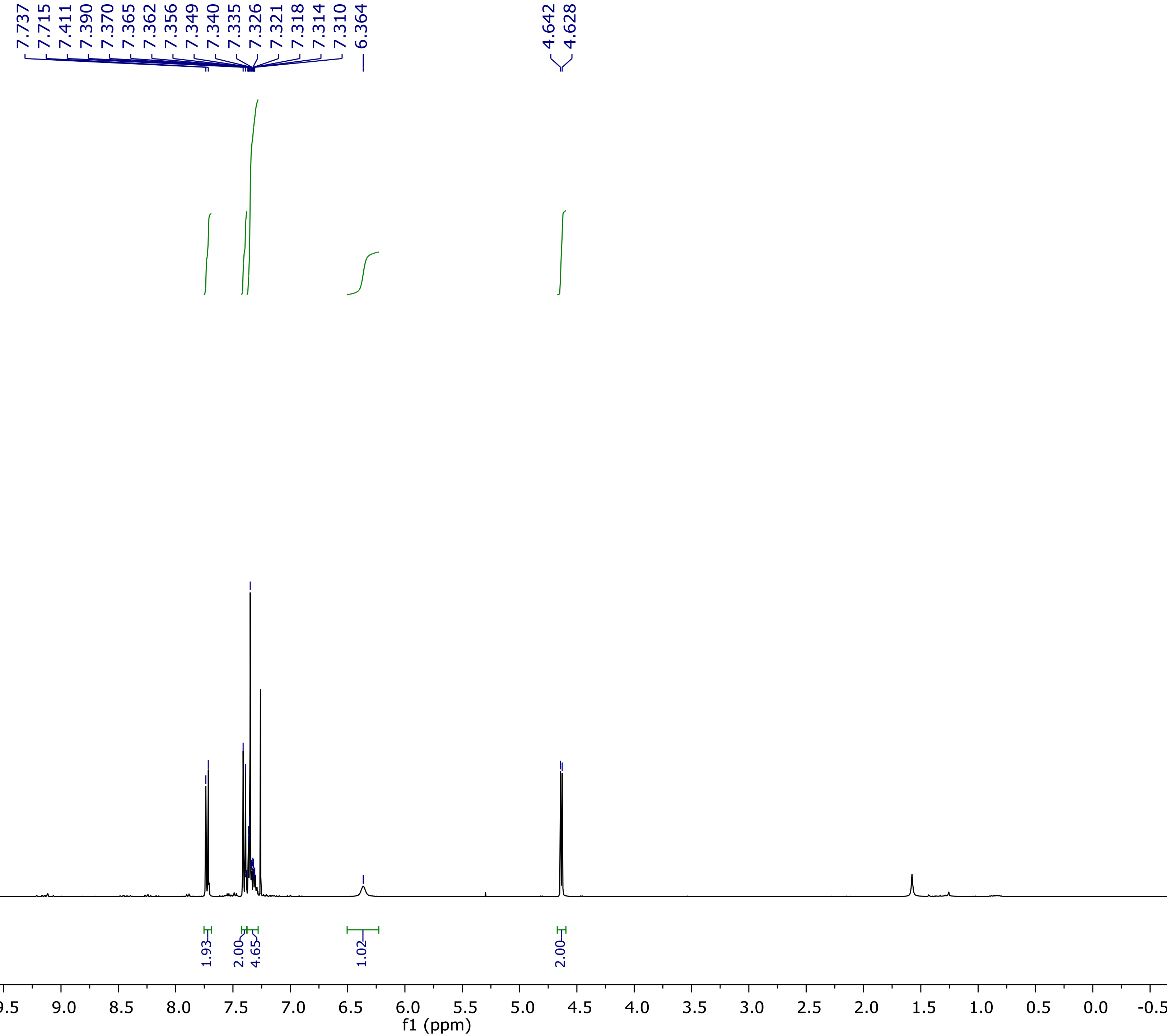

^{13}C NMR: 101 MHz, $CDCl_3$

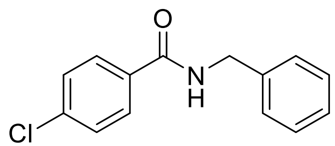

4f

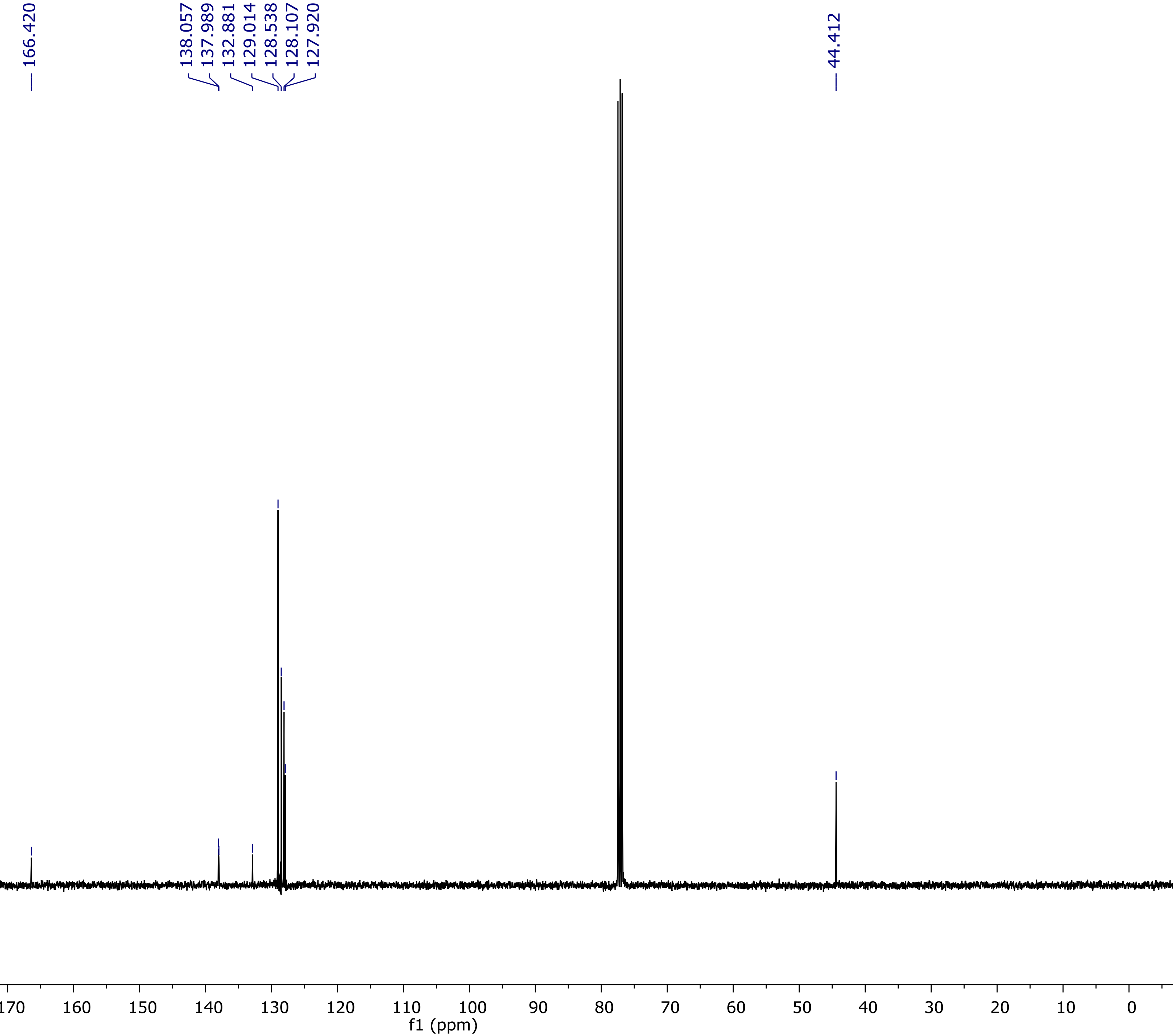

^{1}H NMR: 400 MHz, $CDCl_3$

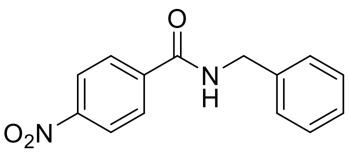

4g

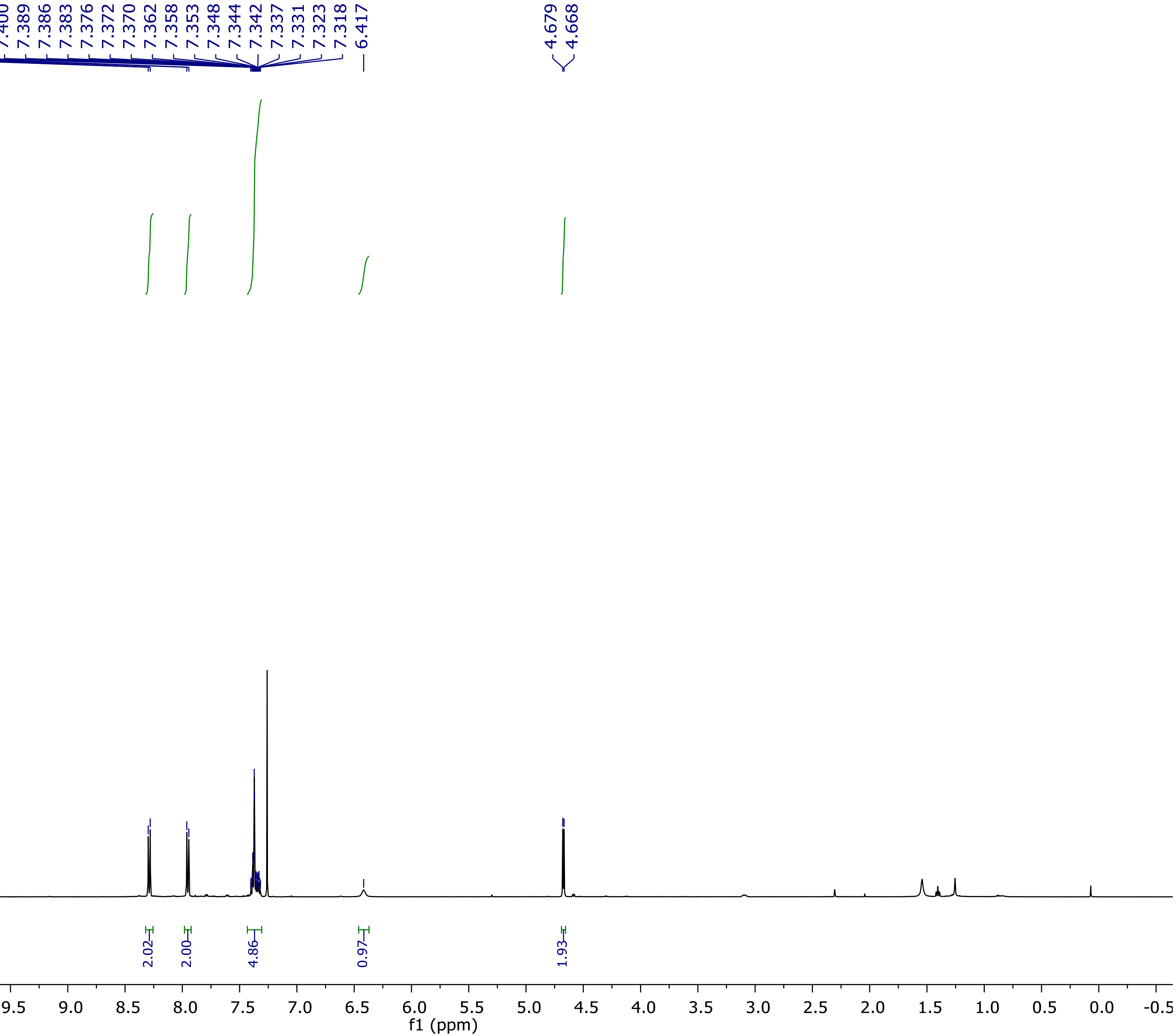

^{13}C NMR: 101 MHz, $CDCl_3$

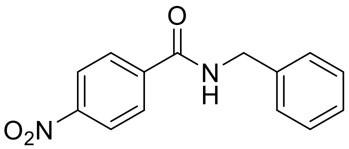

4g

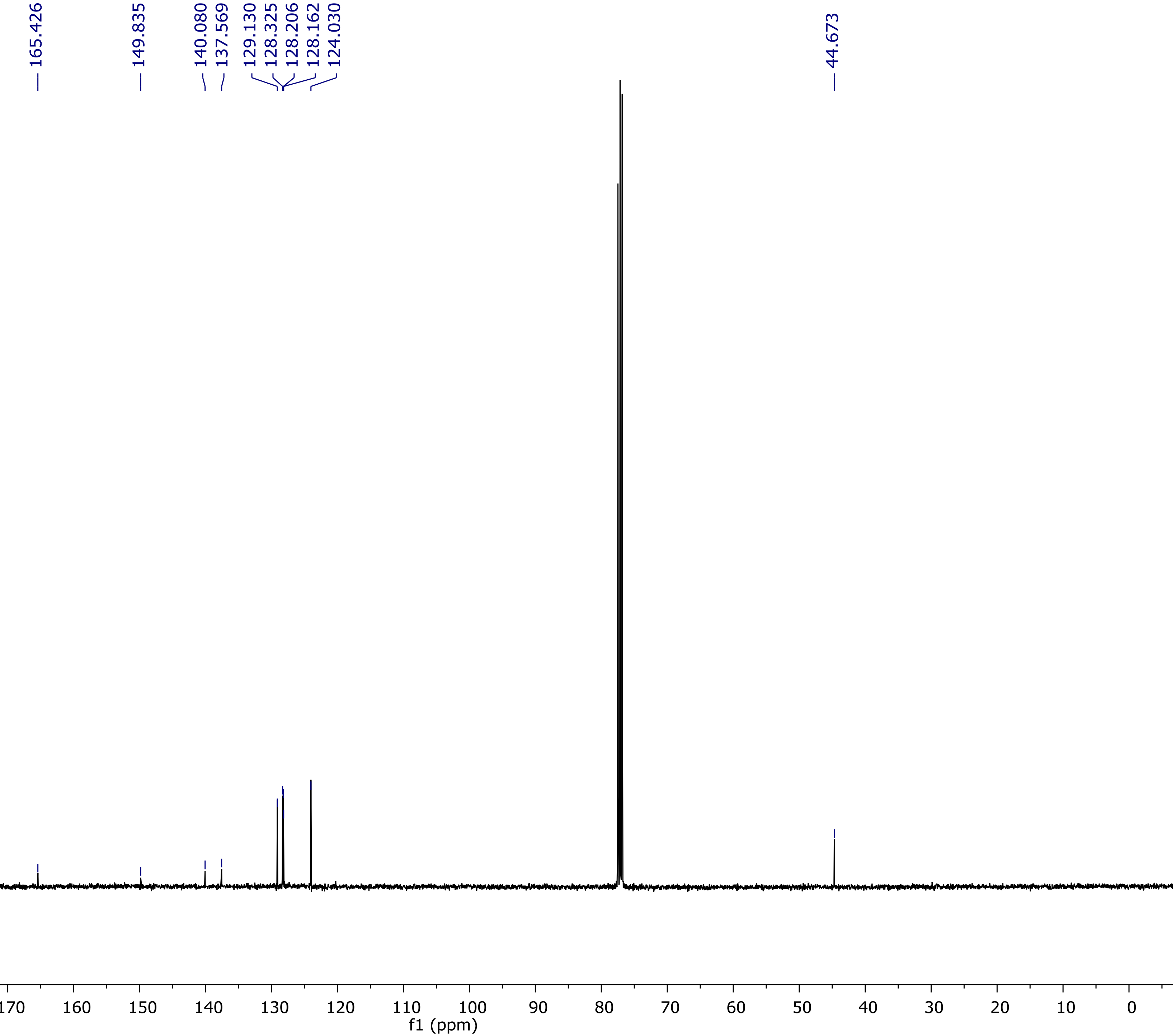

^{1}H NMR: 500 MHz, $CDCl_3$

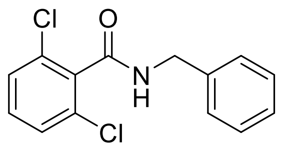

4h

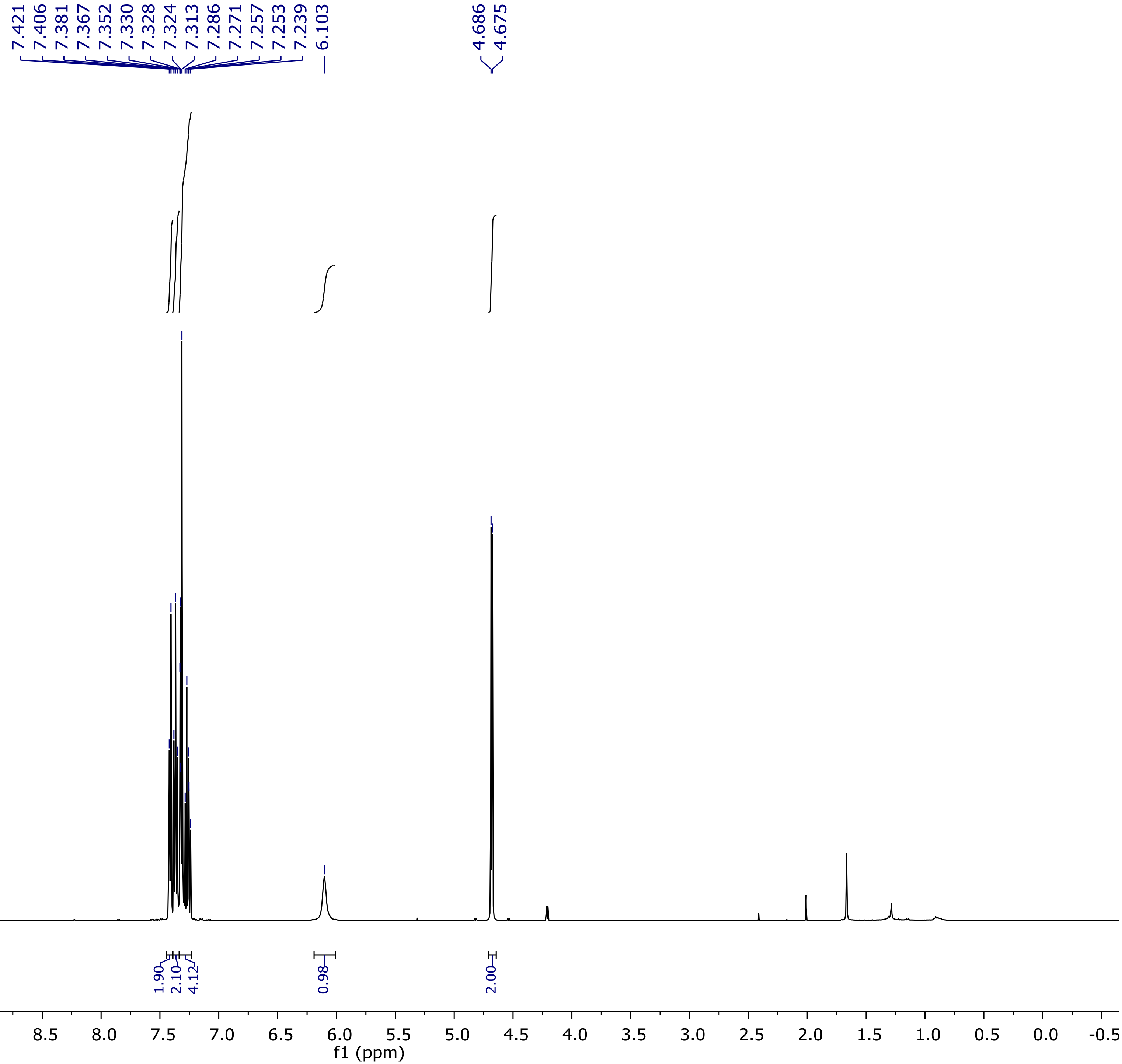

^{13}C NMR: 126 MHz, $CDCl_3$

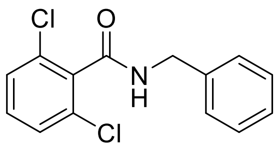

4h

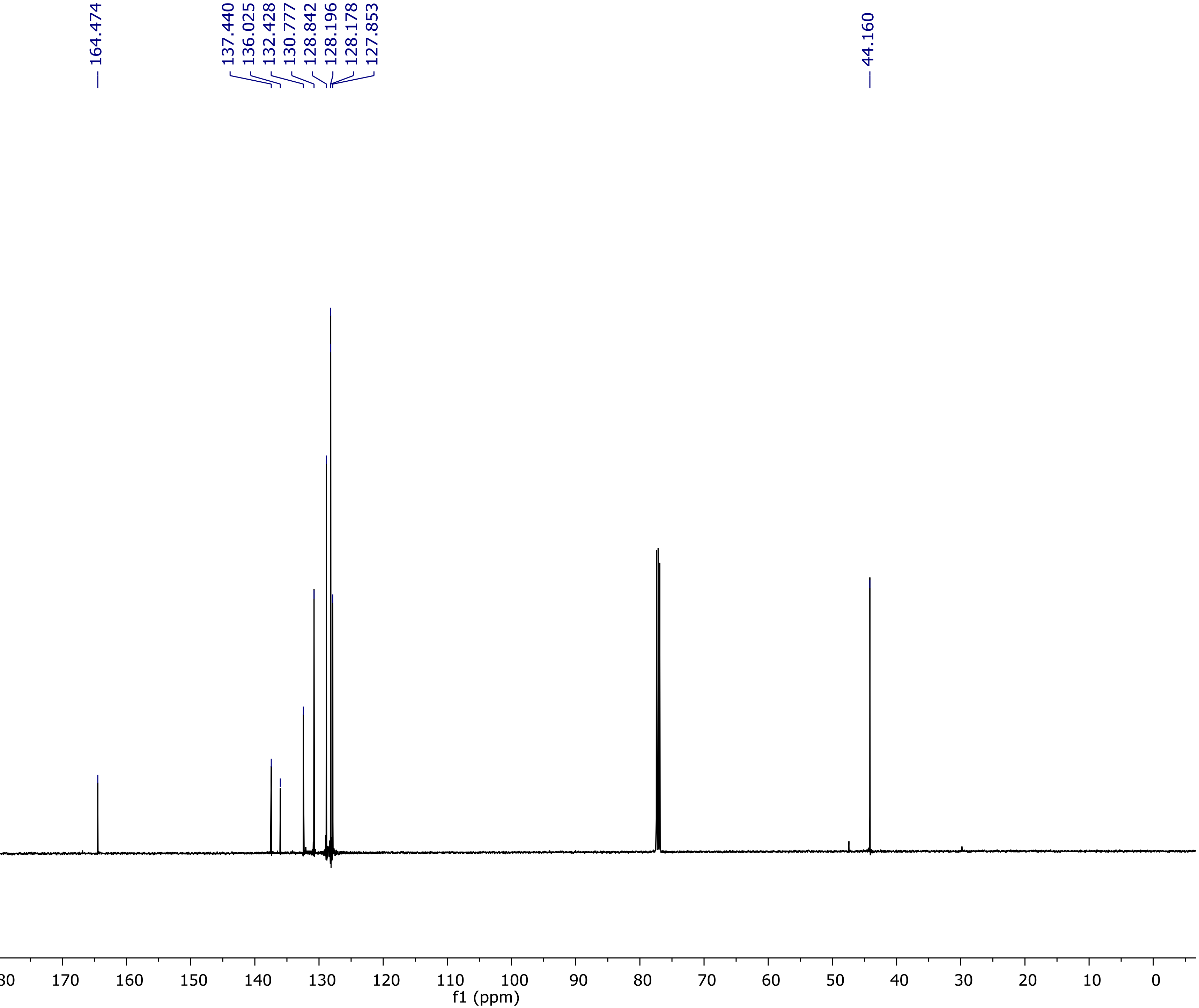

^{1}H NMR: 500 MHz, D_6-DMSO

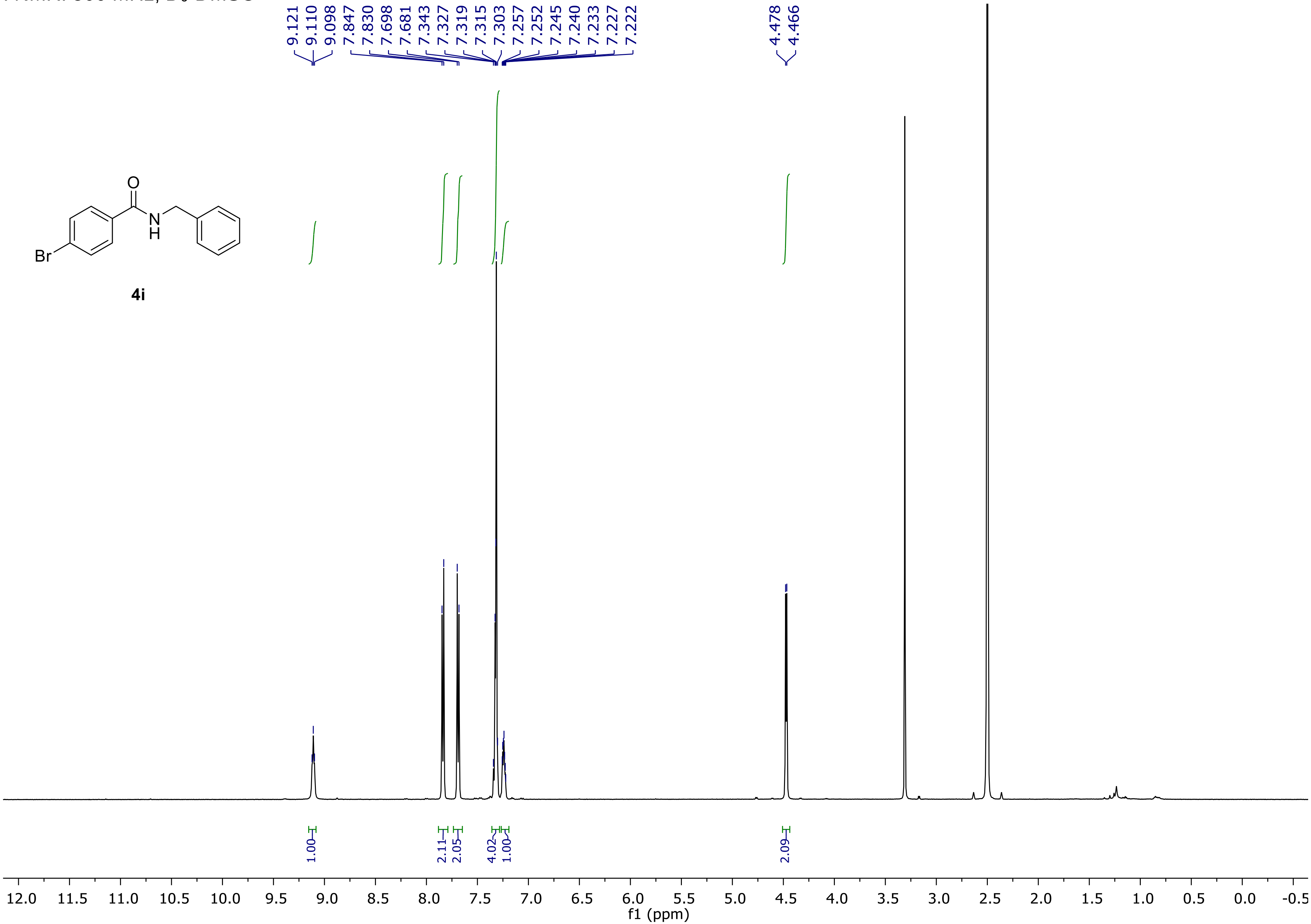

^{13}C NMR: 126 MHz, D_6-DMSO

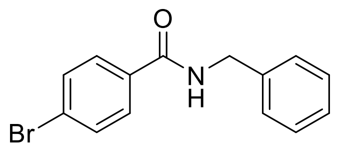

4i

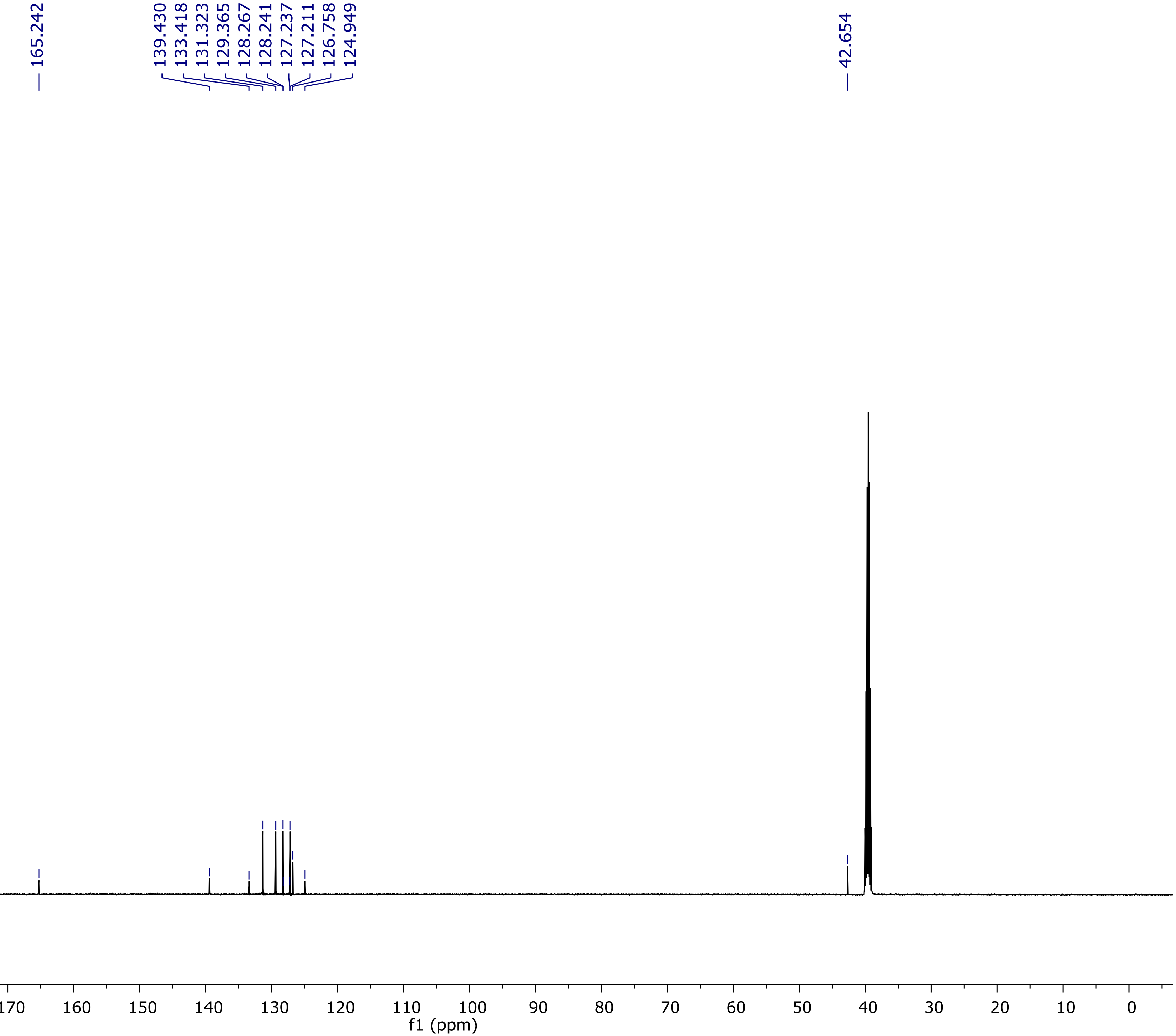

^{1}H NMR: 500 MHz, $CDCl_3$

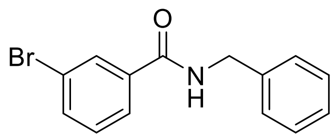

4j

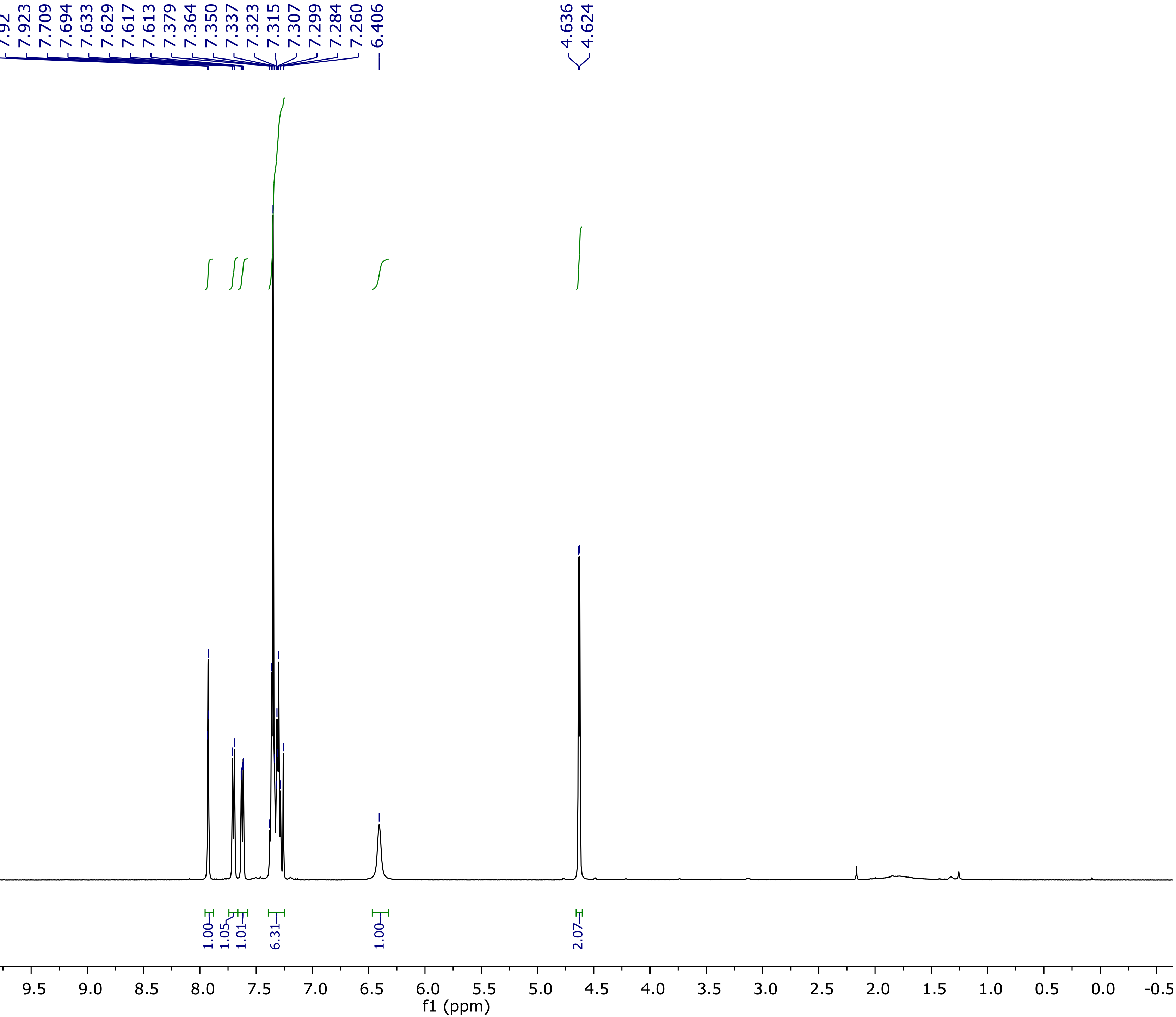

^{13}C NMR: 126 MHz, $CDCl_3$

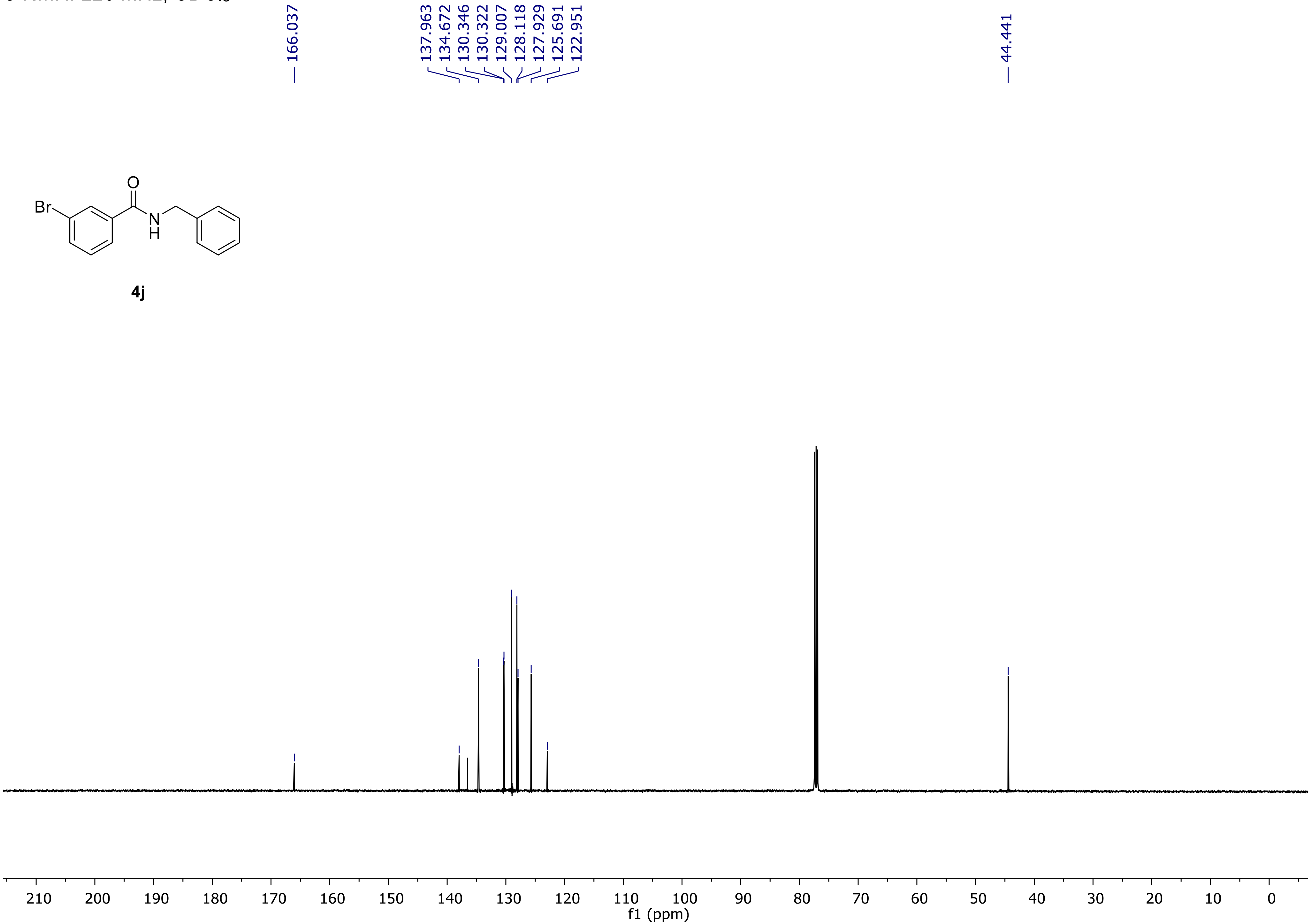

^1H NMR: 400 MHz, $CDCl_3$

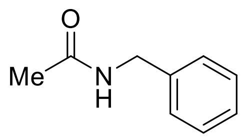

4k

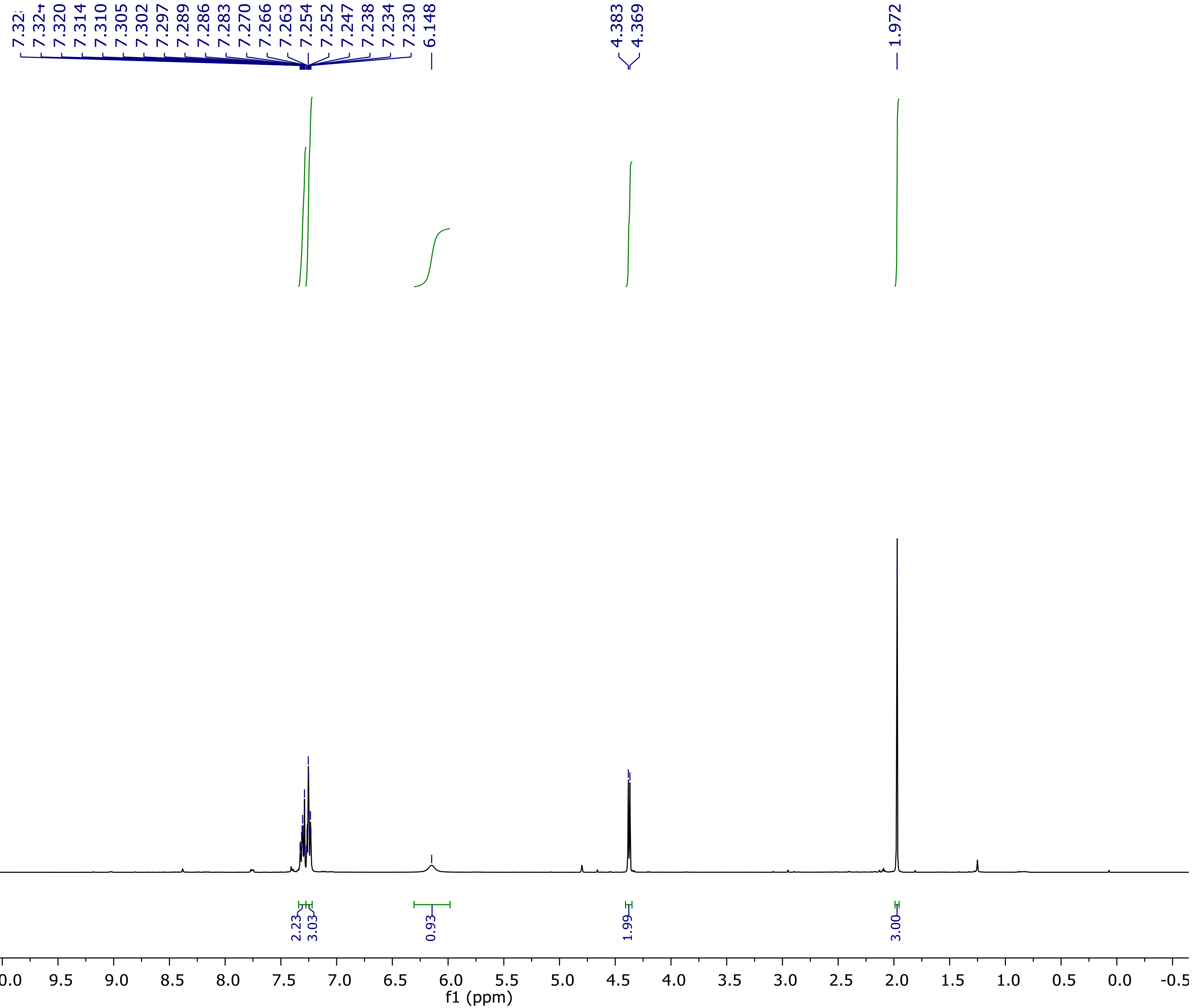

^{13}C NMR: 101 MHz, $CDCl_3$

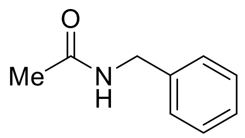

4k

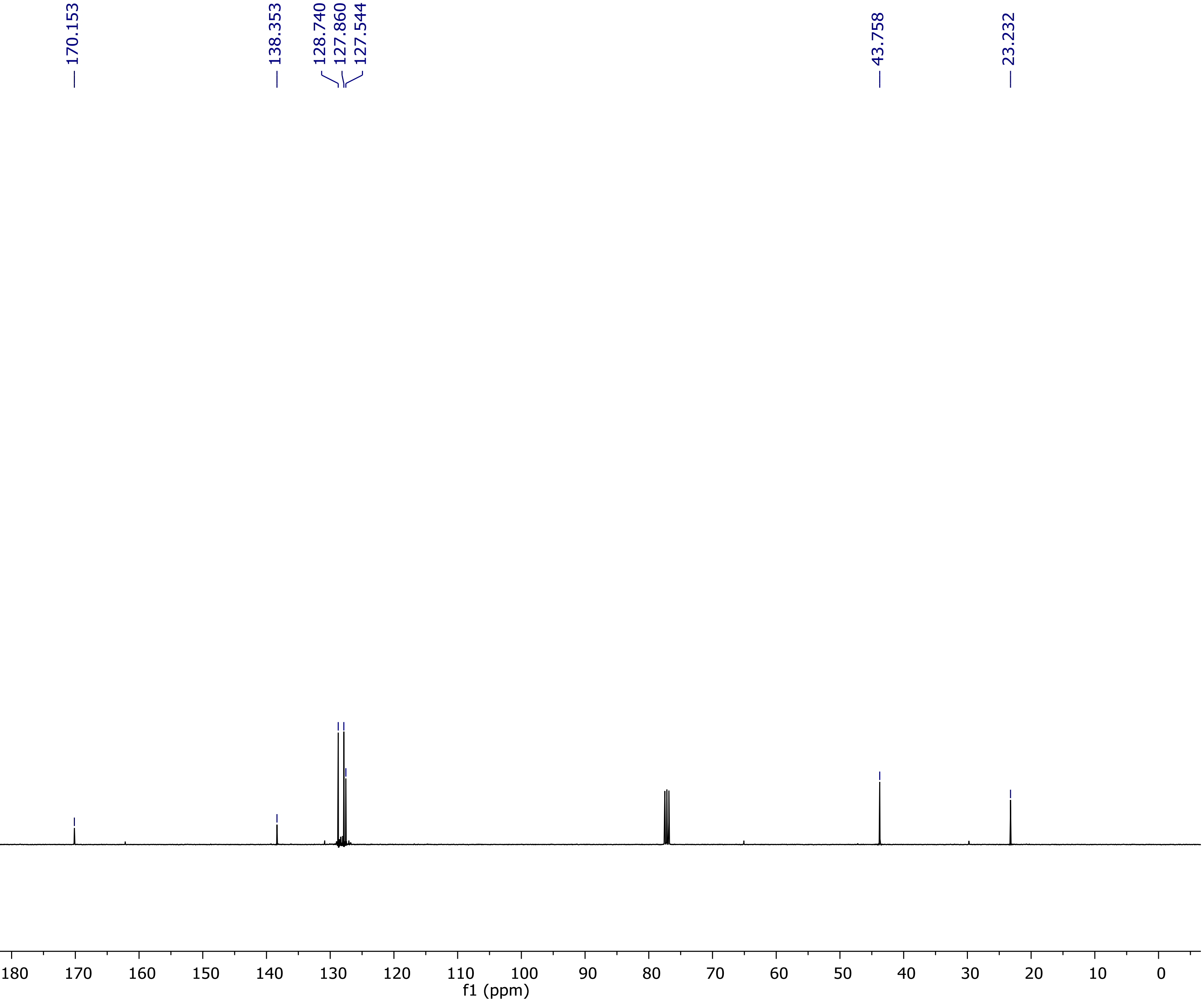

^1H NMR: 400 MHz, $CDCl_3$

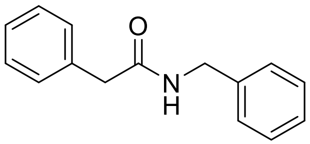

4l

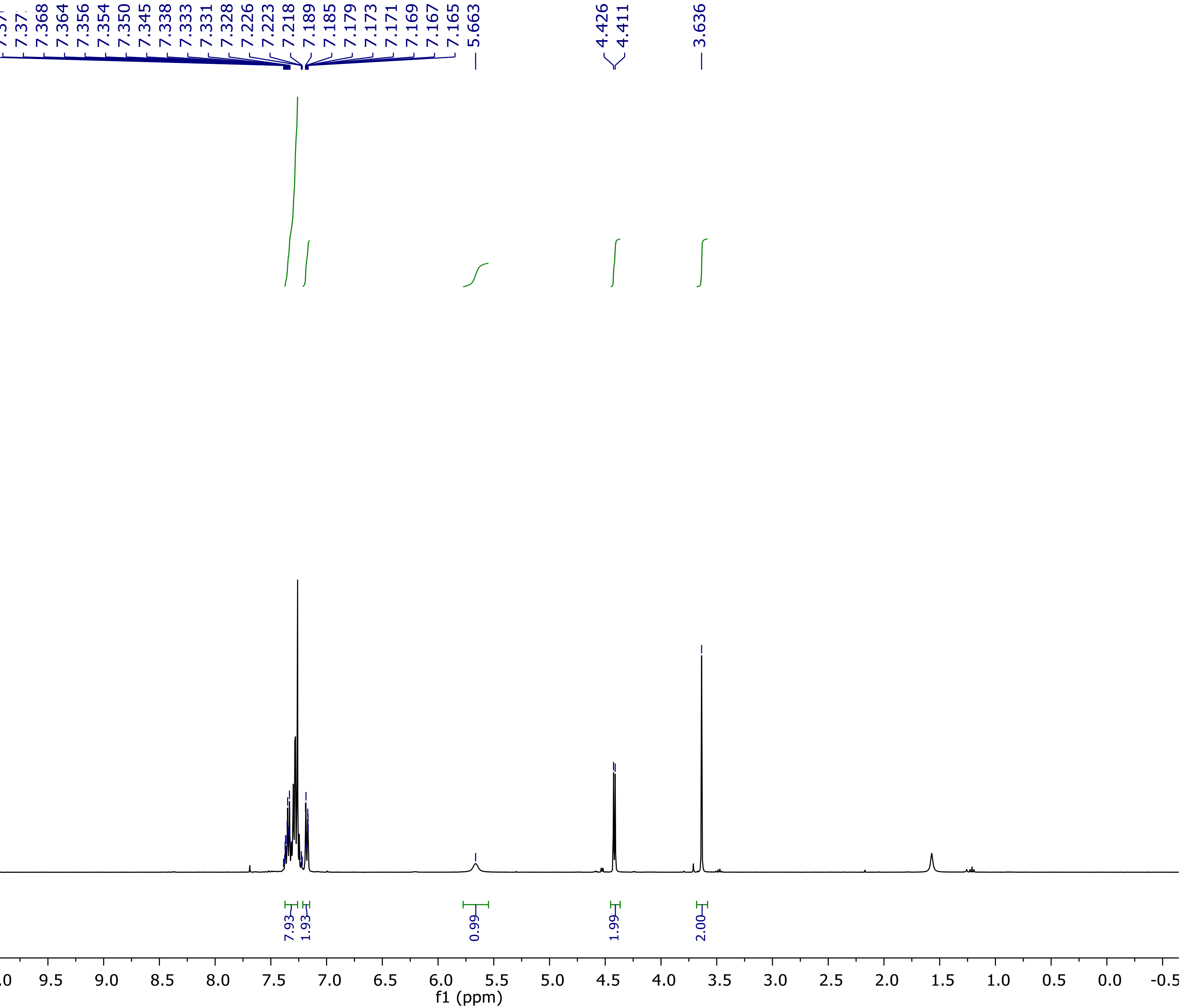

^{13}C NMR: 101 MHz, $CDCl_3$

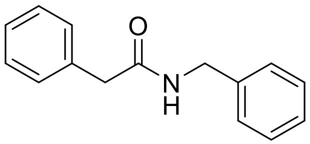

4l

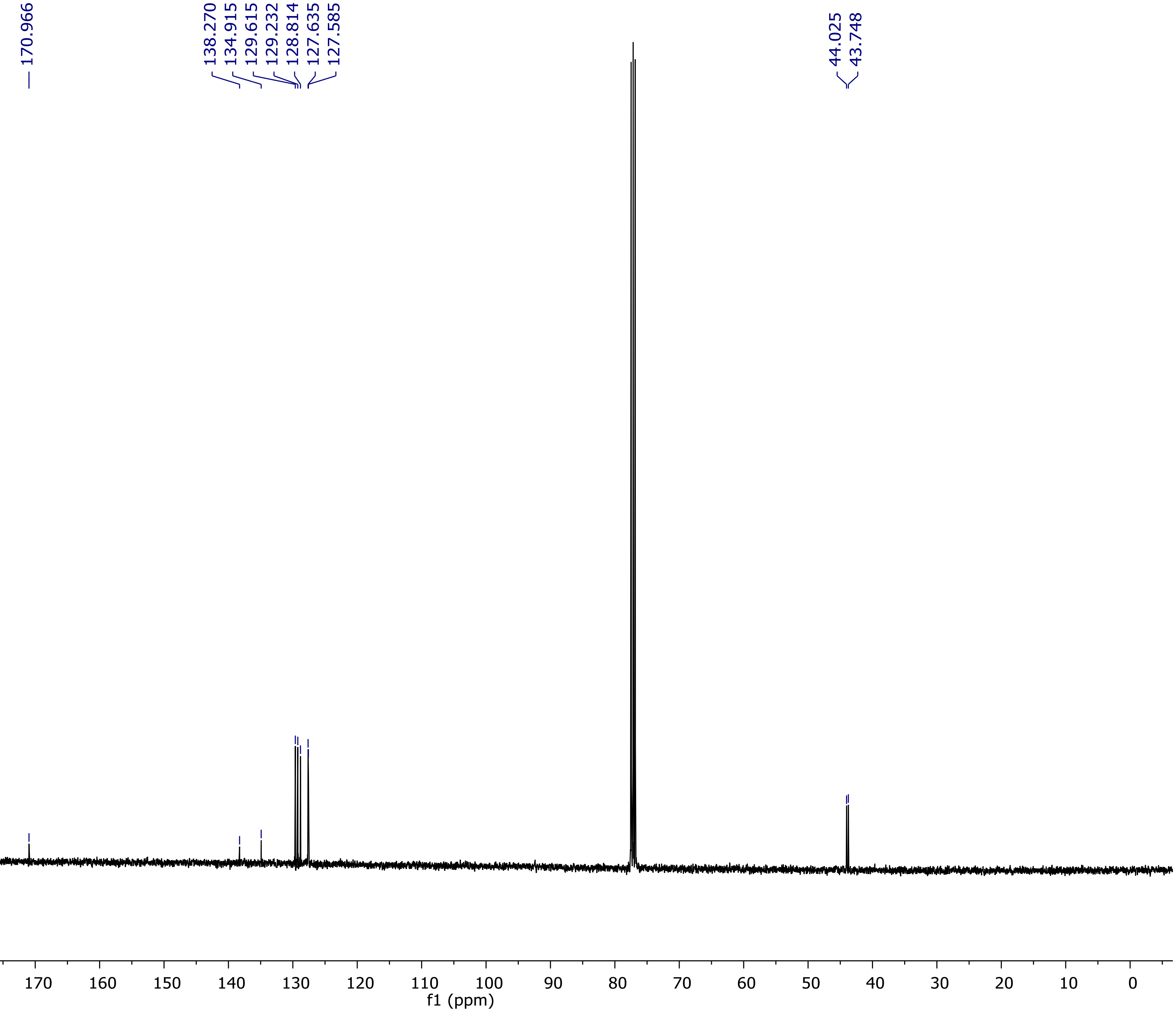

^{1}H NMR: 500 MHz, $CDCl_3$

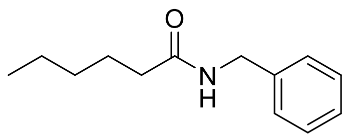

4m

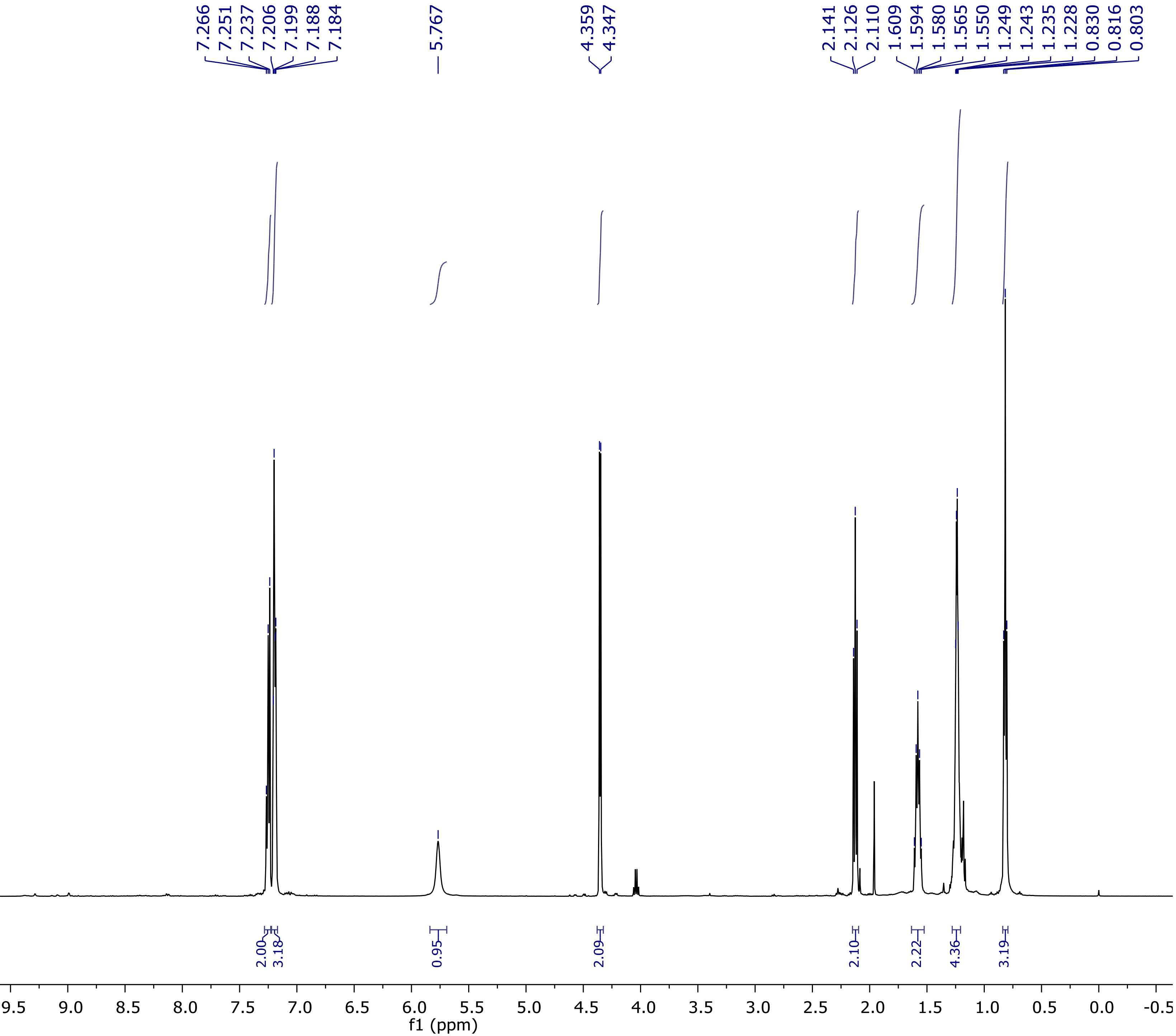

^{13}C NMR: 126 MHz, $CDCl_3$

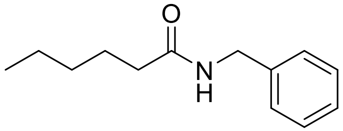

4m

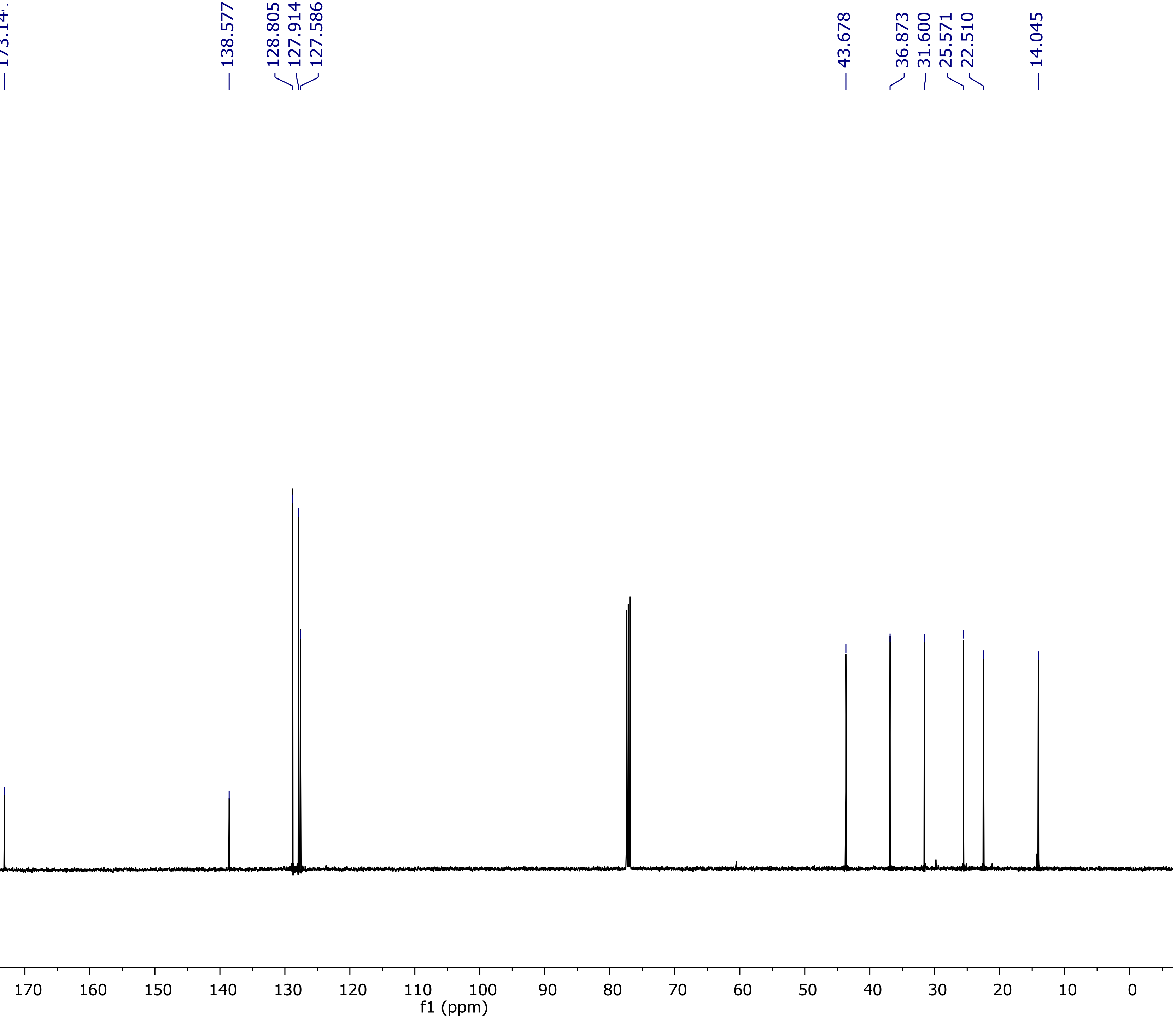

^{1}H NMR: 500 MHz, $CDCl_3$

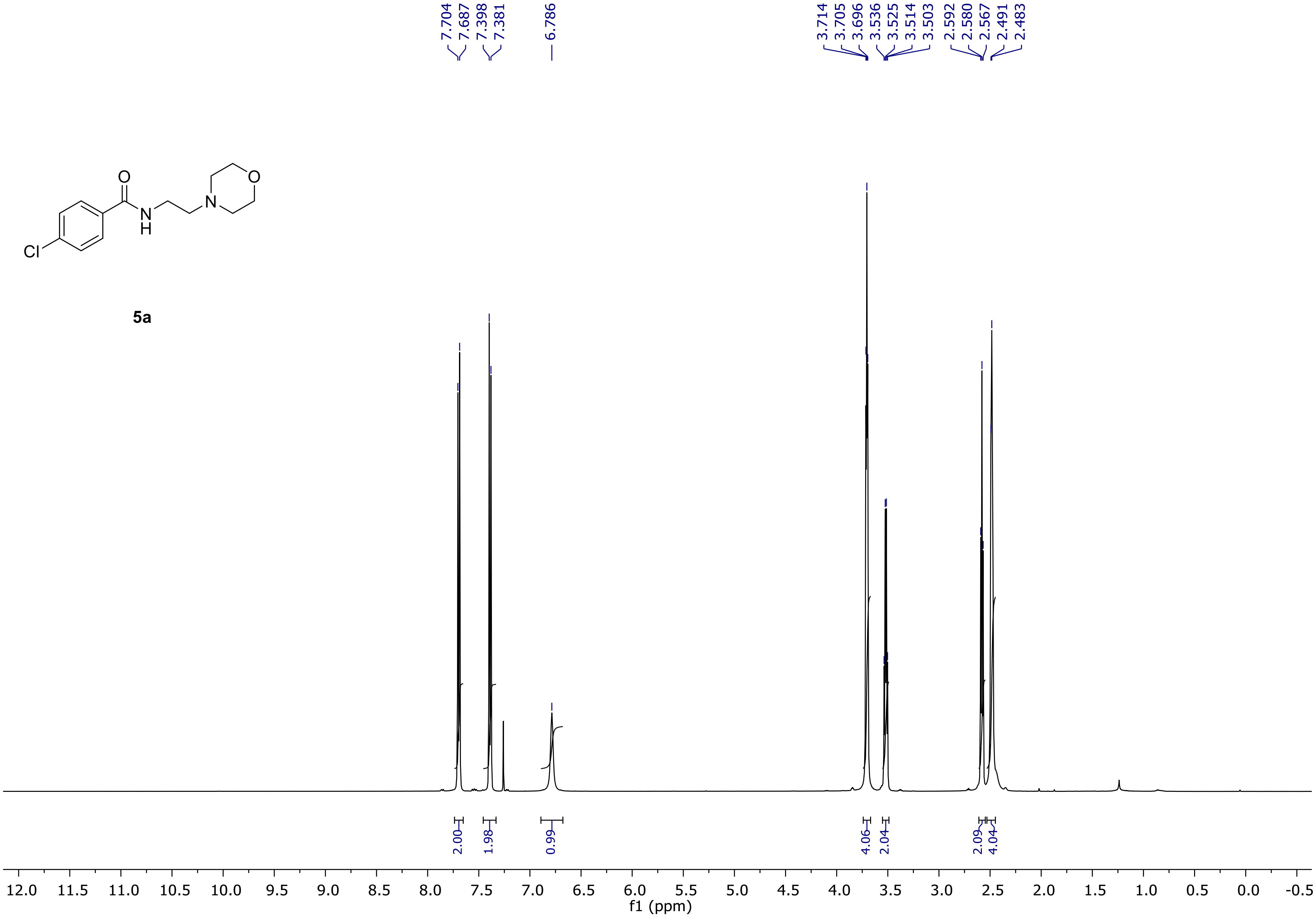

^{13}C NMR: 126 MHz, $CDCl_3$

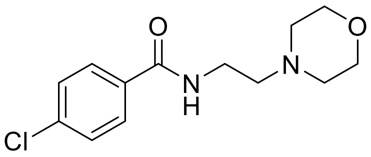

5a

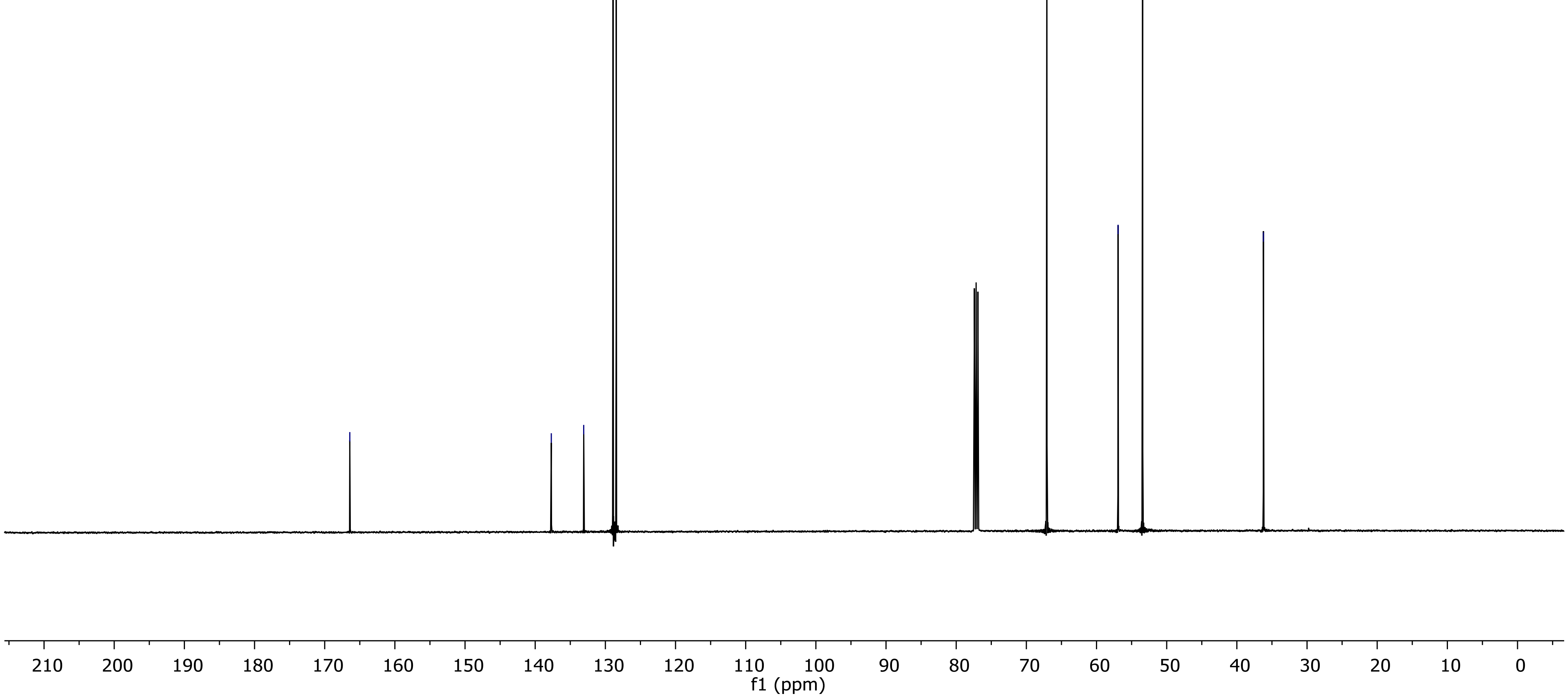

^1H NMR: 400 MHz, $CDCl_3$

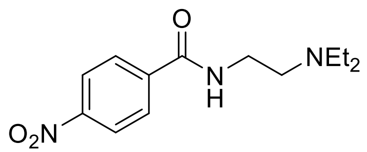

5b

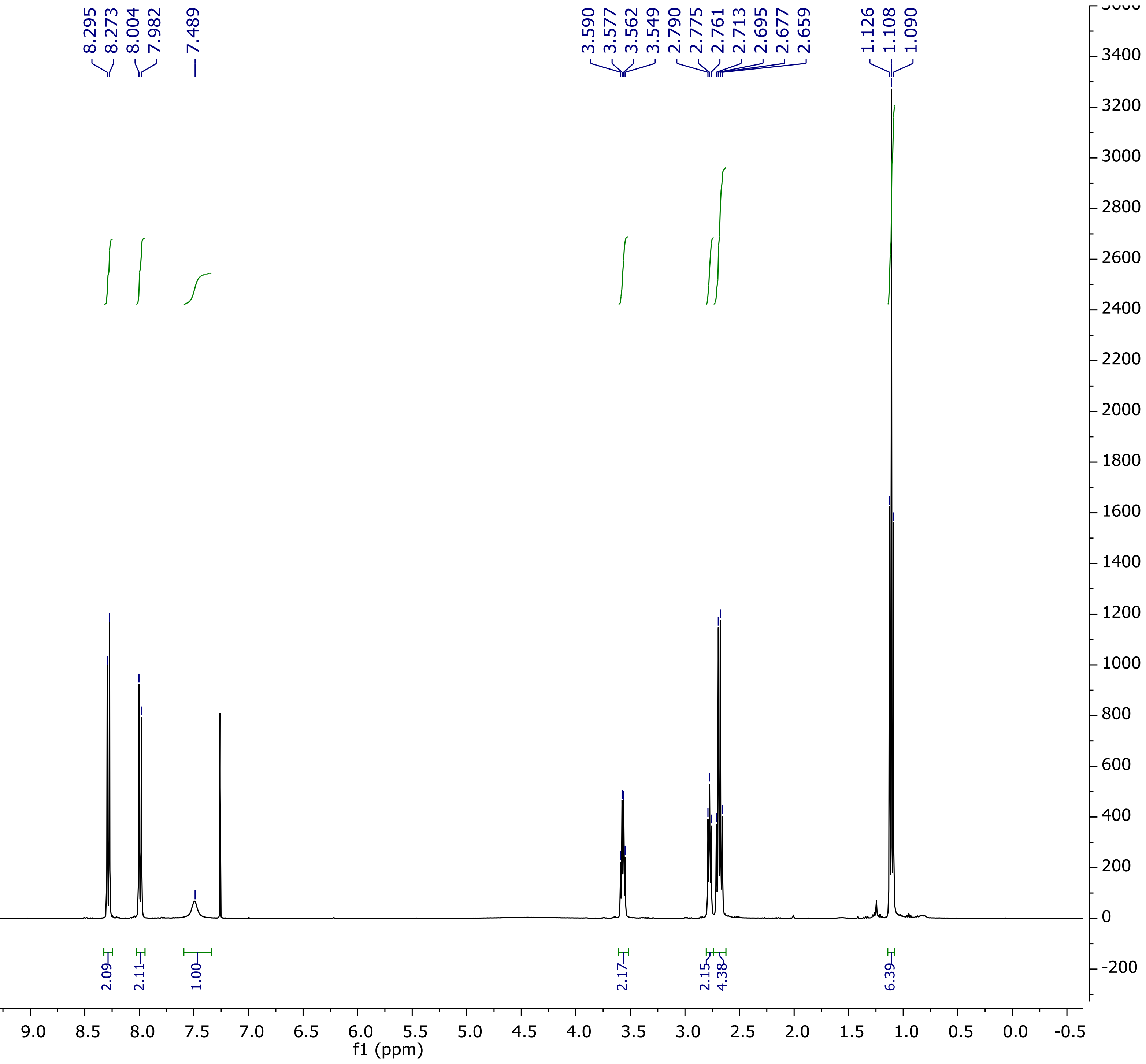

^{13}C NMR: 101 MHz, $CDCl_3$

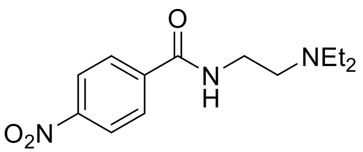

5b

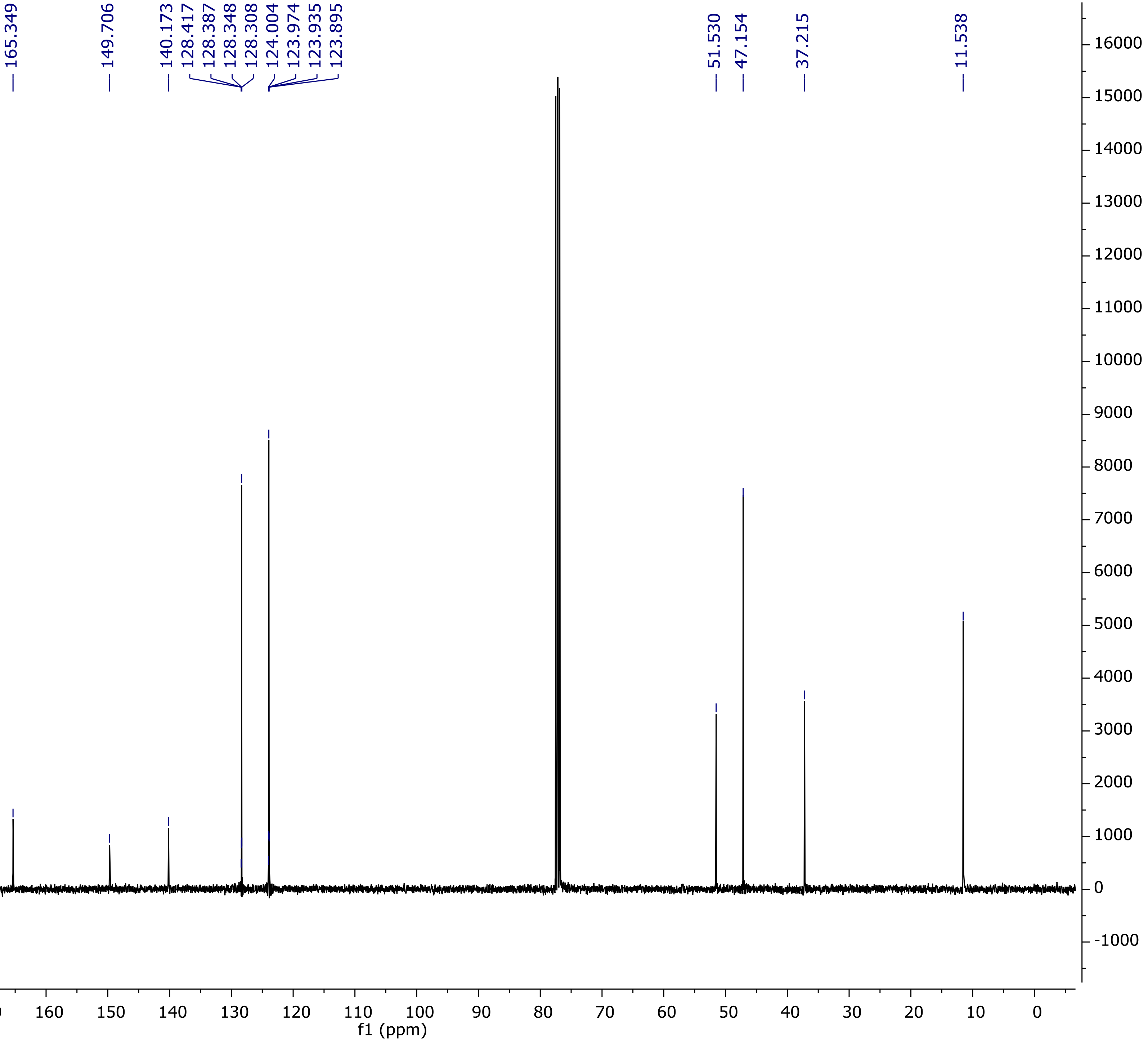

^{1}H NMR: 500 MHz, $CDCl_3$

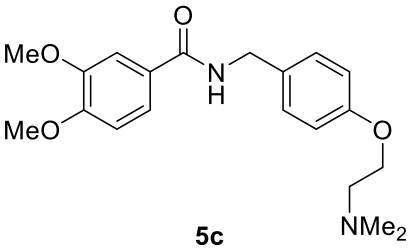

5c

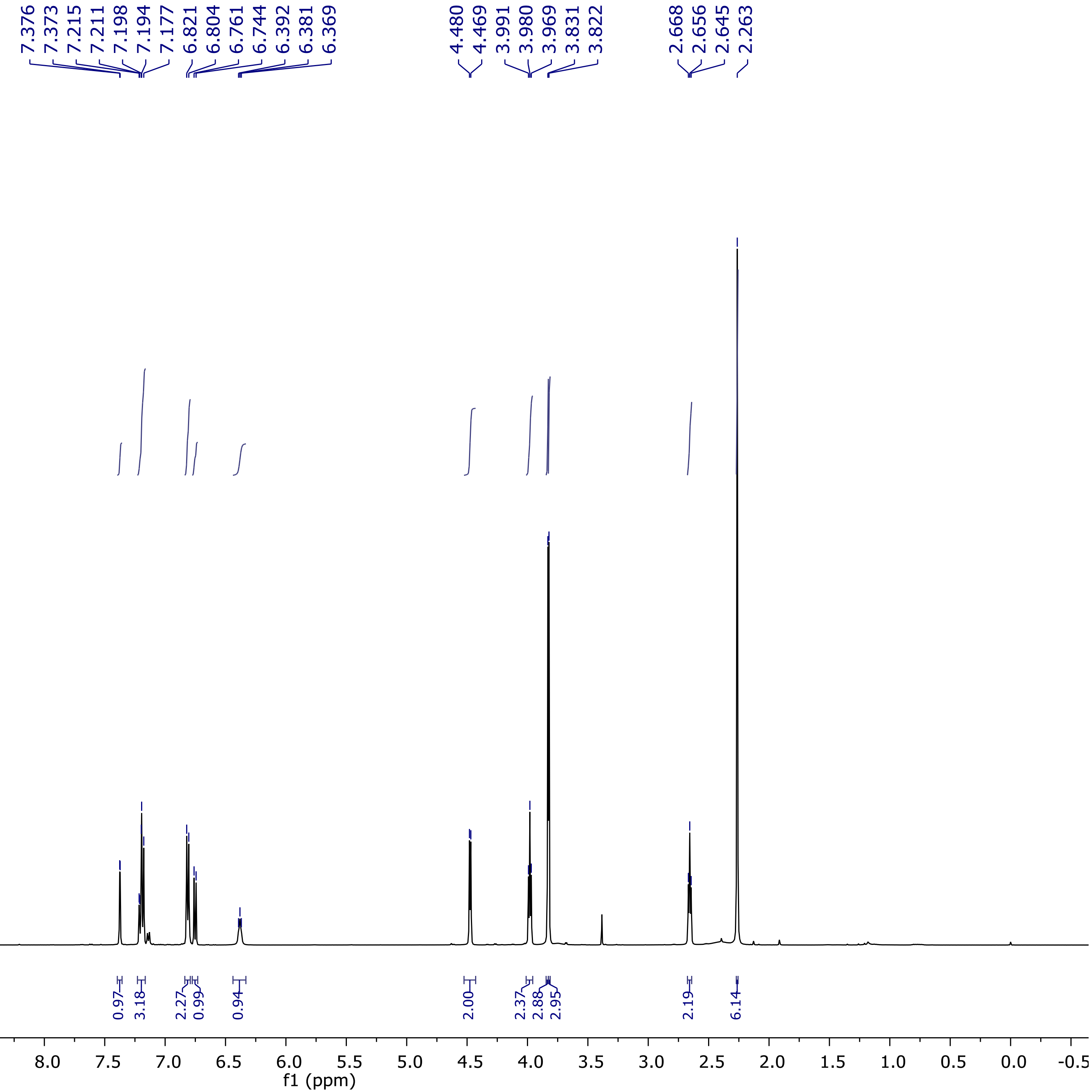

^{13}C NMR: 126 MHz, $CDCl_3$

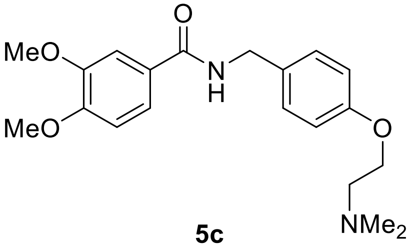

5c

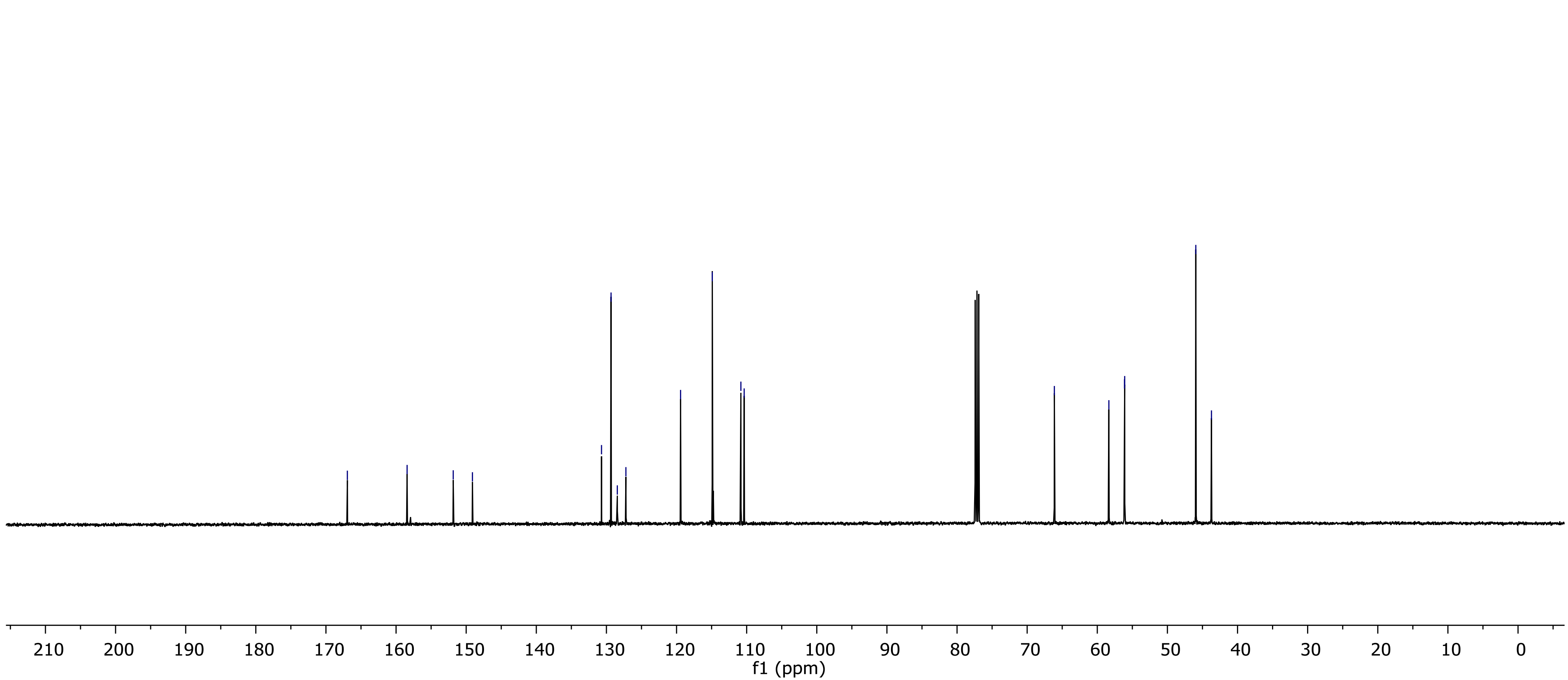

^{1}H NMR: 500 MHz, $CDCl_3$

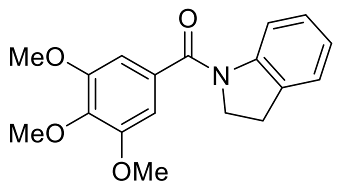

5d

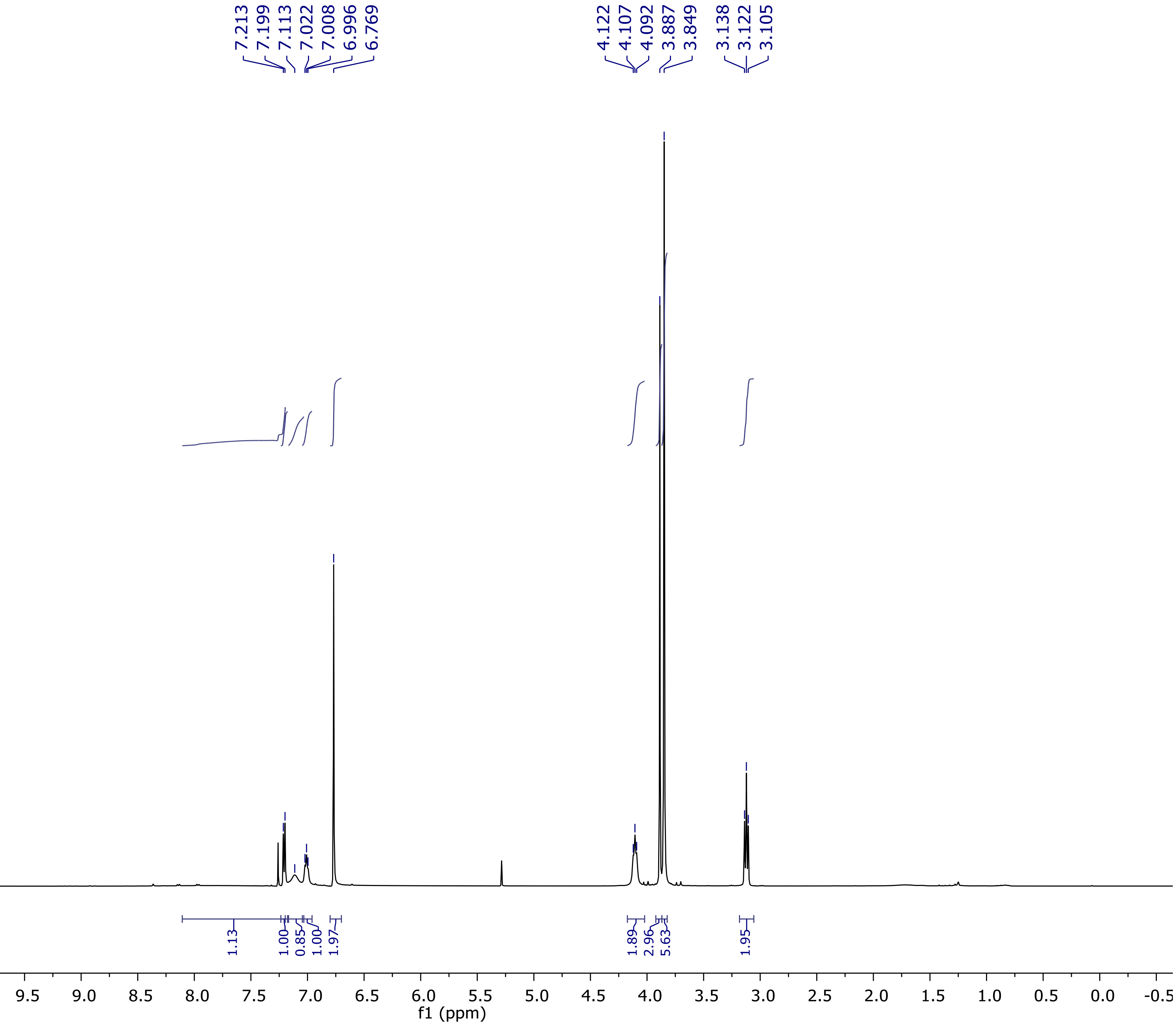

^{13}C NMR: 126 MHz, $CDCl_3$

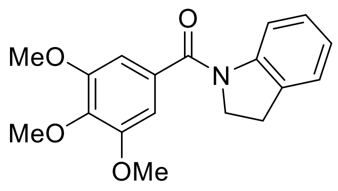

5d

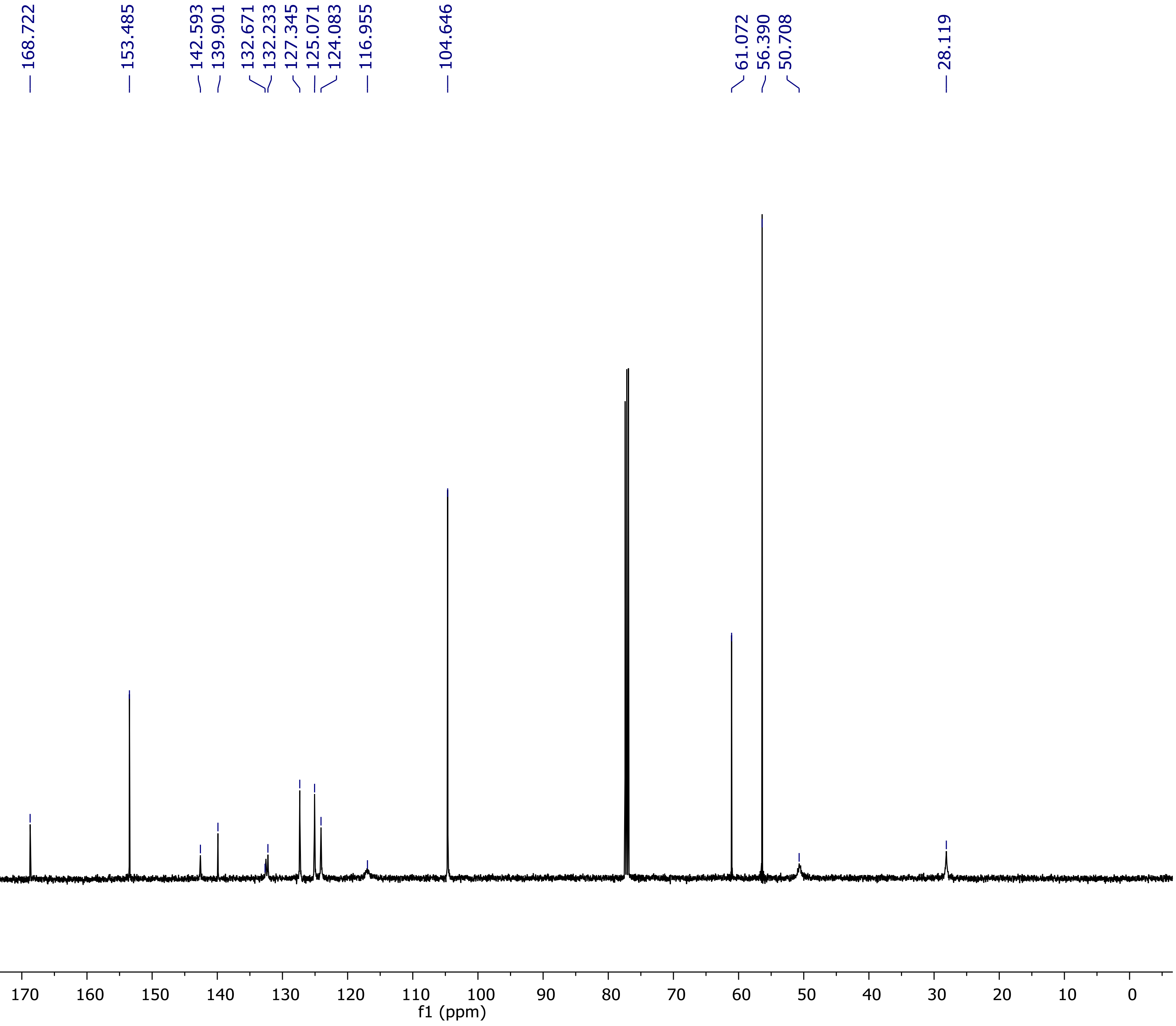

^1H NMR: 400 MHz, $CDCl_3$

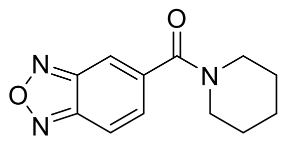

5e

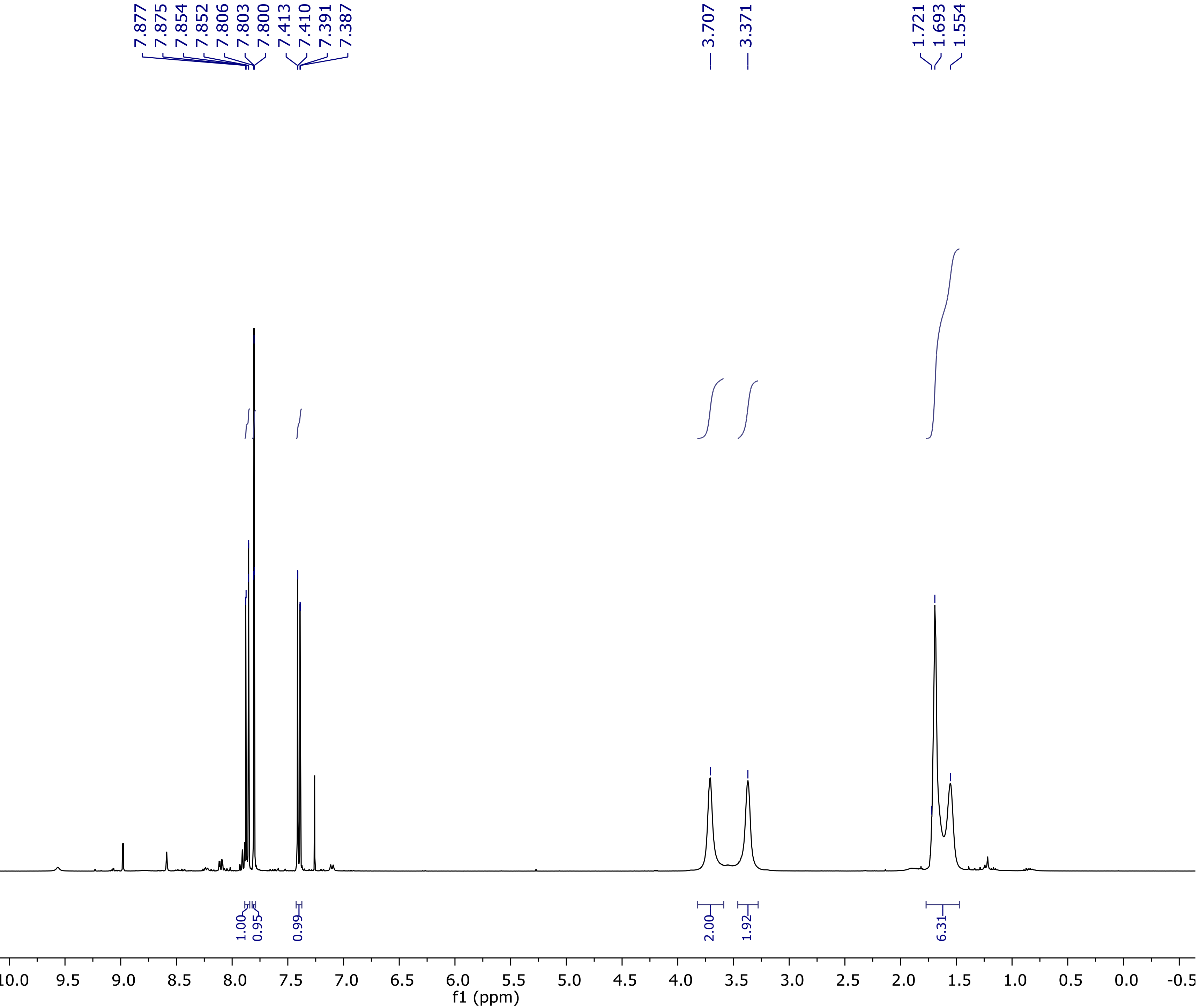

^{13}C NMR: 101 MHz, $CDCl_3$

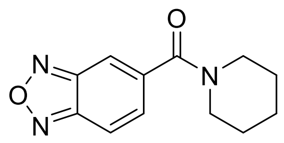

5e

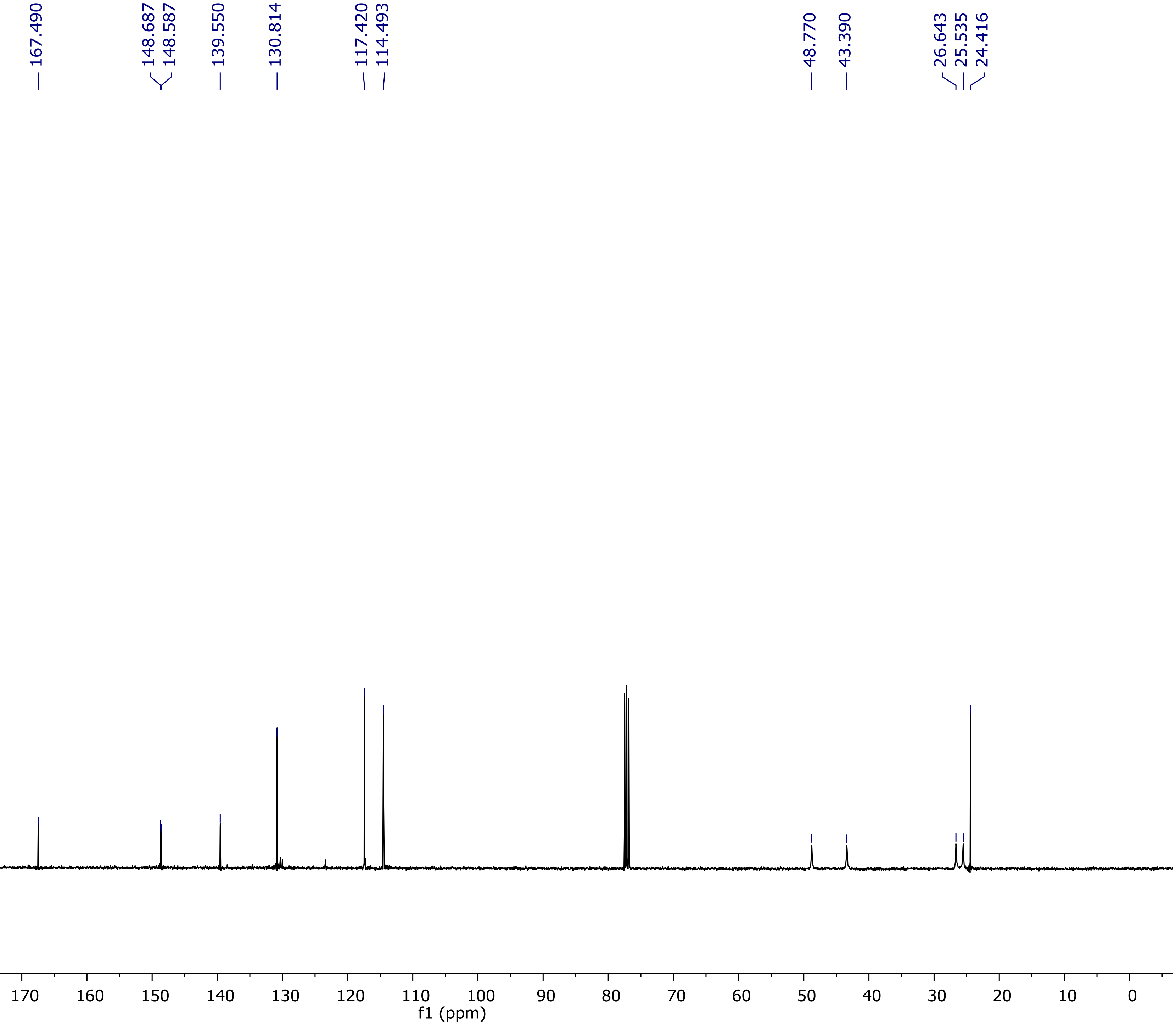

^{1}H NMR: 400 MHz, $CDCl_3$

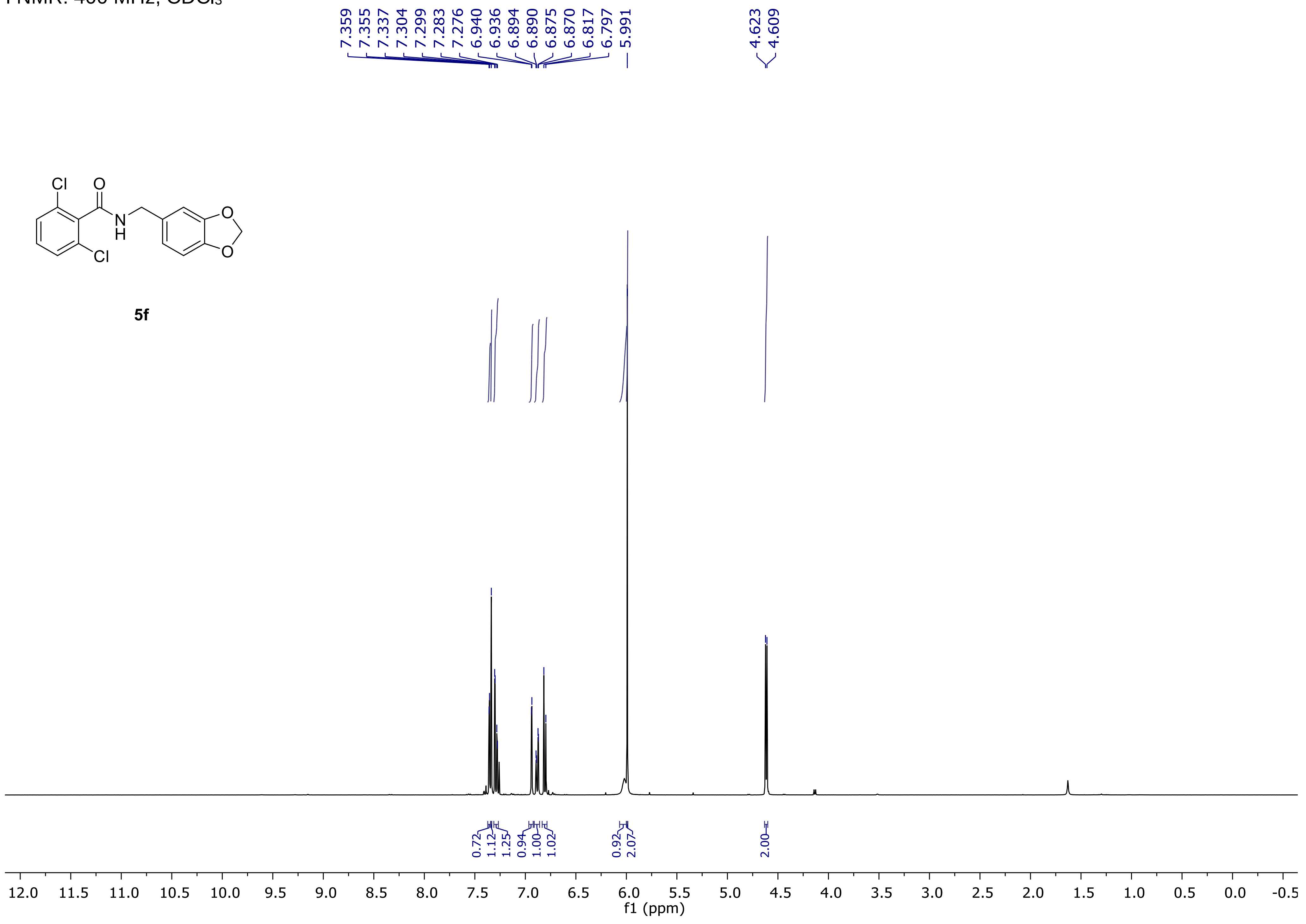

5f

^{13}C NMR: 101 MHz, $CDCl_3$

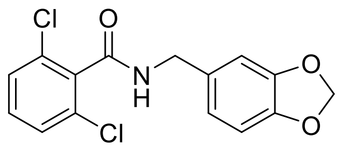

5f

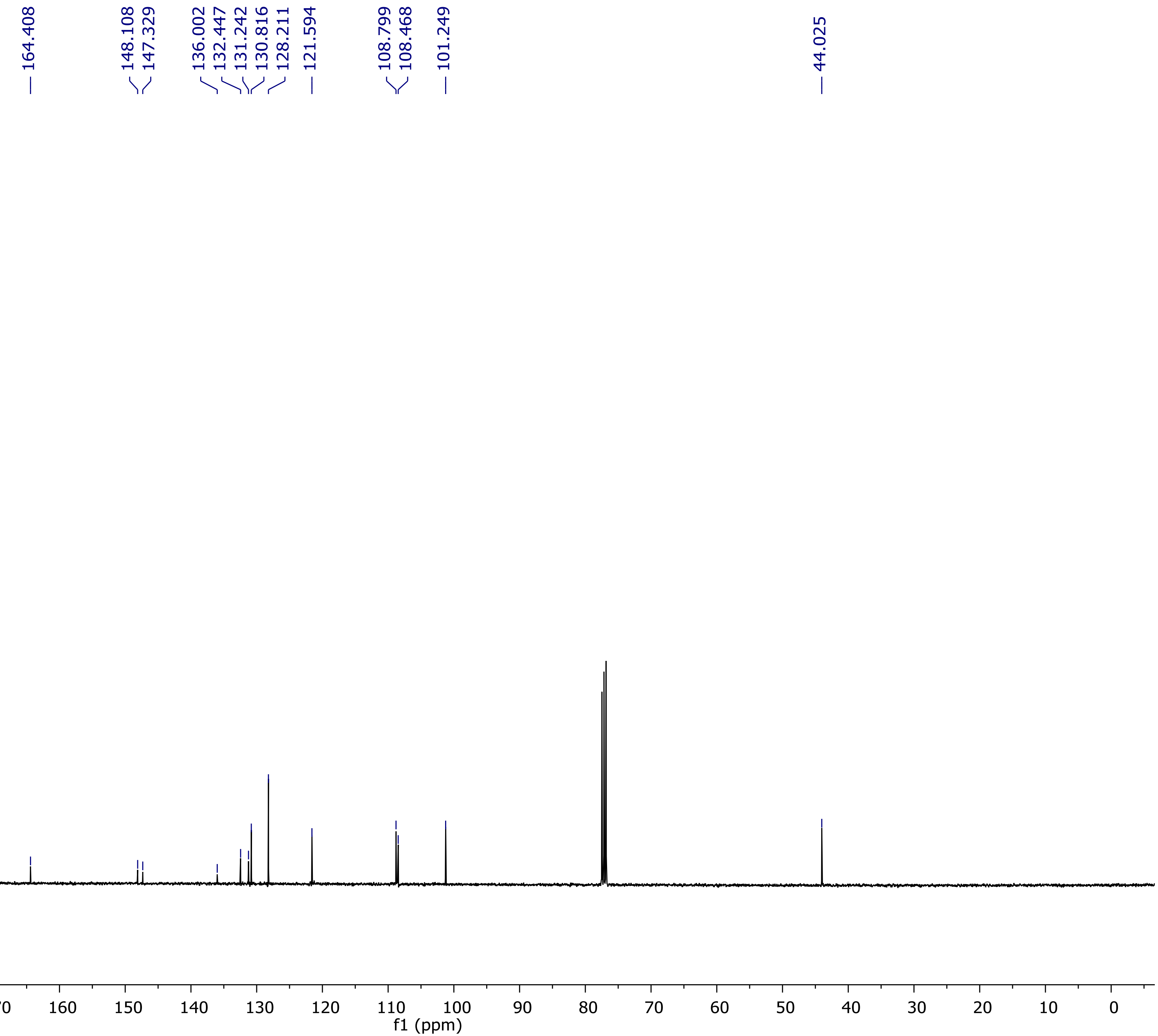

6. HPLC Reports for Peptides

Benzoyl-Leu-Enkephalin (8a)

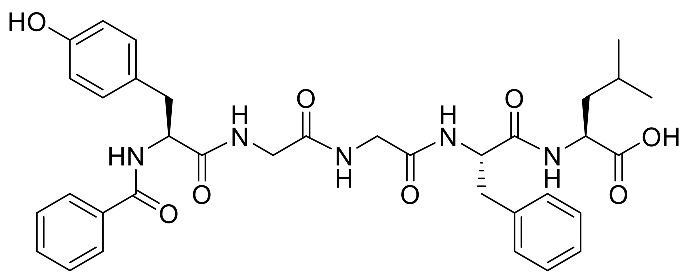

```
Acq. Operator : SYSTEM                                    Seq. Line : 2
Acq. Instrument : Timon HPLC                              Location : 39
Injection Date : 16/09/2022 12:22:34                            Inj : 1
                                                  Inj Volume : 5.000 µl
Method : c:\Chem32\1\Data\Jenna\Jenna 2022-11-11 16-10-49\KM_5_95_30MIN_
           1mlmin.M (Sequence Method)
Last changed : 11/11/2022 16:10:51 by SYSTEM
Additional Info : Peak(s) manually integrated
```

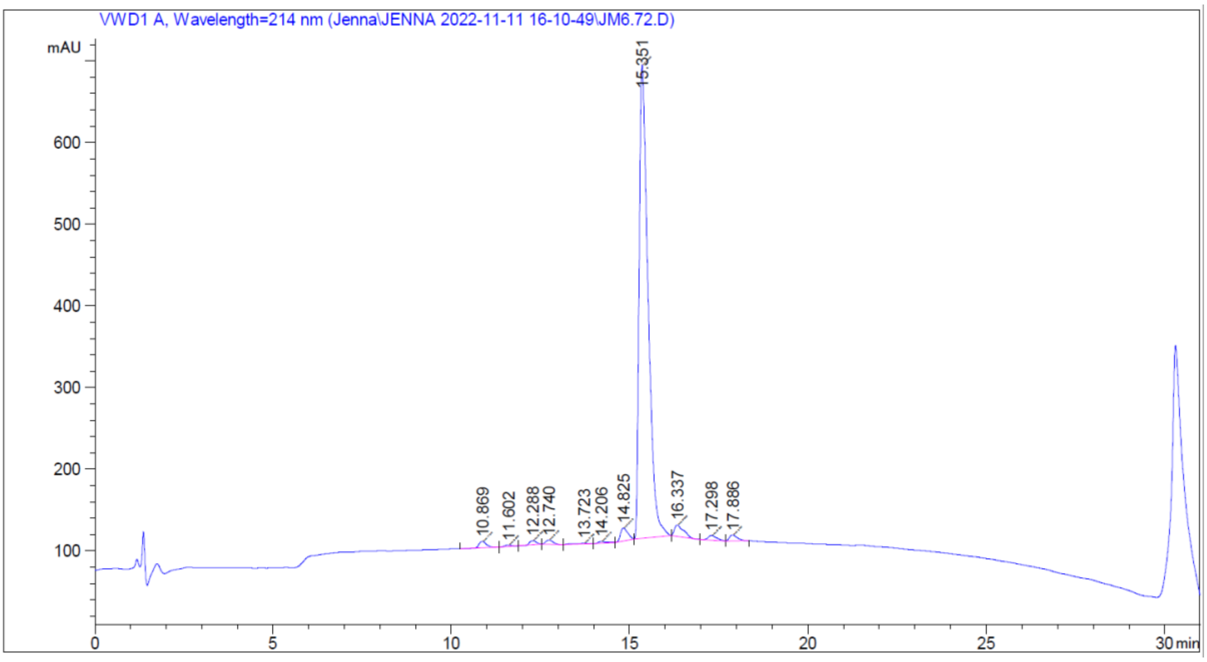


```
=====================================================================
                         Area Percent Report
=====================================================================
```

```
Sorted By : Signal
Multiplier : 1.0000
Dilution : 1.0000
Use Multiplier & Dilution Factor with ISTDs
```

Signal 1: VWD1 A, Wavelength=214 nm

Peak #	RetTime [min]	Type	Width [min]	Area [mAU*s]	Height [mAU]	Area %
1	10.869	MM	0.2348	111.91913	7.94440	1.0148
2	11.602	MM	0.2119	23.88828	1.87905	0.2166
3	12.288	MM	0.2108	60.12086	4.75347	0.5451
4	12.740	MM	0.2337	67.71255	4.82947	0.6140
5	13.723	MM	0.5769	31.64616	9.14313e-1	0.2870
6	14.206	MM	0.2724	30.71158	1.87924	0.2785
7	14.825	MM	0.2308	214.75410	15.50702	1.9473
8	15.351	MM	0.2887	1.00420e4	579.71729	91.0557
9	16.337	MM	0.2949	249.59697	14.10397	2.2632
10	17.298	MM	0.2733	90.39386	5.51152	0.8196
11	17.886	MM	0.2462	105.67006	7.15231	0.9582

Totals : 1.10284e4 644.19205

===

*** End of Report ***

(4-Methoxy-d_3)benzoyl-2,6-d_2-Leu-Enkephalin (8b) and (4-methoxy)benzoyl-Leu-Enkephalin (8c)

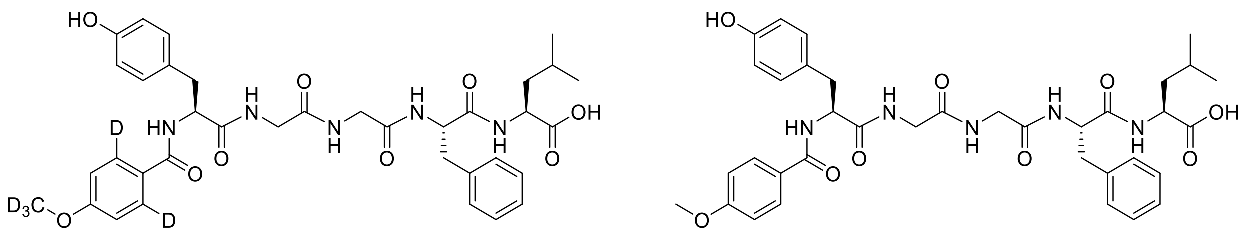

```
Acq. Operator : SYSTEM                                    Seq. Line : 2
Acq. Instrument : Timon HPLC                               Location : 39
Injection Date : 16/09/2022 12:22:34                            Inj : 1
                                                  Inj Volume : 5.000 µl
Method : c:\Chem32\1\Data\Jenna\Jenna 2022-09-16 11-48-47\KM_5_95_30MIN_
           1mlmin.M (Sequence Method)
Last changed : 16/09/2022 11:48:48 by SYSTEM
Additional Info : Peak(s) manually integrated
```

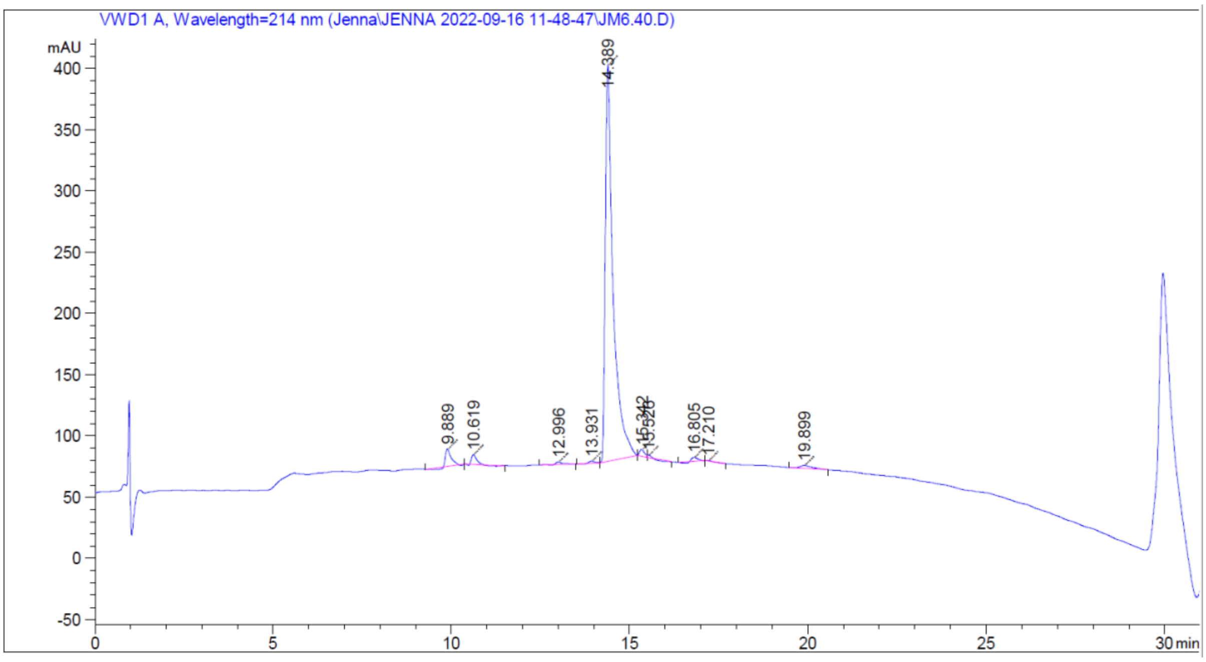

===

Area Percent Report

===

Sorted By : Signal
Multiplier : 1.0000
Dilution : 1.0000
Use Multiplier & Dilution Factor with ISTDs

Signal 1: VWD1 A, Wavelength=214 nm

Peak #	RetTime [min]	Type	Width [min]	Area [mAU*s]	Height [mAU]	Area %
1	9.889	MM	0.1870	160.53792	14.30820	2.9621
2	10.619	MM	0.1773	87.60984	8.23721	1.6165
3	12.996	MM	0.2178	26.22959	2.00714	0.4840
4	13.931	MM	0.1899	21.01335	1.84454	0.3877
5	14.389	MM	0.2555	4961.93652	323.62619	91.5528
6	15.342	MF	0.1627	55.67168	5.70444	1.0272
7	15.526	FM	0.1146	14.34213	2.08537	0.2646
8	16.805	MM	0.1971	37.25498	3.15070	0.6874
9	17.210	MM	0.1169	4.35017	6.20377e-1	0.0803
10	19.899	MM	0.3870	50.80820	2.18835	0.9375
Totals :				5419.75438	363.77252	

==

*** End of Report ***

Benzoyl-Substance P (9)

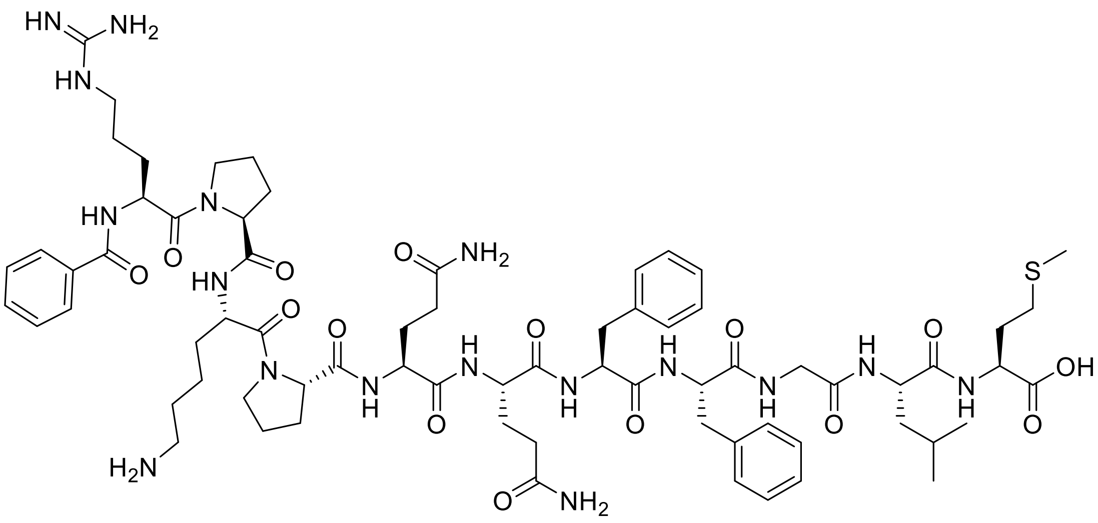

Acq. Operator : SYSTEM   Seq. Line : 2
Acq. Instrument : Timon HPLC   Location : 92
Injection Date : 10/11/2022 09:58:53   Inj : 1
                                                  Inj Volume : 5.000 µl
Method : c:\Chem32\1\Data\Jenna\Jenna 2022-11-10 09-25-05\KM_5_95_30MIN_
         1mlmin.M (Sequence Method)
Last changed : 10/11/2022 09:25:07 by SYSTEM
Additional Info : Peak(s) manually integrated

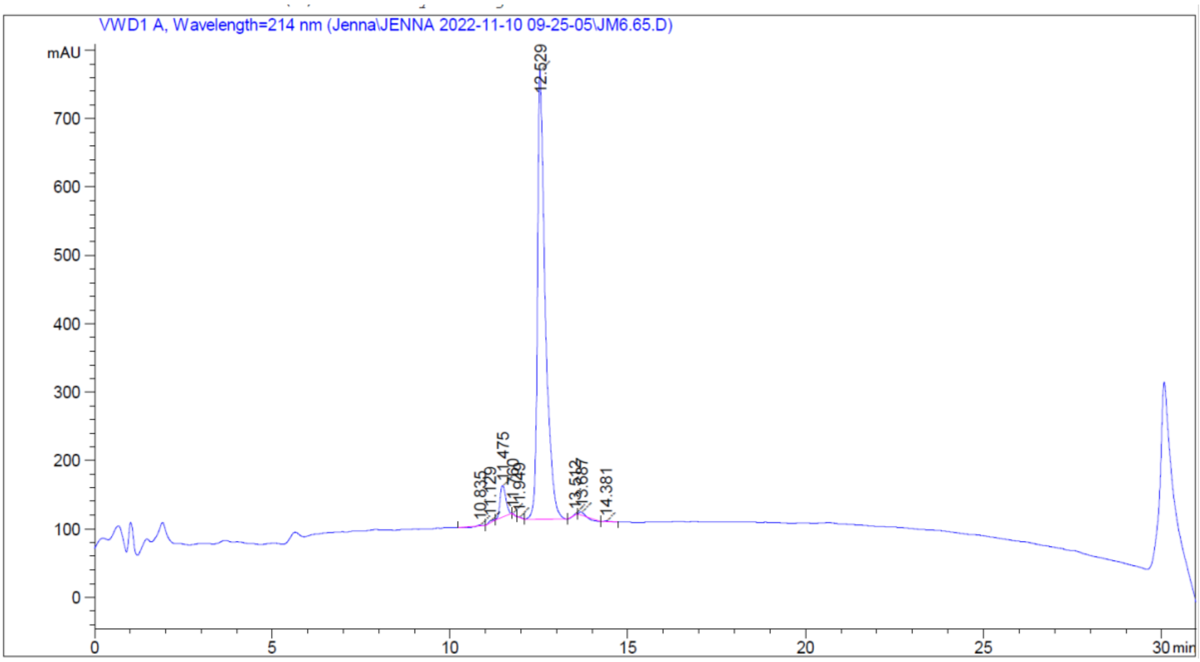

===

Area Percent Report

===

Sorted By : Signal
Multiplier : 1.0000
Dilution : 1.0000
Use Multiplier & Dilution Factor with ISTDs

Signal 1: VWD1 A, Wavelength=214 nm

Peak #	RetTime [min]	Type	Width [min]	Area [mAU*s]	Height [mAU]	Area %
1	10.835	MM	0.1618	11.16052	1.14942	0.1042
2	11.129	MM	0.1492	25.93047	2.89661	0.2421
3	11.475	MM	0.1866	514.33069	45.94299	4.8027
4	11.760	MM	0.0601	5.61843	1.55706	0.0525
5	11.949	MM	0.1723	9.34132	9.03675e-1	0.0872
6	12.529	MM	0.2555	1.00737e4	657.12372	94.0662
7	13.512	MM	0.0886	8.66315	1.62913	0.0809
8	13.687	MM	0.1547	44.14020	4.75648	0.4122
9	14.381	MM	0.2372	16.27405	1.14363	0.1520

Totals : 1.07092e4 717.10272

===

*** End of Report ***
